# Charge-enhanced pyri­dyl tri­fluoro­borate organocatalysts: crystal structures and reactivity

**DOI:** 10.1107/S2053229625010629

**Published:** 2026-01-01

**Authors:** Alex Lovstedt, Stephen H. Dempsey, Steven Kass

**Affiliations:** a Department of Chemistry, University of Minnesota, 207 Pleasant St SE, Minneapolis, Minnesota 55455, USA; Universidade Federal de Minas Gerais, Brazil

**Keywords:** organocatalysts, crystal structure, charge-enhanced reactivity, pyridyl tri­fluoro­borate salt, organic cation

## Abstract

The crystal structures of eleven 3- and 4-pyri­dyl borate salts with a variety of cations are reported. These are potassium tri­fluoro­(pyridin-3/4-yl)borate, K^+^·C_5_H_4_BF_3_N^−^, **1** and **2** (as the monohydrate), tetra­butyl­ammonium tri­fluoro­(pyridin-3/4-yl)borate, C_16_H_36_N^+^·C_5_H_4_BF_3_N^−^, **3** and **4**, tetra­phenyl­phospho­nium tri­fluoro­(pyridin-3/4-yl)borate, C_24_H_20_P^+^·C_5_H_4_BF_3_N^−^, **5** and **6**, tetra­kis­(3,5-di­meth­oxy­phen­yl)phospho­nium tri­fluoro­(pyridin-3/4-yl)borate, C_32_H_36_O_8_P^+^·C_5_H_4_BF_3_N^−^, **7** and **8**, and tetra­kis­[4-(di­methyl­amino)­phen­yl]phospho­nium tri­fluoro­(pyridin-3/4-yl)borate, C_32_H_40_N_4_P^+^·C_5_H_4_BF_3_N^−^, **9** and **10**, and the hemihydrate, C_32_H_40_N_4_P^+^·C_5_H_4_BF_3_N^−^·0.5H_2_O, **10h**. The effects of the position of the pyridine N atom and the identity of the cations on the crystal packing of the salts are examined. The N⋯H and F⋯H anion–cation con­tacts and the Hirshfeld surface com­positions of the anions for the salts containing organic cations are com­pared to the reactivity of the salts in a low-polarity solvent to look for trends between solid-state features and solution-state reactivity.

## Abstract 2

An examination of the relationship between the solid-state features of the crystal structures of eleven 3- and 4-pyridyl tri­fluoro­borate organocatalysts and their solution-state reactivity is reported.

## Introduction

When referring to a catalyst for a chemical reaction, often the first type of species to come to mind is a transition metal. Substances of this sort play an enormous role in modern chemistry, ranging from applications from polymer synthesis to protein modification to the generation of vital chemical feedstocks such as ammonia (Wang *et al.*, 2025 ▸; Ghosh, 2025 ▸; Varadwaj *et al.*, 2025 ▸). Despite their ubiquity, transition-metal catalysts are not without their drawbacks. First, many of them require the use of scarce late transition metals, such as palladium or ruthenium, in order to function. As a result, they are costly to manufacture and expensive to purchase. Significant work has gone into their replacement with abundant early transition metals, such as nickel or titanium, as more sus­tain­able and ‘greener’ alternatives (Sun *et al.*, 2025 ▸; Butler *et al.*, 2024 ▸). Unfortunately, many of these species still suffer from a major drawback of transition-metal catalysts: air and moisture sensitivity. The impressive chemical reactions enabled by transition-metal catalysts are often made more difficult, especially at large scale, by the need to operate under strictly inert conditions. Thus, there is a demand for catalysts that are stable under ambient conditions.

In the last few decades, transition-metal-free catalysts that use only organic mol­ecules to catalyze chemical reactions have been developed for a variety of transformations (List, 2007 ▸). These catalysts, known as organocatalysts, tend to be more tolerant of air and moisture than transition-metal catalysts. However, they are often less catalytically active and therefore require higher catalyst loadings to achieve reasonable reaction rates. For example, chiral amine organocatalysts, such as (*S*)-proline, may require loadings of up to 40%, while transition-metal catalysts, such as the ruthenium-based family of Grubb’s catalysts, often operate with catalyst loadings of less than 1% (Mukherjee *et al.*, 2007 ▸; Kajetanowicz & Grela, 2021 ▸).

**Figure 1:**
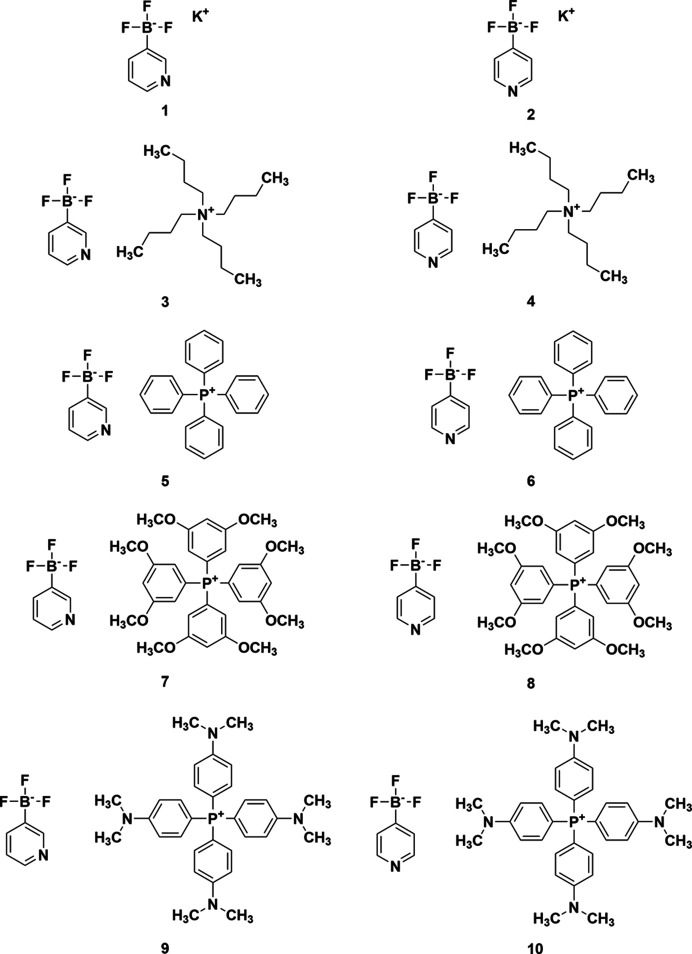
Pyridyl tri­fluoro­borate salts investigated in this work.

Our group has demonstrated that the addition of positively charged centers to Brønsted acid organocatalysts can significantly increase their catalytic activity (Fan & Kass, 2016 ▸; Samet *et al.*, 2015 ▸; Ma & Kass, 2016 ▸; Riegel *et al.*, 2022 ▸). It follows that the addition of a negatively charged center could increase the activity of Brønsted bases or nucleophilic catalysts. The latter, derived from a pyridine scaffold such as 4-(di­methyl­amino)­pyridine (DMAP), have been used as catalysts for a wide range of reactions, including esterifications (Neises & Steglich, 1978 ▸), macrolactonizations (Boden & Keck, 1985 ▸), and silylations (Chaudhary & Hernandez, 1979 ▸). Given the widespread applications of nucleophilic catalysts, the development of more reactive analogues by incorporation of a negative charge center could potentially increase their effectiveness. Recent work has demonstrated the validity of anion-enhanced nucleophilic catalysts with the development of anionic sulfonamide-based catalysts (Helberg *et al.*, 2020 ▸; Burger, Franta, O’Donoghue *et al.*, 2025 ▸; Burger, Franta, Ofial *et al.*, 2025 ▸) that showed significant improvement in reactivity over DMAP and related neutral pyridine derivatives. Re­cent­ly, our group has developed a similar family of negatively charged organocatalysts based on a pyri­dyl borate scaf­fold (Dempsey, Lovstedt *et al.*, 2023 ▸; Dempsey, Cao *et al.*, 2023 ▸). These were found to have increased nucleophilicity com­pared to DMAP, and the most reactive of which was com­parable in performance to the anionic sulfonamide catalysts (Dempsey, Lovstedt *et al.*, 2023 ▸). Following on from our previous studies, in this article, we report the crystal structures of ten different 3- or 4-pyri­dyl tri­fluoro­borate salts (Fig. 1 ▸). Some of the salts had been investigated as anionic catalysts in our previous work, while several novel salts have been synthesized and characterized. Using the crystal structures, we examine the inter­molecular inter­actions between the ion pairs of the various salts and search for trends between selected crystallographic parameters and anion nucleophilicity. Additionally, the effects of counter-ion identity on the nucleophilicity of the catalysts will be examined.

## Experimental

### Synthesis

**Figure 2:**
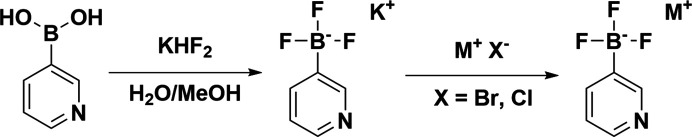
The synthetic pathway for the 3-pyri­dyl tri­fluoro­borate salts. The 4-pyri­dyl salts were synthesized in an analogous manner.

The 3- and 4-pyri­dyl tri­fluoro­borate salts were synthesized as illustrated in Fig. 2 ▸ for the 3-substituted derivatives (Dempsey, Lovstedt *et al.*, 2023 ▸). Potassium bifluoride was reacted with one of the two different pyri­dyl boronic acids to give the corresponding pyri­dyl tri­fluoro­borate anion as its potassium salt. Cation exchange *via* ion metathesis afforded the desired salts. Single crystals of each were grown by evaporation (see the supporting information for specific details). These com­pounds were also characterized by NMR spectroscopy (^1^H, ^13^C, ^11^B, ^19^F, and ^31^P) and high-resolution mass spectrometry. The results of these analyses are provided in the supporting information.

### Crystal structure, data collection and refinement

Crystals were placed onto the tip of a 0.15 mm MiTeGen loop and mounted on a Bruker D8 Venture diffractometer equipped with a PHOTON III CPAD detector and IµS 3.0 Mo and IµS 2.0 Cu microfocus X-ray sources for data collection. A preliminary set of unit-cell constants was calculated from reflections harvested from three sets of frames. These initial sets of frames were oriented such that orthogonal wedges of reciprocal space were surveyed. This produced initial orientation matrices which were used to determine a data collection strategy to ensure com­plete data coverage to a desired resolution using the *APEX5* software package (Bruker, 2023 ▸). The data collection was carried out using Cu *K*α radiation for com­pounds **3** and **4**, and Mo *K*α radiation for com­pounds **1**, **2**, **5**–**10**, and **10h** (the hemihydrate of **10**). All major sections of frames were collected with 1.2° steps in ω or φ at different detector positions in 2θ. The intensity data were corrected for absorption and decay using the *SADABS* or *TWINABS* software (Krause *et al.*, 2015 ▸; Sheldrick, 2012 ▸). Final unit-cell constants were calculated from the *xyz* centroids of strong reflections from the actual data collection after integration in the *SAINT* software (Bruker, 2016 ▸). The structures were solved using *SHELXT* (Sheldrick, 2015*a* ▸) and refined using *SHELXL2019* (Sheldrick, 2015*b* ▸) within the *ShelXle* program (Hübschle *et al.*, 2011 ▸). Space groups were determined based on systematic absences and intensity statistics. A dual-space method solution was calculated which provided most non-H atoms from the E-map. Full-matrix least-squares/difference Fourier cycles were performed which located the remaining non-H atoms. All non-H atoms were refined with anisotropic displacement parameters. H atoms on water mol­ecules were located in the difference map. All other H atoms were placed in ideal positions and refined as riding atoms with relative isotropic displacement parameters. Crystal structure images were created using *Mercury* (Macrae *et al.*, 2020 ▸). Crystal information and refinement details for each structure are provided in Table 1 ▸.

### Hirshfeld surface analysis

Hirshfeld surfaces and fingerprint plots for the crystal structures of com­pounds **3**–**10** and **10h** were com­puted using the *CrystalExplorer* program (Spackman *et al.*, 2021 ▸).

### S_N_2 reaction kinetics

Data were collected using a Bruker Avance 400 MHz spectrometer. In a nitro­gen-atmosphere glovebox, an amount of each salt to make a 25 m*M* solution and iodo­octane to make a 500 m*M* solution were dissolved in deuterated di­chloro­methane (DCM) and placed in a screw cap NMR tube. The tube was inverted several times to ensure mixing, placed in the NMR spectrometer, and ^1^H spectra were taken at inter­vals ranging from 10 to 20 min. Pseudo-first-order rate constants were obtained using the *Reaction Monitoring* program within the *MNova* software package (Mestrelab Research, 2023 ▸). Additional experimental details are provided in the sup­por­ting information.

## Crystal structure analysis

This section will analyze and discuss the crystal packing of the various salts with a particular focus on close con­tacts between the F and N atoms of the anions with H atoms of the cations. Additionally, the effects of the counter-ion and the position of the pyridine N atom in the anion on the packing of the anions will be examined. A close con­tact is defined as a distance between atoms less than the sum of their van der Waals radii. The van der Waals radii used in this work are as follows: H 1.10 Å, C 1.70 Å, N 1.55 Å, O 1.52 Å, and F 1.47 Å (Mantina *et al.*, 2009 ▸). Close con­tacts involving H atoms are defined after normalizing the H-atom bond lengths to the distances observed in neutron diffraction: 1.089 Å for C—H bonds and 0.993 Å for O—H bonds. The standard uncertainties of con­tacts involving H atoms constrained as riding atoms are the uncertainties in the distance between the acceptor atom and the donor atom upon which the H atom is riding. The uncertainties for average distances are the average of the uncertainties of the individual measurements. The terms *ortho*, *meta*, and *para* are used to refer to the positions of aromatic H atoms in relation to the P atom for cations and the B atom for anions.

### Potassium salts

**Figure 3:**
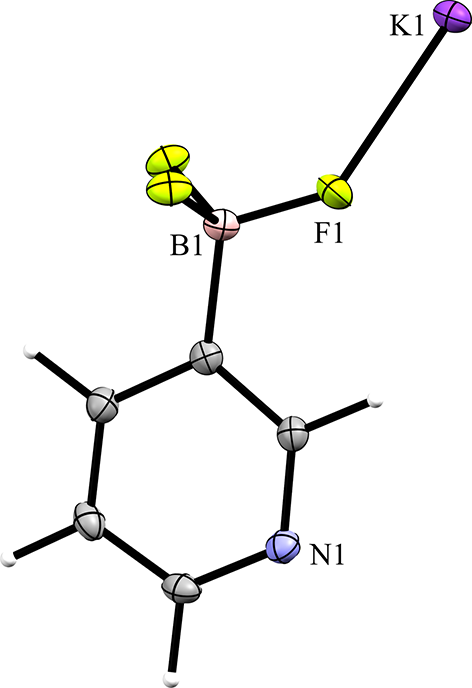
The asymmetric unit of salt **1**. The following applies to all images of crystal structures: non-H atoms are drawn as displacement ellipsoids at the 50% probability level. H atoms are drawn as fixed sized spheres.

**Figure 4:**
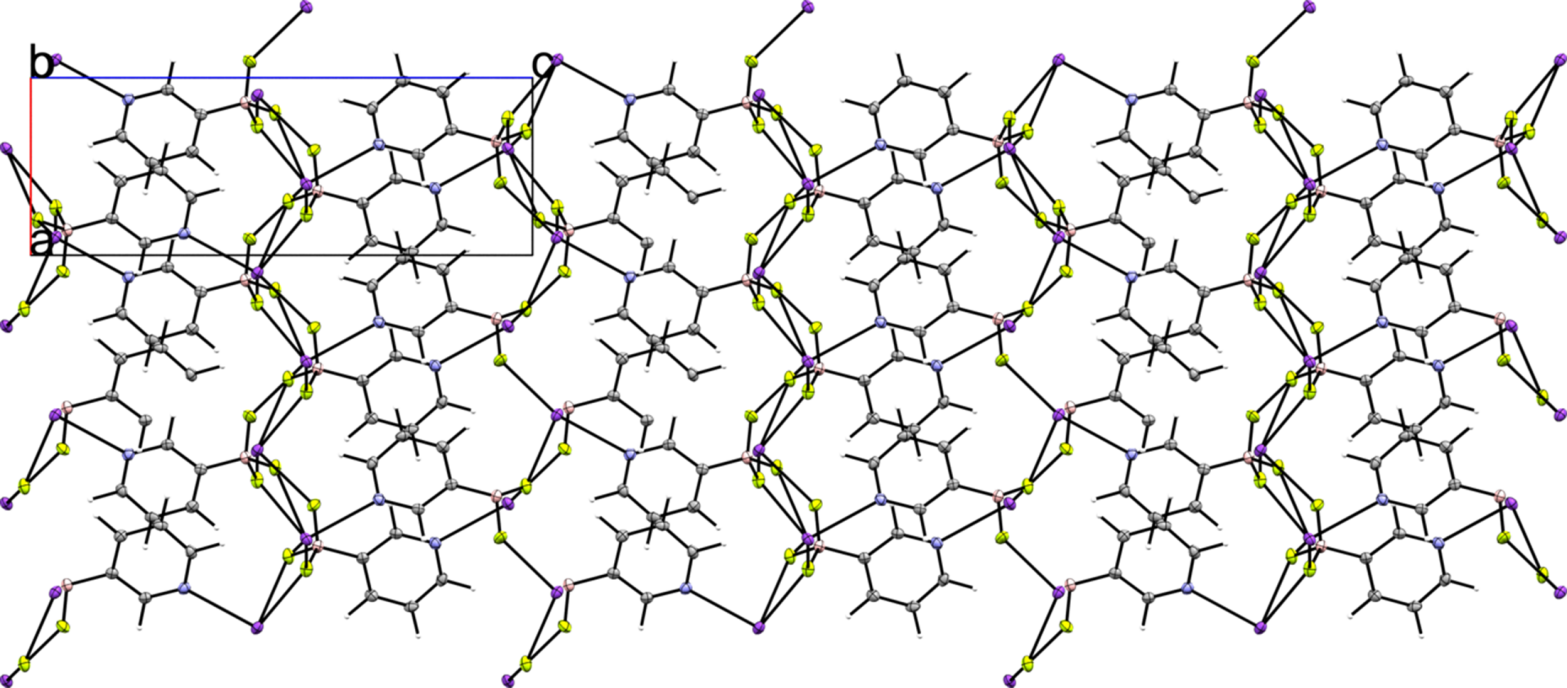
A 3×3×3 crystal packing diagram of salt **1**, viewed along the crystallographic *b* axis.

Compounds **1** and **2** are the potassium salts of the 3-pyri­dyl and 4-pyri­dyl tri­fluoro­borate anions, respectively. Salt **1** crystallized in the ortho­rhom­bic space group *P*2_1_2_1_2_1_ with *Z*′ = 1 (Fig. 3 ▸). The F atoms of the anion inter­act with the potassium cation, forming a layered structure in which layers of tri­fluoro­borate and potassium cations inter­act, separated by layers of pyridine moieties. This layered structure can be clearly seen when viewing a packing diagram of the salt along the crystallographic *a* and *b* axes (Fig. 4 ▸). The pyridine N atom links the two layers *via* an inter­action with a potassium cation in the next layer. The anions form anion–anion con­tacts *via* C—H⋯π con­tacts between the pyridine groups.

**Figure 5:**
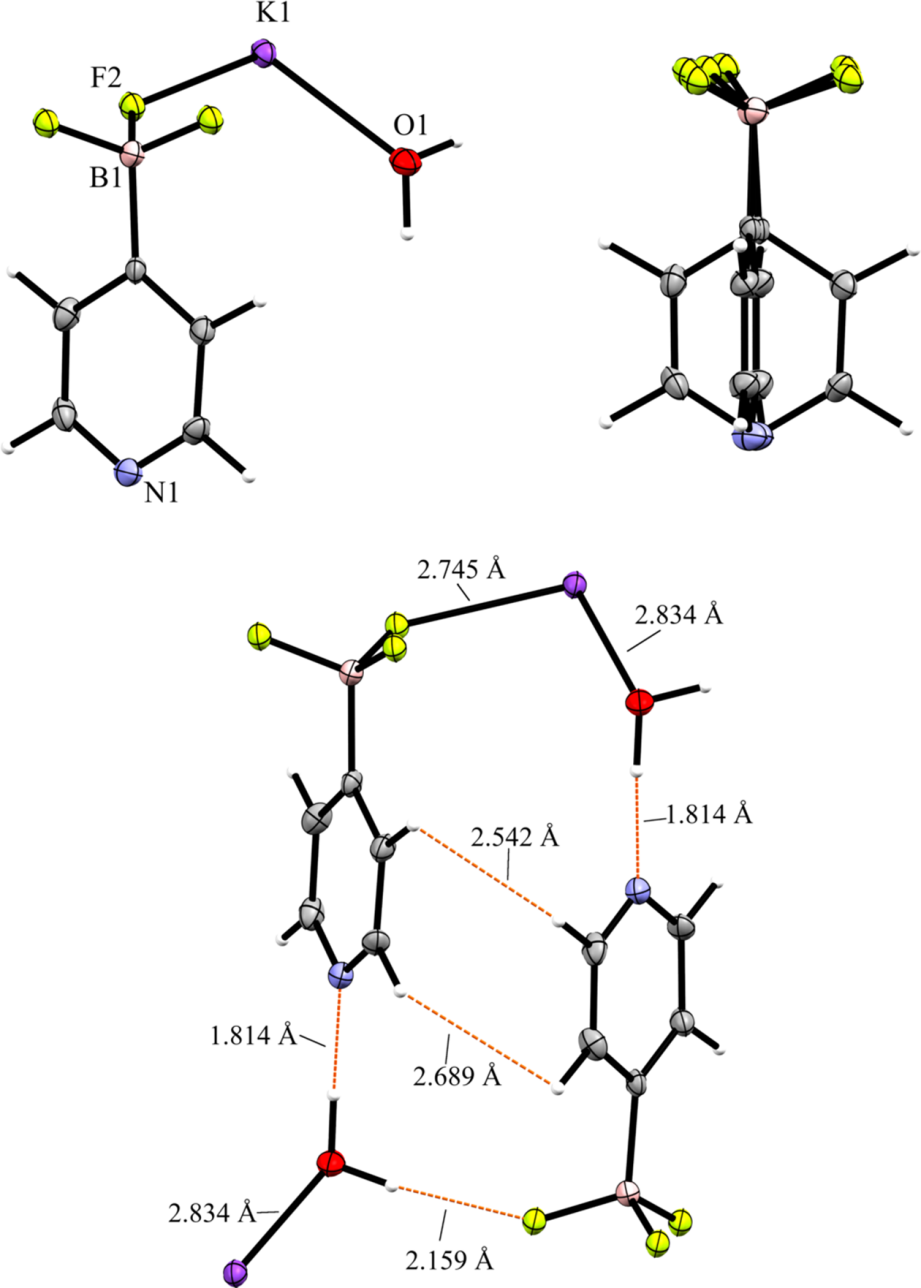
(Top left) The asymmetric unit of salt **2** showing only one of the disordered parts of the anion. (Top right) A superposition of the two equally occupied disordered parts of the anion. (Bottom) A selection of close con­tacts in **2**. O—H bond distances have been normalized to 0.993 Å.

**Figure 6:**
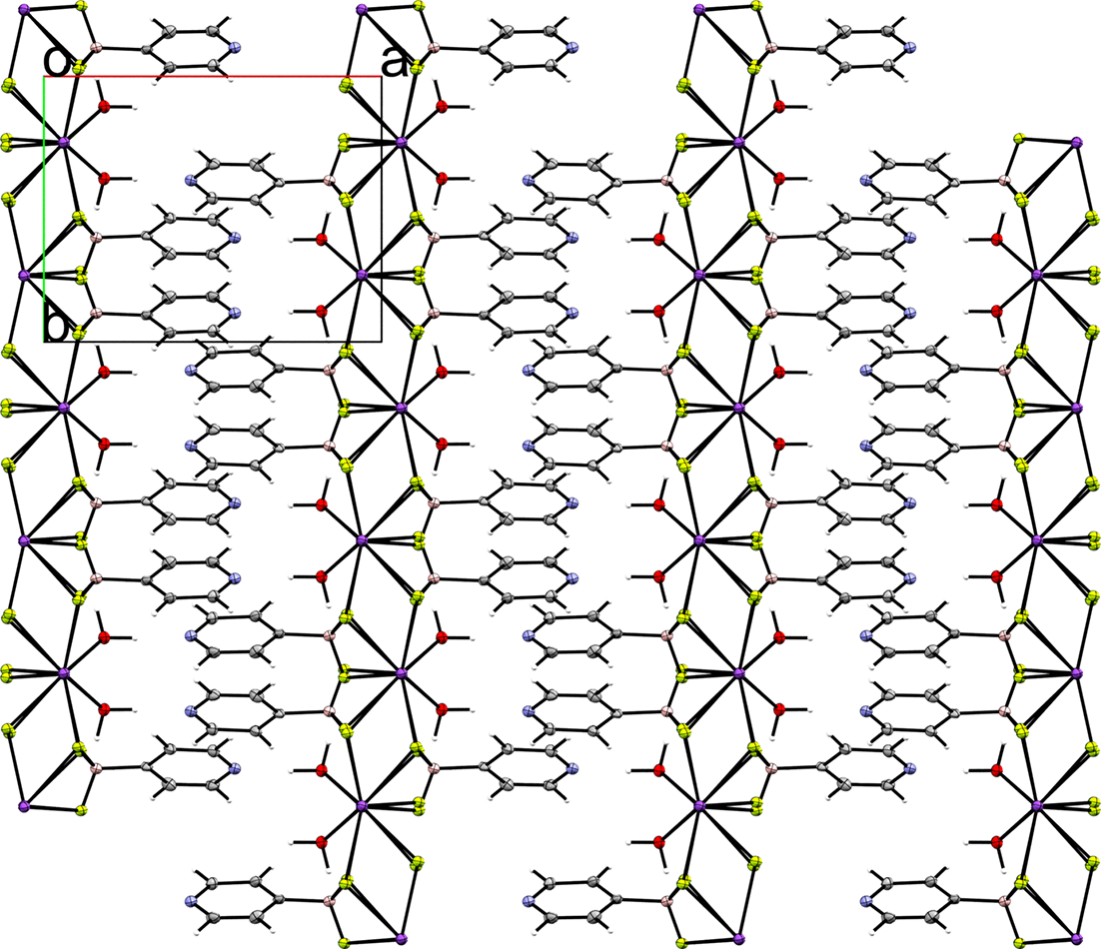
A 3×3×3 crystal packing diagram of **2**, viewed along the crystallographic *c* axis.

Attempts to obtain a neat form of salt **2** were unsuccessful; however, the monohydrate was obtained in the monoclinic space group *P*2_1_/*c* (Fig. 5 ▸). The crystal was a pseudo-merohedral twin with two twin domains having an approximate ratio of 55:45. Like salt **1**, the crystal packing of **2** consists of layers of tri­fluoro­borate moieties and potassium cations alternating with layers of pyridine moieties, which can be clearly seen when viewing a packing diagram of the crystal along the crystallographic *c* axis (Fig. 6 ▸). The pyridine group of the anion is disordered and was modeled in two positions with equal occupancy, with the second position of the pyridine ring rotated by approximately 84° from the first (Fig. 5 ▸, top right). The cocrystallized water mol­ecule plays a vital role in the packing of this crystal, linking the pyridine layers with the tri­fluoro­borate–potassium layers *via* a 1.81 (3) Å O—H⋯N hy­dro­gen bond involving the pyridine N atom, and a K⋯O con­tact with a potassium cation (Fig. 5 ▸, bottom).

The second H atom of the water mol­ecule forms a hy­dro­gen bond with an F atom from an adjacent tri­fluoro­borate moiety. There are no significant con­tacts between the anions in the structure, and the closest anion–anion con­tacts are between H atoms. The importance of the water in the crystal packing offers a potential explanation for why single crystals of the neat salt were difficult to obtain.

### Tetra­butyl­ammonium (TBA) salts

**Figure 7:**
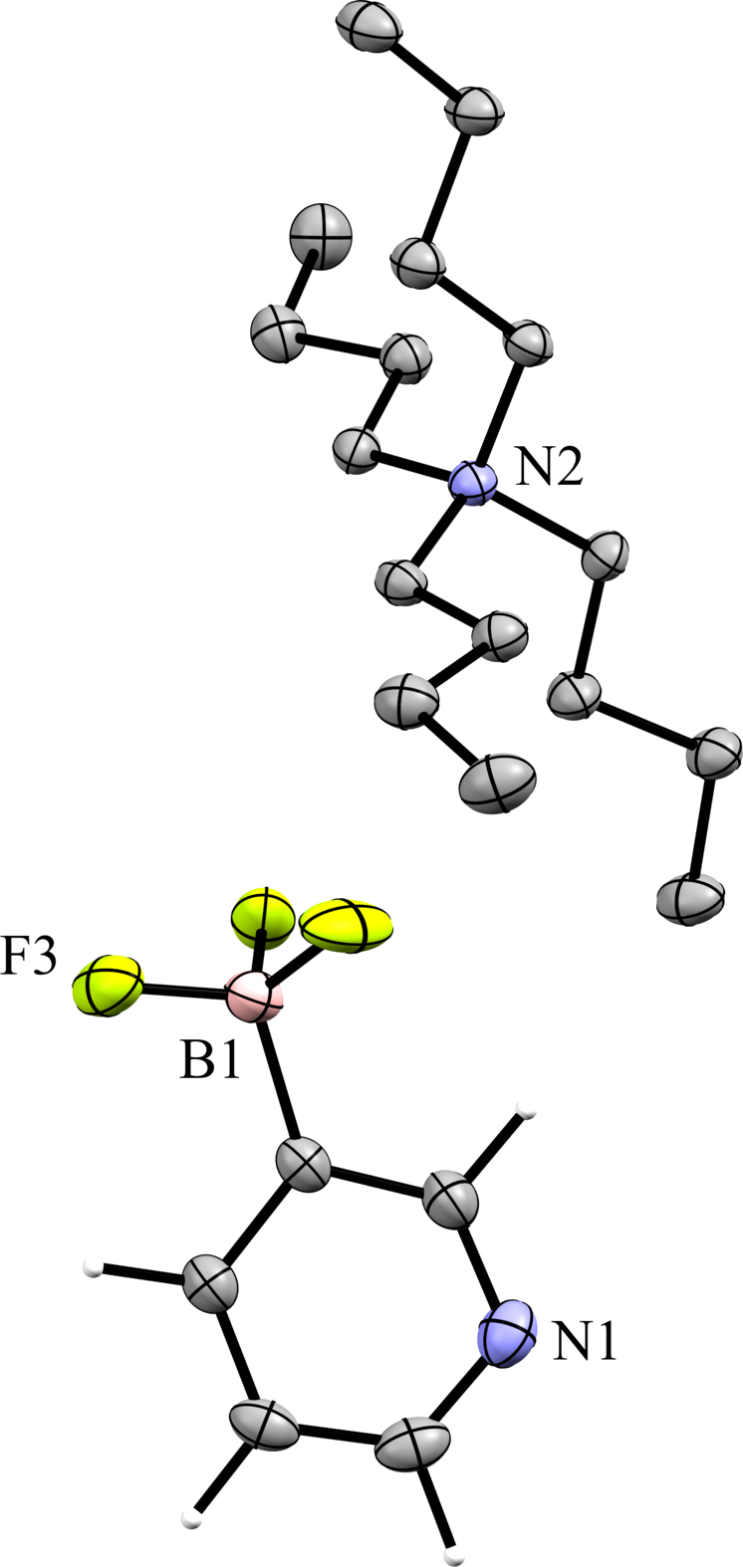
The asymmetric unit of **3**. H atoms have been omitted from the cation for clarity.

**Figure 8:**
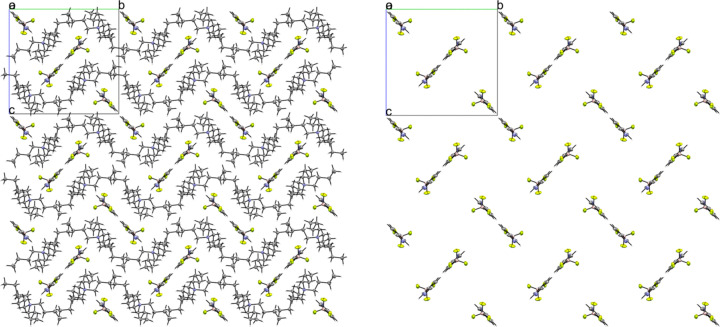
A 3×3×3 packing diagram of salt **3**, viewed along the crystallographic *a* axis. The image on the right shows the same view with the cations hidden to highlight the arrangement of the anions.

**Figure 9:**
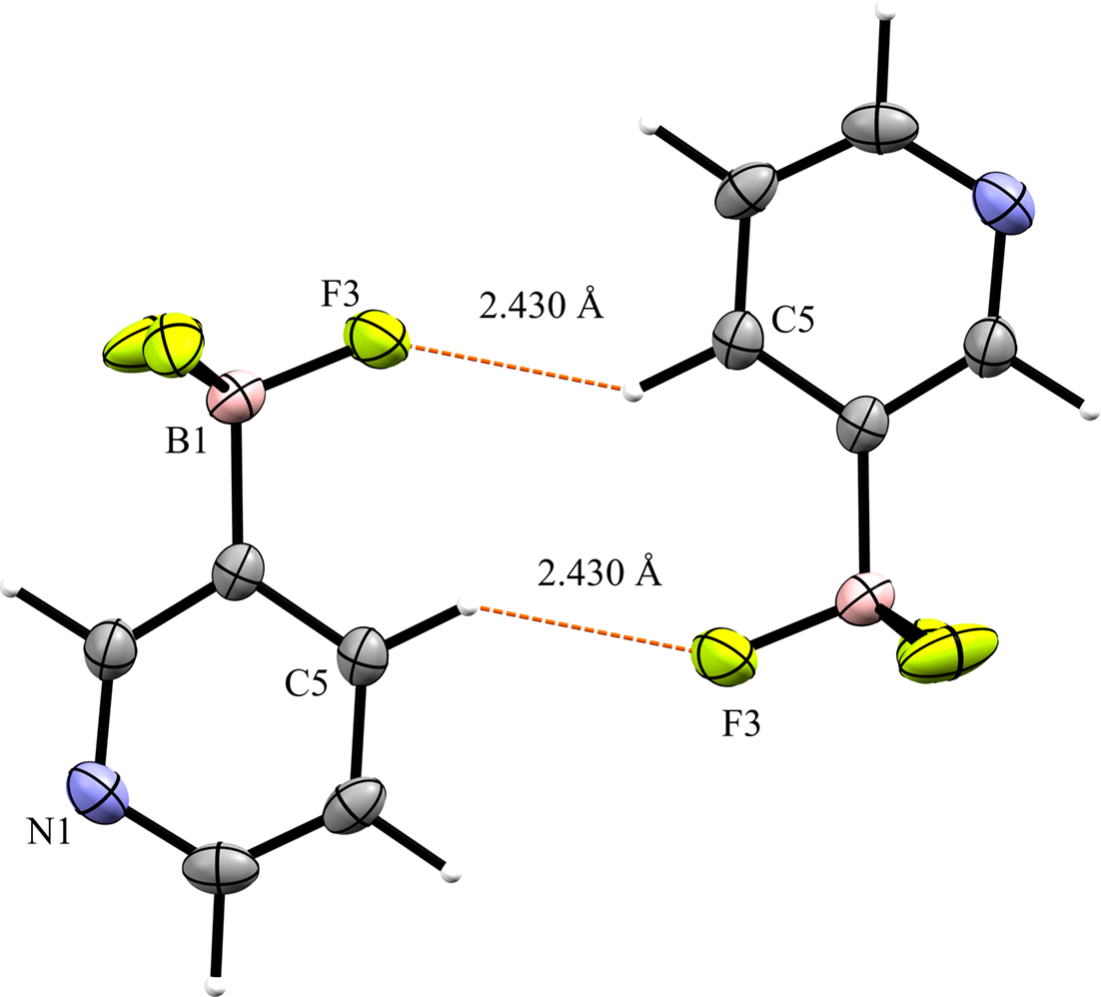
Anion–anion con­tacts in **3**.

Compounds **3** and **4** are the tetra­butyl­ammonium salts of the 3- and 4-pyri­dyl tri­fluoro­borate anions, respectively. Salt **3** crystallized in the monoclinic space group *P*2_1_/*n* with *Z*′ = 1 (Fig. 7 ▸). An inspection of the crystal packing shows that **3** packs with a ‘channel’ structure, with channels of anions running along the crystallographic *a* axis separated by channels of cations (Fig. 8 ▸). This packing motif is particularly apparent when viewing the crystal along the crystallographic *a* axis. The anions in **3** pack as dimers inter­acting across a crystallographic inversion center *via* two 2.430 (2) Å C—H⋯F con­tacts between *ortho* atoms H5 and F3 (Fig. 9 ▸). There are four con­tacts between the F atoms of the anion and the cations, with distances ranging from 2.312 (2) to 2.576 (2) Å, with an average H⋯F con­tact distance of 2.394 (2) Å. The pyridine N atom of the anion forms con­tacts with two α-H atoms on an adjacent cation, with distances of 2.609 (3) and 2.583 (2) Å.

**Figure 10:**
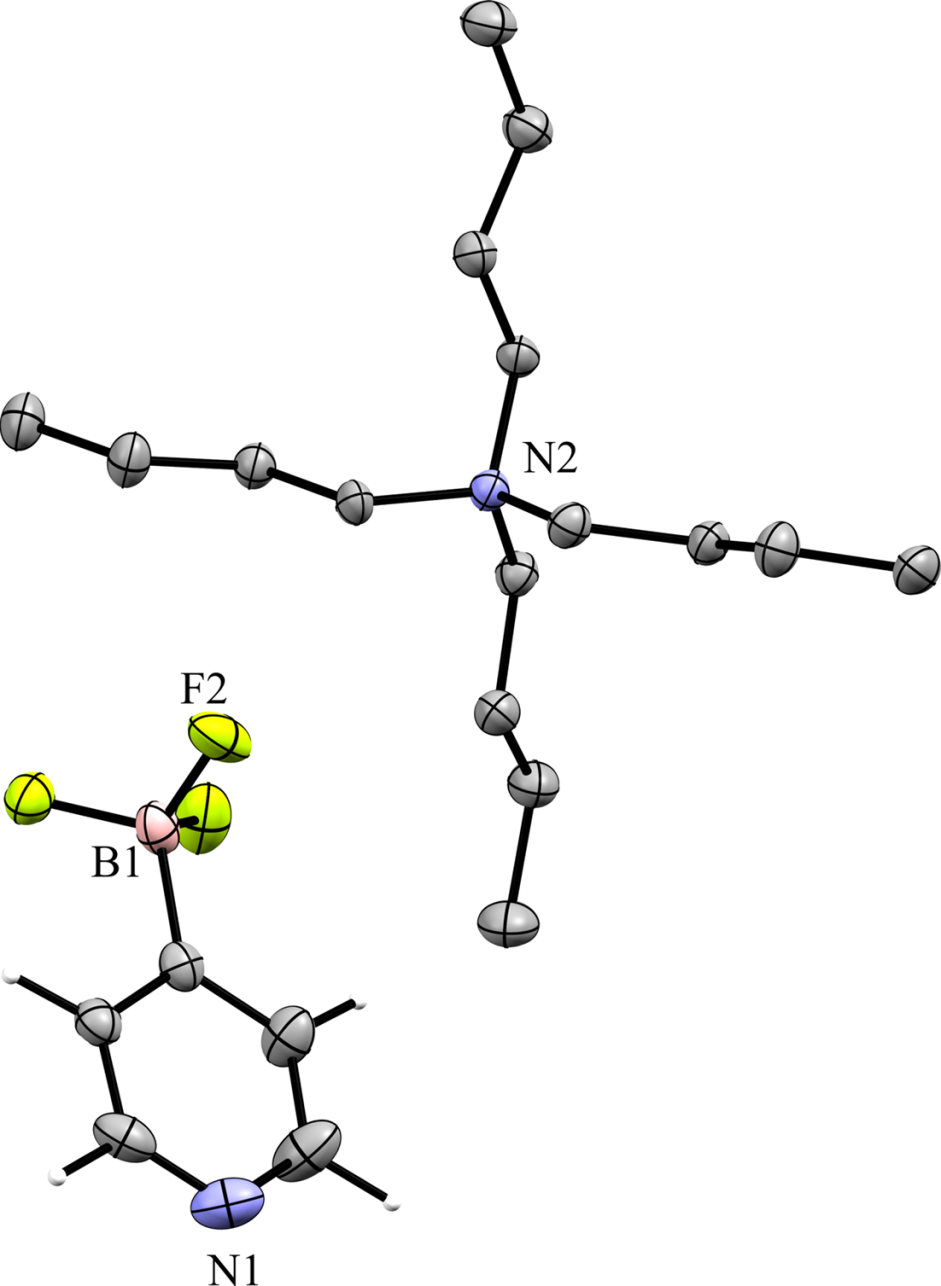
The asymmetric unit of **4**. H atoms have been omitted from the cation for clarity.

**Figure 11:**
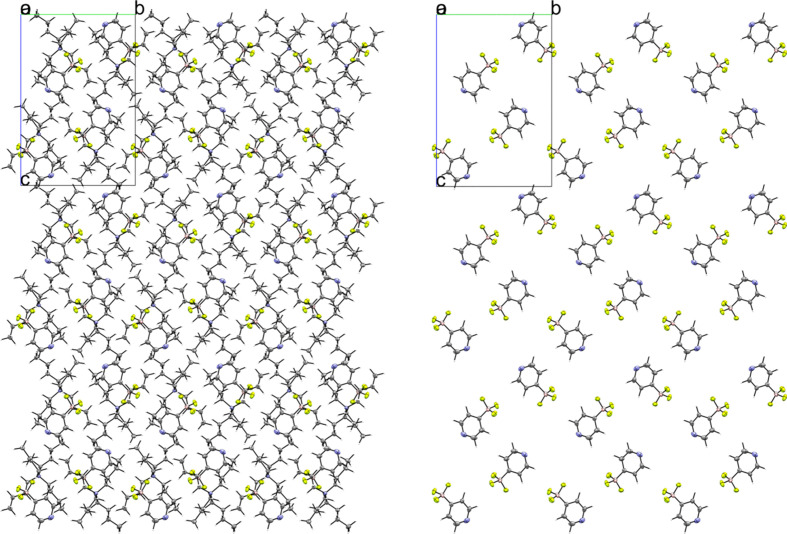
A 3×3×3 packed unit cell of **4**, viewed along the *a* axis. The image on the right is the same view with the cations hidden.

Compound **4** crystallized in the monoclinic space group *P*2_1_/*c* with *Z*′ = 1 (Fig. 10 ▸). The crystal packing of **4** shows that the anions are com­pletely separated by cations and there are no anion–anion con­tacts in the structure (Fig. 11 ▸). In com­parison to **3**, **4** has twice as many anion–cation con­tacts involving the F atoms of the anion, ranging from 2.279 (2) to 2.494 (2) Å. The average H⋯F distance in **4** is 2.391 (2) Å, similar to **3** [2.394 (2) Å]. The environment of the pyridine N atom is quite different com­pared to **3**. Unlike in **3**, which had close con­tacts between the pyridine N atom and two α-H atoms of the cation, the pyridine N atom in **4** only forms one close con­tact to a methyl H atom on the cation, with an N⋯H distance of 2.654 (2) Å.

### Tetra­phenyl­phospho­nium salts

**Figure 12:**
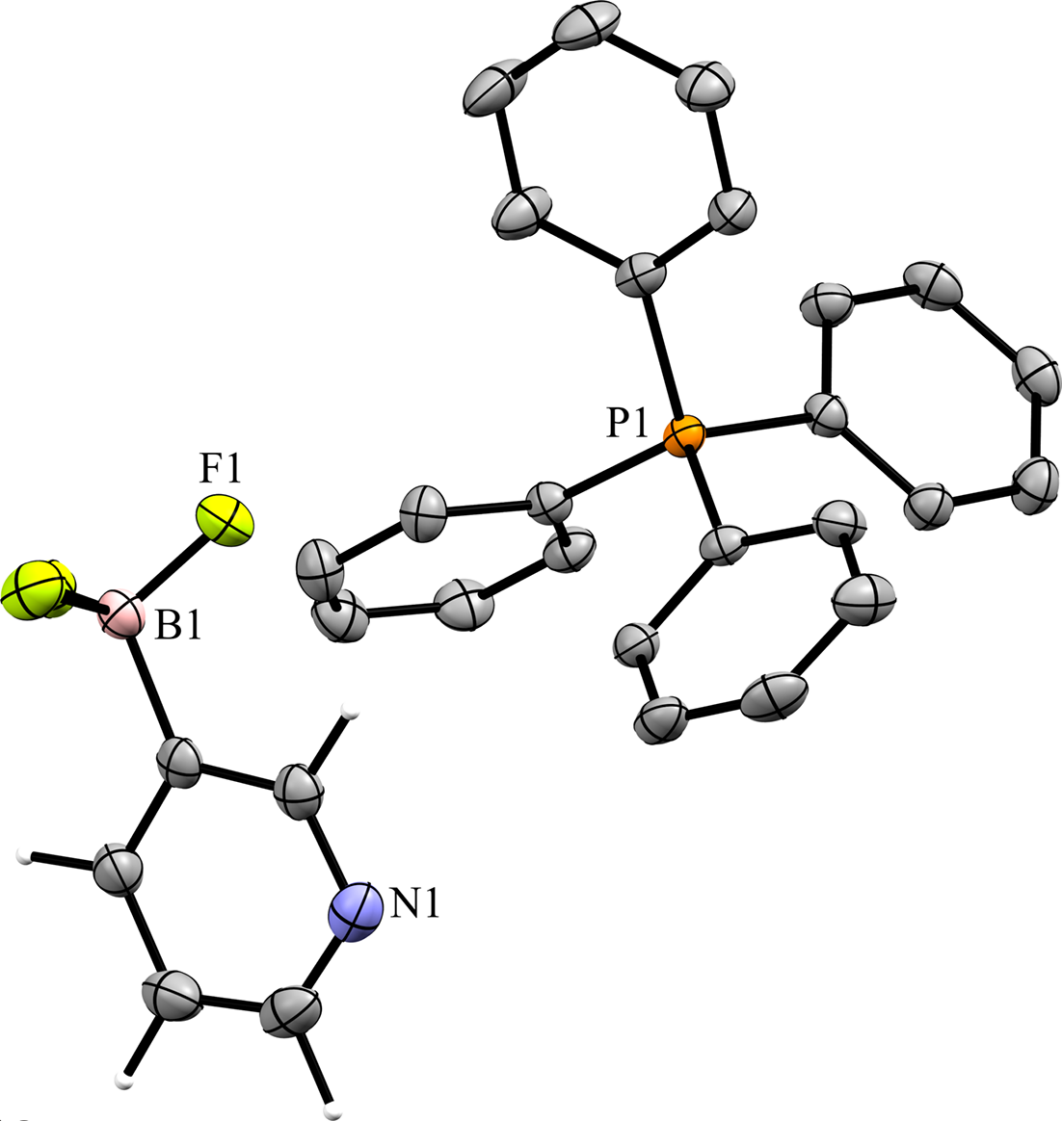
The asymmetric unit of **5**. H atoms have been omitted from the cation for clarity.

**Figure 13:**
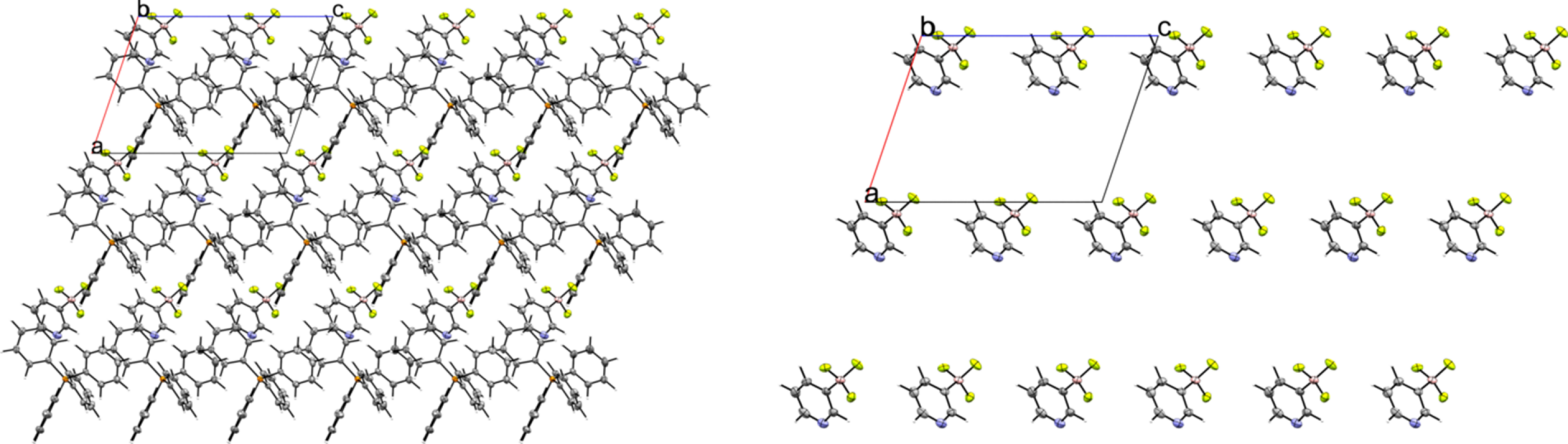
A 3×3×3 packing diagram of salt **5**, viewed along the crystallographic *b* axis. The image on the right shows the same view with the cations hidden.

Compounds **5** and **6** contain the tetra­phenyl­phospho­nium (PPh_4_^+^) cation. The 3-pyri­dyl tri­fluoro­borate salt **5** crystallized in the monoclinic space group *Pc* with *Z*′ = 1 (Fig. 12 ▸). The crystal packing of **5** shows a layer-like structure, with the ions arranged such that the cations and anions form alternating rows parallel to the *b* and *c* axes, alternating along the *a* axis of the crystal (Fig. 13 ▸). As with com­pound **4**, the anions are com­pletely separated by cations in the crystal packing and there are no close con­tacts between the anions in **5**. There are eight cation–anion con­tacts involving the F atoms of the anion ranging from 2.122 (2) to 2.480 (3) Å, with an average distance of 2.310 (3) Å, a shorter average distance than for the two TBA salts. The pyridine N atom of the anion has four close con­tacts with H atoms on different cations. Of these, the con­tacts with the *ortho*-H atom on C29 and the *meta*-H atom on C20 are in a position such that they are likely inter­acting with the lone electron pair of the pyridine N atom, while the other two con­tacts are on the face of the pyridine ring and are likely C—H⋯π con­tacts. Of the con­tacts likely involving the N-atom lone pair, the con­tact with the ortho-H atom [2.591 (3) Å] is shorter than that of the *meta*-H atom [2.699 (3) Å], consistent with com­putations that suggest the *ortho*-H atoms of PPh_4_-derived cations are the preferred site for inter­actions with anions (Dempsey & Kass, 2022 ▸).

**Figure 14:**
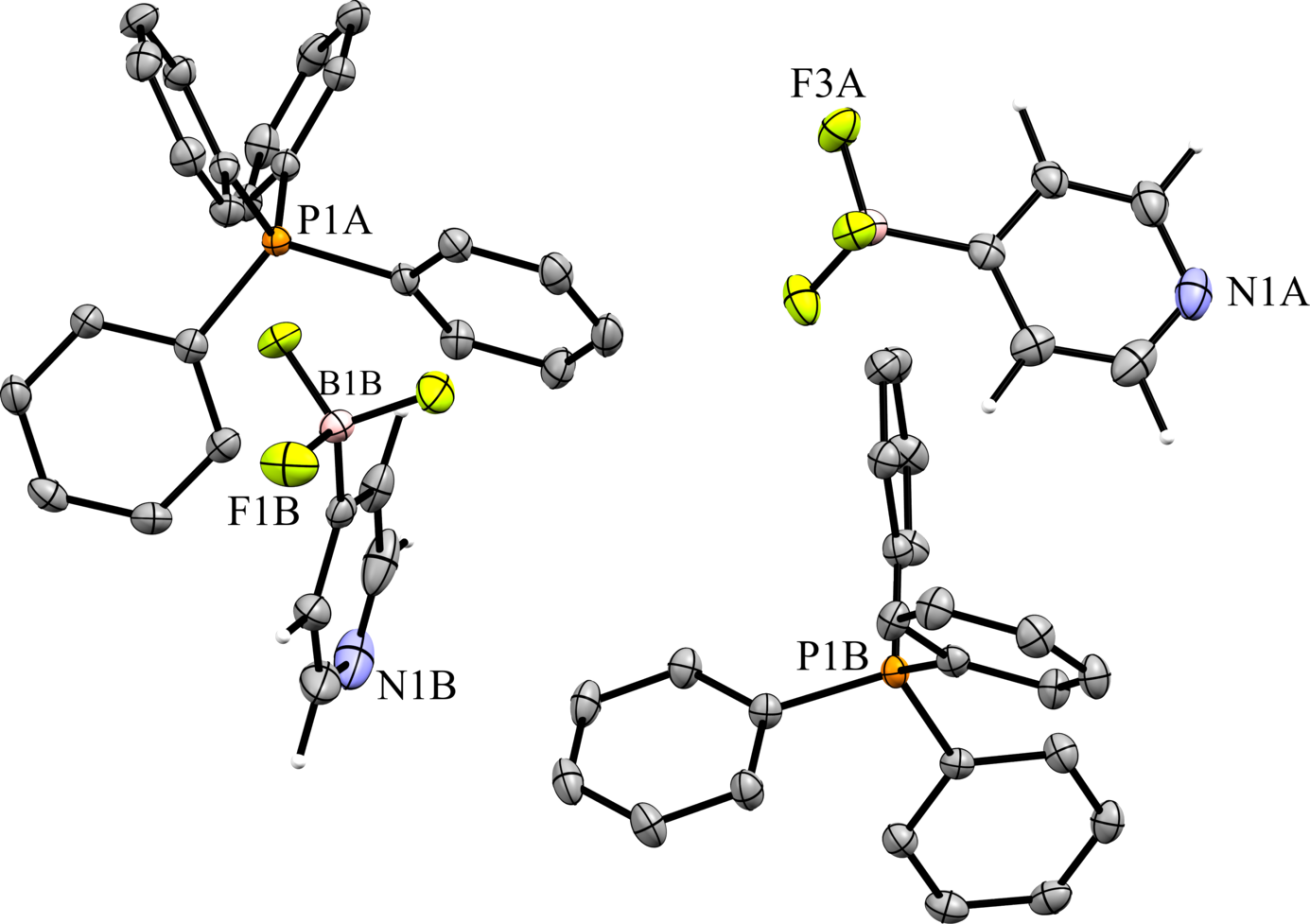
The asymmetric unit of **6**. H atoms have been omitted from the cations for clarity.

**Figure 15:**
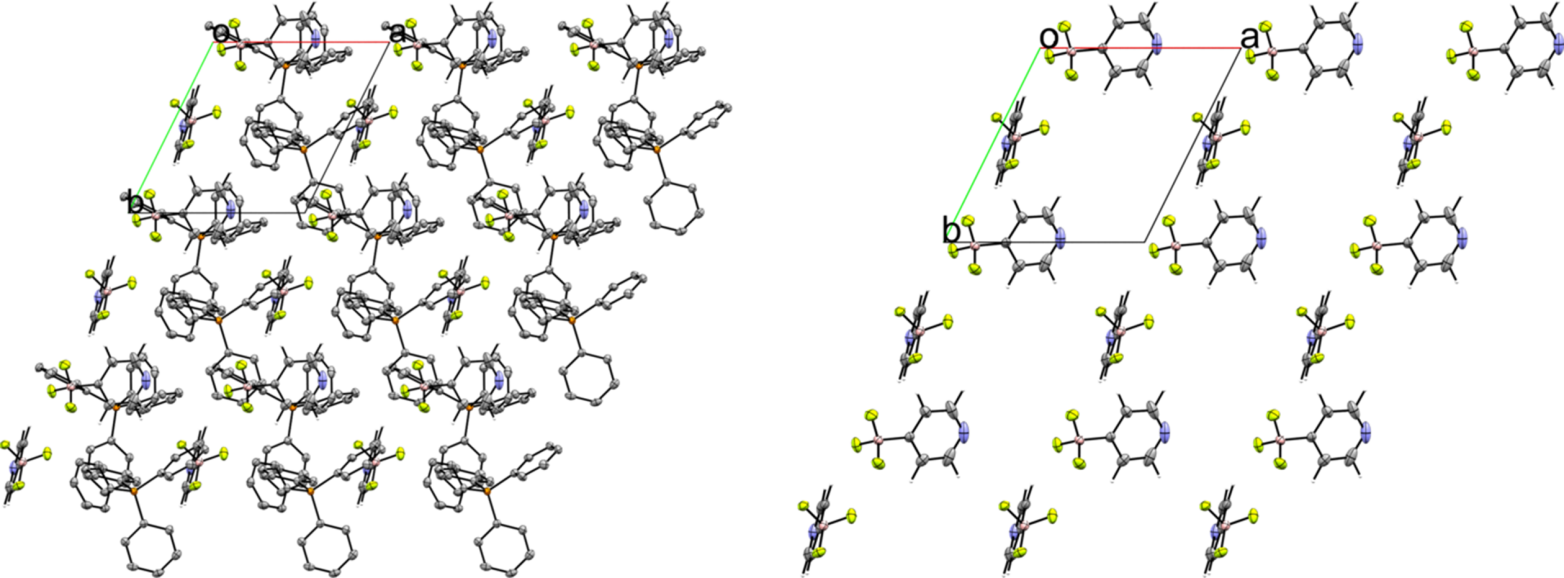
A 3×3×3 packing diagram of salt **6**, viewed along the crystallographic *c* axis. The image on the right shows the same view with the cations hidden.

**Figure 16:**
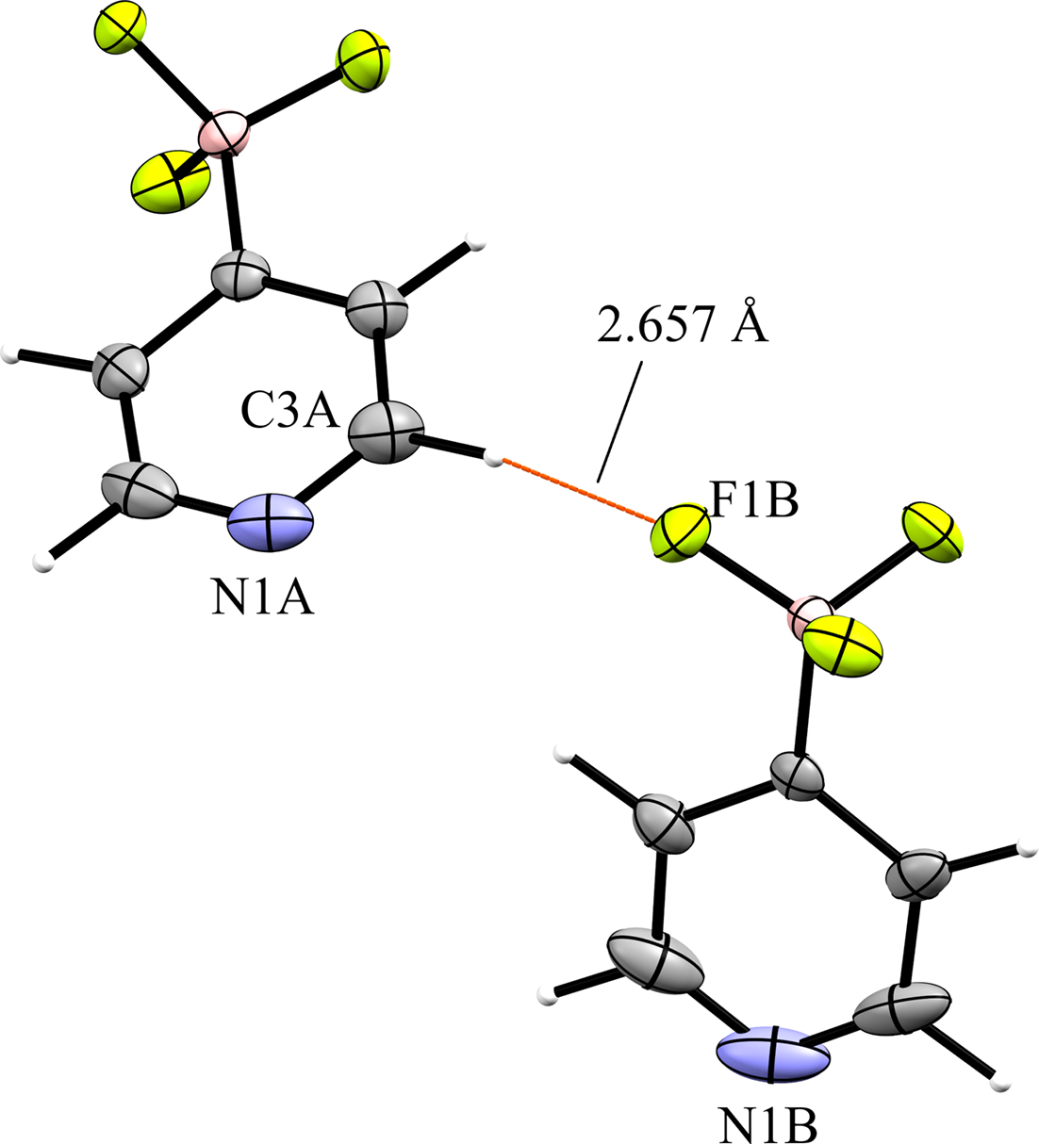
Close con­tacts between anions in **6**.

The 4-pyri­dyl analogue **6** crystallized in the triclinic space group *P*1 with *Z*′ = 2 (Fig. 14 ▸). There are two unique ion pairs in the unit cell that are distinguished by the suffixes *A* and *B*. The anions are arranged such that one anion has the pyridine N atom oriented in the direction of the positive *a* axis, while the other has the pyridine N atom oriented in the direction of the negative *c* axis, forming alternating rows of anions (Fig. 15 ▸). There is a single anion–anion con­tact in the packing of **6**, with the anions linked by a single 2.657 (6) Å C—H⋯F con­tact between a *meta*-H atom on anion *A* with an F atom on anion *B* (Fig. 16 ▸). Anion *A* has ten cation–anion con­tacts involving the F atoms of the anion ranging from 2.216 (5) to 2.626 (4) Å, with an average con­tact distance of 2.427 (4) Å. Anion *B* has eight analogous con­tacts ranging from 2.296 (3) to 2.620 (3) Å, with a slightly longer average con­tact distance of 2.459 (4) Å. The pyridine N atoms of the two anions in the structure differ slightly in their crystallographic environments. Anion *A* has three close con­tacts between the pyridine N atom and three different cations. Only one of these, the 2.566 (5) Å con­tact with the *meta*-H atom on C26*A*, is positioned in such a way that the H atom is able to inter­act with the lone pair of the pyridine N atom. The other two C—H⋯N inter­actions occur on the ‘face’ of the N atom and are likely electrostatic in nature rather than a hy­dro­gen bond, but it should be noted that the con­tact with the *para*-H atom on C9*A* is the shortest of the three con­tacts at 2.494 (5) Å. Anion *B* only forms two con­tacts with the pyridine N atom. In this case, both con­tacts are positioned in such a way that the H atom could be inter­acting with the lone pair of the pyridine N atom. The shortest con­tact is with the *ortho*-H atom of C13*A* at a distance of 2.463 (7) Å, while the second is much longer at 2.780 (6) Å with the *para*-H atom of C15*B*.

### Tetra­kis(3,5-di­meth­oxy­phen­yl)phospho­nium salts

**Figure 17:**
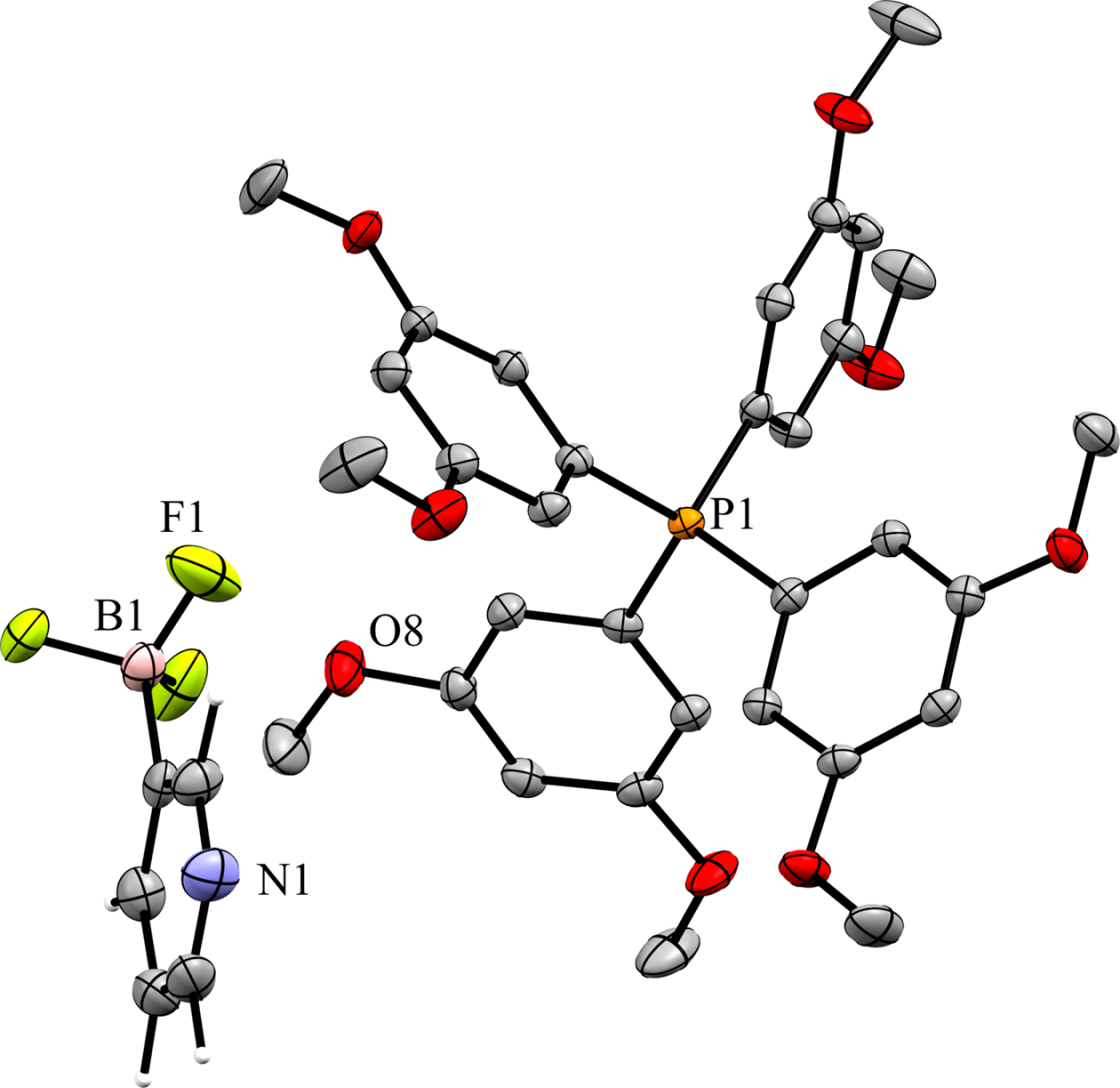
The asymmetric unit of **7**. H atoms have been omitted from the cation for clarity.

**Figure 18:**
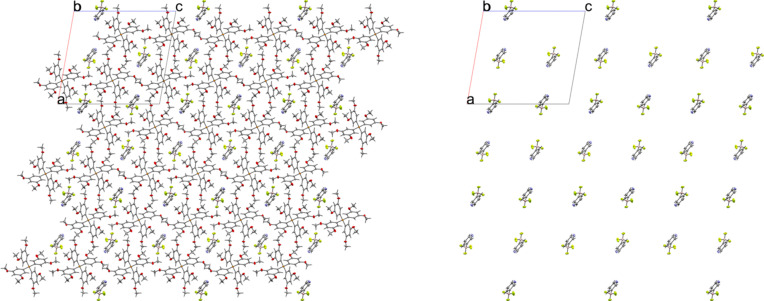
The left image shows a 3×3×3 packing diagram of salt **7**, viewed along the crystallographic *b* axis. The image on the right shows the same view with the cations hidden.

**Figure 19:**
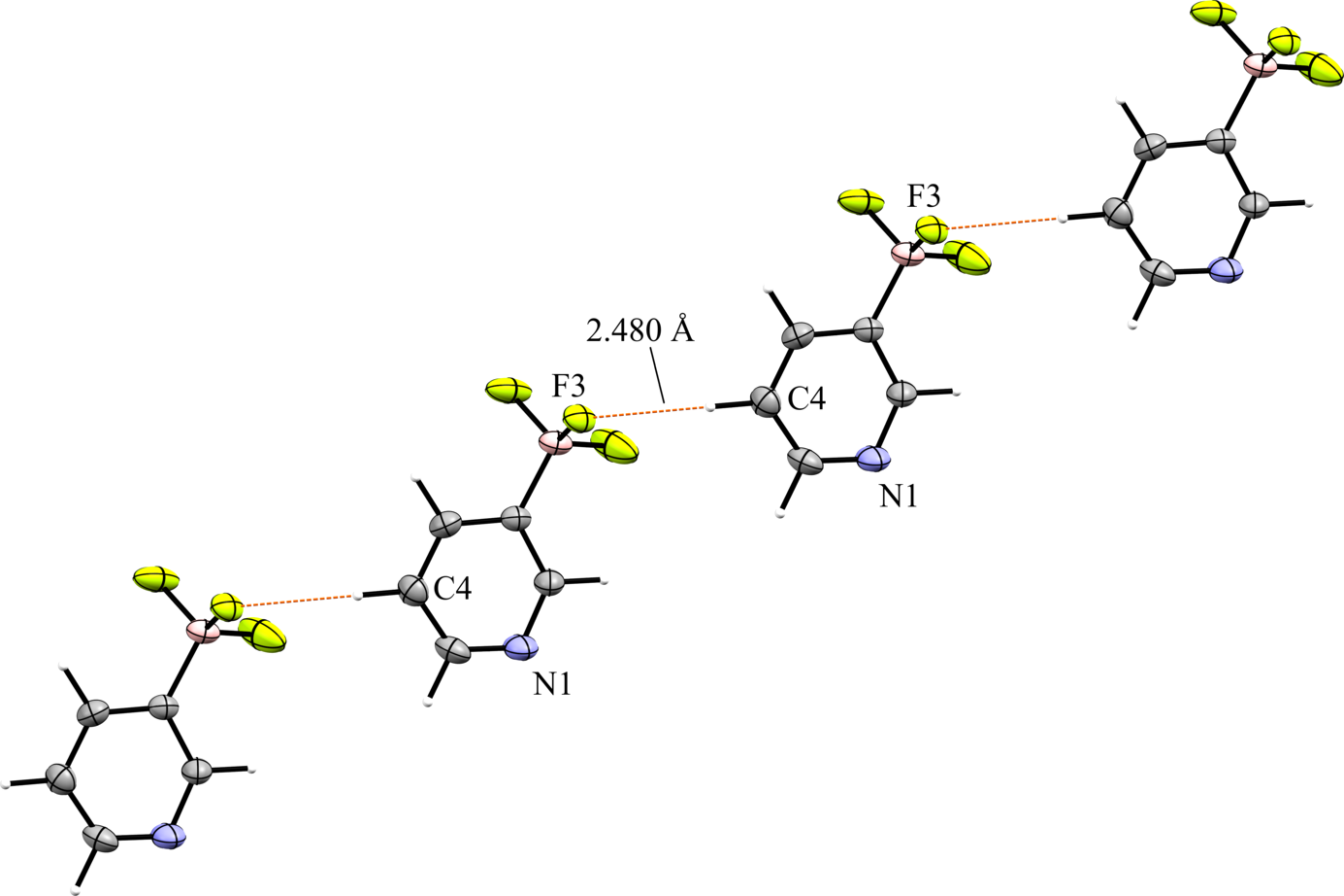
Anion–anion con­tacts present in salt **7**.

Compounds **7** and **8** contain the tetra­kis­(3,5-di­meth­oxy­phen­yl)phospho­nium cation (MeO-Phos). The 3-pyri­dyl bo­rate salt **7** crystallized in the space group *P*2_1_/*n* with *Z*′ = 1 (Fig. 17 ▸). Similar to salt **3**, the crystal packing of **7** shows a channel motif, where anions form channels that are separated by cations (Fig. 18 ▸). These channels run roughly parallel to the *b* axis and layers of cations and anions alternate along the crystallographic *a* and *c* axes. Within each channel the anions are linked *via* a short con­tact of 2.480 (3) Å between the *meta*-H atom of the anion and an F atom of an adjacent anion. The result of this is a one-dimensional polymeric chain of anions (Fig. 19 ▸). This polymeric structure of the anion channels is quite different from the structure of the anion channels in **3**, where the channels were formed by repeating units of anion dimers. There is a total of seven cation–anion con­tacts involving the F atoms of the anions. These con­tacts range in length from 2.287 (2) to 2.627 (3) Å, with an average length of 2.429 (3) Å, a similar average length to anion *A* in **6** [2.427 (4) Å]. The pyridine N atom of the anion in **7** forms three close con­tacts with two different cations. Like in anion *A* of salt **6**, two of the con­tacts formed in **7** occur towards the faces of the pyridine ring. Both of these con­tacts are with methyl H atoms of the cation, and these con­tacts are both long, with lengths of 2.836 (3) and 2.778 (3) Å. The third con­tact is between N1 and the H atom on *para* atom C26 of the cation. This H atom is directly in line with the N-atom lone pair and forms a short C—H⋯N con­tact of 2.250 (3) Å.

**Figure 20:**
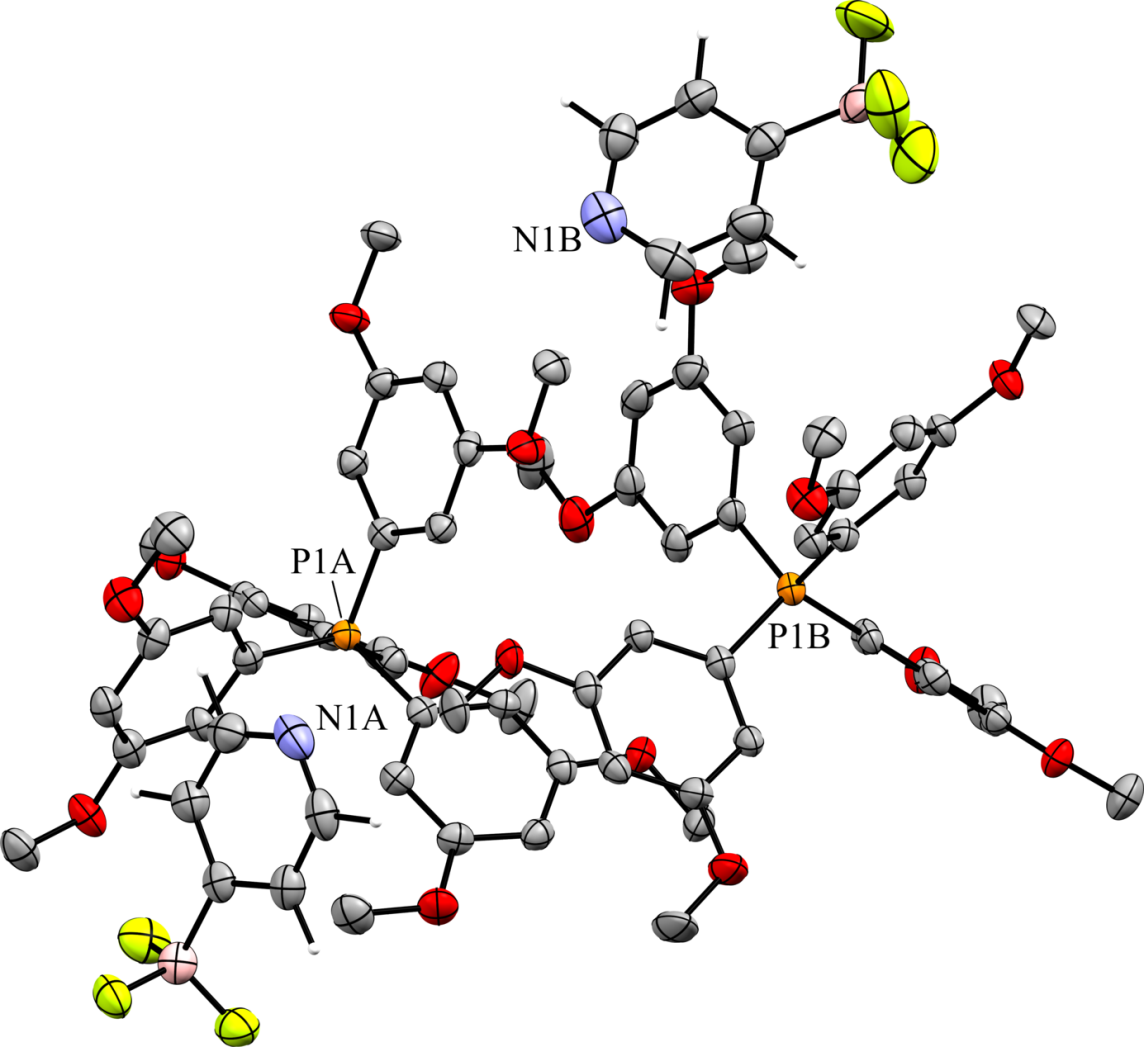
The asymmetric unit of **8**. Only the majorly occupied part of anion *B* is shown. H atoms have been omitted from the cations for clarity.

**Figure 21:**
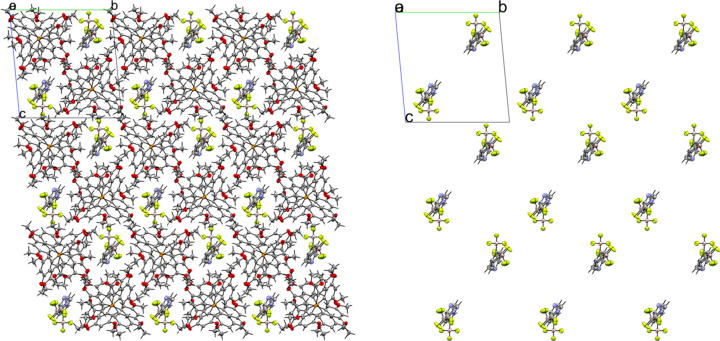
The image on the left shows a 3×3×3 packing diagram of salt **8**, viewed along the crystallographic *a* axis. Only the majorly occupied part of anion *B* is shown. The image on the right shows the same view with the cations hidden.

**Figure 22:**
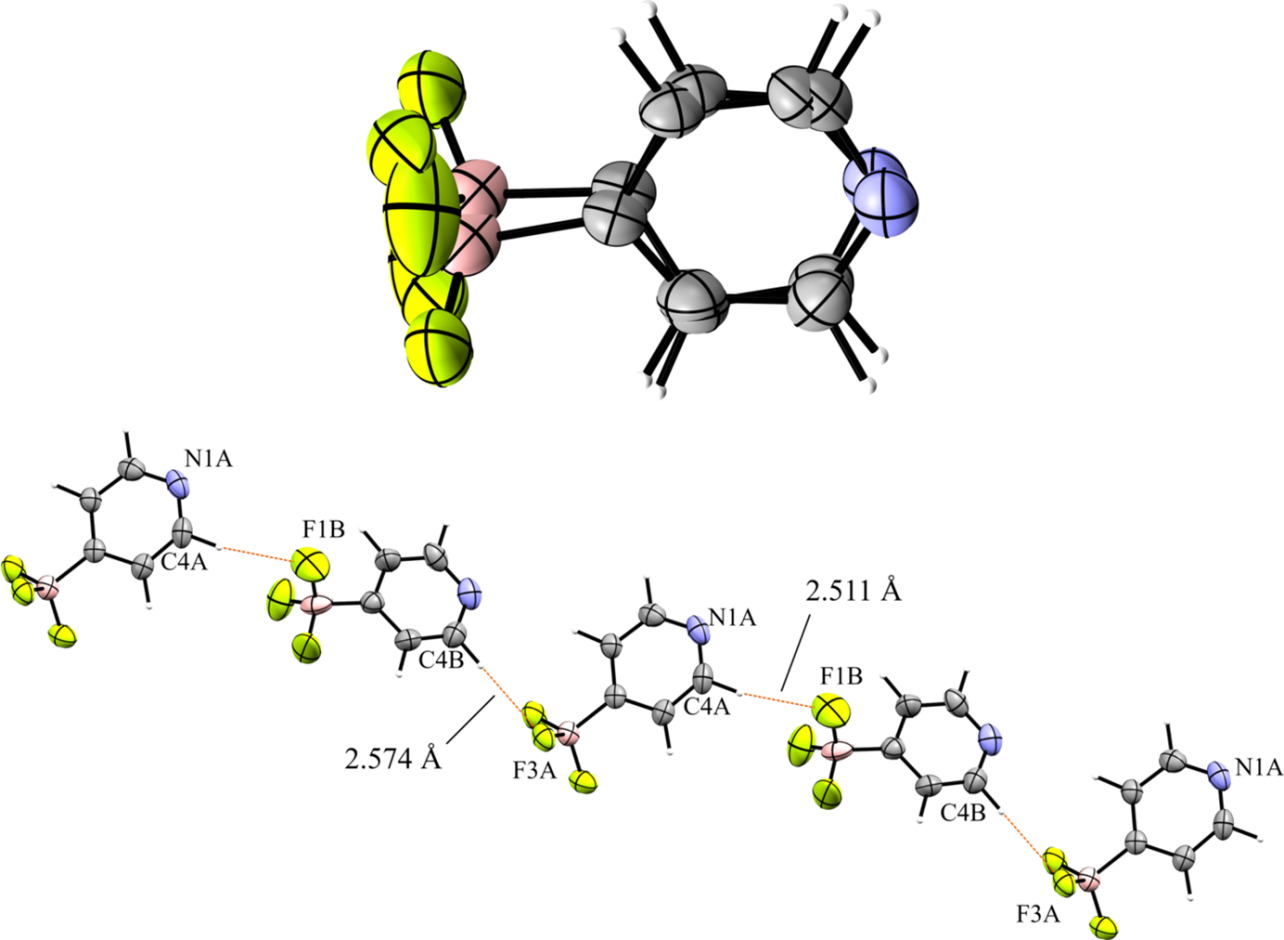
(Top) A superposition of the two parts of the disordered anion in **8**. (Bottom) The head-to-tail chain packing of the anions in **8**. Only the majorly occupied part of anion *B* is shown.

The 4-pyri­dyl borate analogue **8** crystallized in the triclinic space group *P*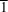 with *Z*′ = 2 as a non-merohedral twin with two twin domains of equal volume (Fig. 20 ▸). The packing in **8** shows a channel motif, with the anions forming one-dimensional ‘head-to-tail’ chains in the direction of the *a* axis, with adjacent layers of chains related by inversion symmetry (Fig. 21 ▸). The anions in the chains are linked by con­tacts between a *meta*-H atom of the pyridine ring with an F atom in the next anion. Like in **6**, there are two unique cations and anions in the asymmetric unit of the crystal, differentiated by the suffixes *A* and *B*. The two unique anions alternate in the anion channel, exhibiting a polymeric structure like that seen in **7** (Fig. 22 ▸, bottom). Anion *B* is disordered and modeled in two parts, with an occupancy ratio of approximately 66:34 (Fig. 22 ▸, top). The primary difference between the two disordered anions is a rotation of the BF_3_ group. Anion *A* has eight cation–anion con­tacts involving the F atoms of the anion (Fig. 22 ▸, top). The con­tacts range from 2.260 (5) to 2.644 (5) Å, with an average length of 2.440 (5) Å. The two disordered parts of anion *B* differ in the number of con­tacts involving the F atoms of the anion. The majorly occupied portion of anion *B* also has eight con­tacts between the cations and the F atoms of the anion ranging from 2.210 (10) to 2.612 (7) Å, with an average length of 2.400 (8) Å, slightly shorter than the average distance for anion *A*. In the minorly occupied part of anion *B*, the BF_3_ group is rotated in such a way that atom F1*B*′ has no con­tacts with the cation, and overall, the number of cation con­tacts involving the F atoms of the anion has reduced to five, ranging from 2.299 (10) to 2.610 (10) Å, with an average distance of 2.486 (14) Å. Averaging the cation–fluorine con­tact distances for all three anions in **8** gives a distance of 2.442 (9) Å, slightly longer than the average distance in **7** [2.429 (3) Å]. Anion *A* shows three C—H⋯N con­tacts between the pyridine N atom and methyl groups on two cations. As seen in several of the other 4-pyri­dyl anions, two of the con­tacts [with methyl H atoms on C21*B* and C28*A* of 2.633 (5) and 2.797 (5) Å, respectively] are on the face of the pyridine ring, while the third con­tact, with a methyl H atom on C13*B*, is in a location such that it could be inter­acting with the pyridine lone pair. However, this con­tact is likely not a C—H⋯N hy­dro­gen bond, as the con­tact is quite long [2.855 (5) Å] and the C—H⋯N angle is 100°, both factors suggesting this is a primarily electrostatic con­tact rather than a hy­dro­gen-bonding inter­action. The majorly and minorly occupied parts of disordered anion *B* have slightly different environments of the pyridine N atom. The majorly occupied part of anion *B* shows only two close con­tacts involving the pyridine N atom, one with a H atom on methyl atom C29*A* [2.74 (2) Å] and the other with the H atom on *para* atom C9*A* [2.661 (10) Å]. The con­tact with the *para*-H atom is in line with the lone pair of the pyridine N atom, while the con­tact with the methyl H atom occurs more on the face of the pyridine ring. The minorly occupied part of anion *B* has con­tacts with these same H atoms, although the pyridine N atom has shifted further from C29*A* and closer to C9*A*, resulting in new con­tact distances of 2.81 (4) Å for the methyl H atom on C29*A* and 2.411 (10) Å for the *para*-H atom on C9*A*. Additionally, the minorly occupied portion of anion *B* has con­tacts with two of the methyl H atoms of C13*A* [distances of 2.69 (4) and 2.67 (4) Å] with the pyridine N atom. The new C—H⋯N con­tacts occur on the face of the pyridine ring.

### Tetra­kis[4-(di­methyl­amino)­phen­yl]phospho­nium salts

**Figure 23:**
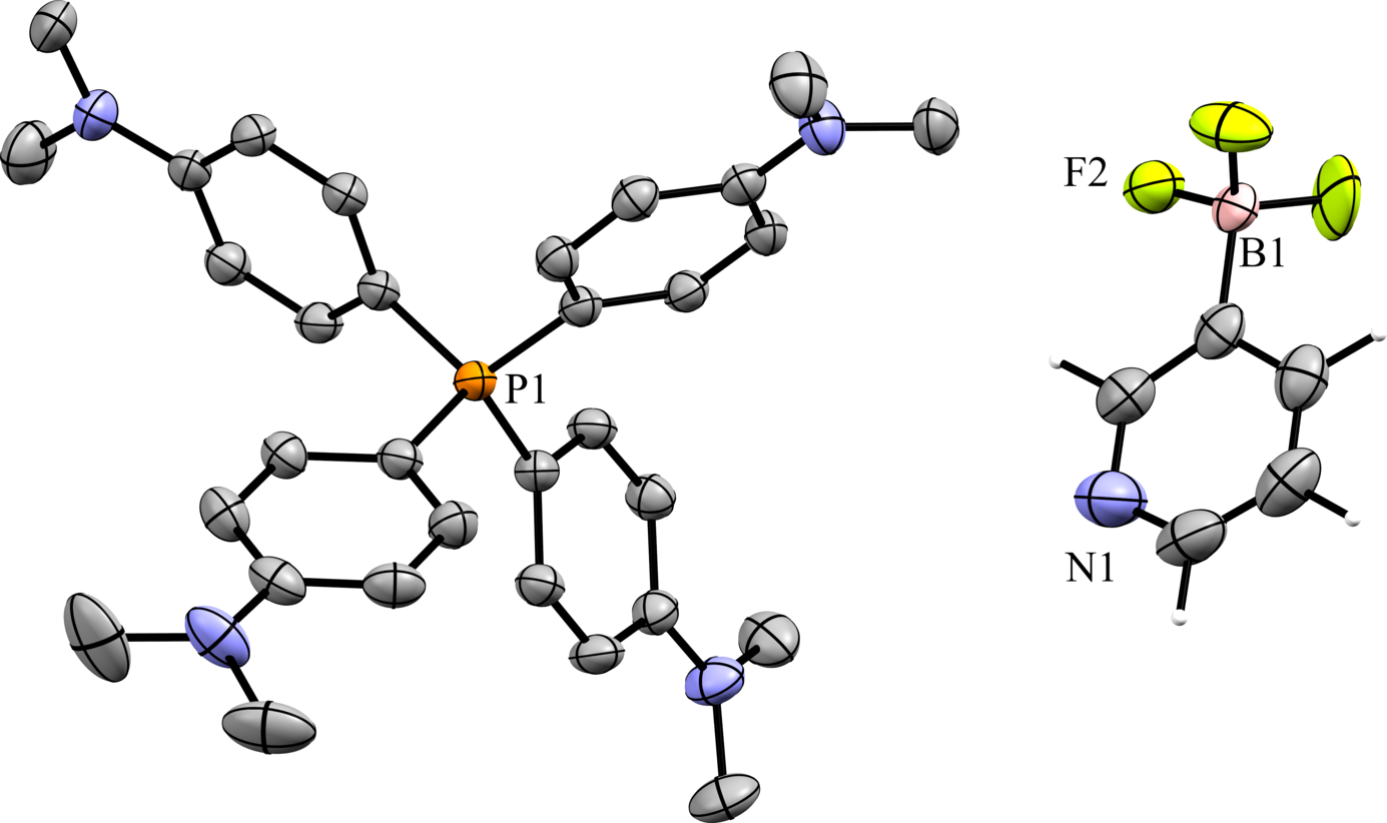
The asymmetric unit of **9**. H atoms have been omitted from the cation for clarity.

**Figure 24:**
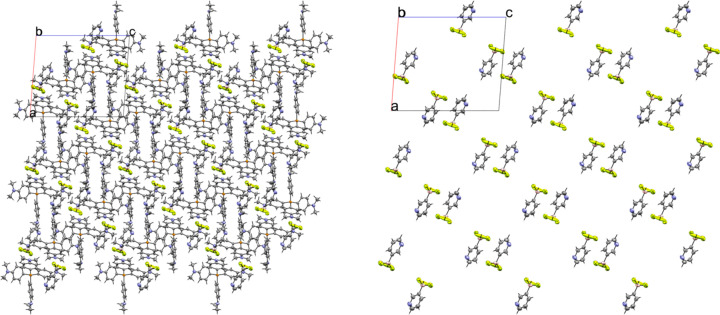
A 3×3×3 packing diagram of salt **9**, viewed along the crystallographic *b* axis. The image on the right shows the same view with the cations hidden. Only the unprimed anion is shown.

**Figure 25:**
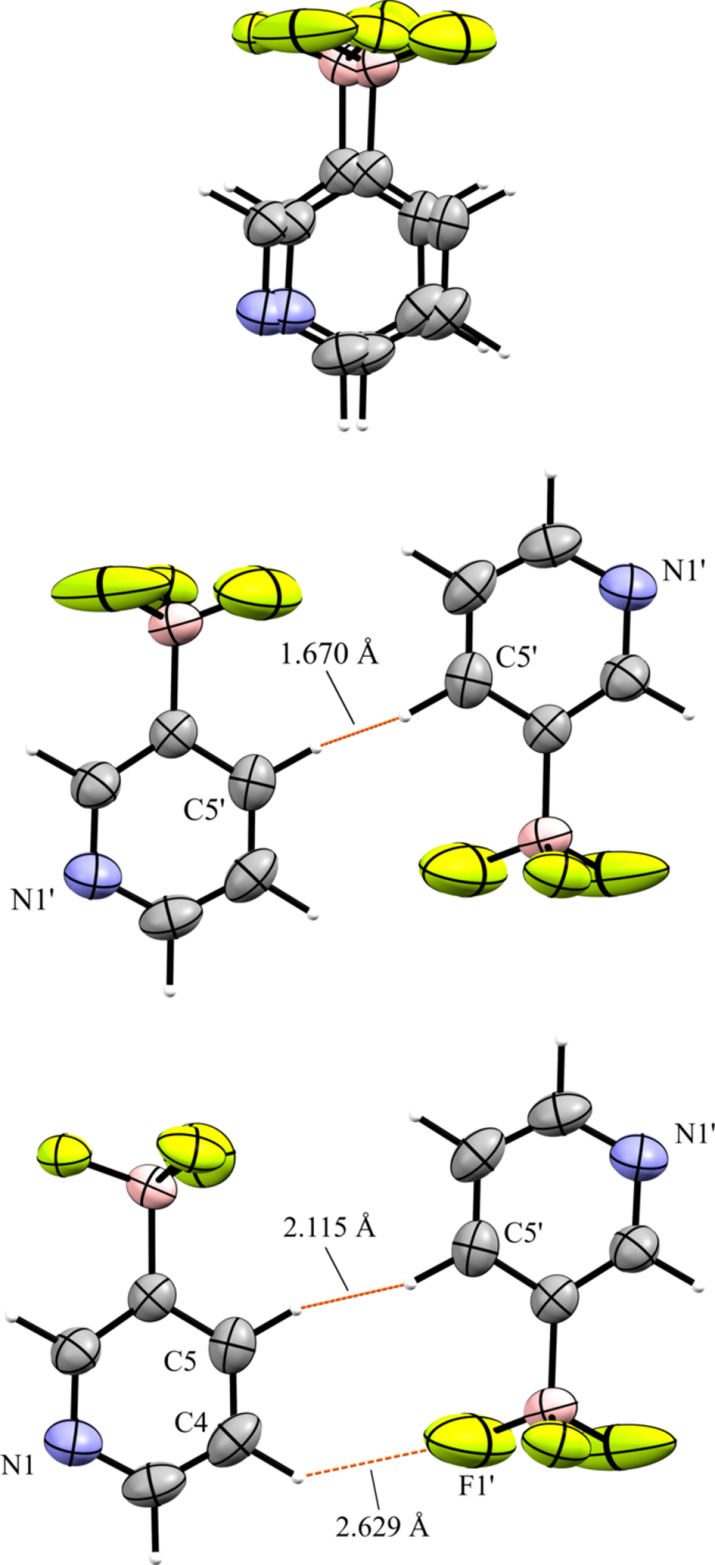
(Top) A superposition of the two equally occupied disordered parts of the anion in **9**. (Middle) The close con­tact between the primed anion and its inversion-related equivalent highlighting the unlikely 1.67 (3) Å H⋯H con­tact. (Bottom) A more realistic anion dimer between the primed and unprimed anions across the inversion center.

Compounds **9** and **10** contain the tetra­kis­[4-(di­methyl­amino)­phen­yl]phospho­nium cation (NMe_2_-Phos). Compound **9** crystallized in the monoclinic space group *P*2_1_/*n* with *Z*′ = 1 (Fig. 23 ▸). Like com­pound **3**, the anions in **9** crystallize as dimers across a crystallographic inversion center. The anion dimers are com­pletely separated from other anion dimers by cations (Fig. 24 ▸). A close con­tact is made between the *meta*-H atom of one anion with an F atom of the inversion-related anion. The anion is disordered and modeled in two parts, each with 50% occupancy (Fig. 25 ▸, top). In the crystal structure, the disordered parts are differentiated by the addition of a prime (′) suffix to the second part. Because the anions have equal occupancy, the two anions will be referred to as the ‘primed’ and ‘unprimed’ anions. The anion dimers are related by an inversion center, however it is unlikely that the anions that are related directly by symmetry pack at the same time (*i.e.* the primed anion forming a dimer with the primed anion), as this results in a very close 1.67 (3) Å H⋯H con­tact between H5′ and it’s inversion-related equivalent (Fig. 25 ▸, middle). Instead, the packing most likely consists of the unprimed anion and the primed anion packed across the inversion center (Fig. 25 ▸, bottom). In this packing arrangement, the con­tact distance between H5 and H5′ is a more reasonable 2.12 (3) Å. The anions form a C—H⋯F con­tact of 2.63 (5) Å between the *meta*-H atom on C4 of the unprimed anion and F1′ of the primed anion. The major difference between the primed and unprimed anions is a rotation of the BF_3_ groups on the anions. The F atoms of the unprimed anion form six cation–anion con­tacts ranging from 2.165 (7) to 2.549 (5) Å, with an average length of 2.361 (5) Å. The F atoms of the primed anion also form six cation–anion con­tacts, ranging from 2.169 (9) to 2.565 (5) Å, with an average of 2.444 (13) Å. The average con­tact distance for both anions is 2.402 (9) Å. The two anions differ only slightly in the chemical environments of their pyridine N atoms. Both anions form C—H⋯N con­tacts with a methyl H atom on C21. The con­tact with the unprimed anion is shorter, with a distance of 2.521 (10) Å, while the con­tact with the primed anion is 2.81 (2) Å.

**Figure 26:**
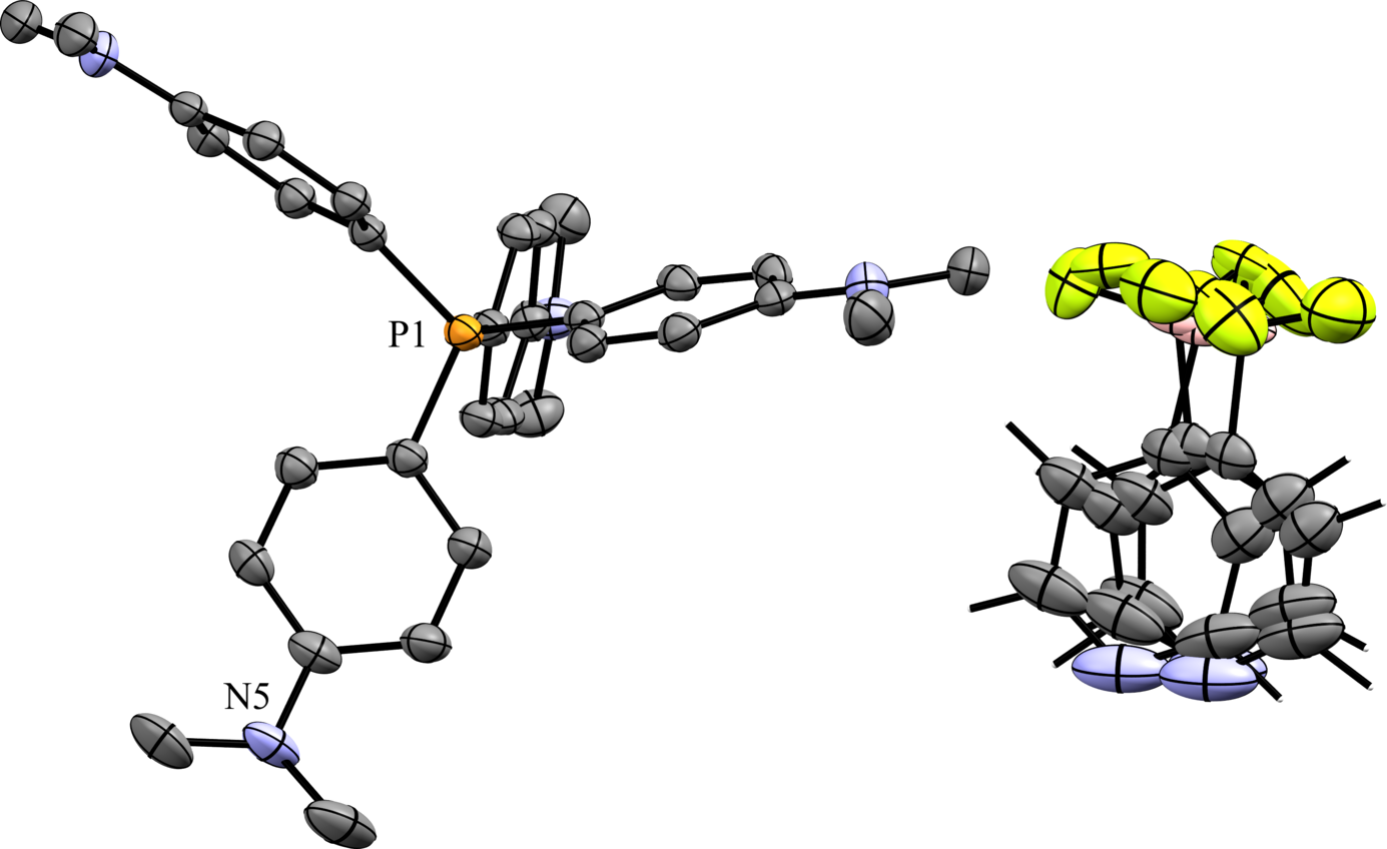
The asymmetric unit of **10**. All parts of the disordered anion are shown superimposed. H atoms have been omitted from the cation for clarity.

**Figure 27:**
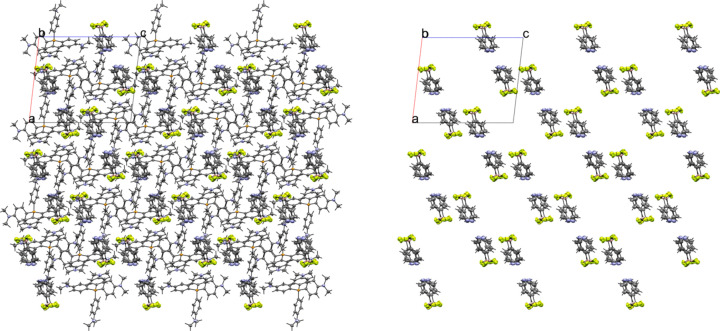
A 3×3×3 packed unit cell of **10**. The image on the right has the cations hidden to highlight the dimer packing arrangement of the anions.

Two different crystals of com­pound **10** were obtained by evaporation from two different solvents. When DCM was used as a solvent, crystals of the neat salt were obtained in the monoclinic space group *P*2_1_/*n* with *Z*′ = 1 (Fig. 26 ▸). The cation in the crystal structure is well resolved; however, the anion is severely disordered. The anion is modeled in three parts with approximate occupancies of 43.3, 33.2, and 23.5%. Because of the highly disordered nature of the anion, there is more uncertainty in the con­tact distances between the mol­ecules in the crystal structure. The packing is very similar to what is seen in **9**, with the anions packed in dimers related by inversion symmetry (Fig. 27 ▸). Like in **9**, the disorder of the anion results in unrealistically close H⋯H con­tacts between anions across a crystallographic inversion center, and it is unlikely that anions directly related by inversion symmetry pack at the same time; however, any detailed analysis of the anion packing is hampered by the disorder. Treating all of the disordered parts of the anion as a whole, it can be seen that there are 25 con­tacts between the F atoms of the anion and H atoms of the cation, ranging in length from 2.097 (8) to 2.662 (8) Å, with an average con­tact distance of 2.421 (9) Å. The average overall anion-to-cation F⋯H con­tact distance is similar to what is seen in **9**. There are only two con­tacts between the anion N and cation H atoms, with a length of 2.77 (2) Å for the con­tact between N1′ and methyl atom H28*A*, and 2.677 (8) Å for the con­tact between N1′′ and H32. The second con­tact occurs on the face of the pyridine ring.

**Figure 28:**
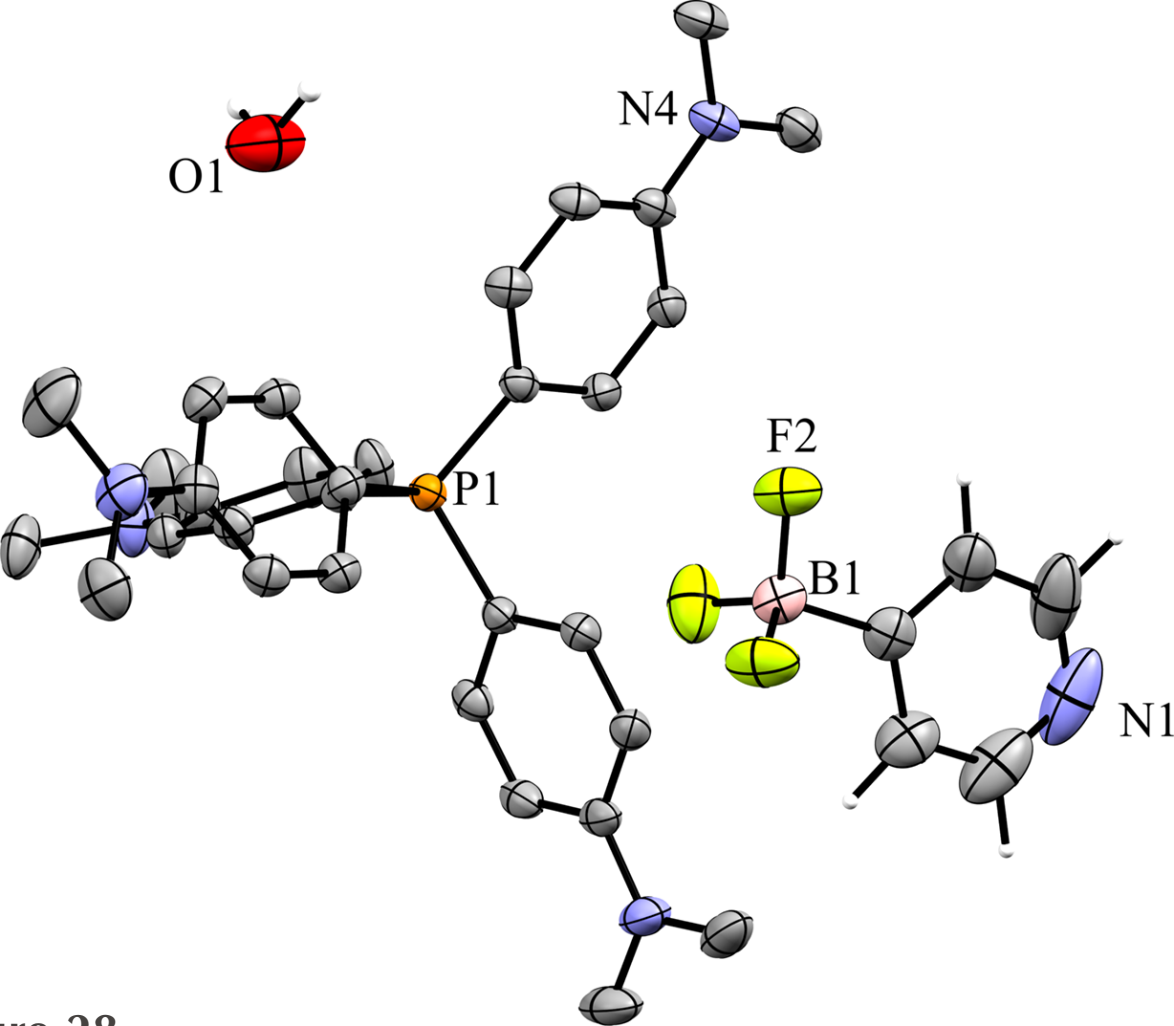
The asymmetric unit of **10h**.

**Figure 29:**
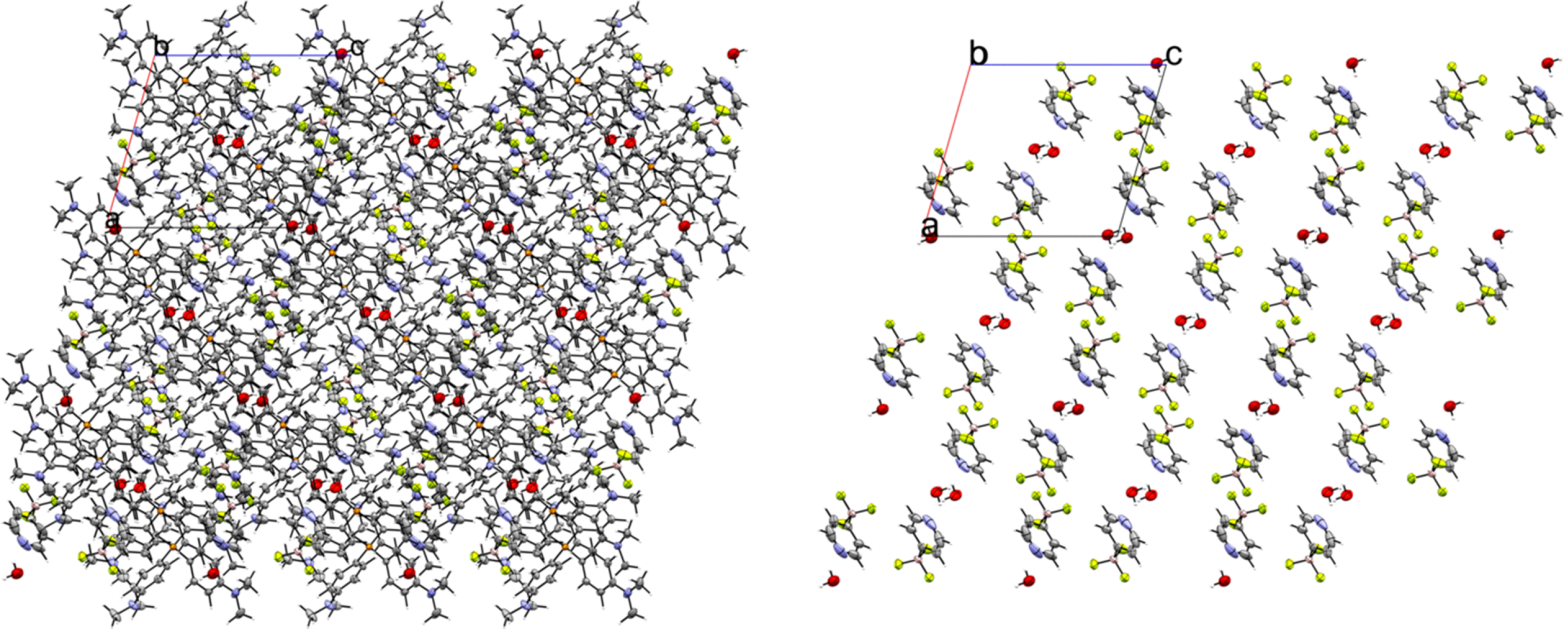
A 3×3×3 packed unit cell of **10h**, viewed along the crystallographic *b* axis. The image on the right has the cations hidden. Each water mol­ecule shown has a 50% occupancy, leading to an overall anion–water stoichiometry of 2:1.

When crystals of **10** are grown by evaporation from acetone, the com­pound is obtained as a hemihydrate (**10h**) in the monoclinic space *P*2_1_/*n* with *Z*′ = 1 (Fig. 28 ▸). The structure solution of this crystal form has both the cation and anion well resolved. Fig. 29 ▸ shows the packing of the crystal, viewed along the *b* axis. The anions form dimers linked by 2.02 (3) Å hy­dro­gen bonds between the pyridine N atom and a single water mol­ecule across a crystallographic inversion center, with the water mol­ecule positioned on the inversion center. There are no con­tacts between anions. The F atoms of the anion form ten cation–anion con­tacts in the crystal structure. These con­tacts range in length from 2.308 (2) to 2.638 (3) Å, with an average of 2.482 (2) Å. This is the longest cation–anion H⋯F average distance of all the salts with organic cations; however, it is also the only salt to have cocrystallized water, which impacts the packing of the anion. The pyridine N atom primarily forms a hy­dro­gen bond with the water mol­ecule, but there is also a 2.595 (4) Å N⋯H con­tact with a methyl H atom on atom C37 of the cation.

### Trends in cation–anion N⋯H and F⋯H con­tacts for salts 3–10 and 10h

Because the potassium salts **1** and **2** are not soluble in low-polarity solvents such as DCM, they are not well suited for use as charge-enhanced catalysts and the focus of the upcoming analyses will be on the more soluble salts with organic cations (**3**–**10** and **10h**). Table 2 ▸ shows the number and average lengths of the cation–anion N⋯H and F⋯H con­tacts for each of the salts containing organic cations. For all of the salts, the F⋯H con­tacts were more numerous and shorter in length than the N⋯H con­tacts. Examining the N⋯H con­tacts (excluding **10h**, in which the sole N⋯H con­tact of the anion is with the cocrystallized water mol­ecule), it can be seen that **3** has the shortest observed N⋯H con­tact at 2.583 (2) Å. Salt **10** has the longest N⋯H con­tact distance at 2.726 (14) Å (averaged across all parts of the disordered anion). Arranging the salts by average N⋯H con­tact distance gives the following order: **3** < **6** < **7** < **4** < **9** < **5** < **8** < **10**; however, it should be noted that the average lengths for salts **4** and **9**, **6** and **7**, and **8** and **10** have values that overlap when uncertainties are taken into con­sid­er­a­tion. For the F⋯H con­tact distances, salt **5** has the shortest average distance at 2.310 (3) Å. The longest average F⋯H con­tact distances are in **10h**; however, the presence of the water mol­ecule precludes this com­pound from direct com­parison to the others. Ordering the salts by average F⋯H con­tact distance gives: **5** < **4** < **3** < **9** < **10** < **7** < **8** < **6** < **10h**, noting that salts **3** and **4**, **9**, **6**, and **8**, and **7** and **10** have values that overlap when considering uncertainties. Across salts with the same cation, the position of the N atom in the pyridine ring of the anions has only a small effect on the average N⋯H con­tact distance for most of the salts, with the largest difference being seen in the MeO-Phos salts **7** and **8**, which differ by 0.092 (9) Å. Examining the F⋯H con­tacts of the 3- and 4-pyri­dyl tri­fluoro­borate anions with the same cation, it is found that the position of the pyridine N atom on the anion has little effect on the average F⋯H con­tact length of the TBA salts (**3** and **4**), the MeO-Phos salts (**7** and **8**), and the neat NMe_2_-Phos salts (**9** and **10**). There is a significant difference between the PPh_4_ salts **5** and **6**, with the average F⋯H con­tact distance being 0.132 (4) Å shorter in the 3-pyri­dyl salt **5** com­pared to the 4-pyri­dyl analogue **6**. The average F⋯H con­tact distance difference between **10** and **10h** is 0.061 (10) Å. Overall, there are no significant trends between the position of the pyridine N atom or the identity of the cation on the average lengths of the N⋯H and F⋯H con­tacts in the crystal structures containing organic cations.

## Hirshfeld surface analysis

**Figure 30:**
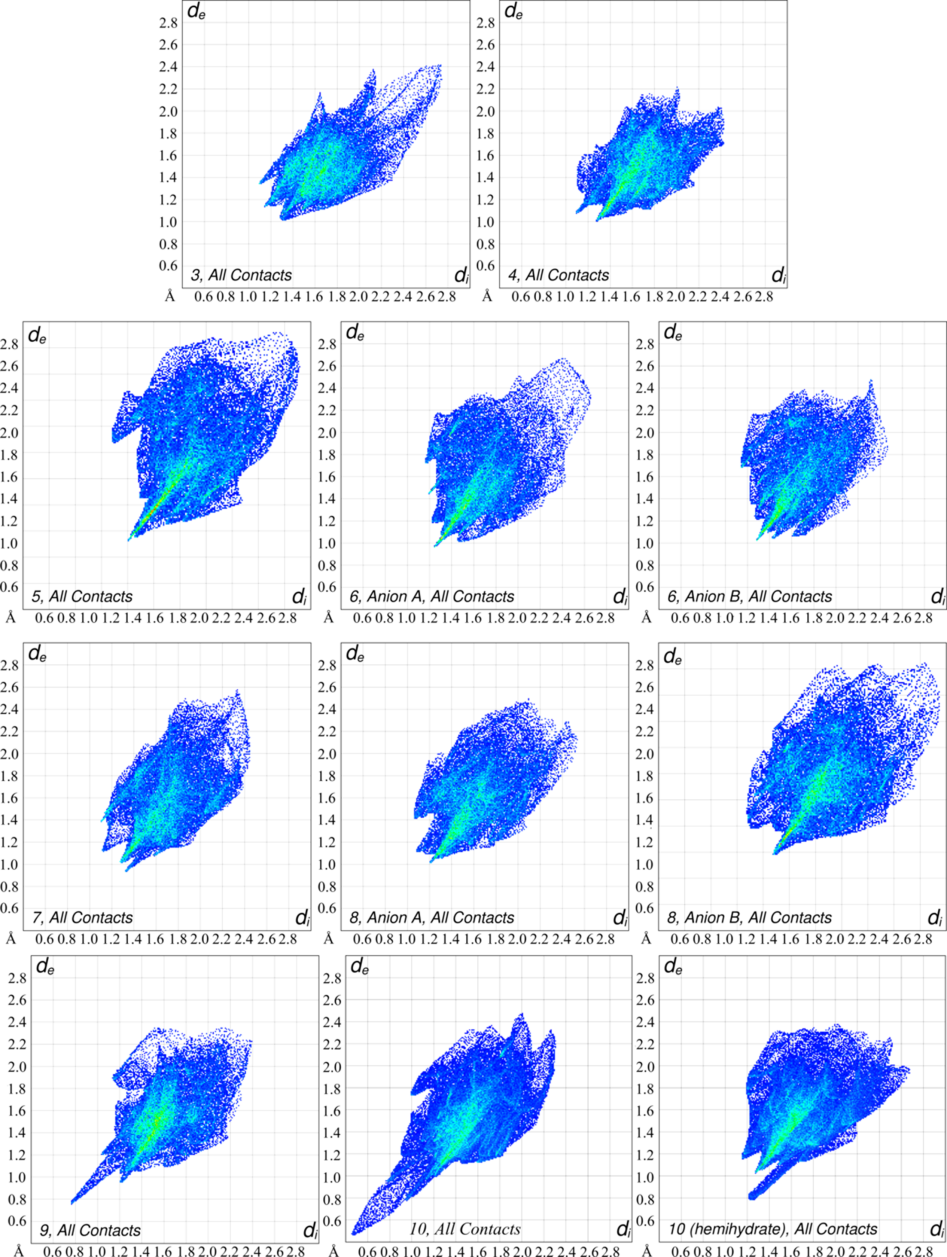
The Hirshfeld surface fingerprint plots for the anions in salts **3**–**10** and **10h**.

Hirshfeld surface analysis offers a unique way to visualize the chemical environment of different mol­ecules within a crystal (Spackman & Jayatilaka, 2009 ▸; Spackman *et al.*, 2021 ▸). Briefly, Hirshfeld surfaces are a means by which the electron density of the crystal is broken down into the contributions from each of the mol­ecules within the crystal. The distances of atoms to the Hirshfeld surface of a mol­ecule can be plotted for atoms both inside (*d*_i_) and outside (*d*_e_) of the surface to provide a unique plot, called a fingerprint plot, for each mol­ecule in a crystal structure. ‘Spikes’ in the direction of the lower left portion of the fingerprint plots (*i.e.* small *d*_i_/*d*_e_ distances) indicate the presence of close con­tacts between atoms inside and outside of the Hirshfeld surface, corresponding to close con­tacts between atoms in different mol­ecules. Fingerprint plots are colored ranging from blue to green to red by the number of contributing points of the Hirshfeld surface to the particular *d*_i_/*d*_e_ distance, with blue points having few contributions and red points having many. Fingerprint plots can be divided to show the types of inter­molecular con­tacts contributing to the surface and the percentage that each con­tact type contributes to the overall surface. Fig. 30 ▸ shows the fingerprint plots for the anions of salts **3**–**10** and **10h**. In the cases where the anion was disordered, all parts of the disordered anion were treated as one to generate a single Hirshfeld surface. While the fingerprint plots for each anion are unique, there is little difference in the fingerprint plots between the 3- and 4-pyri­dyl tri­fluoro­borate anions when paired with the same cation, particularly in the region of small *d*_i_/*d*_e_ values that correspond to close con­tacts between ions. Of these, the anions that appear the most different are **9**, **10**, and **10h**. The fingerprint plots of com­pounds **9** and **10** are similar, with both showing a sharp spike terminating in the 0.8/0.8 Å *d*_i_/*d*_e_ region (**9**) and the 0.6/0.86 Å *d*_i_/*d*_e_ region (**10**) (Fig. 30 ▸, bottom row, left and middle). These spikes are a result of unrealistic H⋯H con­tacts across the inversion center resulting from the disordered anions discussed in Section 3.5. Salt **10h** shows a spike terminating in the 1.2/0.8 Å *d*_i_/*d*_e_ region that corresponds to the hy­dro­gen bond between the anion and the water mol­ecule (Fig. 30 ▸, bottom row, right). Because of the presence of the cocrystallized water in **10h**, it is not surprising to see a large difference between this fingerprint plot and those of the neat forms **9** and **10**. Table 3 ▸ shows the breakdown of the major contributions of different types of con­tacts to the Hirshfeld surface of each anion. The Hirshfeld surfaces of the tetra­butyl­ammonium salts **3** and **4** have roughly the same N⋯H and C⋯H contributions, while the 4-pyri­dyl analogue has about 4% fewer F⋯H and 4% more H⋯H con­tacts. For the PPh_4_ salts **5** and **6**, there is very little difference in the amount that each type of con­tact contributes to the Hirshfeld surface, with the differences in the amount of each type of con­tact not exceeding 2.1%. The MeO-Phos salts **7** and **8** likewise show little difference in their Hirshfeld surface com­position, with similar N⋯H and O⋯H contributions. The 4-pyri­dyl borate anions in **8** have slightly higher F⋯H contributions. Salt **7** and anion *A* of **8** have similar 14.6 and 13.2% C⋯H contributions, and 33.0 and 34.0% H⋯H contributions, respectively.

Anion *B* of **8** is the most different in this regard with 17.6% C⋯H and 29.3% H⋯H contributions. The environments of the anions in the NMe_2_-Phos salts are again quite similar, with the biggest differences being in the N⋯H (9.0% for **9** and 6.8% for **10**) and C⋯H (14.0% for **9** and 16.0% for **10**) regions. Comparing salt **10** to **10h**, there is a fairly large difference in the F⋯H contributions, with **10h** having a smaller surface by 3.4%. Salt **10h** has an approximately 2% larger contribution of both N⋯H and C⋯H con­tacts com­pared to **10**.

Examining how the identity of the cation affects the Hirshfeld surface com­positions, it can be seen that the average contributions of F⋯H and N⋯H con­tacts to the Hirshfeld surfaces are similar, with F⋯H con­tacts accounting for 38.3, 35.1, 37.2, and 37.8%, and N⋯H con­tacts accounting for 9.0, 8.8, 8.4, and 8.1% for the TBA, PPh_4_, MeO-Phos, and NMe_2_-Phos salts, respectively. On average, the PPh_4_ salts have a higher C⋯H contribution of 22.5% com­pared to 13.6, 15.1, and 16.0% for the TBA, MeO-Phos, and NMe_2_-Phos salts, respectively. The TBA salts have the largest average contributions of H⋯H con­tacts at 39.2%, followed closely by 37.5% for the NMe_2_-Phos salts. In com­parison, the MeO-Phos and PPh_4_ salts have lower H⋯H con­tacts at 32.1 and 31.8% for the MeO-Phos and PPh_4_ salts, respectively. The MeO-Phos salts contain an average of 4.3% O⋯H contributions to the surface, contributions that are not present in the TBA salts, the PPh_4_ salts, and salt **9**, and only offer a very minor 0.2% contribution in **10h** due to the cocrystallized water. In summary, it can be seen that the position of the pyridine N atom has little effect on the overall environment of the anions across the salts, regardless of the cation, and that changing the cation primarily affects the amount of C⋯H and H⋯H con­tacts for the anions.

## Comparisons to reactivity

**Figure 31:**
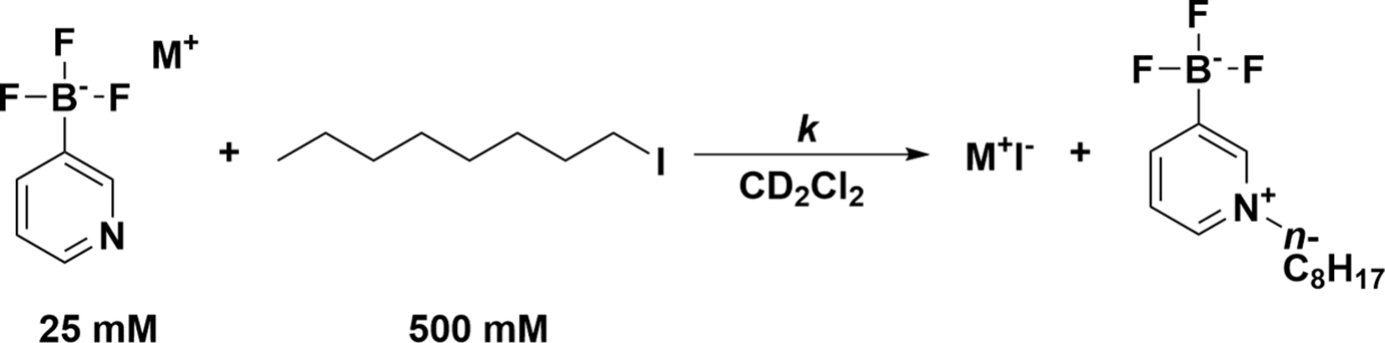
The model S_N_2 reaction for evaluating the nucleophilicity of the 3-pyri­dyl tri­fluoro­borate salts. The 4-pyri­dyl tri­fluoro­borate salts were evaluated in the same manner.

In contrast to polar solvents, where ion pairs exist primarily as solvated ‘free’ ions, ionic com­pounds in non-polar solvents exist primarily as ion pairs or higher-order aggregates, especially as the concentration of the salt increases (Marcus & Hefter, 2006 ▸). Inter­actions between the anions and cations in the crystalline state may be present in the solvated ion pairs and have a correlation to the reactivity of the salts. The nucleophilicity for each of the salts was evaluated by com­paring the pseudo-first-order rate constants of the S_N_2 reaction between each salt and 1-iodo­octane in di­chloro­methane (Fig. 31 ▸). Given the low dielectric constant of the solvent, it is not unreasonable to expect that the salts exist as ion pairs or higher-order aggregates while in solution, with con­tacts between the ions possibly affecting the reactivity of the salts. As such, it is worthwhile to investigate if trends exist between the crystallographic environment of the anions and their nucleophilicity. Salts **3** and **4** were evaluated previously using this reaction (Dempsey, Lovstedt *et al.*, 2023 ▸), although the concentrations of reagents are slightly different in this work. The neutral nucleophiles pyridine and DMAP were also evaluated in this manner. Table 4 ▸ gives the reaction rate constants and relative rates for pyridine, DMAP, and salts **3**–**10**. Salts **1** and **2** are insoluble in DCM and as a result could not be evaluated.

**Figure 32:**
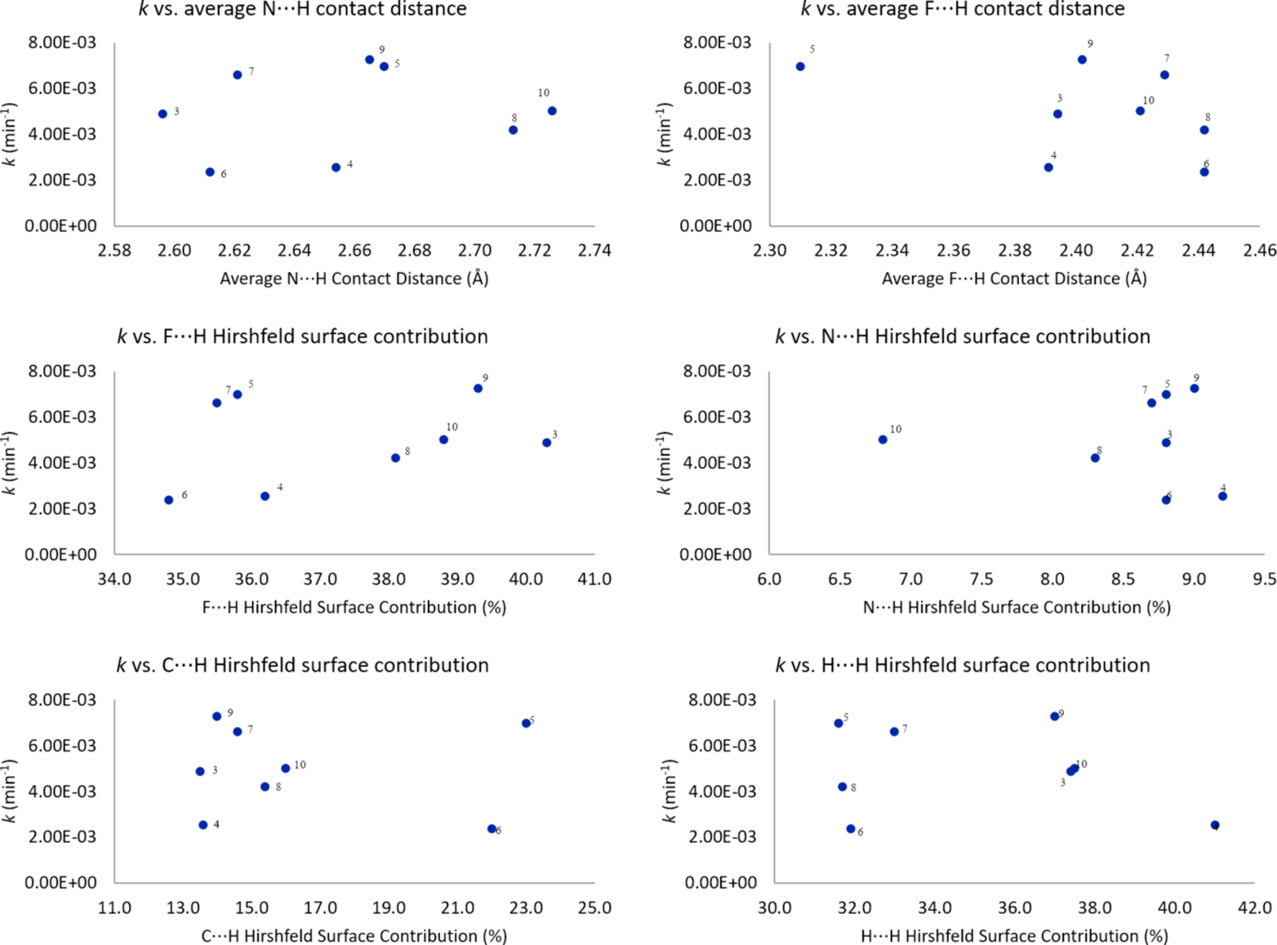
The plots of *k versus* various crystallographically determined parameters. The Hirshfeld surface contributions for **6** and **8** are the average across both anions.

DMAP showed a moderate 6.6-fold rate increase over pyridine. All of the anionic catalysts had a faster rate that DMAP, however, the difference in reaction rates is small, with the fastest reacting salt **9** having a rate about four times greater than DMAP. Consistent with previous work (Demp­sey, Lovstedt *et al.*, 2023 ▸), the 3-pyri­dyl tri­fluoro­borate anions were all more reactive than their 4-pyri­dyl analogues. Salt **3** was the slowest reacting 3-pyri­dyl tri­fluoro­borate salt, while the other three 3-pyri­dyl tri­fluoro­borate salts **5**, **7**, and **9** all had roughly the same rates. Inter­estingly, the trend is quite different for the 4-pyri­dyl tri­fluoro­borate anions. For these anions, the TBA salt **4** and PPh_4_ salt **6** had roughly the same rates, while the MeO-Phos salt **8** and NMe_2_-Phos salt **10** exhibited faster rates. Salt **10** was about as reactive as **3**, while **4** reacted about half as fast as **3**. Fig. 32 ▸ shows plots of *k versus* average N⋯H and F⋯H con­tact distances, as well as F⋯H, N⋯H, C⋯H, and H⋯H Hirshfeld surface contributions. It is clear that no trend exists between the reactivity of the salts and the selected crystallographic properties. Thus, at least in the case of the pyri­dyl tri­fluoro­borate salts and model S_N_2 reaction examined in this study, no correlations are observed between solid-state crystal packing and solution-state reactivity.

## Conclusion

This work examined the trends between the crystal structures and nucleophilicity of several 3- and 4-pyri­dyl tri­fluoro­borate salts with an assortment of cations. For the salts with organic cations, it was found that the identity of the cation and the position of the N atom on the pyridine ring changed the manner in which the anions packed within the crystals, and that the lengths and number of N⋯H and F⋯H con­tacts did not show any trend with the position of the pyridine N atom or the identity of the cation. Hirshfeld surfaces were used to probe the total crystallographic environments of the anions across the salts, and it was determined that the position of the pyridine N atom had little effect on the overall environment of the anions across the salts regardless of the cation, and that changing the cation had primarily affected the amount of C⋯H and H⋯H con­tacts for the anions. The nucleophilicity of the salts with organic cations was determined using a model S_N_2 reaction, and all of the anionic salts showed moderate rate increases over pyridine, with the 3-pyri­dyl tri­fluoro­borate anions being more reactive than their 4-pyri­dyl counterparts. The effect of the counter-ion on the reactivity of the salts was different between the 3- and 4-pyri­dyl anions, suggesting that cation coordination behavior is dependent on the identity of the anion. Lastly, it was determined that cation–anion N⋯H and F⋯H con­tact lengths and anion Hirshfeld surface com­positions had no correlation to the reactivity of the salts.

## Related literature

The following references are cited in the supporting information for this article: Batey & Quach (2001 ▸); Li *et al.* (2009 ▸); Petruzziello *et al.* (2013 ▸); Van Geet (1970 ▸).

## Supplementary Material

Crystal structure: contains datablock(s) 5, 6, 9, 7, 10h, 8, 4, 1, 2, 3, 10, global. DOI: 10.1107/S2053229625010629/dg3080sup1.cif

Structure factors: contains datablock(s) 5. DOI: 10.1107/S2053229625010629/dg30805sup6.hkl

Structure factors: contains datablock(s) 6. DOI: 10.1107/S2053229625010629/dg30806sup7.hkl

Structure factors: contains datablock(s) 8. DOI: 10.1107/S2053229625010629/dg30808sup9.hkl

Structure factors: contains datablock(s) 4. DOI: 10.1107/S2053229625010629/dg30804sup5.hkl

Structure factors: contains datablock(s) 1. DOI: 10.1107/S2053229625010629/dg30801sup2.hkl

Structure factors: contains datablock(s) 2. DOI: 10.1107/S2053229625010629/dg30802sup3.hkl

Structure factors: contains datablock(s) 3. DOI: 10.1107/S2053229625010629/dg30803sup4.hkl

Structure factors: contains datablock(s) 10. DOI: 10.1107/S2053229625010629/dg308010sup11.hkl

Supporting information file. DOI: 10.1107/S2053229625010629/dg30805sup17.cml

Supporting information file. DOI: 10.1107/S2053229625010629/dg30806sup18.cml

Supporting information file. DOI: 10.1107/S2053229625010629/dg30809sup21.cml

Supporting information file. DOI: 10.1107/S2053229625010629/dg30807sup19.cml

Supporting information file. DOI: 10.1107/S2053229625010629/dg308010hsup23.cml

Supporting information file. DOI: 10.1107/S2053229625010629/dg30808sup20.cml

Supporting information file. DOI: 10.1107/S2053229625010629/dg30804sup16.cml

Supporting information file. DOI: 10.1107/S2053229625010629/dg30801sup13.cml

Supporting information file. DOI: 10.1107/S2053229625010629/dg30802sup14.cml

Supporting information file. DOI: 10.1107/S2053229625010629/dg30803sup15.cml

Supporting information file. DOI: 10.1107/S2053229625010629/dg308010sup22.cml

Supporting information file. DOI: 10.1107/S2053229625010629/dg3080sup24.pdf

CCDC references: 2498616, 2471467, 2471466, 2471465, 2471468, 2471472, 2471474, 2471471, 2471473, 2471470, 2471469

## Figures and Tables

**Table:** Experiments were carried out using a Bruker D8 Venture diffractometer equipped with a PHOTON III CPAD detector.

	**1**	**2**	**3**	**4**
Crystal data				
Chemical formula	K^+^·C_5_H_4_BF_3_N^−^	K^+^·C_5_H_4_BF_3_N^−^·H_2_O	C_16_H_36_N^+^·C_5_H_4_BF_3_N^−^	C_16_H_36_N^+^·C_5_H_4_BF_3_N^−^
*M*_r_	185.00	203.02	388.36	388.36
Crystal system, space group	Orthorhombic, *P*2_1_2_1_2_1_	Monoclinic, *P*2_1_/*c*	Monoclinic, *P*2_1_/*n*	Monoclinic, *P*2_1_/*c*
Temperature (K)	100	100	100	100
*a*, *b*, *c* (Å)	5.8637 (2), 7.2260 (3), 16.5602 (8)	10.8271 (15), 8.5041 (11), 8.8435 (12)	9.4405 (7), 15.9663 (10), 15.4847 (11)	8.8089 (6), 13.2030 (8), 19.9075 (14)
α, β, γ (°)	90, 90, 90	90, 90.043 (5), 90	90, 100.159 (2), 90	90, 98.652 (2), 90
*V* (Å^3^)	701.67 (5)	814.26 (19)	2297.4 (3)	2289.0 (3)
*Z*	4	4	4	4
Radiation type	Mo *K*α	Mo *K*α	Mo *K*α	Mo *K*α
μ (mm^−1^)	0.74	0.65	0.08	0.08
Crystal size (mm)	0.16 × 0.09 × 0.04	0.17 × 0.17 × 0.07	0.32 × 0.06 × 0.06	0.08 × 0.07 × 0.06

Data collection				
Absorption correction	Multi-scan (*SADABS*; Krause *et al.*, 2015 ▸)	Multi-scan (*SADABS*; Krause *et al.*, 2015 ▸)	Multi-scan (*SADABS*; Krause *et al.*, 2015 ▸)	Multi-scan (*SADABS*; Krause *et al.*, 2015 ▸)
*T*_min_, *T*_max_	0.665, 0.746	0.579, 0.745	0.662, 0.745	0.705, 0.745
No. of measured, independent and observed [*I* > 2σ(*I*)] reflections	11196, 1733, 1694	19163, 1723, 1514	23792, 4228, 3058	24696, 4206, 2997
*R*_int_	0.034	0.075	0.071	0.059
(sin θ/λ)_max_ (Å^−1^)	0.666	0.632	0.605	0.603

Refinement				
*R*[*F*^2^ > 2σ(*F*^2^)], *wR*(*F*^2^), *S*	0.019, 0.060, 0.99	0.032, 0.085, 1.11	0.043, 0.106, 1.03	0.040, 0.098, 1.03
No. of reflections	1733	1723	4228	4206
No. of parameters	100	158	248	248
No. of restraints	0	176	0	0
H-atom treatment	H-atom parameters constrained	H atoms treated by a mixture of independent and constrained refinement	H-atom parameters constrained	H-atom parameters constrained
Δρ_max_, Δρ_min_ (e Å^−3^)	0.27, −0.17	0.47, −0.32	0.20, −0.19	0.19, −0.20
Absolute structure	Flack *x* determined using 680 quotients [(*I*^+^) − (*I*^−^)]/[(*I*^+^) + (*I*^−^)] (Parsons *et al.*, 2013 ▸)	–	–	–
Absolute structure parameter	0.008 (16)	–	–	–

**Table:** 

	**5**	**6**	**7**	**8**
Crystal data				
Chemical formula	C_24_H_20_P^+^·C_5_H_4_BF_3_N^−^	C_24_H_20_P^+^·C_5_H_4_BF_3_N^−^	C_32_H_36_O_8_P^+^·C_5_H_4_BF_3_N^−^	C_32_H_36_O_8_P^+^·C_5_H_4_BF_3_N^−^
*M*_r_	485.27	485.27	725.48	725.48
Crystal system, space group	Monoclinic, *Pc*	Triclinic, *P*1	Monoclinic, *P*2_1_/*n*	Triclinic, *P* 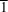
Temperature (K)	126	125	150	150
*a*, *b*, *c* (Å)	10.1711 (2), 9.3254 (2), 13.7076 (3)	9.4898 (3), 10.2000 (3), 14.4239 (5)	21.3755 (8), 7.5380 (3), 23.0617 (8)	14.702 (4), 15.141 (5), 16.017 (5)
α, β, γ (°)	90, 108.701 (1), 90	99.787 (2), 101.625 (2), 113.231 (2)	90, 99.998 (2), 90	84.473 (11), 88.059 (10), 85.136 (10)
*V* (Å^3^)	1231.52 (5)	1207.43 (7)	3659.5 (2)	3534.7 (18)
*Z*	2	2	4	4
Radiation type	Cu *K*α	Cu *K*α	Mo *K*α	Mo *K*α
μ (mm^−1^)	1.33	1.36	0.14	0.15
Crystal size (mm)	0.25 × 0.25 × 0.24	0.10 × 0.07 × 0.06	0.31 × 0.07 × 0.05	0.16 × 0.07 × 0.05

Data collection				
Absorption correction	Multi-scan (*SADABS*; Krause *et al.*, 2015 ▸)	Multi-scan (*SADABS*; Krause *et al.*, 2015 ▸)	Multi-scan (*SADABS*; Krause *et al.*, 2015 ▸)	Multi-scan (*TWINABS*; Sheldrick, 2012 ▸)
*T*_min_, *T*_max_	0.661, 0.754	0.605, 0.754	0.661, 0.746	0.682, 0.745
No. of measured, independent and observed [*I* > 2σ(*I*)] reflections	24137, 4783, 4708	24637, 9470, 9274	43069, 9058, 6479	12796, 12796, 9875
*R*_int_	0.035	0.034	0.052	–
(sin θ/λ)_max_ (Å^−1^)	0.625	0.638	0.667	0.600

Refinement				
*R*[*F*^2^ > 2σ(*F*^2^)], *wR*(*F*^2^), *S*	0.029, 0.076, 1.02	0.038, 0.106, 1.06	0.046, 0.123, 1.02	0.053, 0.132, 1.06
No. of reflections	4783	9470	9058	12796
No. of parameters	316	631	468	967
No. of restraints	2	3	0	30
H-atom treatment	H-atom parameters constrained	H-atom parameters constrained	H-atom parameters constrained	H-atom parameters constrained
Δρ_max_, Δρ_min_ (e Å^−3^)	0.25, −0.26	0.35, −0.26	0.45, −0.35	0.44, −0.35
Absolute structure	Flack *x* determined using 2179 quotients [(*I*^+^) − (*I*^−^)]/[(*I*^+^) + (*I*^−^)] (Parsons *et al.*, 2013 ▸)	Flack *x* determined using 4183 quotients [(*I*^+^) − (*I*^−^)]/[(*I*^+^) + (*I*^−^)] (Parsons *et al.*, 2013 ▸)	–	–
Absolute structure parameter	−0.011 (12)	0.000 (15)	–	–

**Table:** 

	**9**	**10**	**10h**
Crystal data			
Chemical formula	C_32_H_40_N_4_P^+^·C_5_H_4_BF_3_N^−^	C_32_H_40_N_4_P^+^·C_5_H_4_BF_3_N^−^	C_32_H_40_N_4_P^+^·C_5_H_4_BF_3_N^−^·0.5H_2_O
*M*_r_	657.55	657.55	666.56
Crystal system, space group	Monoclinic, *P*2_1_/*n*	Monoclinic, *P*2_1_/*n*	Monoclinic, *P*2_1_/*n*
Temperature (K)	150	100	150
*a*, *b*, *c* (Å)	15.802 (2), 12.0571 (15), 18.121 (2)	15.9231 (9), 11.5958 (6), 18.7330 (12)	11.9373 (3), 23.4315 (7), 13.0949 (3)
α, β, γ (°)	90, 94.405 (3), 90	90, 96.696 (2), 90	90, 105.938 (1), 90
*V* (Å^3^)	3442.4 (8)	3435.3 (3)	3521.96 (16)
*Z*	4	4	4
Radiation type	Mo *K*α	Mo *K*α	Mo *K*α
μ (mm^−1^)	0.13	0.13	0.13
Crystal size (mm)	0.17 × 0.10 × 0.05	0.34 × 0.25 × 0.04	0.16 × 0.16 × 0.10

Data collection			
Absorption correction	Multi-scan (*SADABS*; Krause *et al.*, 2015 ▸)	Multi-scan (*SADABS*; Krause *et al.*, 2015 ▸)	Multi-scan (*SADABS*; Krause *et al.*, 2015 ▸)
*T*_min_, *T*_max_	0.629, 0.746	0.629, 0.745	0.644, 0.745
No. of measured, independent and observed [*I* > 2σ(*I*)] reflections	31607, 6295, 4970	52555, 6526, 4822	31433, 7198, 5810
*R*_int_	0.037	0.060	0.032
(sin θ/λ)_max_ (Å^−1^)	0.602	0.610	0.625

Refinement			
*R*[*F*^2^ > 2σ(*F*^2^)], *wR*(*F*^2^), *S*	0.039, 0.106, 1.04	0.051, 0.142, 1.04	0.044, 0.117, 1.04
No. of reflections	6295	6526	7198
No. of parameters	487	525	448
No. of restraints	186	367	4
H-atom treatment	H-atom parameters constrained	H-atom parameters constrained	H atoms treated by a mixture of independent and constrained refinement
Δρ_max_, Δρ_min_ (e Å^−3^)	0.21, −0.31	0.96, −0.35	0.55, −0.46

Computer programs: *APEX5* (Bruker, 2023 ▸), *SAINT* (Bruker, 2016 ▸), *SHELXT2018* (Sheldrick, 2015*a* ▸), *SHELXL2019* (Sheldrick, 2015*b* ▸), *Mercury* (Macrae *et al.*, 2020 ▸), and *ShelXle* (Hübschle *et al.*, 2011 ▸).

**Table 2:** The number and average lengths of cation–anion N⋯H and F⋯H con­tacts for each of the salts containing organic cations

Salt	Number of N⋯H con­tacts	Average N⋯H con­tact distance (Å)	Number of F⋯H con­tacts	Average F⋯H con­tact distance (Å)
**3**	2	2.596 (3)	4	2.394 (2)
**4**	1	2.654 (2)	8	2.391 (2)
**5**	4	2.670 (4)	9	2.310 (3)
**6**	5^*a*^	2.612 (6)^*b*^	18^*a*^	2.442 (4)^*b*^
**7**	3	2.621 (3)	8	2.429 (3)
**8**	9^*a*,*c*^	2.713 (9)^*b*,*d*^	21^*a*,*c*^	2.441 (8)^*b*,*d*^
**9**	2^*c*^	2.665 (15)^*d*^	12^*c*^	2.402 (9)^*d*^
**10**	2^*c*^	2.726 (14)^*d*^	21^*c*^	2.421 (9)^*d*^
**10h**	1	2.595 (2)	10	2.482 (4)

Notes: (*a*) the total number of con­tacts across both anions; (*b*) the average value across both anions; (*c*) the total number of con­tacts between all parts of the disordered anion; (*d*) the average values between all parts of the disordered anion.

**Table 3:** Contributions (%) of selected con­tacts to the Hirshfeld surfaces for all of the anions Minor contributors have been omitted, leading to a <100% sum of contributions for some of the anions.

	Contribution to anion Hirshfeld surface				
Salt	F⋯H	N⋯H	C⋯H	H⋯H	O⋯H
**3**	40.3	8.8	13.5	37.4	NA
**4**	36.2	9.2	13.6	41.0	NA
**5**	35.8	8.8	23.4	31.6	NA
**6**, Anion *A*	35.6	8.4	22.7	32.2	NA
**6**, Anion *B*	34.0	9.1	21.3	31.6	NA
**7**	35.5	8.7	14.6	33.0	4.4
**8**, Anion *A*	38.7	8.0	13.2	34.0	4.1
**8**, Anion *B*	37.4	8.6	17.6	29.3	4.3
**9**	39.3	9.0	14.0	37.0	NA
**10**	38.8	6.8	16.0	37.5	NA
**10h**	35.4	8.6	17.9	37.9	0.2

**Table 4:** Pseudo-first-order reaction-rate constants and relative rates for each com­pound in the model S_N_2 reaction at 298 K

Compound	*k* (min^−1^)	*k* (relative to pyridine)
Pyridine	0.000259	1.0
DMAP	0.00171	6.6
**3**	0.00488	18.9
**4**	0.00254	9.8
**5**	0.00698	26.9
**6**	0.00237	9.1
**7**	0.00661	25.5
**8**	0.00420	16.2
**9**	0.00727	28.1
**10**	0.00502	19.4

## Data Availability

The crystallographic information files for structures **1**–**10** and **10h** are available in the supporting information and from the Cambridge Crystallographic Data Centre (CCDC). The CCDC deposition numbers for each crystal structure, kinetics, NMR, and additional crystallographic information can be found in the supporting information for this article.
