# Supplementary material for: Charge-enhanced pyri­dyl tri­fluoro­borate organocatalysts: crystal structures and reactivity
Source: Acta Crystallogr C Struct Chem. 2026 Jan 1;82(Pt 1):8–26. doi: 10.1107/S2053229625010629 (PMC12809446; doi:10.1107/S2053229625010629)
Supplement: Supplementary file 21 [file c-82-00008-sup24.pdf]

STRUCTURAL CHEMISTRY

Volume 81 (2025)

Supporting information for article:

Charge-enhanced pyridyl trifluoroborate organocatalysts: crystal structures and reactivity

Alex Lovstedt, Stephen H. Dempsey and Steven Kass

S1. Synthesis

S1.1. General

Tetraphenylphosphonium chloride and bromide were purchased from Oakwood Chemical. 3- and 4-Pyridylboronic acids were purchased from Ambeed. Methanol, dichloromethane (DCM), tetrahydrofuran (THF), diethyl ether and anhydrous K_2CO_3 were obtained from Fisher Scientific. Deuterated solvents were acquired from Cambridge Isotope Laboratories and all other reagents and solvents were purchased from Sigma-Aldrich. Substituted tetraarylphosphonium chlorides were previously synthesized according to published procedures (Dempsey & Kass, 2022). Glassware for air free reactions was flame dried or placed in an oven at 120 °C for at least one hour before use. CD_2Cl_2 used for rate constant determinations was dried using 3 Å molecular sieves and stored and handled in a nitrogen atmosphere glovebox. NMR spectra were recorded on Bruker Avance III spectrometers equipped with either a 400 or 500 MHz magnet. For some compounds, certain ^{13}C resonances are weak enough (particularly the quaternary carbons adjacent to the boron center) as to be invisible even with many scans on an LN_2 cryoprobe equipped spectrometer. Attempts to observe the peaks through the proton channel via HMBC were unsuccessful. The combination of the NMR data, HRMS data, and crystal structures provide definitive confirmation of the compounds' identity. Spectra with missing ^{13}C resonances are noted in the corresponding experimental description. Uncorrected melting points were determined with a Thomas Hoover Uni-Melt apparatus using unsealed capillary tubes. High resolution mass spectrometry with electrospray ionization (HRMS-ESI) data were collected using a Bruker ESI-BioTOF instrument for compounds **1**, **2**, **3**, **5**, **6** ,**7**, and **9**. HRMS-ESI spectra for compounds **4**, **8**, and **10** were collected on a SciEx X500R QTOF-MS instrument.

S1.2. Potassium trifluoro(pyridin-3-yl)borate (1).

This compound was synthesized according to a previous report with only minor alterations (Petruzziello *et al.*, 2013). 3-Pyridylboronic acid (1.85 g, 15.1 mmol, 1.00 eq.) was suspended in 10 mL of methanol in a 50 mL round-bottomed flask under an argon atmosphere and cooled with an ice water bath. KHF_2 (3.53 g, 45.2 mmol, 2.99 eq) and water (12.0 mL, 0.67 mol, 44.4 eq.) was added in one part. The cooled mixture was stirred for 30 minutes before being allowed to warm to room temperature where it was maintained for 18 hours. The solvent was then removed under reduced pressure and the resulting crude white powder was boiled in 80 mL of acetone and subsequently filtered. The solid was returned to the 125 mL Erlenmeyer flask and the boiling and filtering process was repeated three times in total. Concentration of the combined acetone solutions to ~100 mL was followed by the addition of 3.00 g (21.7 mmol, 1.4 eq.) of K_2CO_3 and the resulting suspension was boiled overnight. Filtration of the hot mixture and concentration of the solution under reduced

pressure led to a solid that was dissolved in a minimal amount of methanol which was then triturated into 200 mL of ether. The resulting solid was isolated by filtration and dried under vacuum to afford 1.88 g (10.1 mmol, 65% yield) of the potassium salt with spectra that are in accord with the literature.(Petruzziello *et al.*, 2013) Crystals suitable for X-ray diffraction were grown by evaporation from acetonitrile. ^{1}H-NMR (500 MHz, CD_3OD): δ 8.58 (s, 1H), 8.28 (dd, *J* = 5.0, 1.9 Hz, 1H), 7.92 (dt, *J* = 7.5, 1.8 Hz, 1H), 7.24 (dd, *J* = 7.5, 5.0 Hz, 1H). ^{11}B-NMR (128 MHz, CD_3OD): δ 3.32 (d, $J_{B\text{-}F}$ = 51.8 Hz). ^{13}C{H}-NMR (126 MHz, CD_3OD): [missing 1 resonance] δ 152.4, 146.9, 141.8, 124.3. ^{19}F{H}-NMR (471 MHz, CD_3OD): δ -144.21 (q, $J_{F\text{-}B}$ = 37.0 Hz).

S1.3. Potassium trifluoro(pyridin-4-yl)borate (2).

4-Pyridylboronic acid (2.15 g, 17.5 mmol, 1.00 eq.) and KHF_2 (4.09 g, 52.4 mmol, 2.99 eq.) afforded the crude trifluoroborate using the same procedure as for the trifluoro(pyridin-3-yl)borate. The crude solid material was taken up in minimal methanol (~80 mL) and triturated into 200 mL of ether to afford 1.58 g (8.50 mmol, 49% yield) of the potassium salt. This compound previously was commercially available.(Li *et al.*, 2009) Crystals suitable for X-ray diffraction were grown by evaporation from acetonitrile. ^{1}H-NMR (500 MHz, acetone-d_6): δ 8.26 (d, *J* = 5.5 Hz, 2H), 7.35 (d, *J* = 4.6 Hz, 2H). ^{11}B-NMR (161 MHz, CD_3OD): δ 2.75 (d, $J_{B\text{-}F}$ = 42.9 Hz). ^{13}C{H}-NMR (126 MHz, CD_3OD): [missing 1 resonance] δ 147.5, 128.7. ^{19}F{H}-NMR (471 MHz, acetone-d_6): δ -144.62 (q, $J_{F\text{-}B}$ = 49.4 Hz).

S1.4. Tetrabutylammonium trifluoro(pyridin-3-yl)borate (3).

In a 20 mL vial, potassium trifluoro(pyridin-3-yl)borate (231 mg, 1.25 mmol, 1.00 eq.) and tetrabutylammonium chloride (347 mg, 1.25 mmol, 1.00 eq.) were dissolved in acetonitrile and stirred for 30 minutes. The resulting suspension was filtered through tightly packed column containing celite and concentrated under reduced pressure to afford 474.8 mg (1.22 mmol, 98% yield) of the tetrabutylammonium salt as a white powder (MP = 59-61 °C). Crystals suitable for X-ray diffraction were grown by evaporation from acetonitrile. Spectra are in agreement with literature values.(Batey & Quach, 2001) ^{1}H-NMR (500 MHz, $CDCl_3$): δ 8.71 (s, 1H), 8.34 (d, *J* = 4.9 Hz, 1H), 7.84 (d, *J* = 7.6 Hz, 1H), 7.09 (t, *J* = 6.2 Hz, 1H), 3.02 (t, *J* = 7.4 Hz, 8H), 1.47 (quintet, *J* = 7.6 Hz, 8H), 1.31 (sextet, *J* = 7.4 Hz, 8H), 0.93 (t, *J* = 7.4 Hz, 12H). ^{11}B-NMR (161 MHz, $CDCl_3$): δ 3.24. ^{13}C{H}-NMR (126 MHz, $CDCl_3$): δ 153.0, 146.8, 139.56, 122.6, 58.3, 23.7, 19.5, 13.5. ^{19}F{H}-NMR (471 MHz, $CDCl_3$): δ -141.67. HRMS-ESI calculated for $C_{16}H_{36}N^+$ $(M - C_5H_4BF_3N^-)^+$ 242.2848, found 242.2846; calculated for $C_5H_4BF_3N^-$ $(M - NBu_4^+)^-$ 146.0394, found 146.0388.

S1.5. Tetrabutylammonium trifluoro(pyridin-4-yl)borate (4).

Potassium trifluoro(pyridin-4-yl)borate (24.8 mg, 0.13 mmol, 1.4 eq.) and tetrabutylammonium chloride (36.4 mg, 0.09 mmol, 1 eq.) were reacted using the same procedure as **3**. After removal of

acetonitrile, the residue was dissolved in DCM to remove any residual potassium trifluoro(pyridin-4-yl)borate. Solvent was removed, and the crude material was recrystallized from DCM/Ether to give 29.8 mg of **4** (0.077 mmol, 59% yield). (MP 74-76 °C). Crystals suitable for diffraction were obtained from the recrystallization step. ^{1}H-NMR (400 MHz, CD_2Cl_2): δ 8.35 (br. s 2H), 7.39 (br. s, 2H), 3.08-3.03 (m, 8H), 1.58-1.50 (m, 8H), 1.36 (sextet, *J* = 7.3 Hz, 8H), 0.97 (t, *J* = 7.2 Hz, 12 H). ^{11}B-NMR (128 MHz, CD_2Cl_2): δ 2.72 (q, J_{B-F} = 52.6 Hz). ^{13}C{H}-NMR (101 MHz, CD_2Cl_2): [missing 1 resonance] δ 148.3, 127.6, 59.0, 24.1, 20.0, 13.7. ^{19}F{H}-NMR (376 MHz, CD_2Cl_2): δ -143.75 (dd, J_{F-B} = 39.3 Hz, 93.4 Hz). HRMS-ESI calculated for $C_{16}H_{36}N^+$ (M - $C_5H_4BF_3N^-$)$^+$ 242.2848, found 242.2835; calculated for $C_5H_4BF_3N^-$ (M - NBu_4^+)$^-$ 146.0394, found 146.0388.

S1.6. Tetraphenylphosphonium trifluoro(pyridin-3-yl)borate (5).

Tetraphenylphosphonium chloride (600 mg, 1.60 mmol, 1.00 eq.) and potassium trifluoro(pyridin-3-yl)borate (608 mg, 1.62 mmol, 1.01 eq.) were dissolved in 5 mL of DCM and stirred vigorously with 5 mL of dilute K_2CO_3 in water for 1 hour. Upon addition of 10 mL of DCM and separation of the organic layer, concentration under reduced pressure afforded 738 mg (1.52 mmol, 94% yield) of the tetraphenylphosphonium salt as a white powder (MP = 174-177 °C). Crystals suitable for X-ray diffraction were grown by evaporation from an acetone solution of the salt. ^{1}H-NMR (500 MHz, $CDCl_3$): δ 8.68 (s, 1H), 8.25 (d, *J* = 5.0 Hz, 1H), 7.94 (d, *J* = 7.4 Hz, 1H), 7.86 (td, *J* = 7.4, 2.0 Hz, 4H), 7.73 (td, *J* = 7.9, 3.5 Hz, 8H), 7.63 – 7.50 (m, 8H), 7.07 (dd, *J* = 7.4, 4.9 Hz, 1H). ^{11}B-NMR (161 MHz, $CDCl_3$): δ 3.14 (d, J_{B-F} = 52.0 Hz). ^{13}C{H}-NMR (126 MHz, $CDCl_3$): δ 152.8, 146.1, 140.2, 135.8 (d, J_{P-C} = 3.2 Hz), 134.4 (d, J_{P-C} = 10.3 Hz), 130.8 (d, J_{P-C} = 12.8 Hz), 122.5, 117.5 (d, J_{P-C} = 89.5 Hz). ^{19}F{H}-NMR (471 MHz, $CDCl_3$): δ -144.06. ^{31}P{H}-NMR (203 MHz, $CDCl_3$): δ 23.13. HRMS-ESI calculated for $C_{24}H_{20}P^+$ (M - $C_5H_4BF_3N^-$)$^+$ 339.1298, found 339.1304; calculated for $C_5H_4BF_3N^-$ (M - PPh_4^+)$^-$ 146.0394, found 146.0399.

S1.7. Tetraphenylphosphonium trifluoro(pyridin-4-yl)borate (6).

The compound was synthesized via the same procedure as **5**. Tetraphenylphosphonium chloride (328 mg, 0.875 mmol, 1.00 eq.) and potassium trifluoro(pyridin-4-yl)borate (170 mg, 0.919 mmol, 1.05 eq.) afforded 395 mg (0.813 mmol, 93% yield) of the product as a white solid (MP = 156-158 °C). Crystals suitable for X-ray diffraction were grown by evaporation from an acetone solution of the salt. ^{1}H-NMR (500 MHz, $CDCl_3$): δ 8.26 (d, *J* = 5.5 Hz, 2H), 7.93 – 7.83 (m, 4H), 7.74 (td, *J* = 7.9, 3.6 Hz, 8H), 7.63 – 7.54 (m, 8H), 7.44 (d, *J* = 5.2 Hz, 2H). ^{11}B-NMR (128 MHz, $CDCl_3$) δ 2.85 (d, J_{F-B} = 52.3 Hz). ^{13}C{H}-NMR (126 MHz, $CDCl_3$): [missing 1 resonance] δ 147.7, 135.8 (d, J_{P-C} = 3.1 Hz), 134.4 (d, J_{P-C} = 10.4 Hz), 130.8 (d, J_{P-C} = 12.8 Hz), 127.5, 117.5 (d, J_{P-C} = 89.5 Hz). ^{19}F{H}-NMR (376 MHz, $CDCl_3$): δ -145.80 (d, J_{B-F} = 78.7 Hz). ^{31}P{H}-NMR (203 MHz, $CDCl_3$): δ 23.14. HRMS-ESI calculated for $C_{24}H_{20}P^+$ (M - $C_5H_4BF_3N^-$)$^+$ 339.1298, found 339.1302; calculated for $C_5H_4BF_3N^-$ (M - PPh_4^+)$^-$ 146.0394, found 146.0399.

S1.8. Tetrakis(3,5-dimethoxyphenyl)phosphonium trifluoro(pyridin-3-yl)borate (7).

Tetrakis(3,5-dimethoxyphenyl)phosphonium chloride (82.7 mg, 0.135 mmol, 1.05 eq.) and potassium trifluoro-3-pyridylborate (67.6 mg, 0.128 mmol, 1.00 eq.) afforded 88.2 mg (0.122 mmol, 95% yield) of the product as a pale brownish-red solid (MP > 220 °C) using the same procedure as the tetraphenylphosphonium salt **5**. Crystals suitable for X-ray diffraction were grown by evaporation from an acetone solution of the salt. ^{1}H-NMR (500 MHz, CD_2Cl_2): δ 8.58 (s, 1H), 8.26 (s, 1H), 7.79 (d, *J* = 7.3 Hz, 1H), 7.12 – 7.03 (m, 1H), 6.88 (s, 4H), 6.68 (d, *J* = 14.5 Hz, 8H), 3.80 (s, 24H). ^{11}B-NMR (128 MHz, CD_2Cl_2): δ 2.97 (q, $J_{F\text{-}B}$ = 52.4 Hz). ^{13}C{H}-NMR (126 MHz, CD_2Cl_2): [missing 1 resonance] δ 162.2 (d, $J_{P\text{-}C}$ = 19.6 Hz), 152.9, 146.5, 140.2, 123.0, 119.4 (d, $J_{P\text{-}C}$ = 90.4 Hz), 113.3 (d, $J_{P\text{-}C}$ = 11.7 Hz), 106.4, 56.6. ^{19}F{H}-NMR (376 MHz, CD_2Cl_2): δ -143.74 (dd, $J_{B\text{-}F}$ = 99.8, 46.5 Hz), -153.44. ^{31}P{H}-NMR (203 MHz, CD_2Cl_2): δ 26.66. HRMS-ESI calculated for $C_{32}H_{36}O_8P^+$ (M - $C_5H_4BF_3N^-$)$^+$ 579.2143 found 579.2138; calculated for $C_5H_4BF_3N^-$ (M - P(3,5-(MeO)$_2C_6H_3$)$_4^+$)$^-$ 146.0394, found 146.0396.

S1.9. Tetrakis(3,5-dimethoxyphenyl)phosphonium trifluoro(pyridin-4-yl)borate (8).

Tetrakis(3,5-dimethoxyphenyl)phosphonium chloride (20.2 mg, 0.033 mmol, 1.0 eq.) and potassium trifluoro(pyridin-4-yl)borate (7.1 mg, 0.038 mmol, 1.3 eq.) were reacted in the same manner as **5** to give 22.4 mg (0.031 mmol, 93% yield) of the product as a yellow solid. (MP 150-152 °C). Crystals suitable for X-ray diffraction were grown by evaporation from an acetone solution of the salt. ^{1}H-NMR (500 MHz, CD_2Cl_2): δ 8.28 (d, *J* = 4.8 Hz, 2H), 7.36 (d, *J* = 4.9 Hz, 2H), 6.87 (br. s, 4H), 6.68 (dd, *J* = 2.2 Hz, 14.5 Hz, 8H), 3.80 (s, 24 H). ^{11}B-NMR (160 MHz, CD_2Cl_2): δ 2.67 (q, $J_{B\text{-}F}$ = 52.4 Hz), ^{13}C{H}-NMR (126 MHz, CD_2Cl_2): [missing 1 resonance]: δ 162.6 (d, $J_{P\text{-}C}$ = 19.6 Hz), 148.1, 127.5 (d, *J* = 2.1 Hz), 119.6, 118.9, 113.1 (d, $J_{P\text{-}C}$ = 11.7 Hz), 106.3 (d, *J* = 2.6 Hz), 56.4. ^{19}F{H}-NMR (471 MHz, CD_2Cl_2): δ -145.30 (dd, $J_{F\text{-}B}$ = 44.9 Hz, 99.5 Hz). ^{31}P{H}-NMR (202 MHz, CD_2Cl_2): δ 26.66. HRMS-ESI calculated for $C_{32}H_{36}O_8P^+$ (M - $C_5H_4BF_3N^-$)$^+$ 579.2143 found 579.2127; calculated for $C_5H_4BF_3N^-$ (M - P(3,5-(MeO)$_2C_6H_3$)$_4^+$)$^-$ 146.0394, found 146.0387.

S1.10. Tetrakis[4-(dimethylamino)phenyl]phosphonium trifluoro(pyridin-3-yl)borate (9).

Tetrakis(4-(dimethylamino)phenyl)phosphonium chloride (79.2 mg, 0.145 mmol, 1.05 eq.) and potassium trifluoro-3-pyridylborate (72.7 mg, 0.138 mmol, 1.00 eq.) afforded the product in 97% yield (88.1 mg, 0.133 mmol) as a pale-yellow solid (MP = 161-163 °C) using the same procedure as the tetraphenylphosphonium salt **5**. Crystals suitable for X-ray diffraction were grown by evaporation from an acetone solution of the salt. ^{1}H-NMR (500 MHz, acetone-d$_6$): δ 8.68 (s, 1H), 8.19 (s, 1H), 7.78 – 7.66 (m, 1H), 7.45 – 7.30 (m, 8H), 7.03 – 6.87 (m, 9H), 3.11 (s, 24H). ^{11}B-NMR (128 MHz, acetone-d$_6$): δ 2.98 (q, $J_{B\text{-}F}$ = 51.0 Hz). ^{13}C{H}-NMR (126 MHz, acetone-d$_6$): [missing 1 resonance] δ 154.6, 154.4, 146.8, 139.6, 135.9 (d, $J_{P\text{-}C}$ = 11.6 Hz), 122.7, 113.0 (d, $J_{P\text{-}C}$ = 13.2 Hz), 104.7 (d, $J_{P\text{-}C}$ = 103.4 Hz), 40.0. ^{19}F{H}-NMR (471 MHz, acetone-d$_6$): δ -142.86 (dd, $J_{F\text{-}B}$ = 101.1, 50.2 Hz). ^{31}P{H}-

NMR (203 MHz, $CDCl_3$): δ 18.76. HRMS-ESI calculated for $C_{32}H_{40}NP^+$ (M - $C_5H_4BF_3N^-$)$^+$ 511.2986, found 511.2982; calculated for $C_5H_4BF_3N^-$ (M - P(4-$Me_2NC_6H_4)_4^+$)$^-$ 146.0394, found 146.0402.

S1.11. Tetrakis[4-(dimethylamino)phenyl]phosphonium trifluoro(pyridin-4-yl)borate (10).

Tetrakis[4-(dimethylamino)phenyl]phosphonium (74.1 mg, 0.11 mmol, 1 eq.) and potassium trifluoro-4-pyridylborate (31.4 mg, 0.17 mmol, 1.5 eq.) were reacted using the same procedure as **5** to give 67.6 mg of **10** (0.10 mmol, 90% yield) as a white solid. (MP > 200 °C). Crystals suitable for X-ray diffraction were grown by evaporation from either DCM (yielding the neat form) or acetone (yielding the hemihydrate). ^{1}H-NMR (500 MHz, CD_2Cl_2): δ 8.29 (d, J = 4.8 Hz, 2H), 7.38 (d, J = 4.7 Hz, 2H), 7.29, (dd, J = 9.0 Hz, 47.4 Hz, 8H), 6.79 (dd, J = 2.6 Hz, 9.2 Hz, 8H), 3.06 (s, 24 H). ^{11}B-NMR (160 MHz, CD_2Cl_2): δ 2.69 (q, $J_{B\text{-}F}$ = 52.0 Hz). ^{13}C{H}-NMR (126 MHz, CD_2Cl_2): [missing 1 resonance] δ 153.9 (d, J = 2.3 Hz), 148.1, 135.4 (d, $J_{P\text{-}C}$ = 11.7 Hz), 127.6 (d, J = 2.1 Hz), 112.3 (d, $J_{P\text{-}C}$ = 13.6 Hz), 104.1 (d, $J_{P\text{-}C}$ = 103.4 Hz), 40.1. ^{19}F{H}-NMR (471 MHz, CD_2Cl_2): δ -145.37 (dd, $J_{F\text{-}B}$ = 46.1 Hz, 99.9 Hz). ^{31}P{H}-NMR (203 MHz, CD_2Cl_2): δ 18.73. HRMS-ESI calculated for $C_{32}H_{40}NP^+$ (M - $C_5H_4BF_3N^-$)$^+$ 511.2986, found 511.2963; calculated for $C_5H_4BF_3N^-$ (M - P(4-$Me_2NC_6H_4)_4^+$)$^-$ 146.0394, found 146.0390.

S2. NMR Spectra

Potassium trifluoro(pyridin-3-yl)borate (**1**) (^{1}H, CD_3OD, 500 MHz)

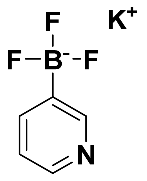

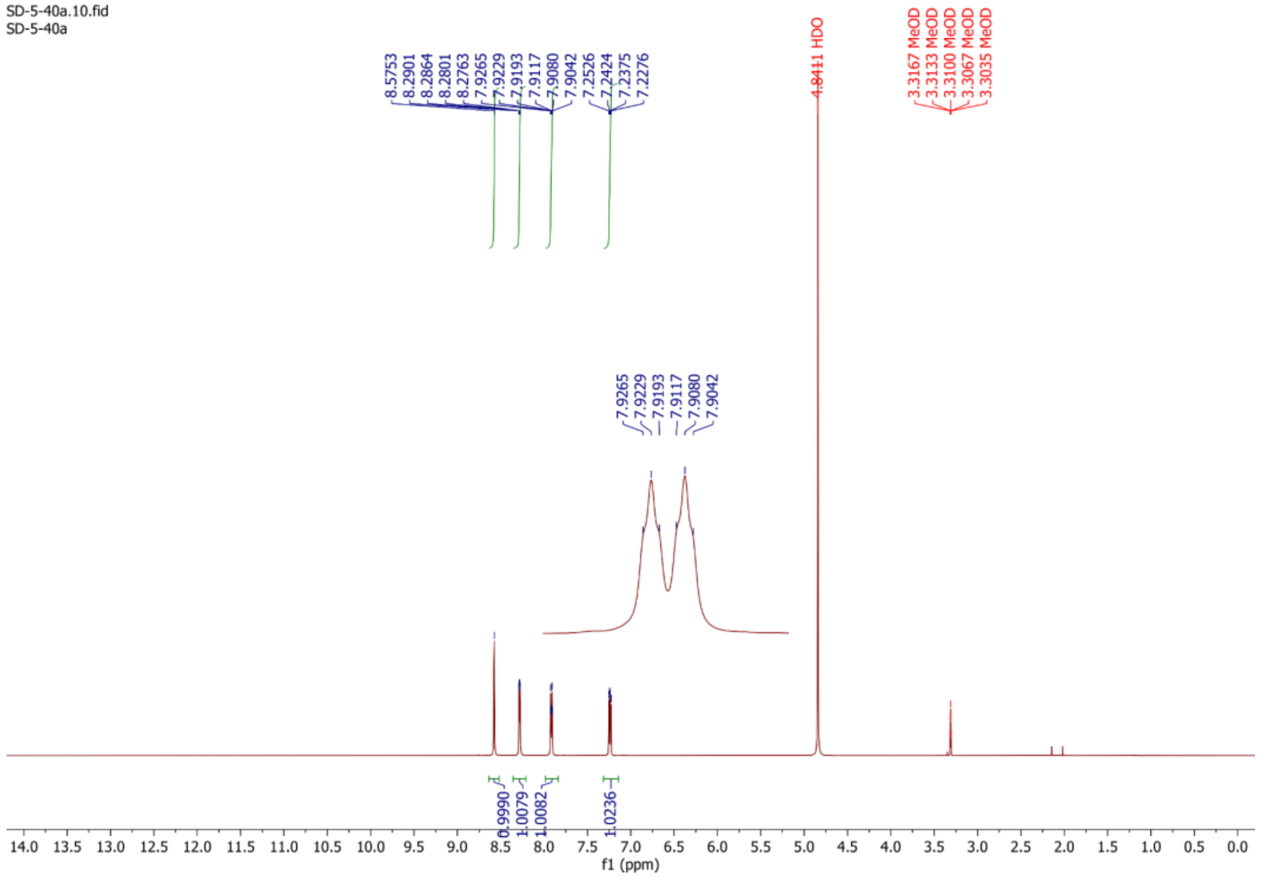

Potassium trifluoro(pyridin-3-yl)borate (**1**) (^{11}B, CD_3OD, 128 MHz)

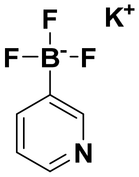

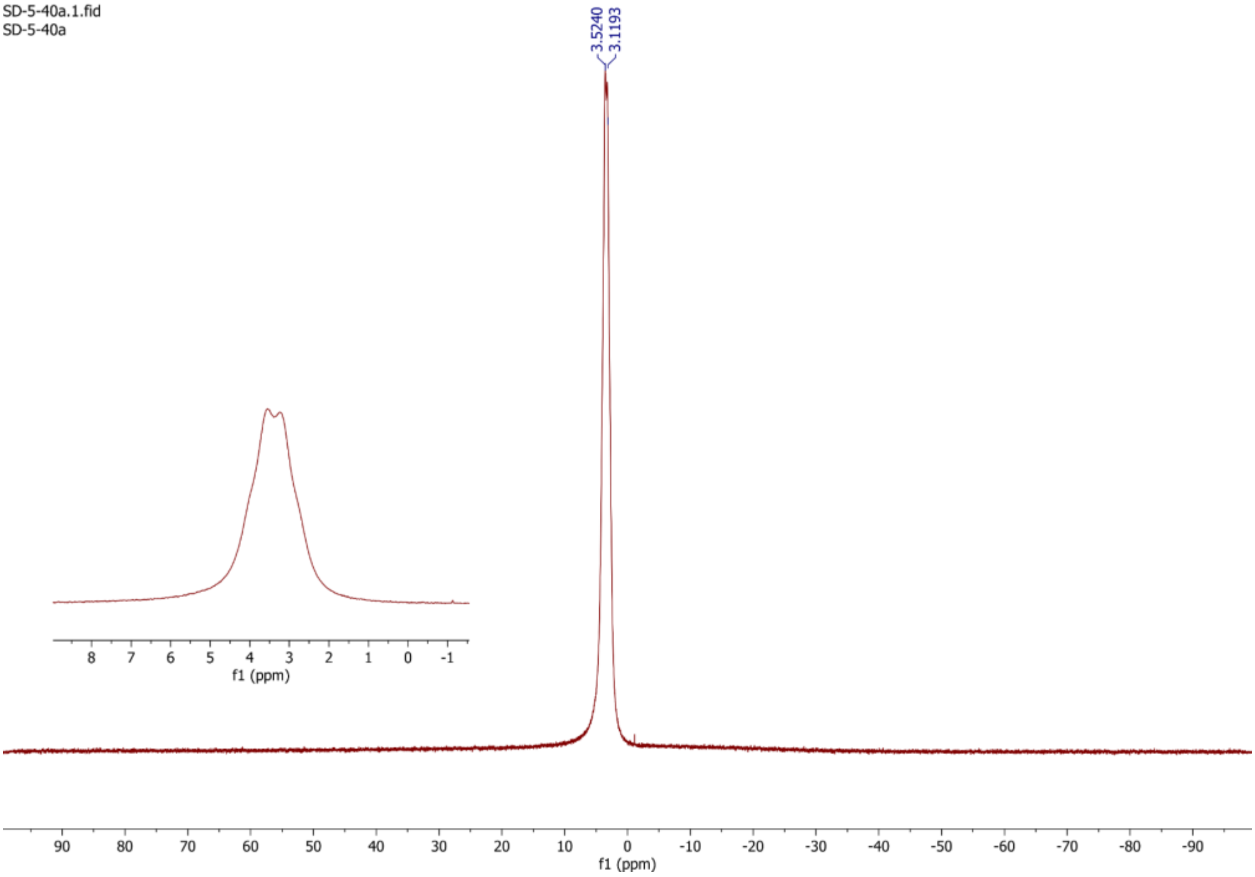

Potassium trifluoro(pyridin-3-yl)borate (**1**) ($^{13}C\{H\}$, CD_3OD, 126 MHz)

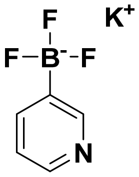

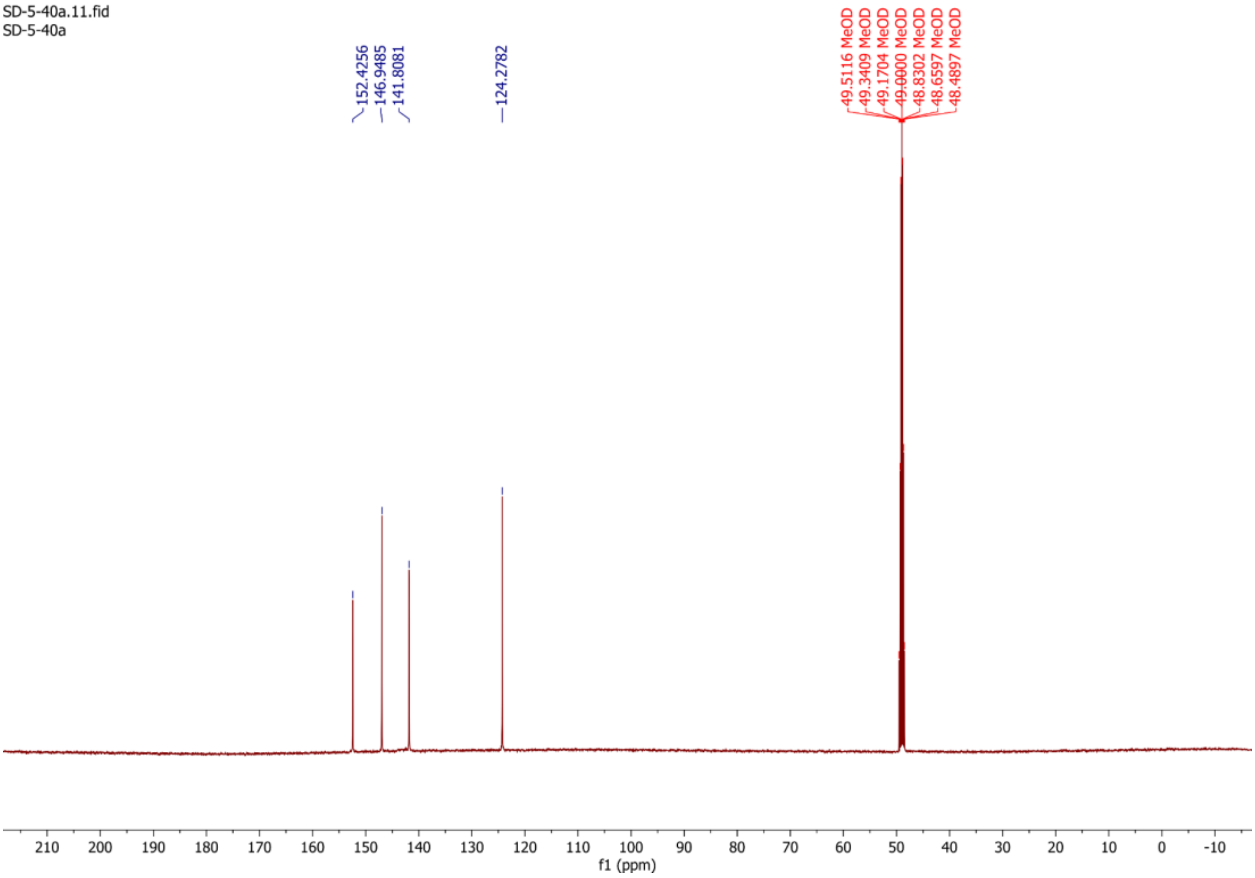

Potassium trifluoro(pyridin-3-yl)borate (**1**) ($^{19}F\{H\}$, CD_3OD, 471 MHz)

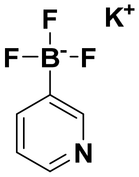

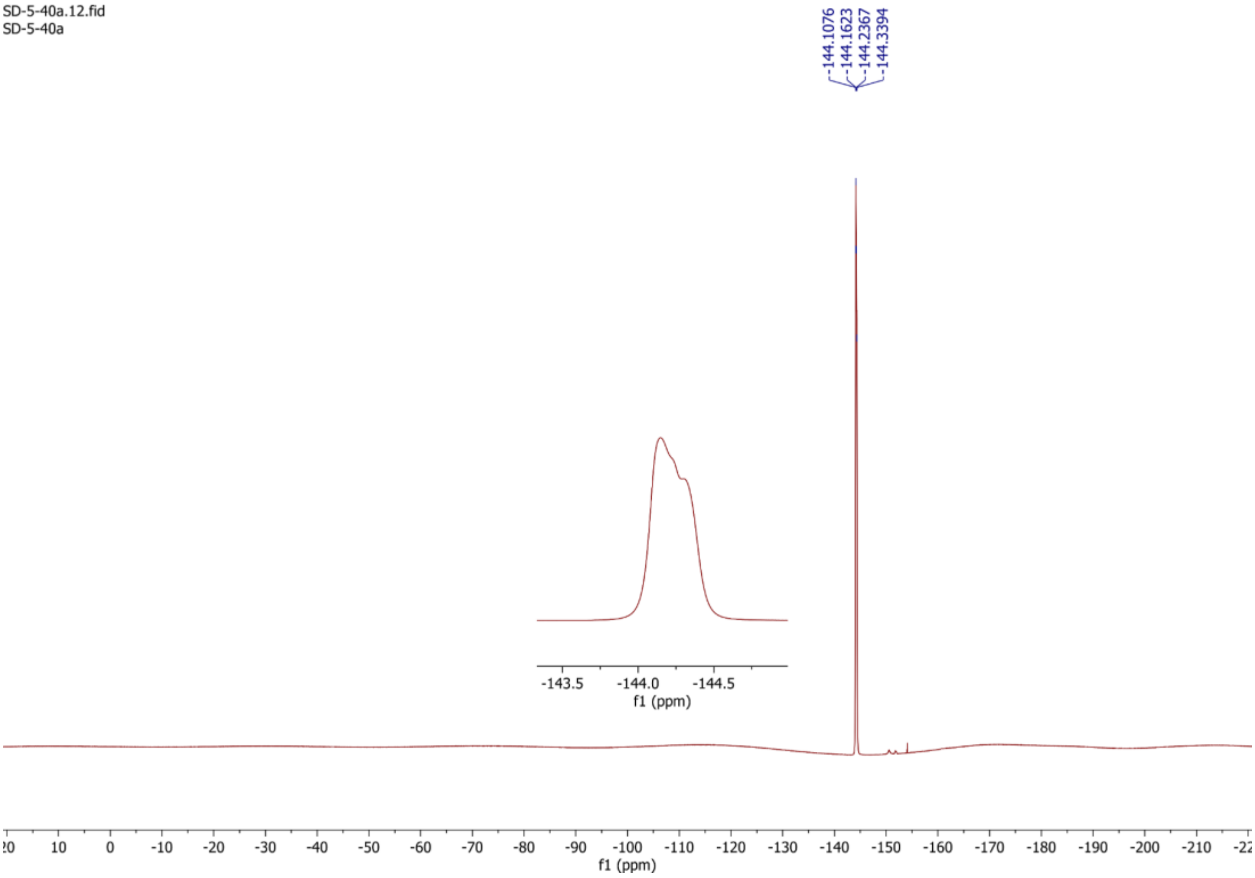

Potassium trifluoro(pyridin-4-yl)borate (**2**) (^{1}H, $(CD_3)_2CO$, 500 MHz)

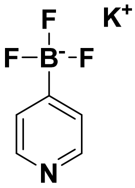

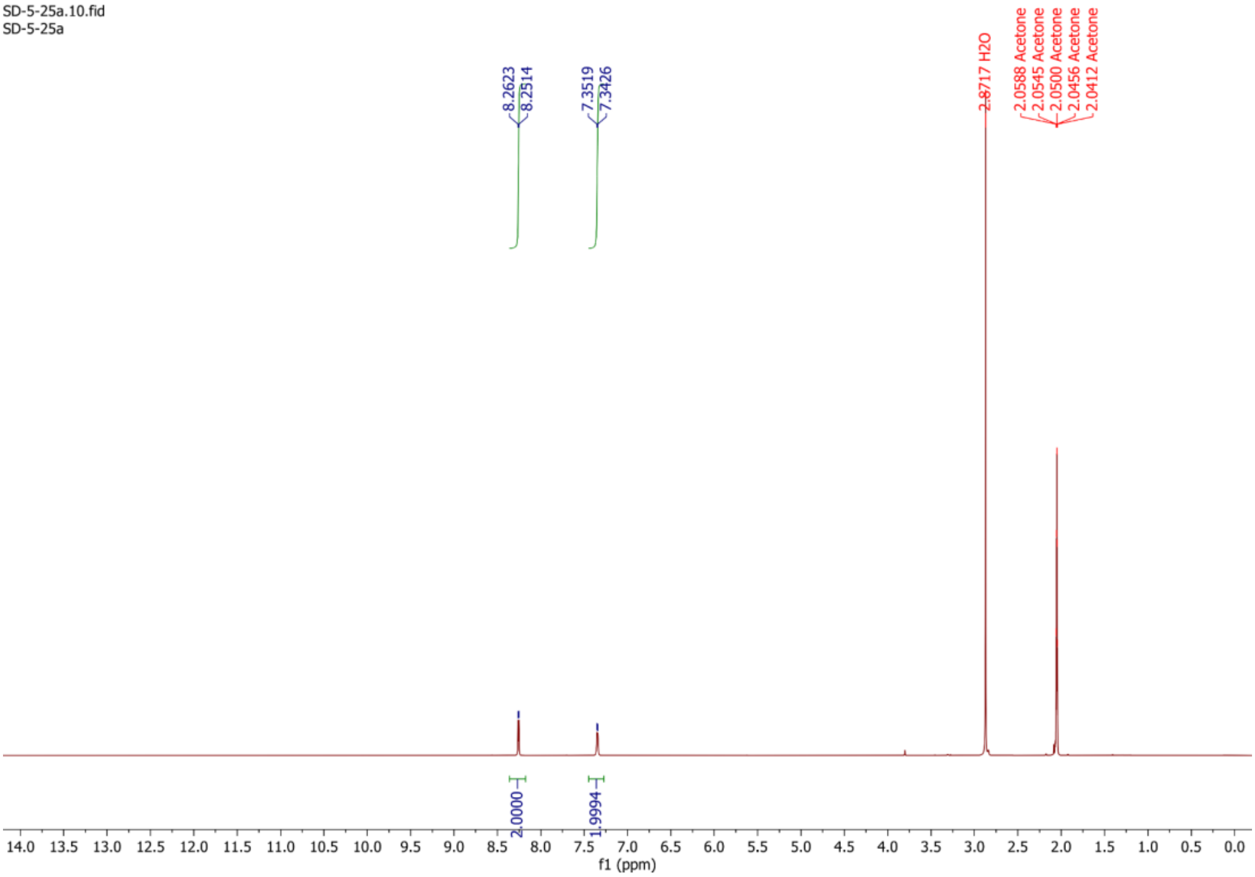

Potassium trifluoro(pyridin-4-yl)borate (**2**) (^{11}B, CD_3OD, 161 MHz) (not background suppressed)

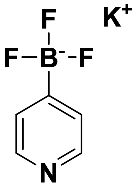

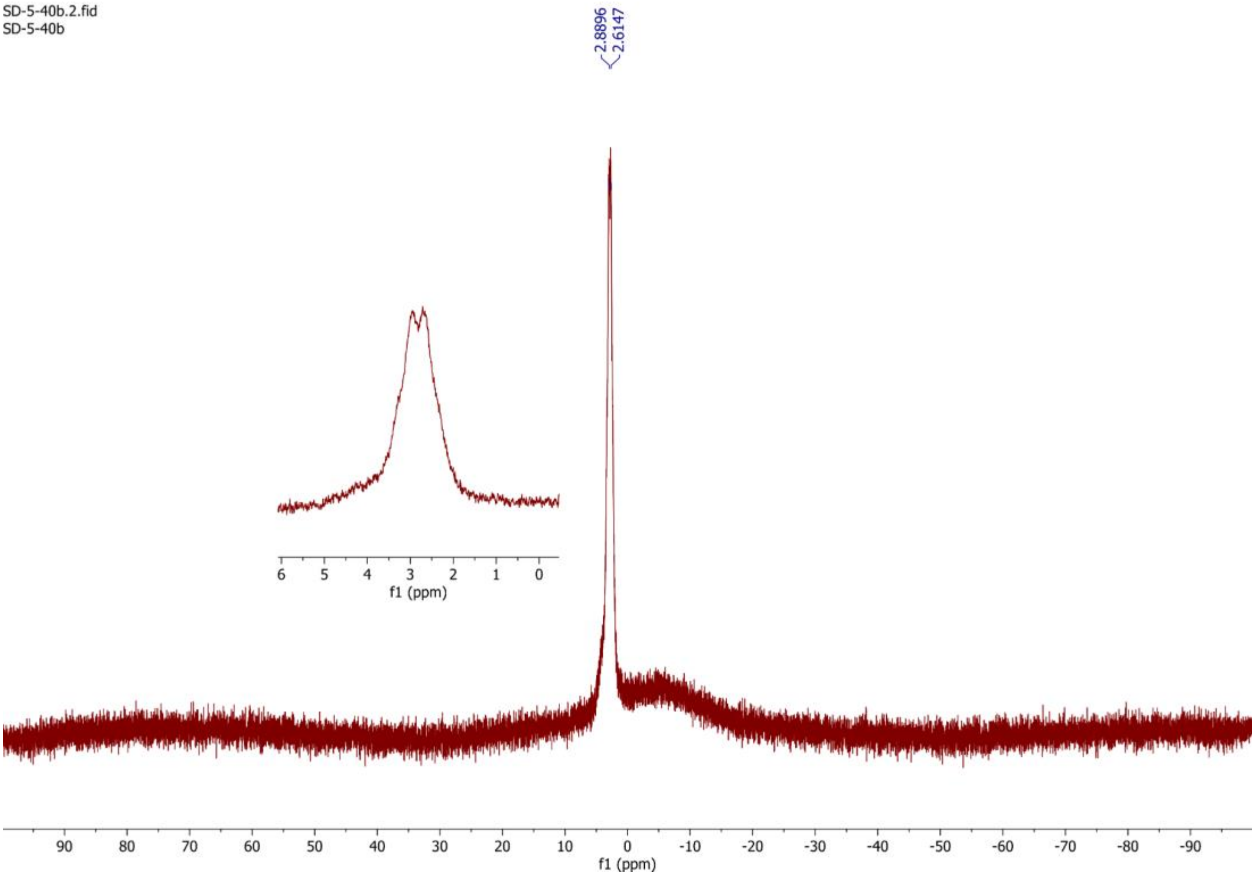

Potassium trifluoro(pyridin-4-yl)borate (**2**) ($^{13}C\{H\}$, CD_3OD, 126 MHz)

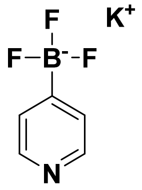

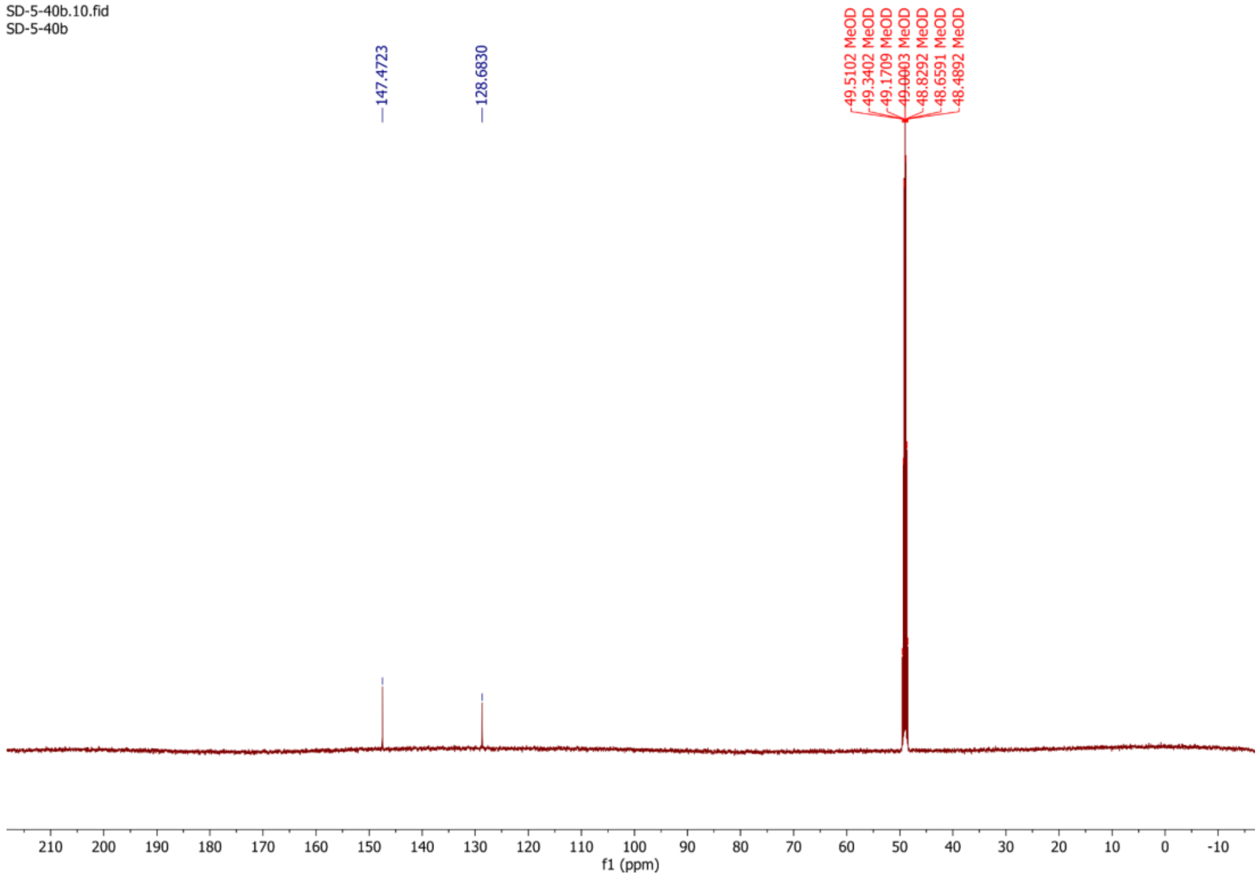

Potassium trifluoro(pyridin-4-yl)borate (**2**) (^{19}F{H}, $(CD_3)_2CO$, 470 MHz)

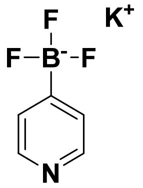

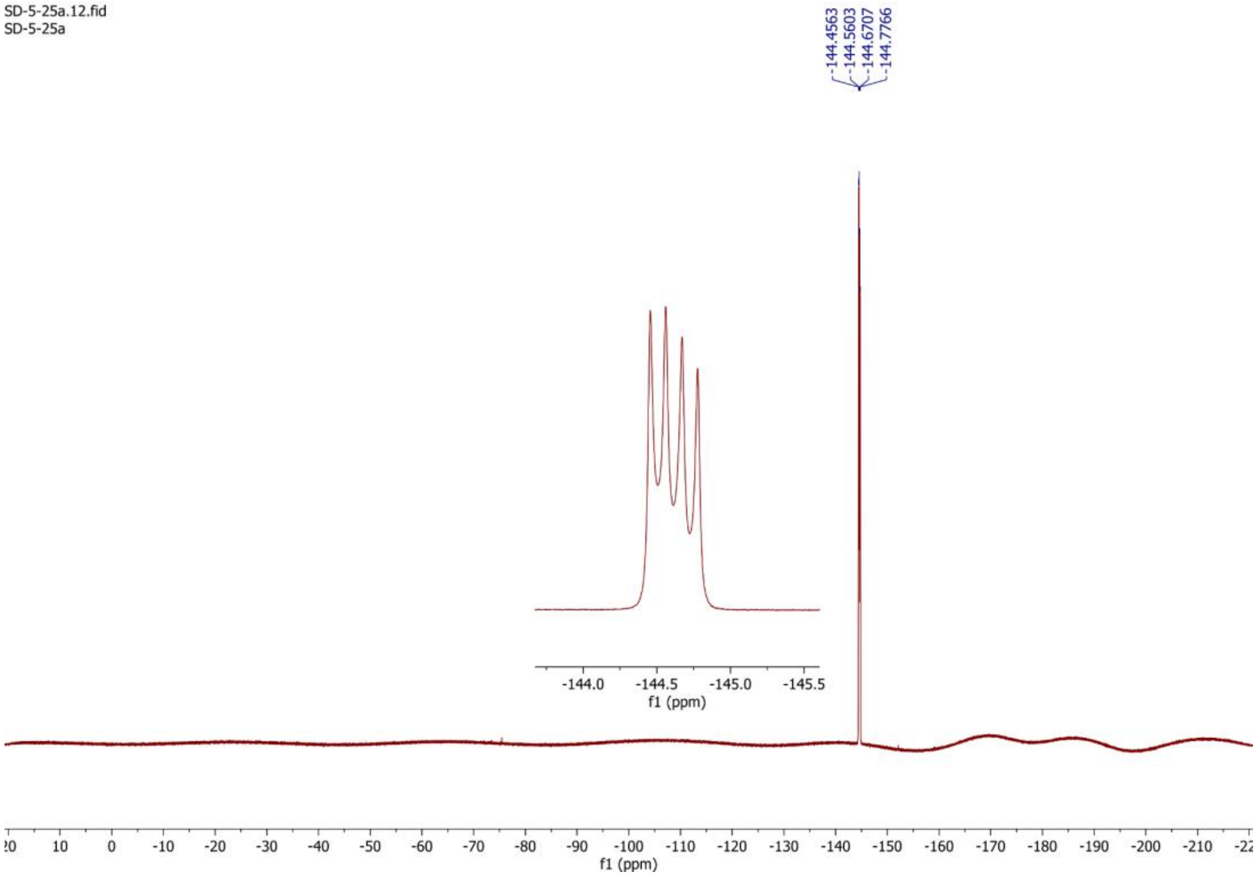

Tetrabutylammonium trifluoro(pyridin-3-yl)borate (**3**) (^{1}H, $CDCl_3$, 500 MHz)

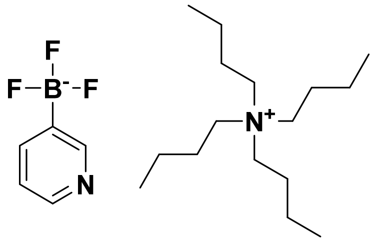

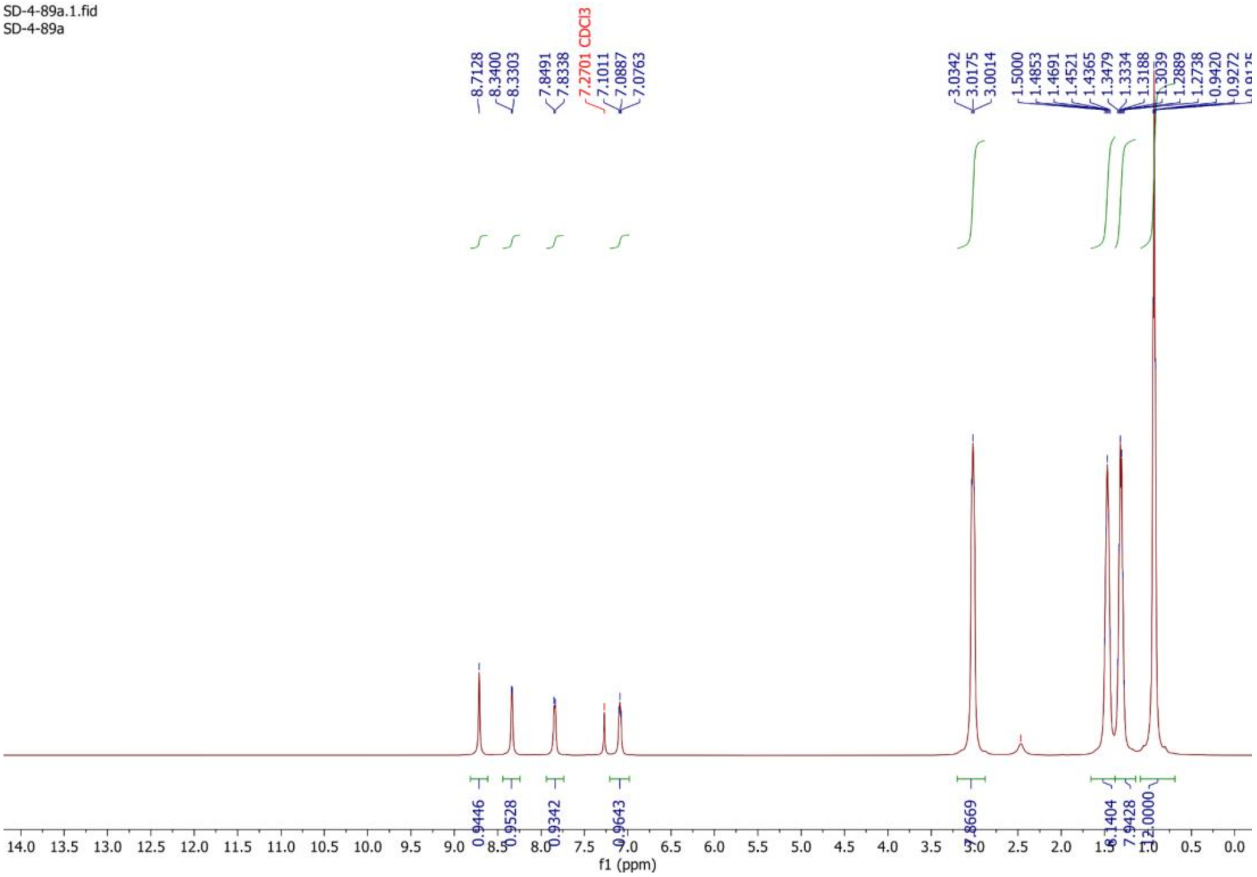

Tetrabutylammonium trifluoro(pyridin-3-yl)borate (**3**) (^{11}B, $CDCl_3$, 161 MHz) (not background suppressed)

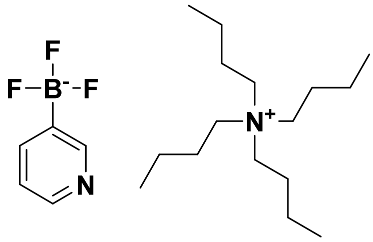

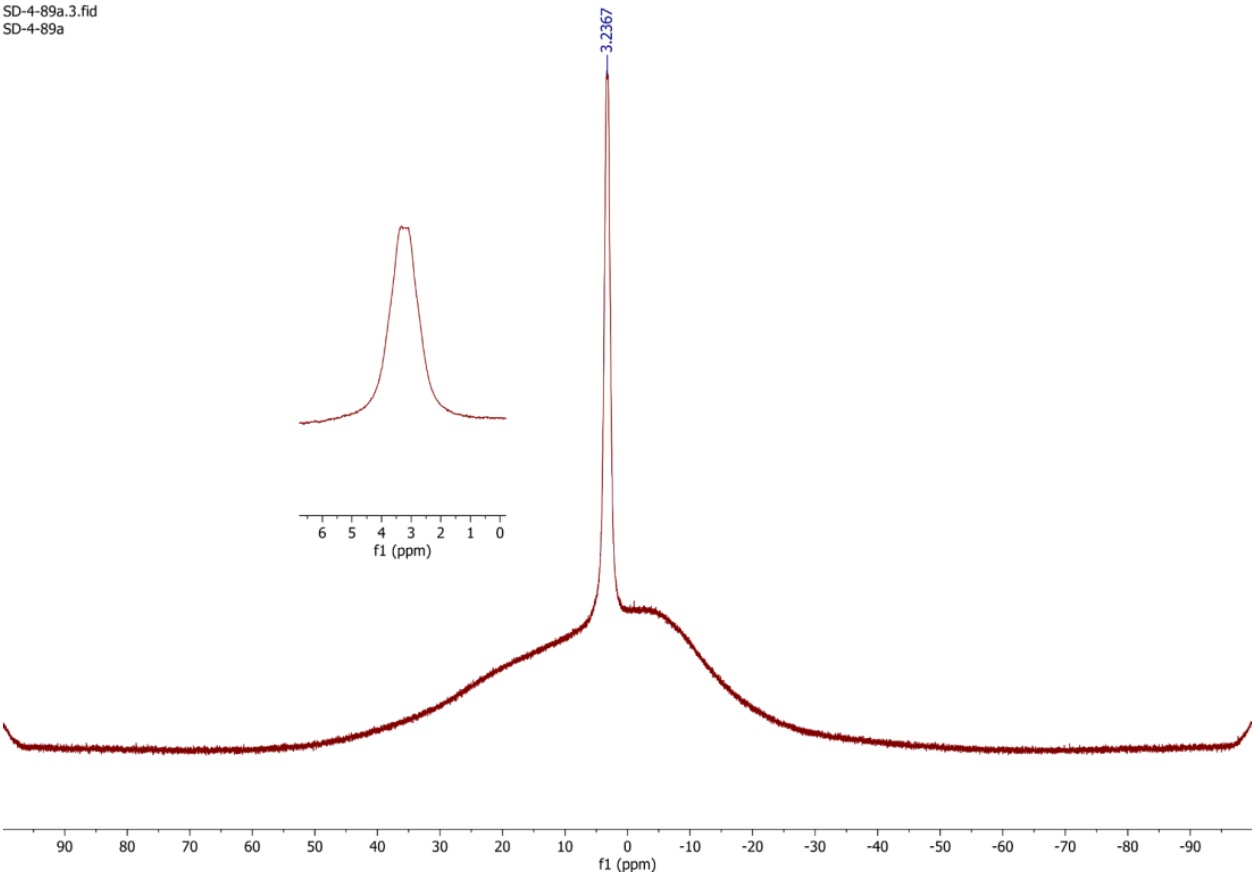

Tetrabutylammonium trifluoro(pyridin-3-yl)borate (**3**) ($^{13}C\{H\}$, $CDCl_3$, 126 MHz)

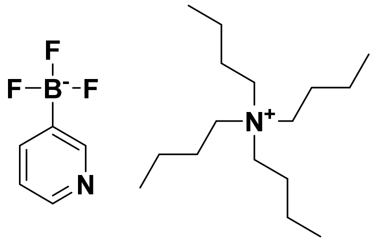

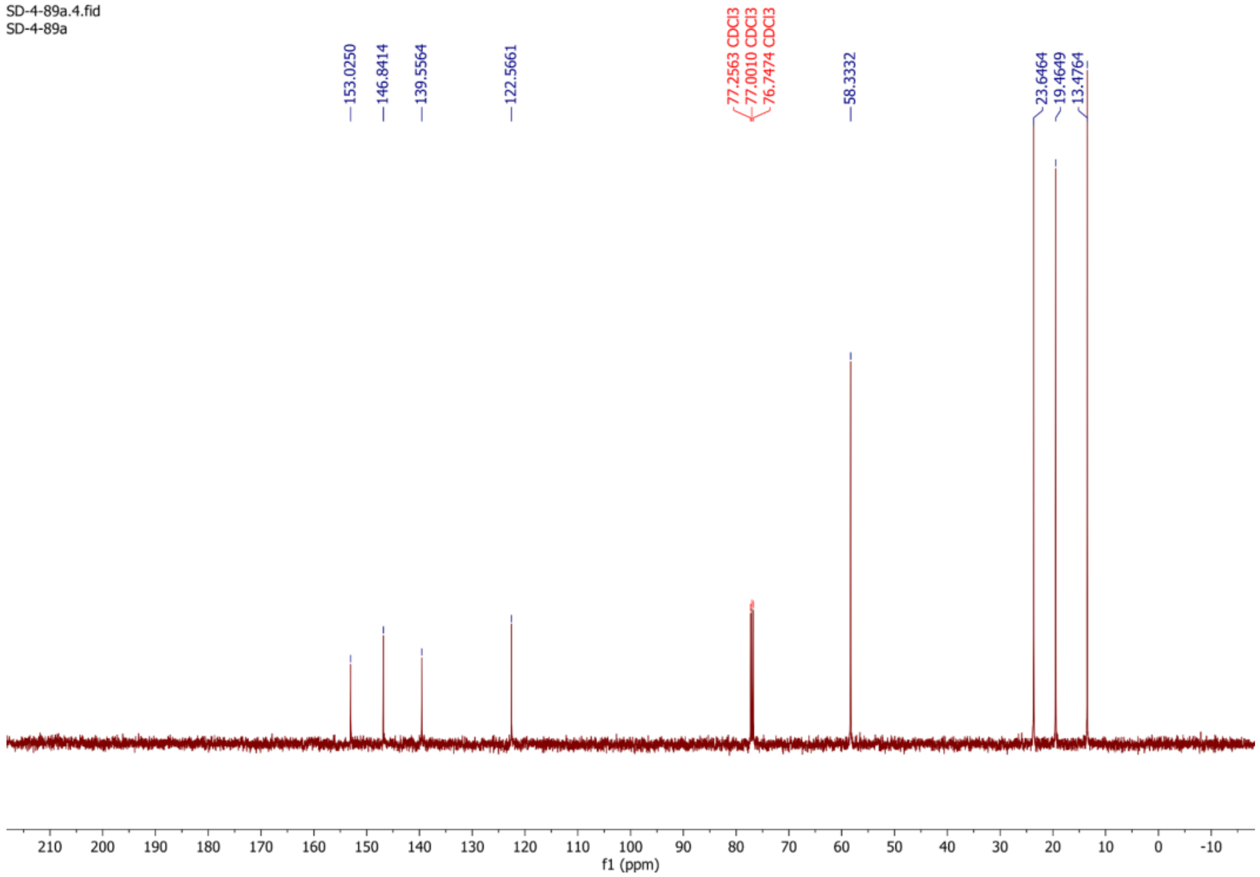

Tetrabutylammonium trifluoro(pyridin-3-yl)borate (**3**) ($^{19}F\{H\}$, $CDCl_3$, 471 MHz)

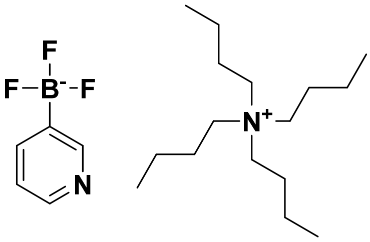

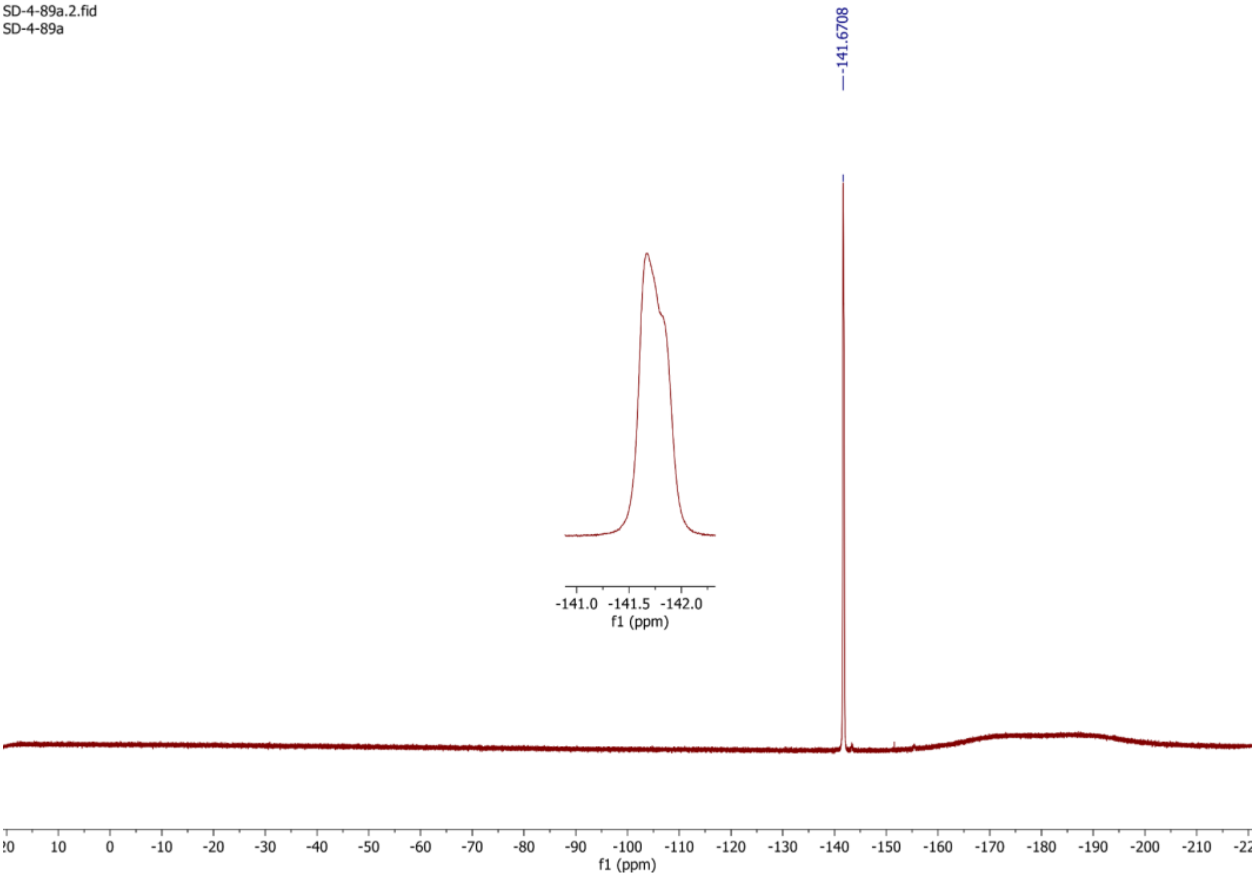

Tetrabutylammonium trifluoro(pyridin-4-yl)borate (**4**) (^{1}H, CD_2Cl_2, 400 MHz)

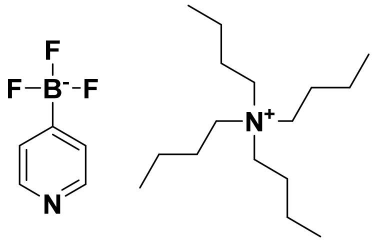

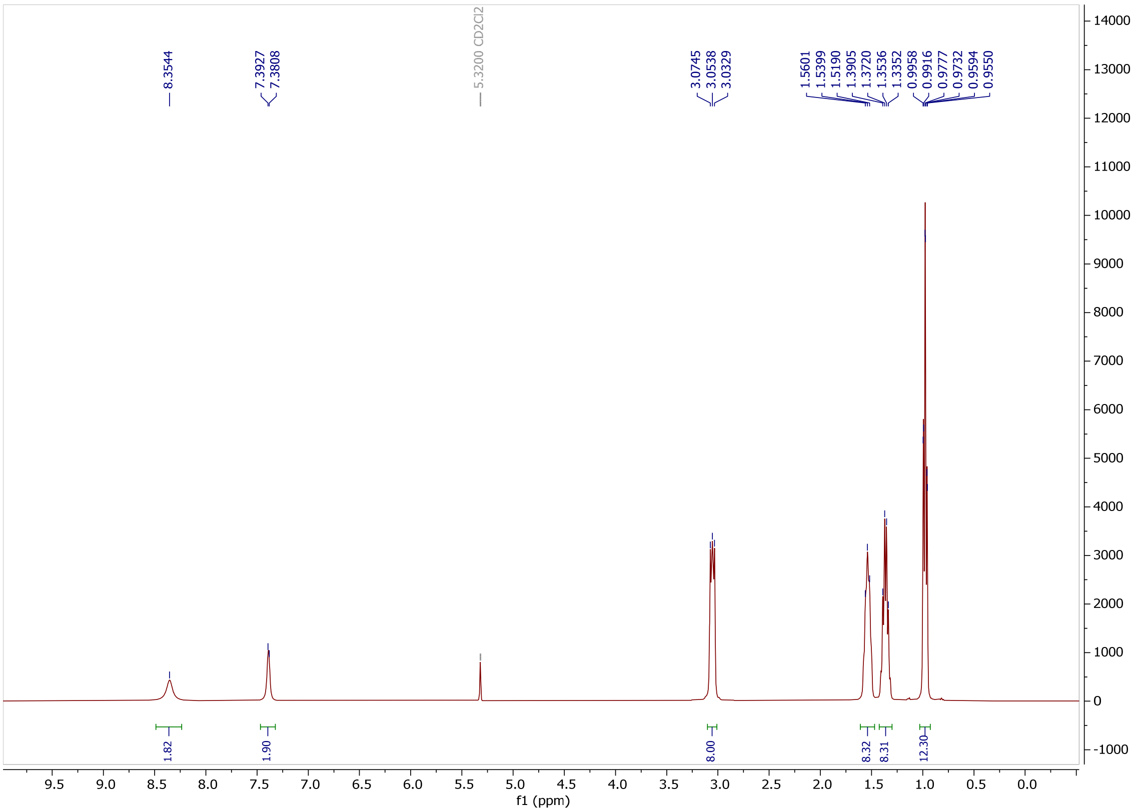

Tetrabutylammonium trifluoro(pyridin-4-yl)borate (**4**) (^{11}B, CD_2Cl_2, 128 MHz) (not background suppressed)

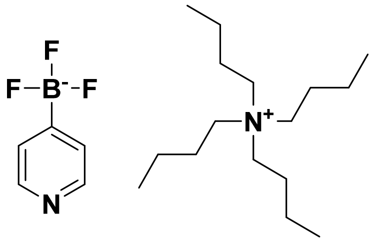

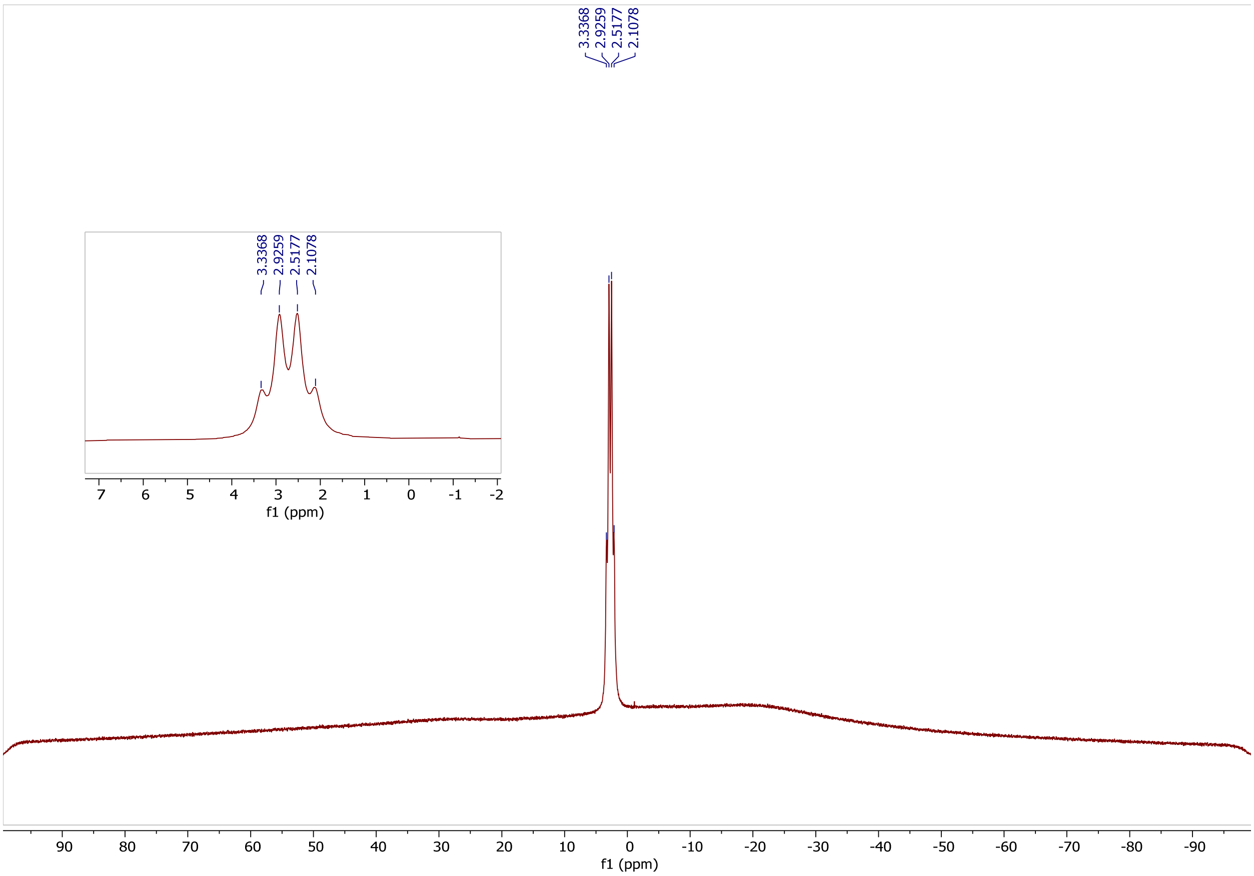

Tetrabutylammonium trifluoro(pyridin-4-yl)borate (**4**) ($^{13}C\{H\}$, CD_2Cl_2, 101 MHz)

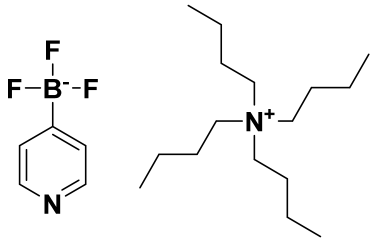

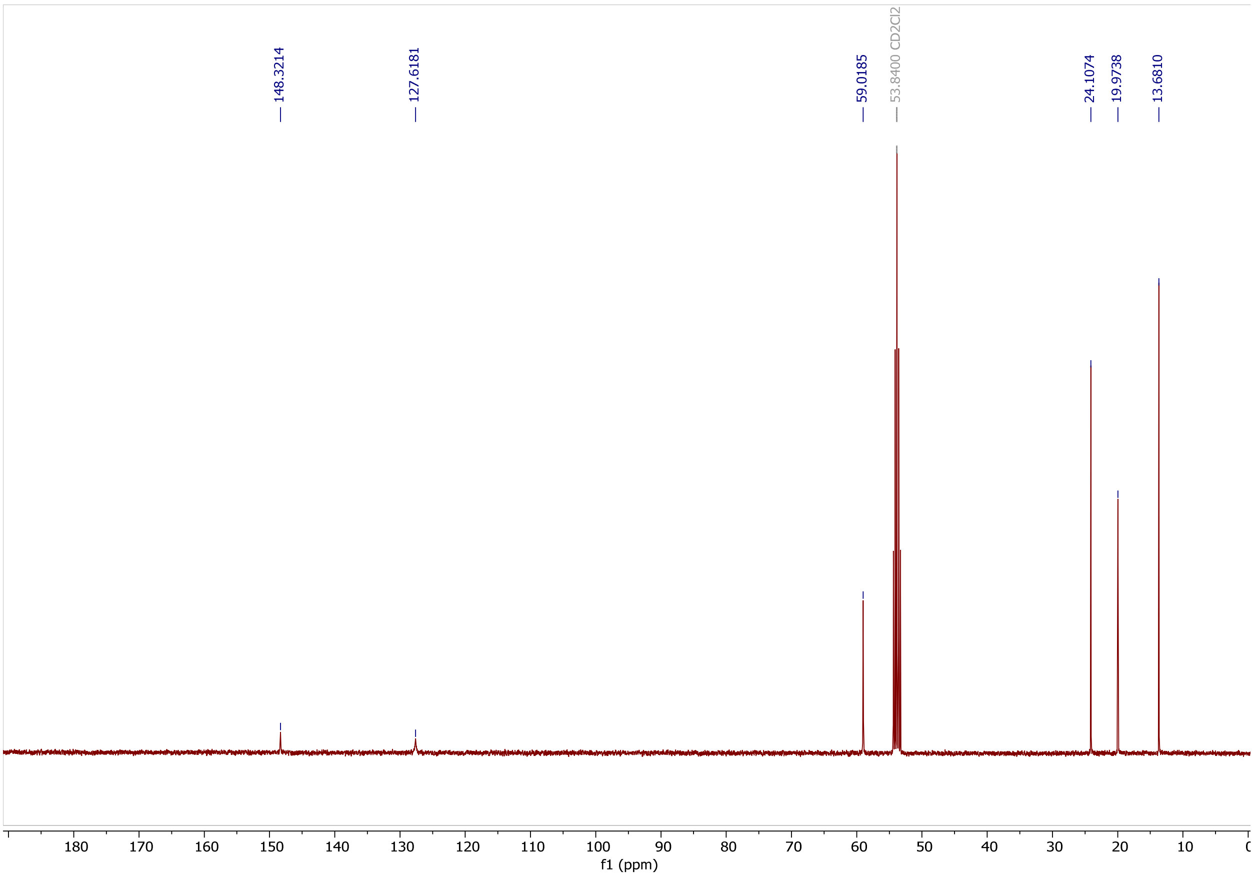

Tetrabutylammonium trifluoro(pyridin-4-yl)borate (**4**) ($^{19}F\{H\}$, CD_2Cl_2, 376 MHz)

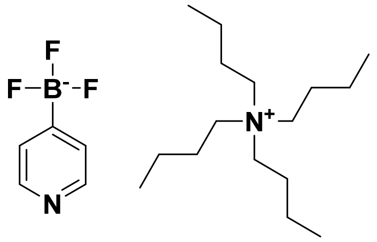

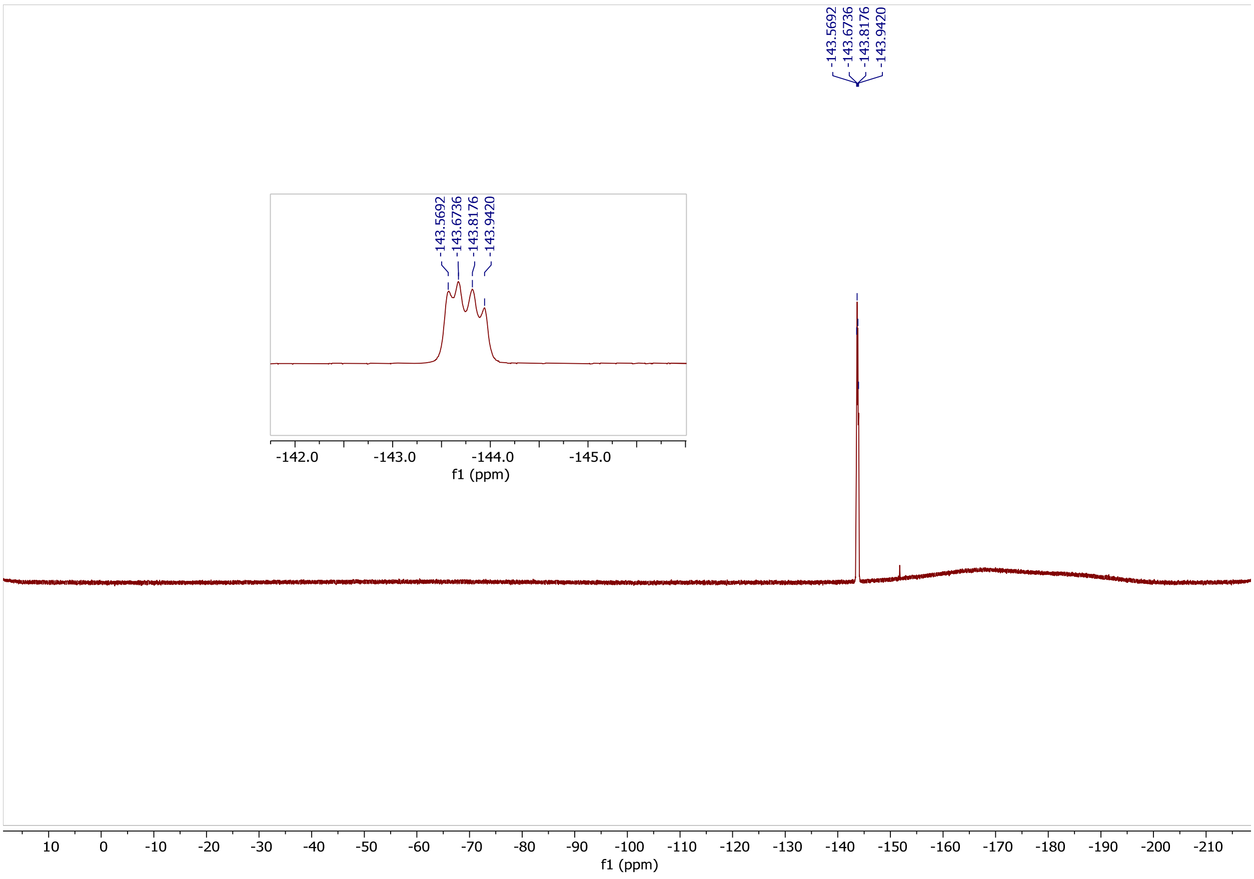

Tetraphenylphosphonium trifluoro(pyridin-3-yl)borate (**5**) (^{1}H, $CDCl_3$, 500 MHz)

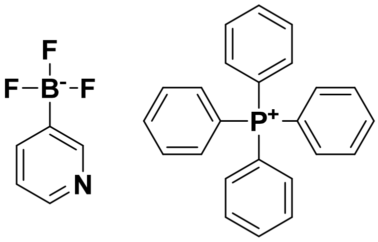

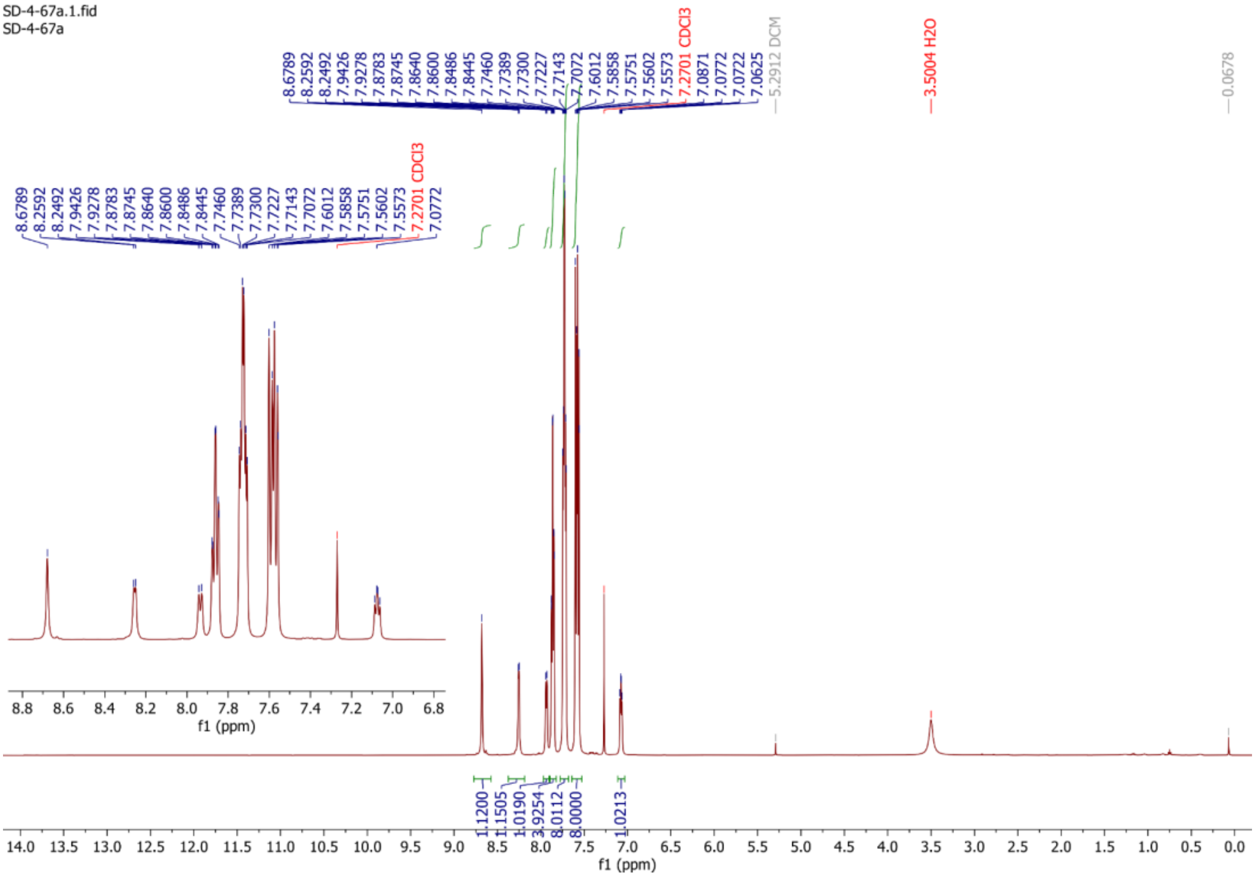

Tetraphenylphosphonium trifluoro(pyridin-3-yl)borate (**5**) (^{11}B, $CDCl_3$, 161 MHz) (not background suppressed)

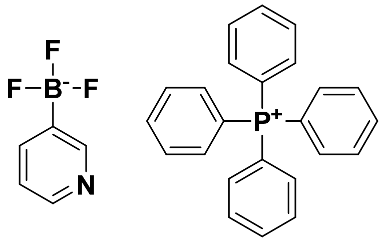

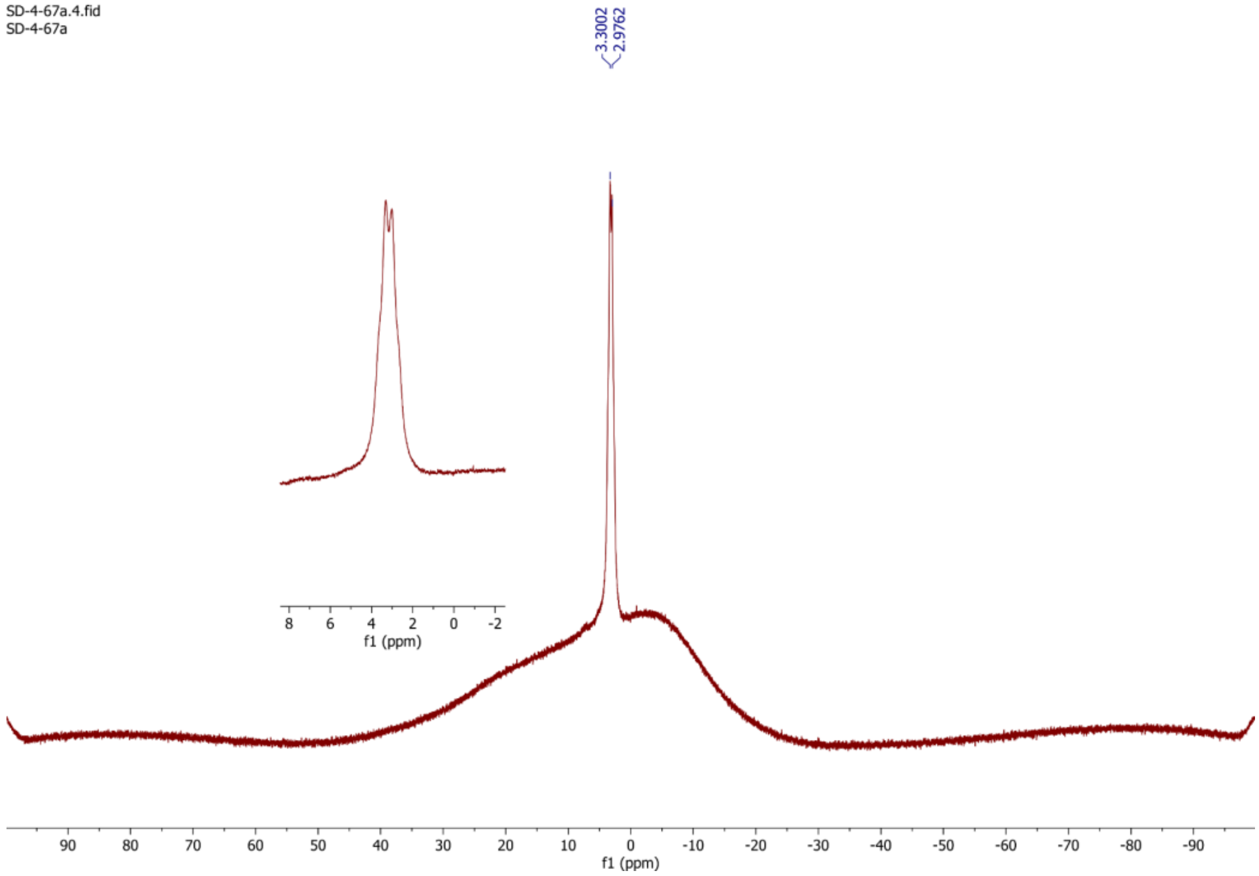

Tetraphenylphosphonium trifluoro(pyridin-3-yl)borate (**5**) ($^{13}C\{H\}$, $CDCl_3$, 126 MHz)

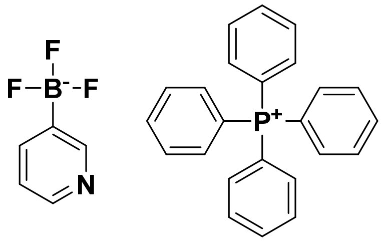

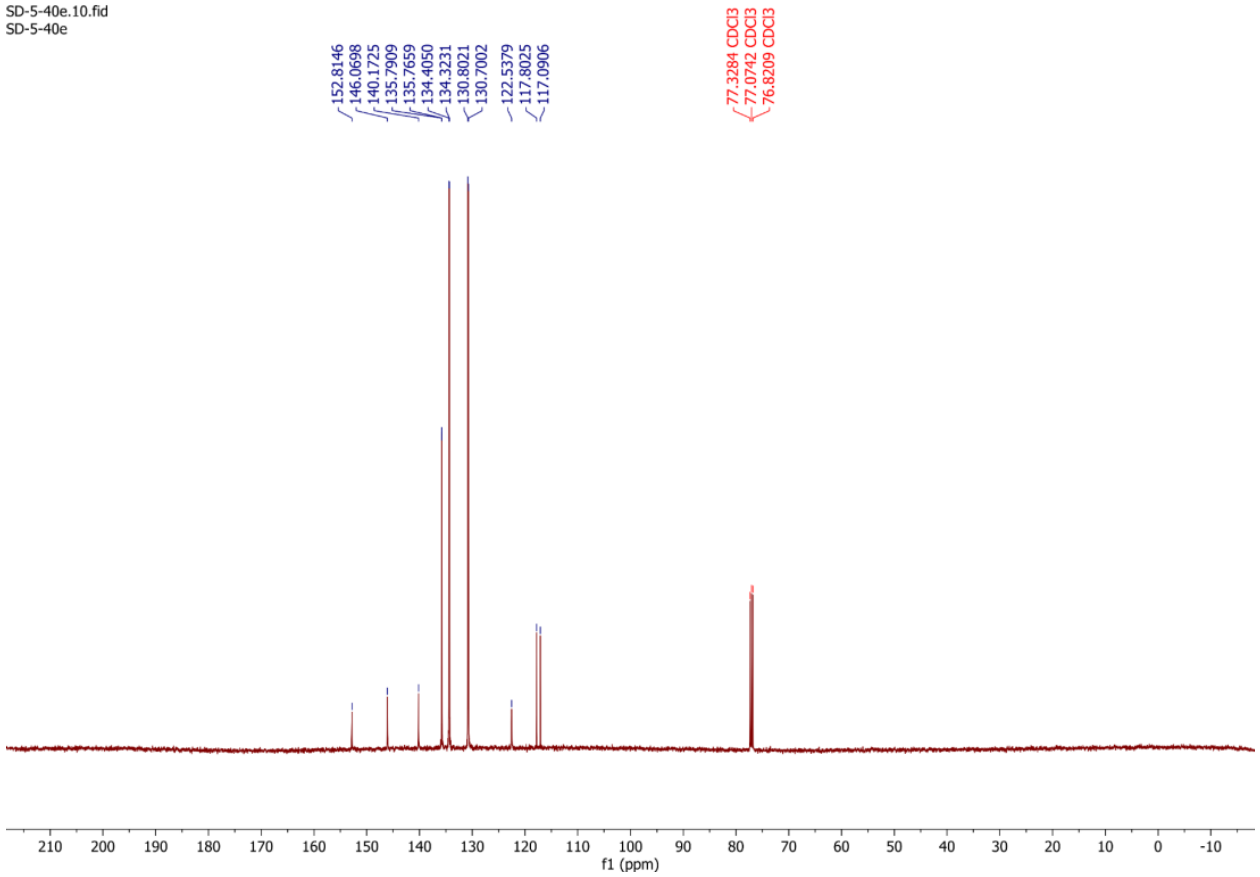

Tetraphenylphosphonium trifluoro(pyridin-3-yl)borate (**5**) (^{19}F{H}, $CDCl_3$, 471 MHz)

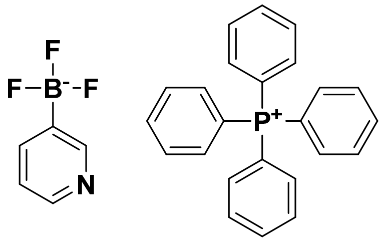

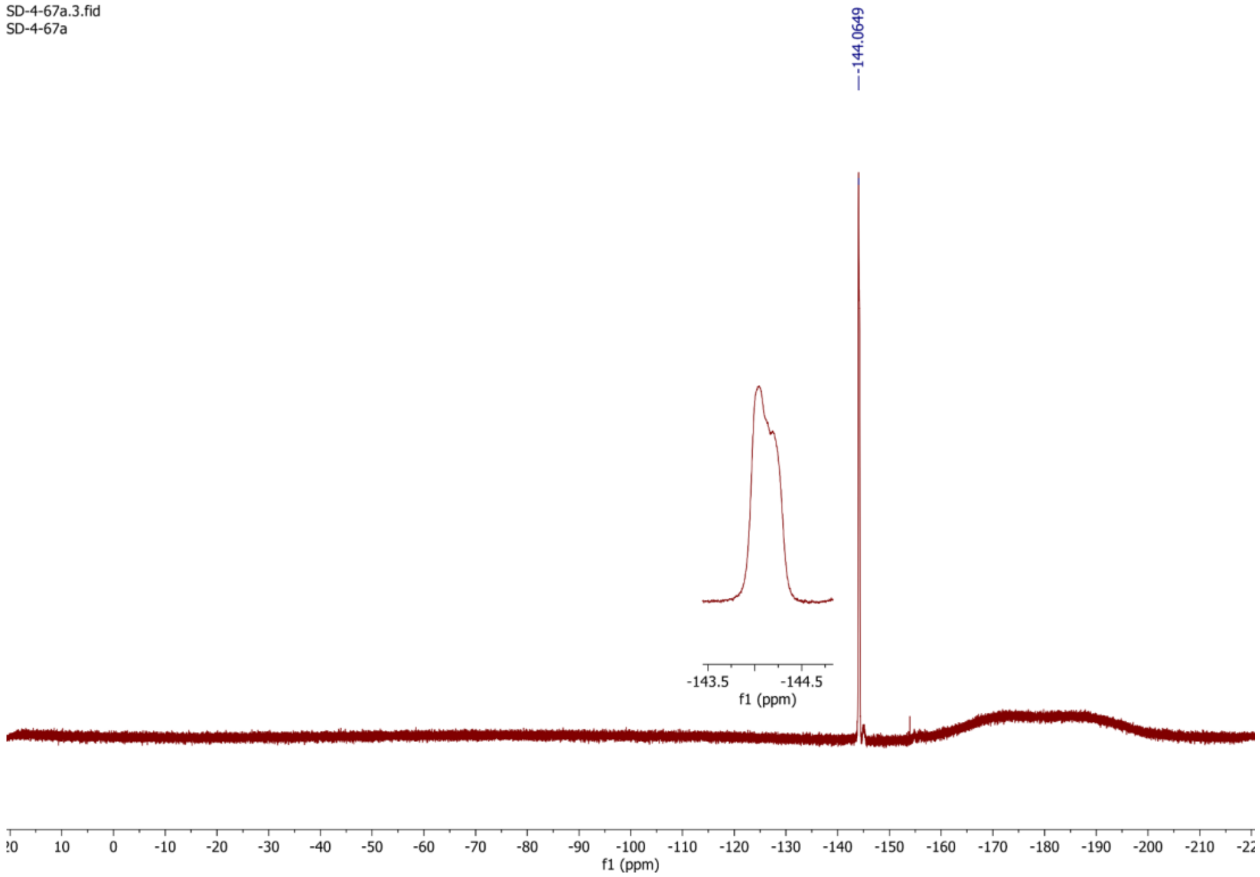

Tetraphenylphosphonium trifluoro(pyridin-3-yl)borate (**5**) (^{31}P{H}, $CDCl_3$, 203 MHz)

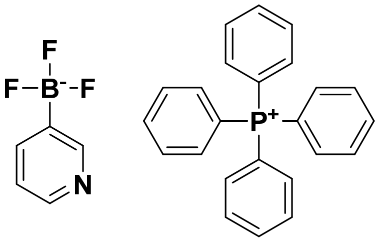

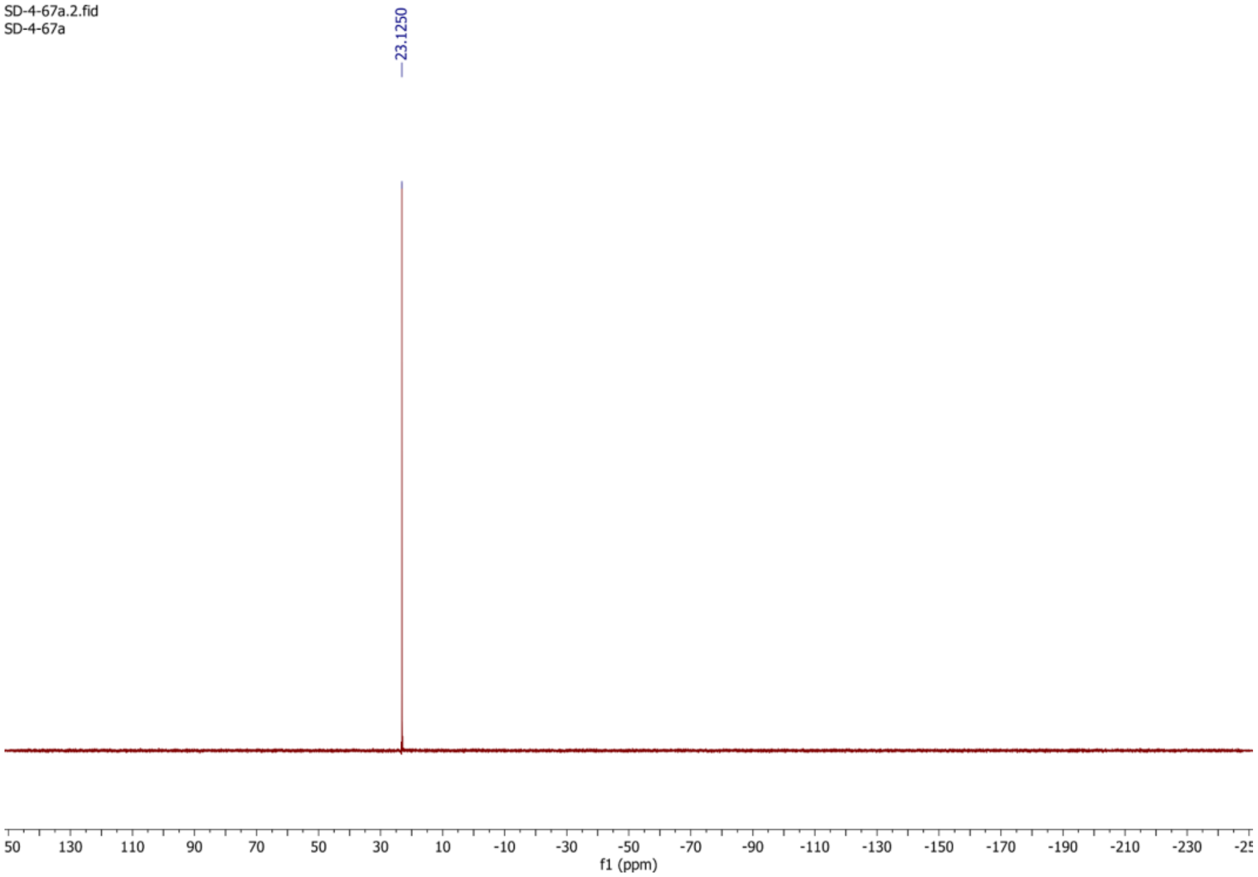

Tetraphenylphosphonium trifluoro(pyridin-4-yl)borate (**6**) (^{1}H, $CDCl_3$, 500 MHz)

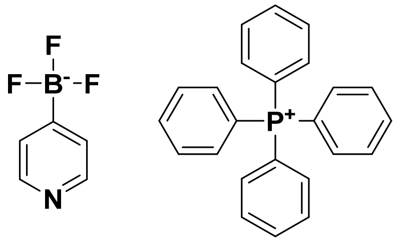

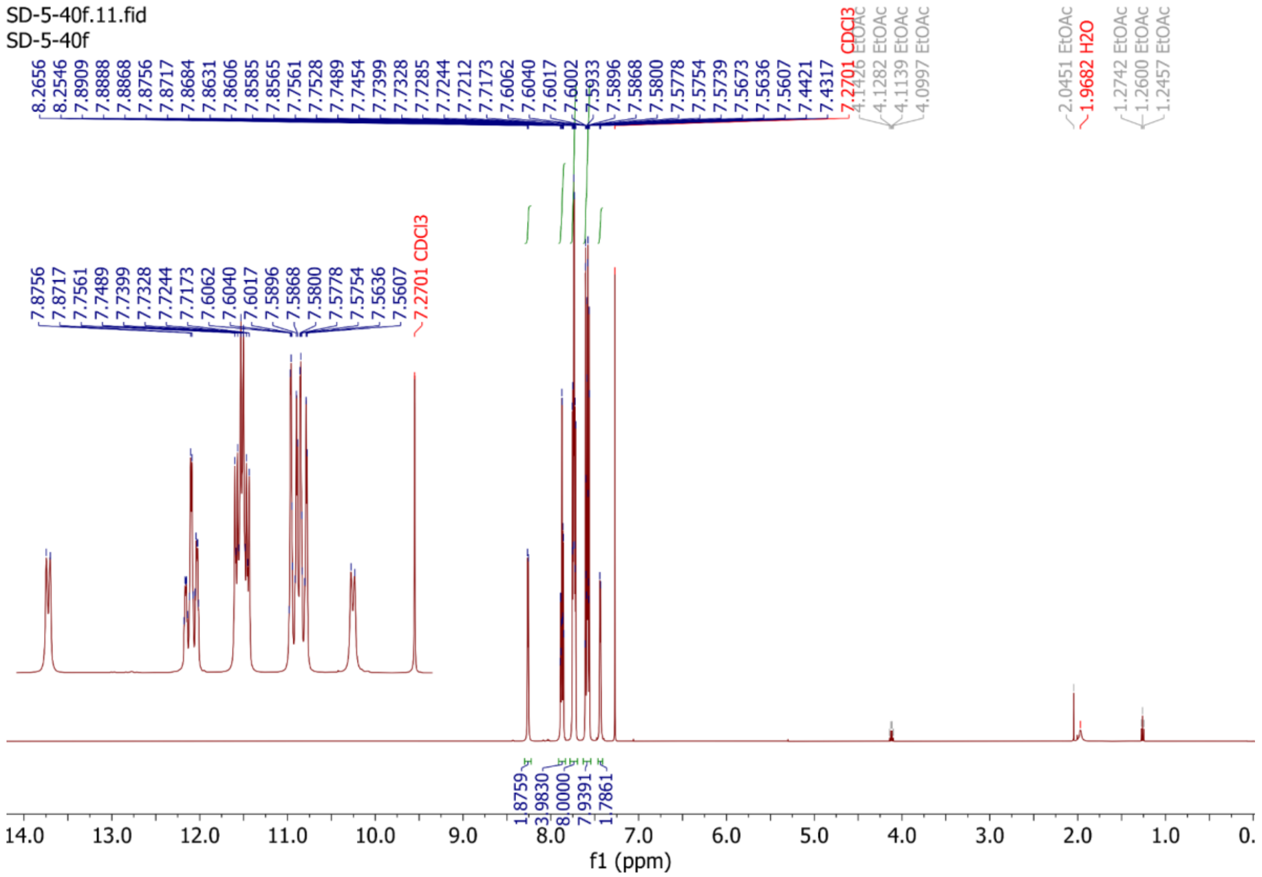

Tetraphenylphosphonium trifluoro(pyridin-4-yl)borate (**6**) (^{11}B, $CDCl_3$, 128 MHz) (not background suppressed).

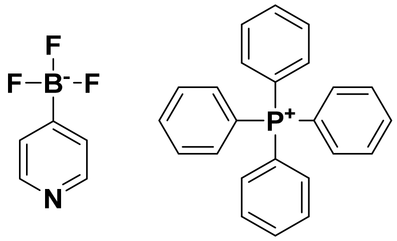

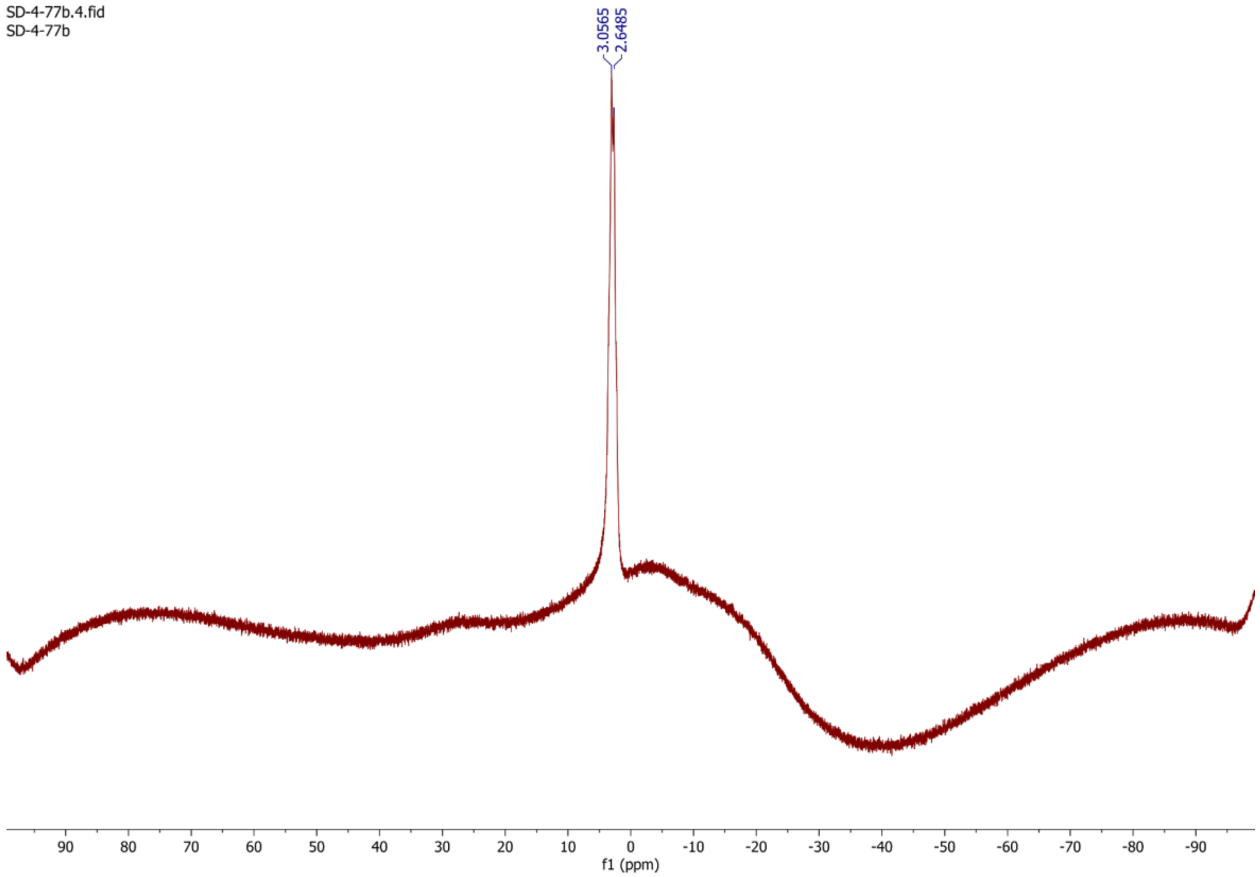

Tetraphenylphosphonium trifluoro(pyridin-4-yl)borate (**6**) ($^{13}C\{H\}$, $CDCl_3$, 126 MHz)

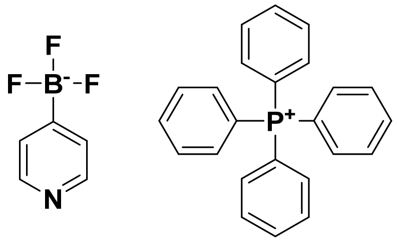

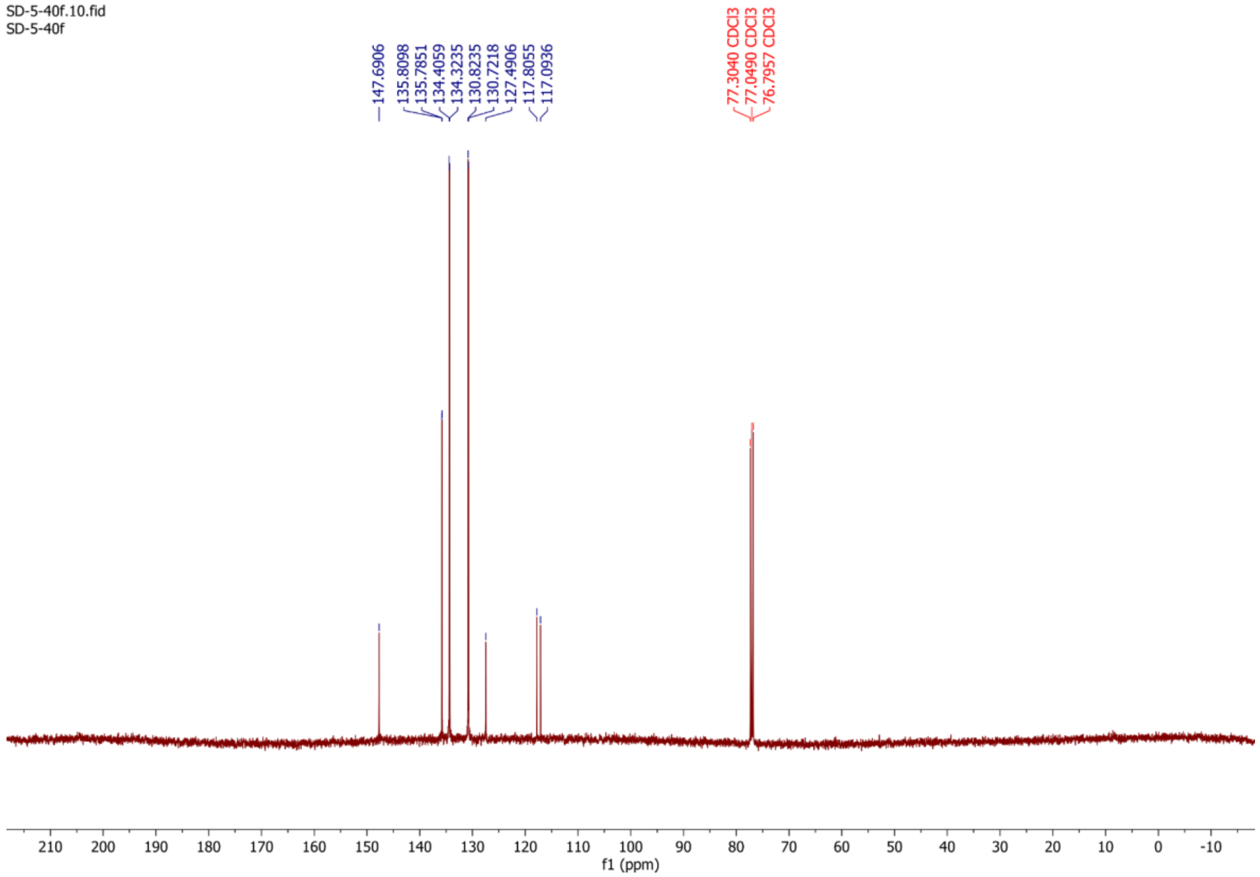

Tetraphenylphosphonium trifluoro(pyridin-4-yl)borate (**6**) ($^{19}F\{H\}$, $CDCl_3$, 376 MHz)

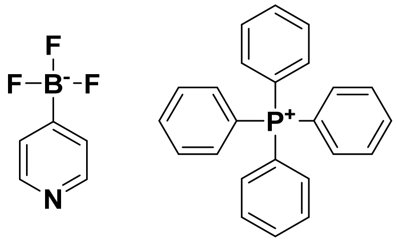

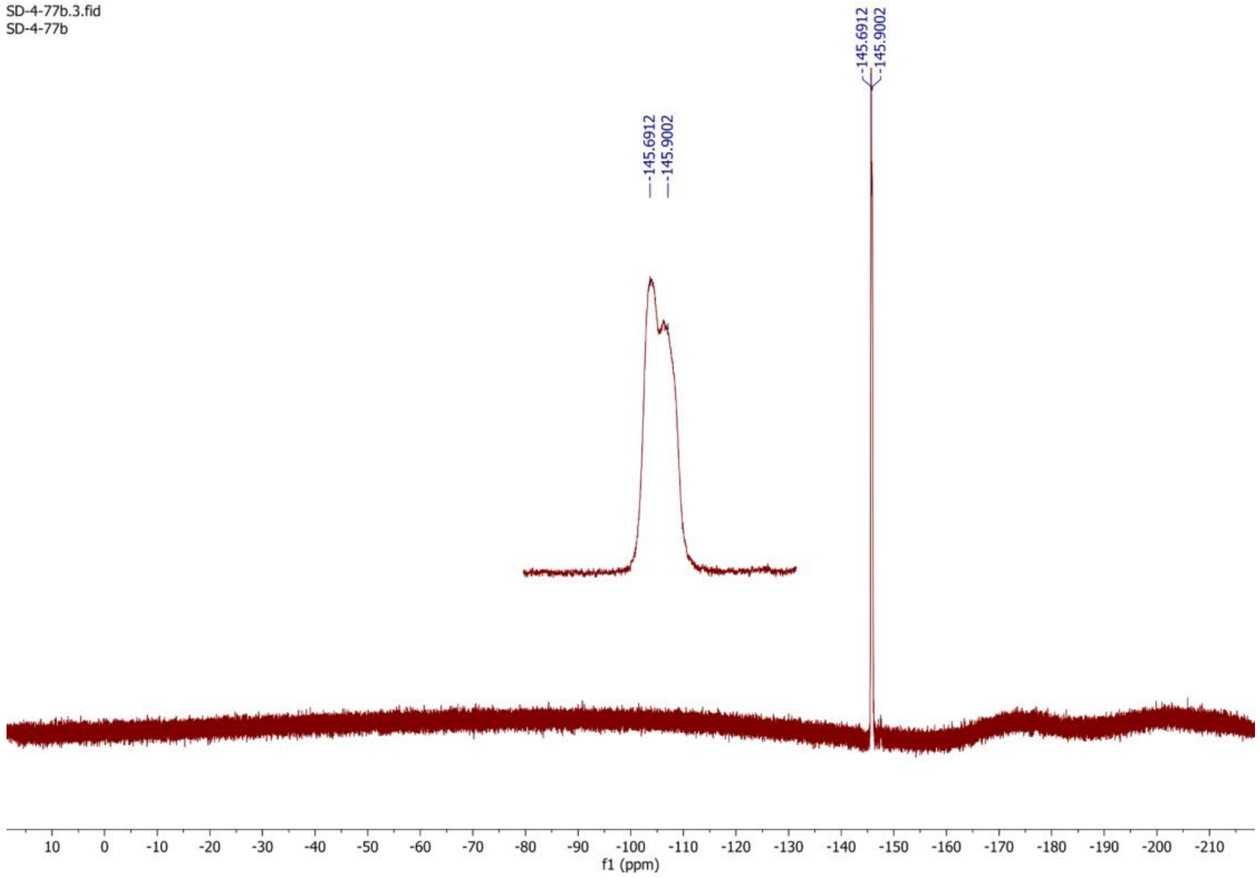

Tetraphenylphosphonium trifluoro(pyridin-4-yl)borate (**6**) ($^{31}P\{H\}$, $CDCl_3$, 203 MHz)

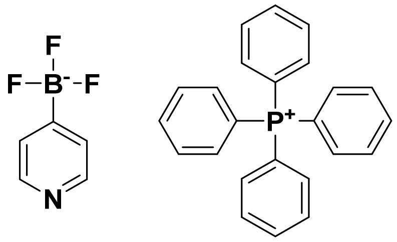

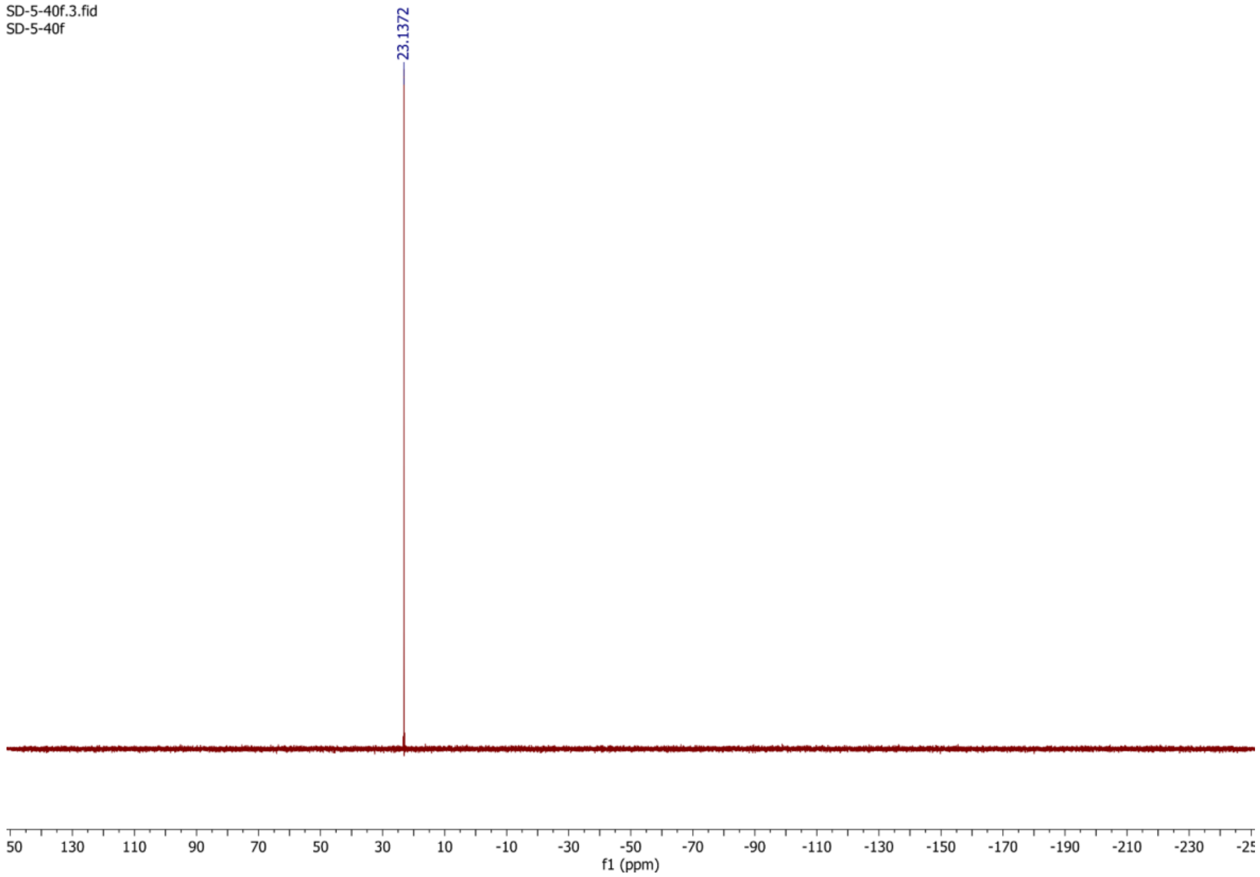

Tetrakis(3,5-dimethoxyphenyl)phosphonium trifluoro(pyridin-3-yl)borate (**7**)(^{1}H, (CD_2Cl_2, 500 MHz)

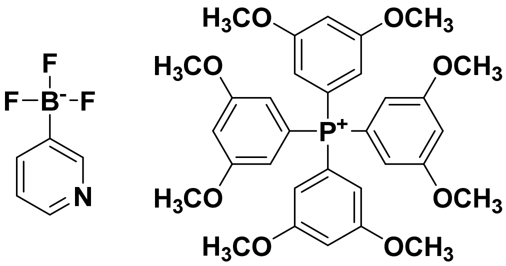

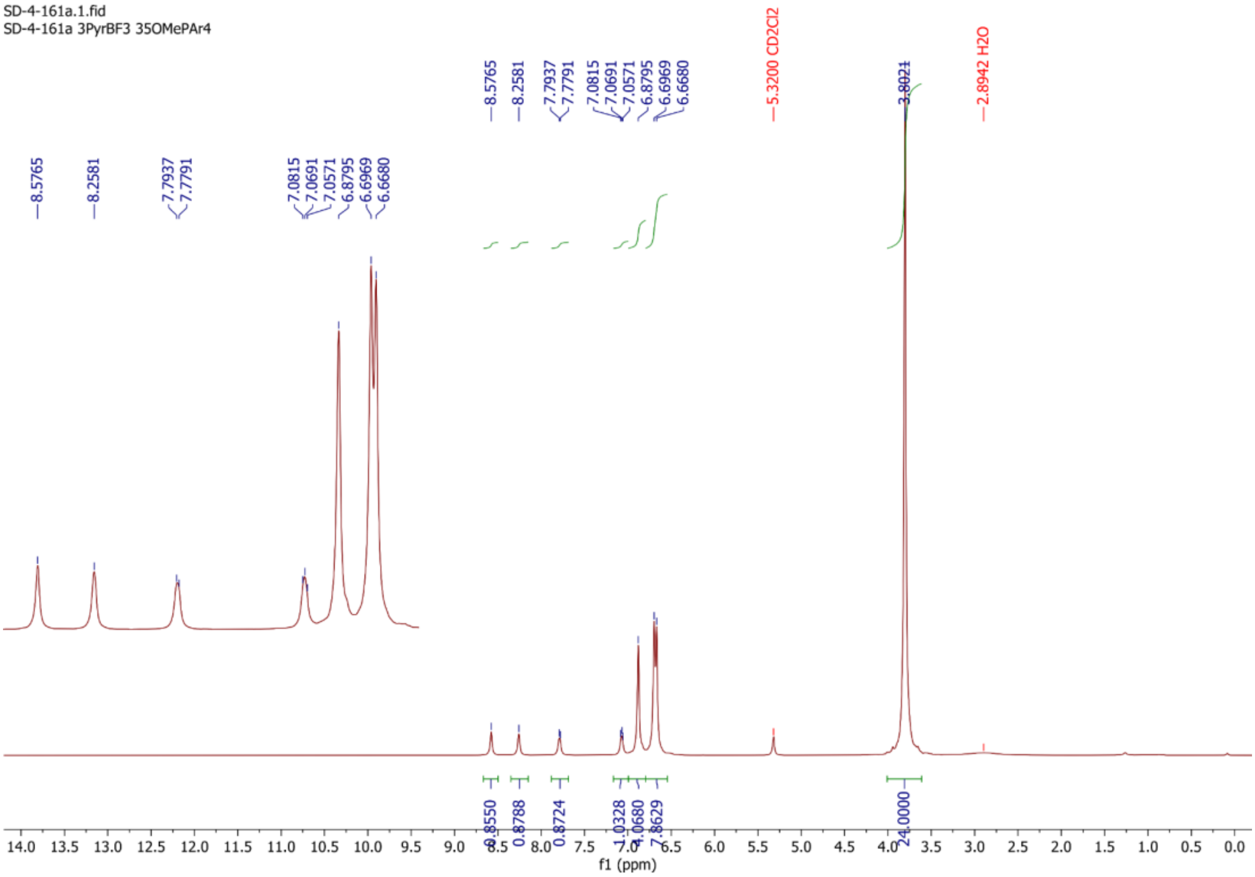

Tetrakis(3,5-dimethoxyphenyl)phosphonium trifluoro(pyridin-3-yl)borate (**7**)(^{11}B, (CD_2Cl_2, 128 MHz)

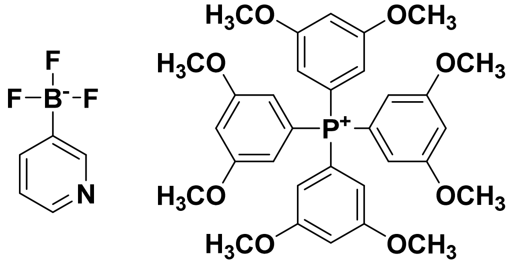

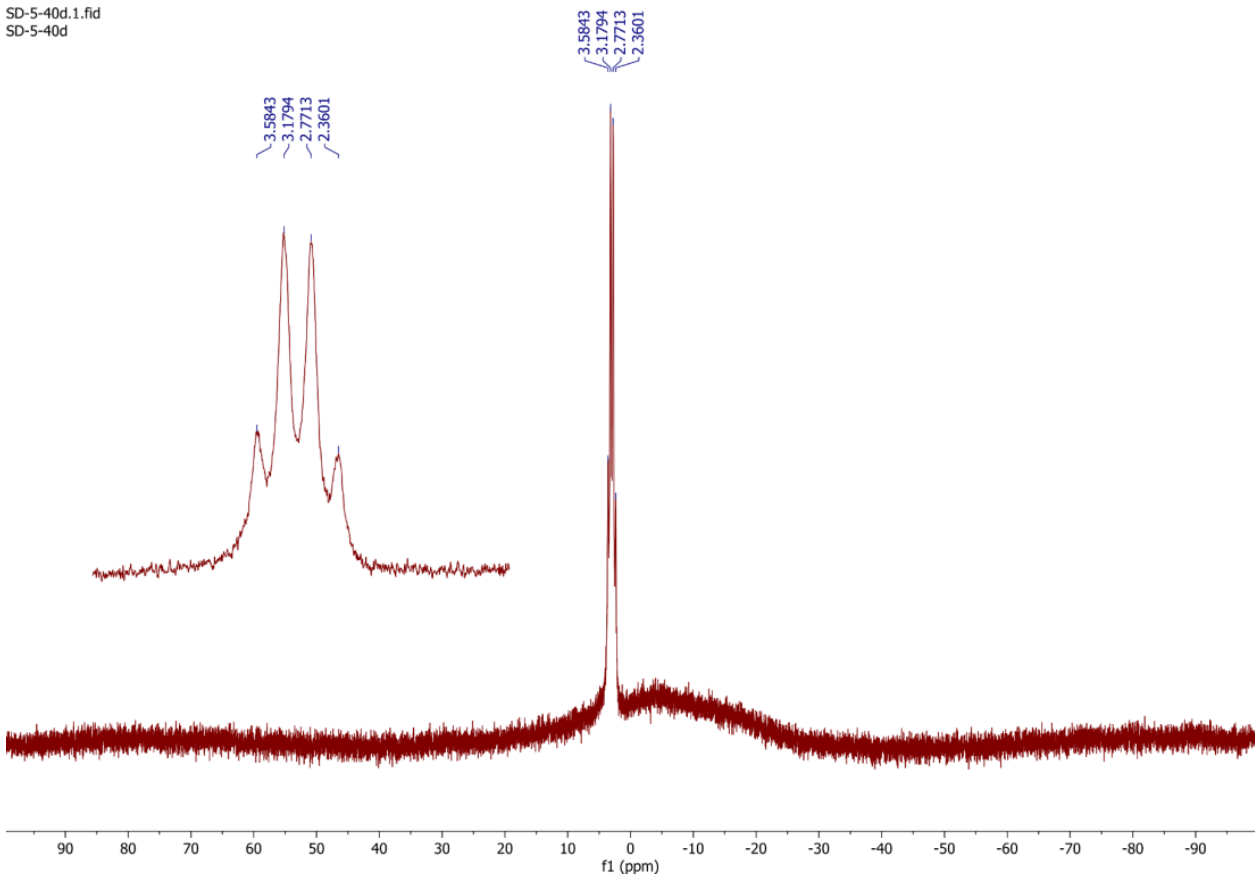

Tetrakis(3,5-dimethoxyphenyl)phosphonium trifluoro(pyridin-3-yl)borate (**7**) ($^{13}C\{H\}$, (CD_2Cl_2, 126 MHz)

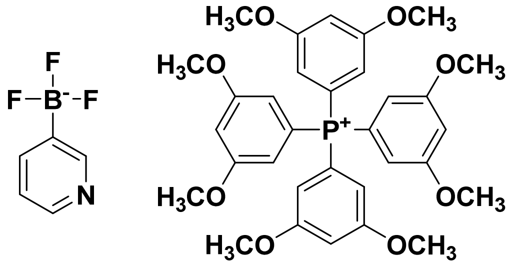

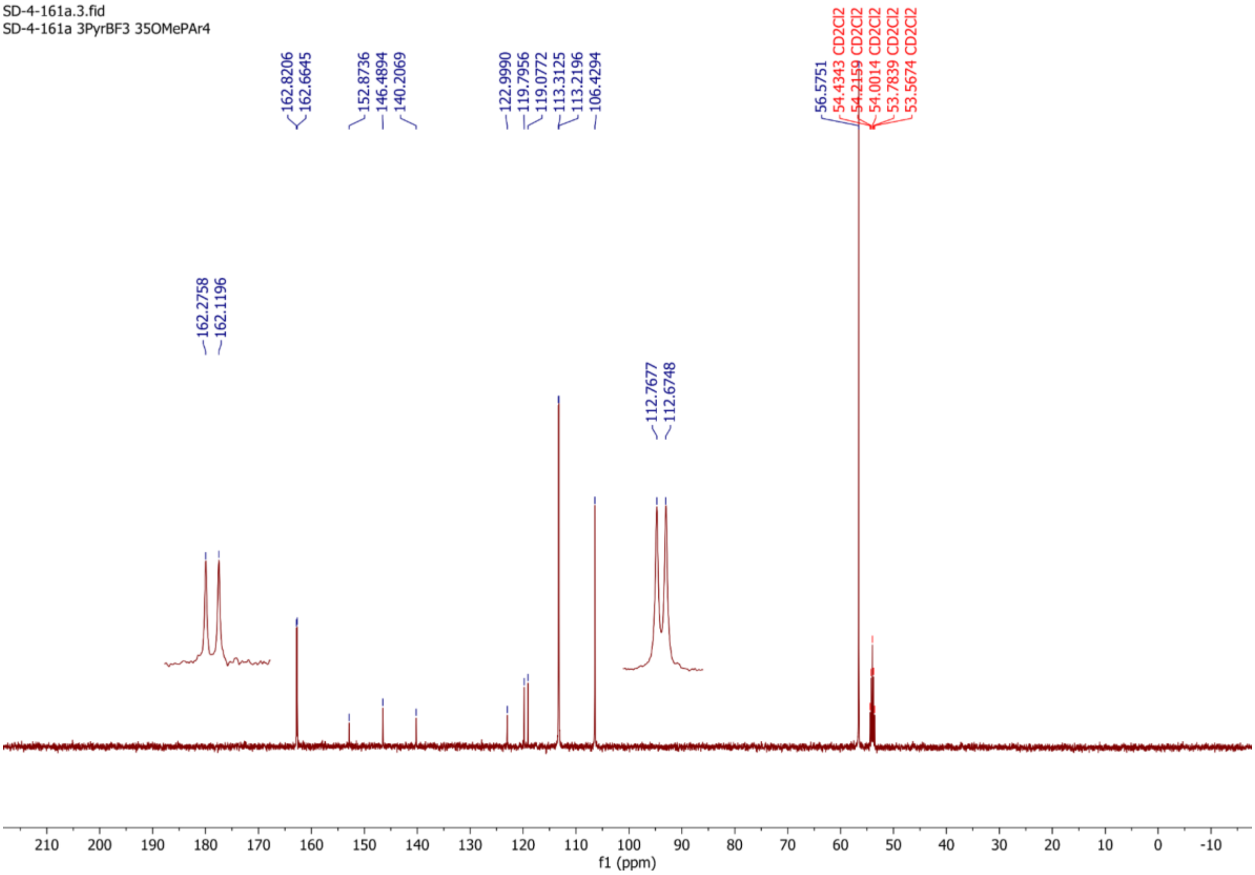

Tetrakis(3,5-dimethoxyphenyl)phosphonium trifluoro(pyridin-3-yl)borate (**7**)($^{19}F\{H\}$, (CD_2Cl_2, 376 MHz)

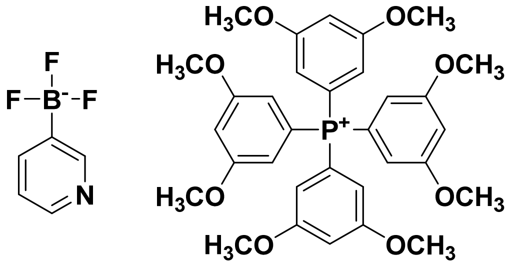

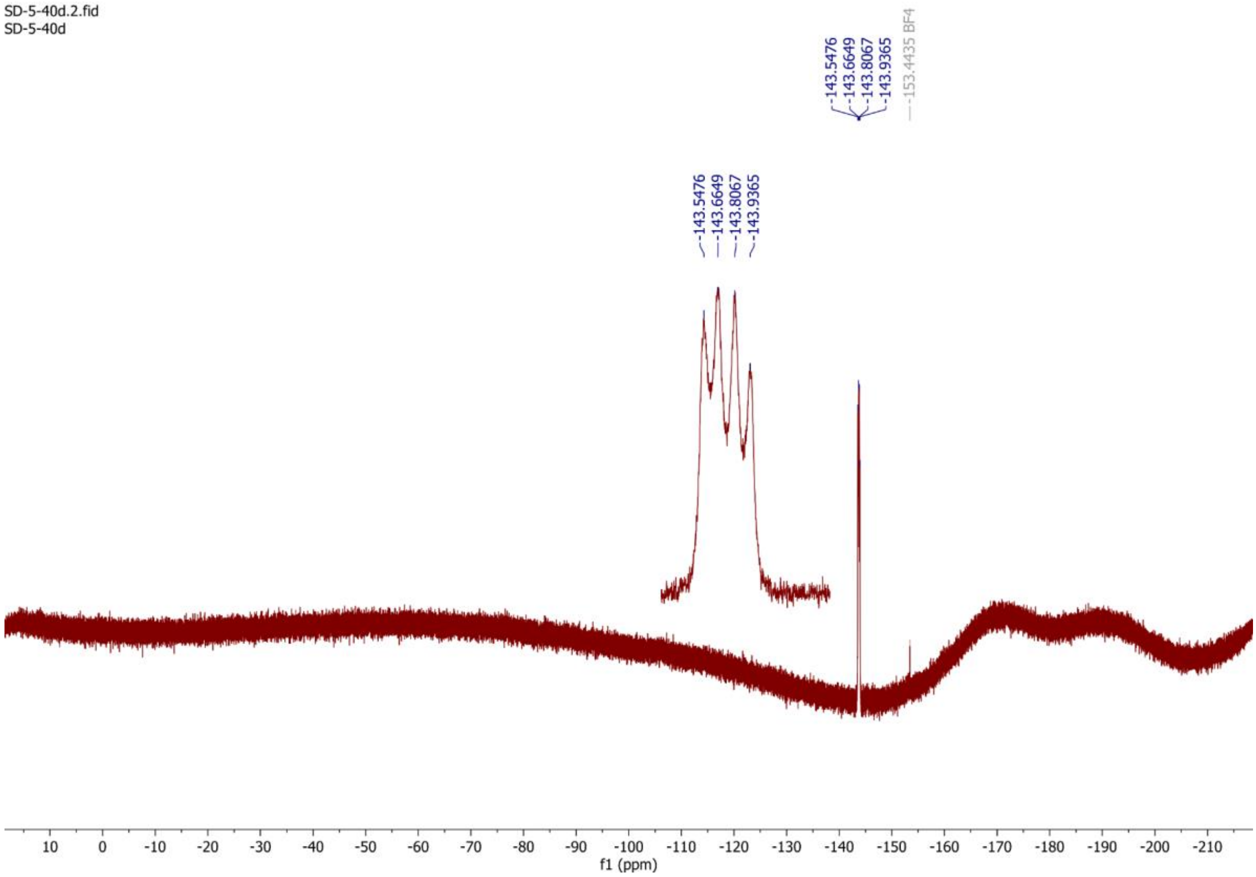

Tetrakis(3,5-dimethoxyphenyl)phosphonium trifluoro(pyridin-3-yl)borate (**7**)(^{31}P{H}, (CD_2Cl_2, 203 MHz)

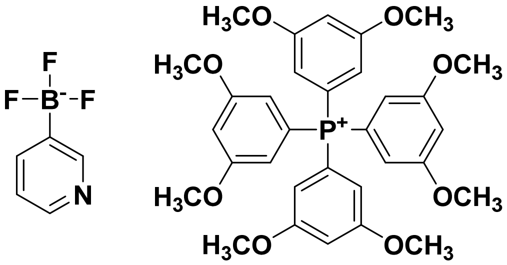

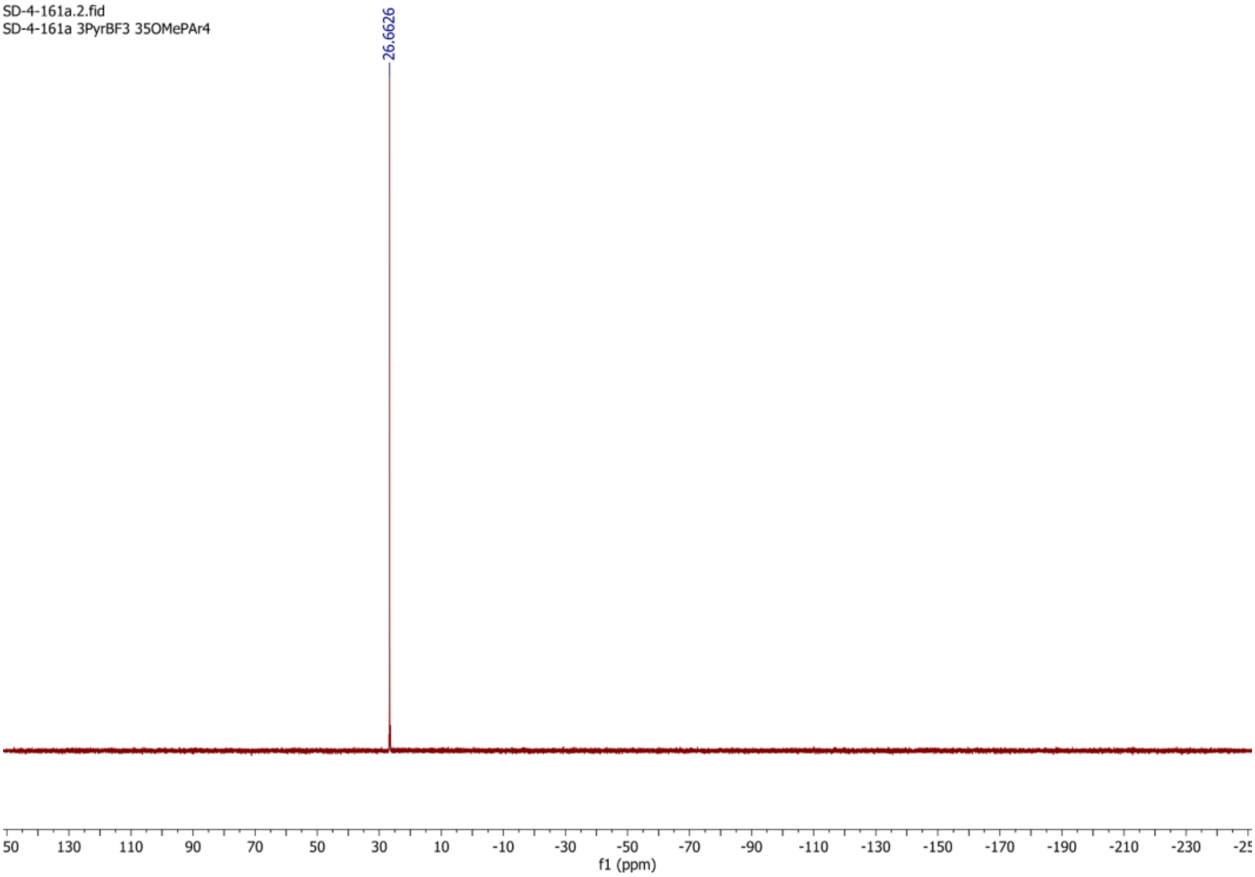

Tetrakis(3,5-dimethoxyphenyl)phosphonium trifluoro(pyridin-4-yl)borate (**8**)(^{1}H, (CD_2Cl_2, 500 MHz)

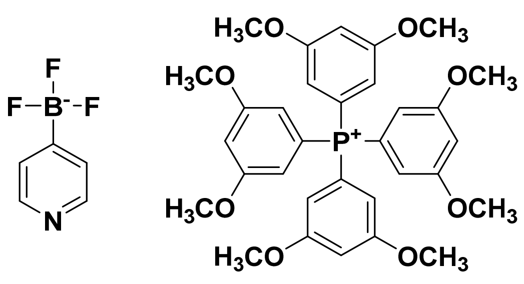

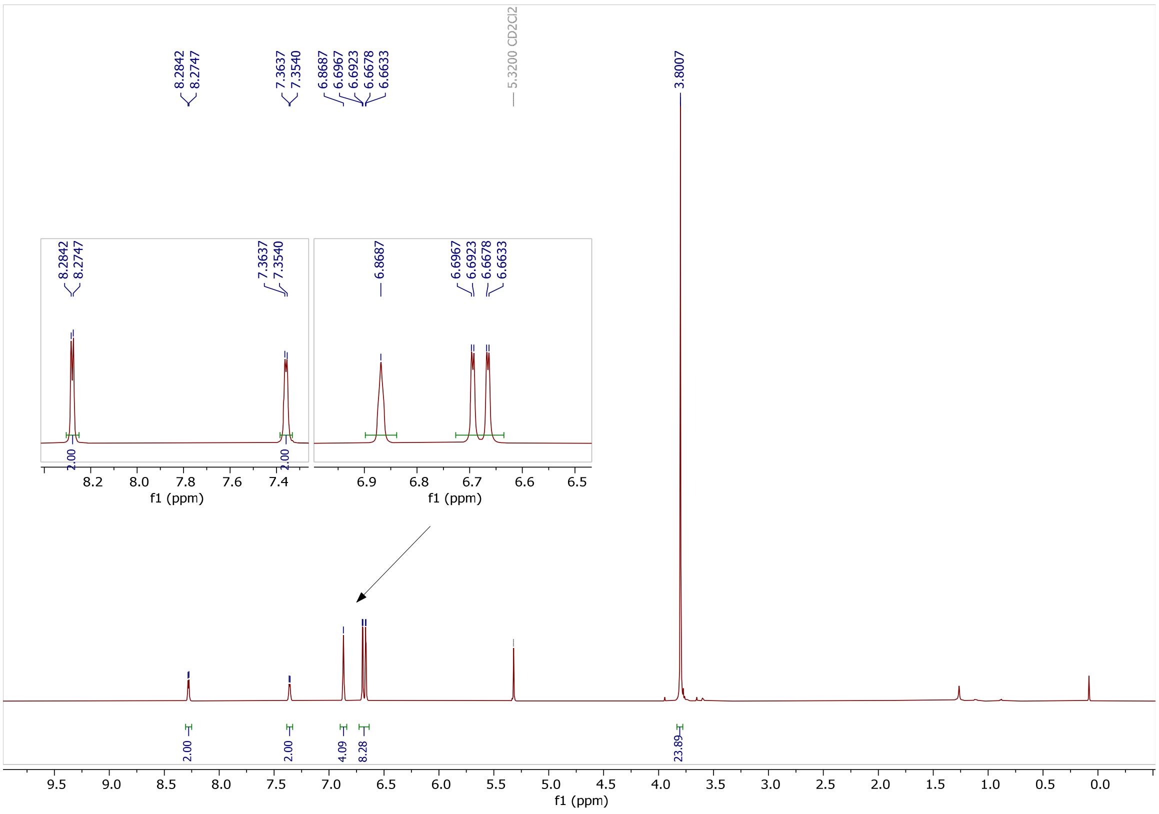

Tetrakis(3,5-dimethoxyphenyl)phosphonium trifluoro(pyridin-4-yl)borate (**8**)(^{11}B, (CD_2Cl_2, 160 MHz) (not background suppressed)

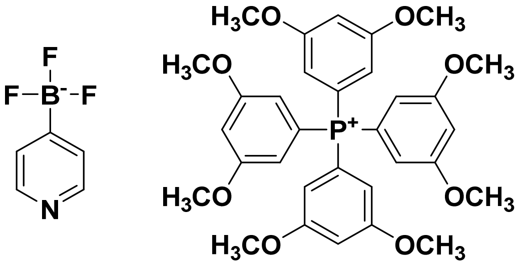

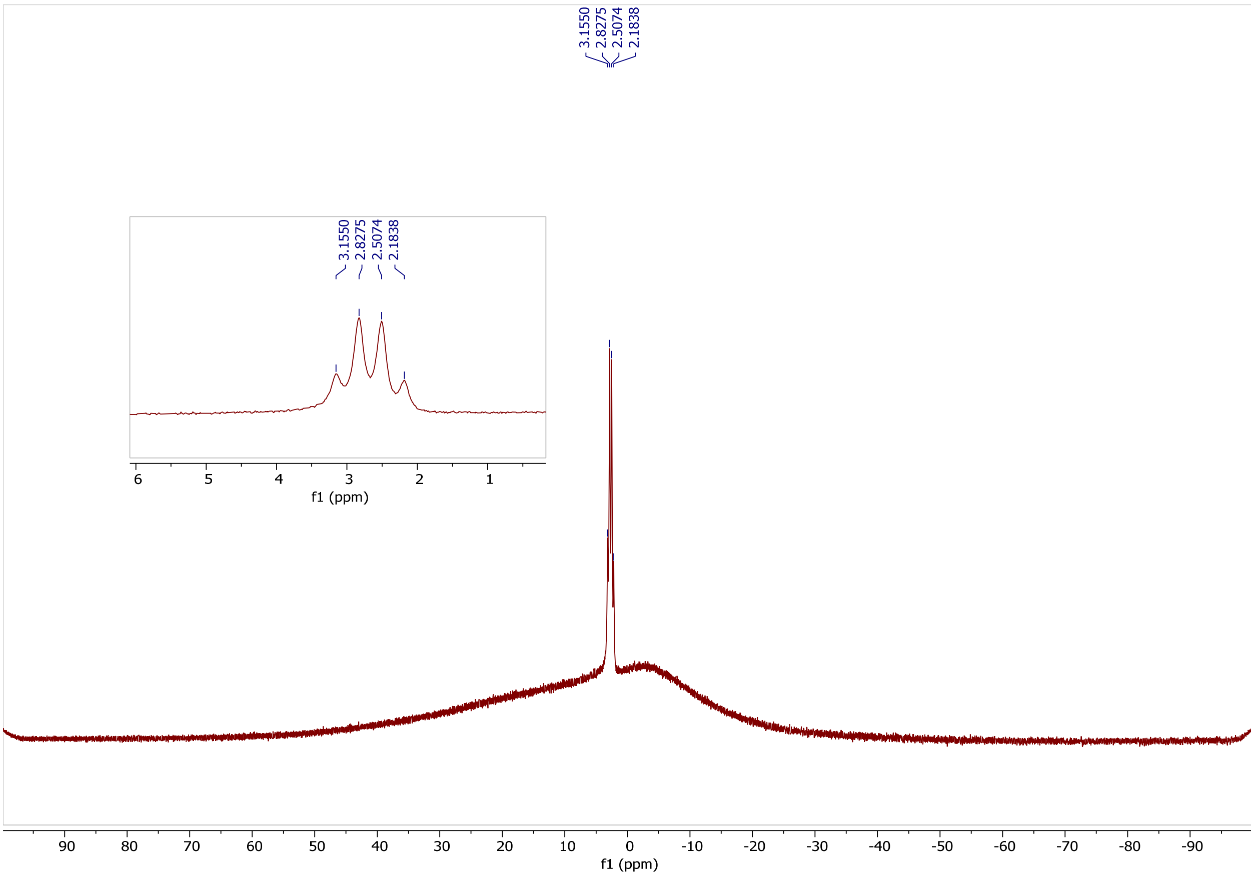

Tetrakis(3,5-dimethoxyphenyl)phosphonium trifluoro(pyridin-4-yl)borate (**8**)(^{13}C{H}, (CD_2Cl_2, 126 MHz)

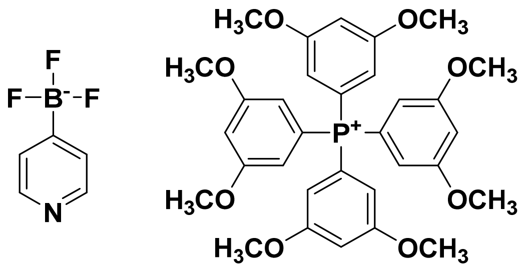

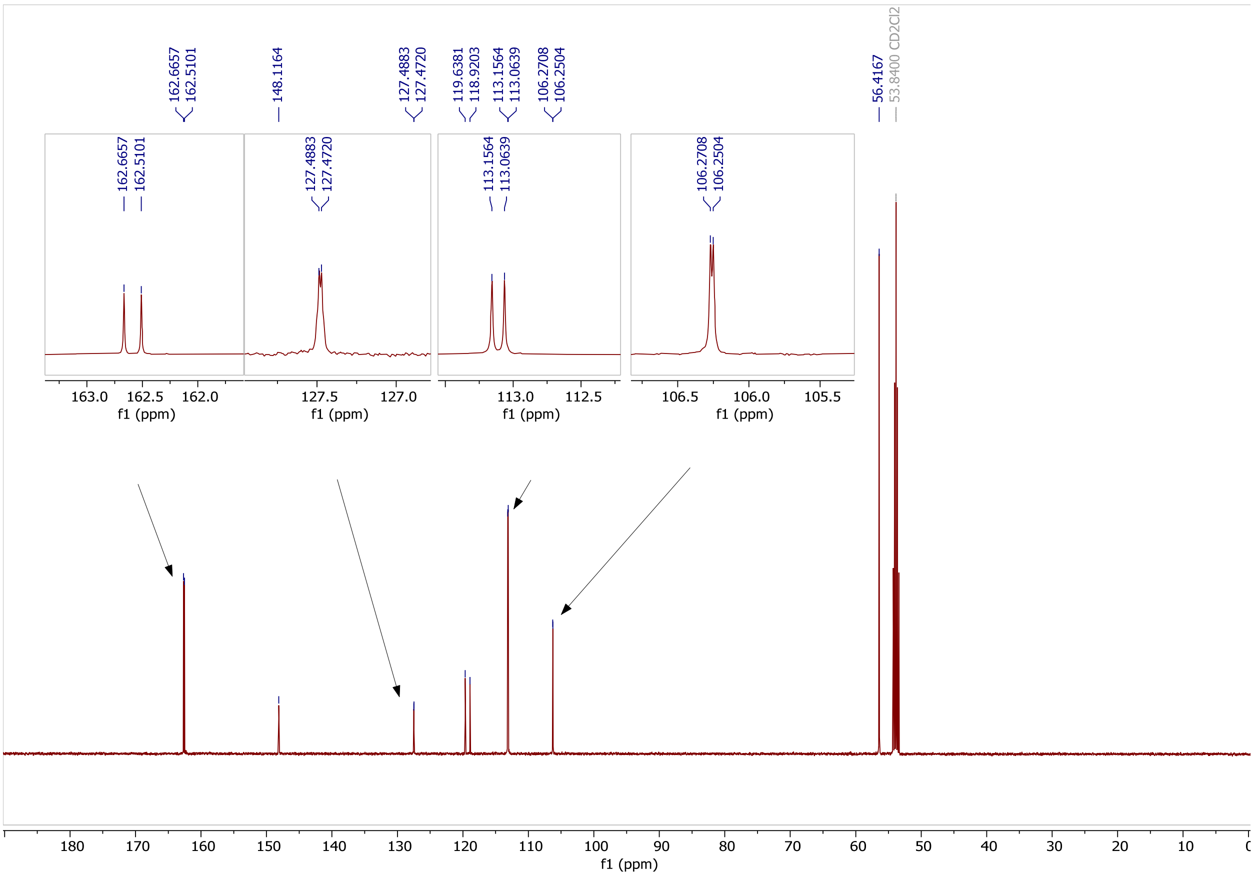

Tetrakis(3,5-dimethoxyphenyl)phosphonium trifluoro(pyridin-4-yl)borate (**8**)(^{19}F{H}, (CD_2Cl_2, 471 MHz)

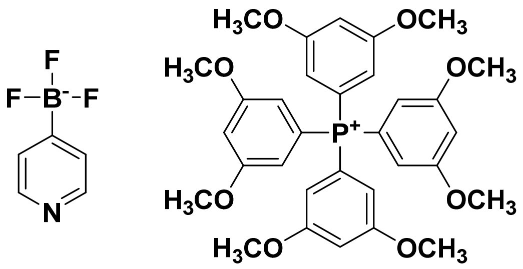

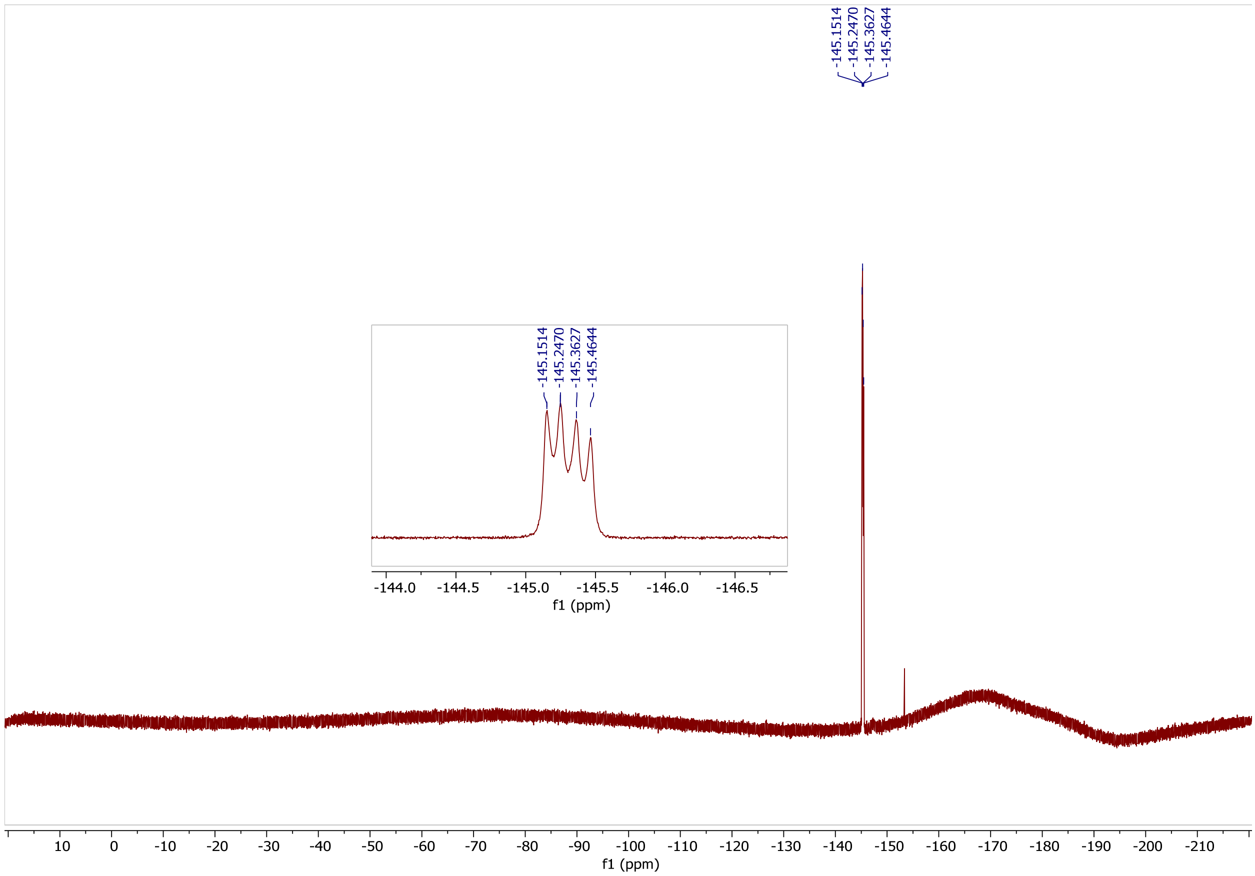

Tetrakis(3,5-dimethoxyphenyl)phosphonium trifluoro(pyridin-4-yl)borate (**8**)(^{31}P{H}, (CD_2Cl_2, 202 MHz)

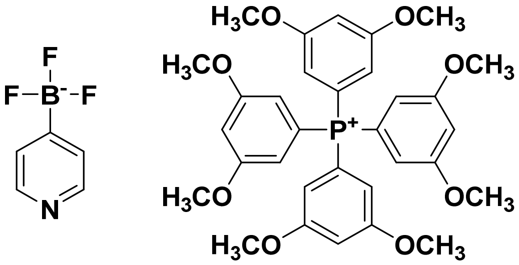

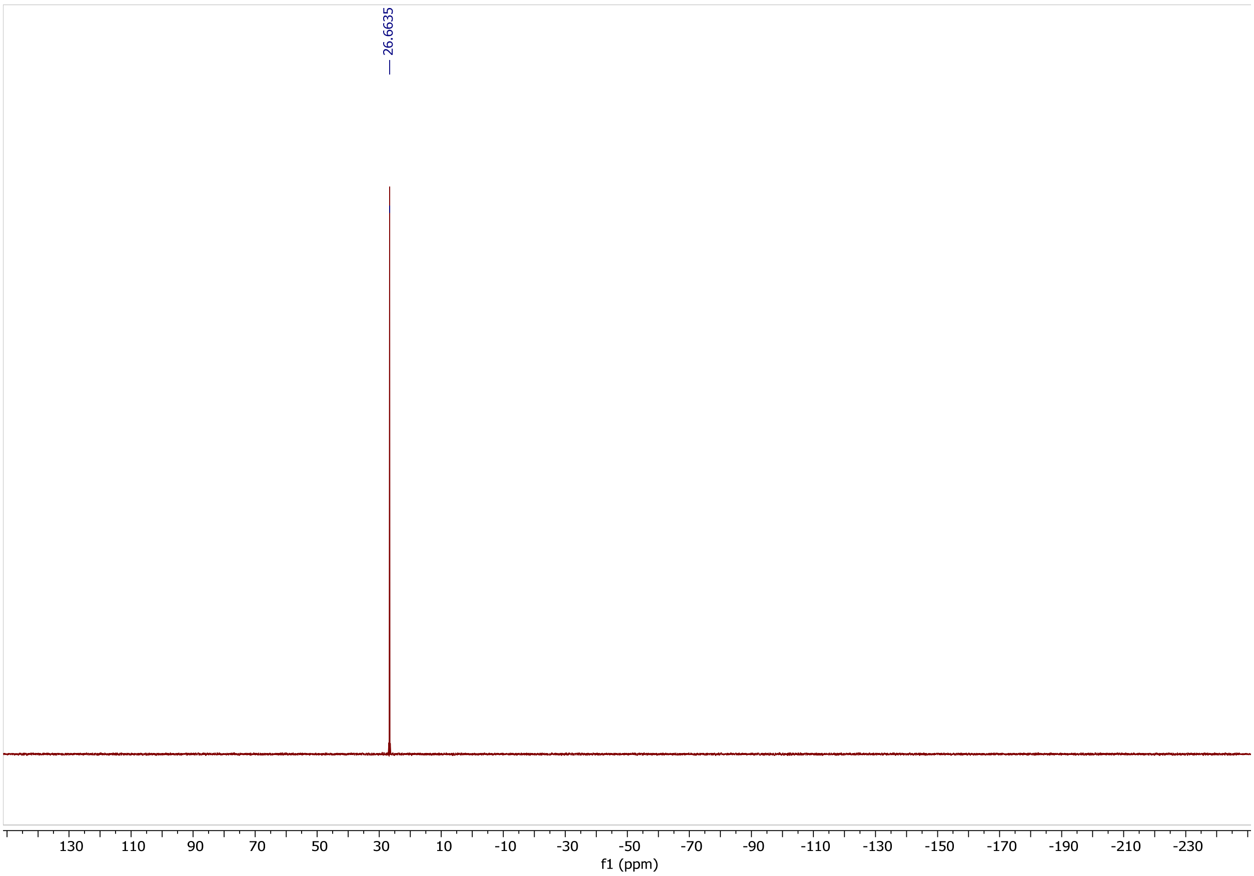

Tetrakis[4-(dimethylamino)phenyl]phosphonium trifluoro(pyridin-3-yl)borate (**9**) (^{1}H, $CDCl_3$, 500 MHz)

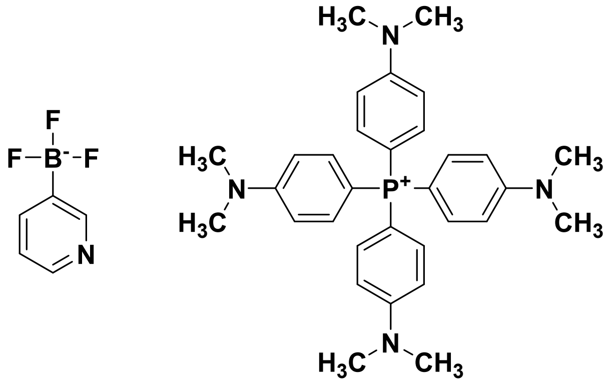

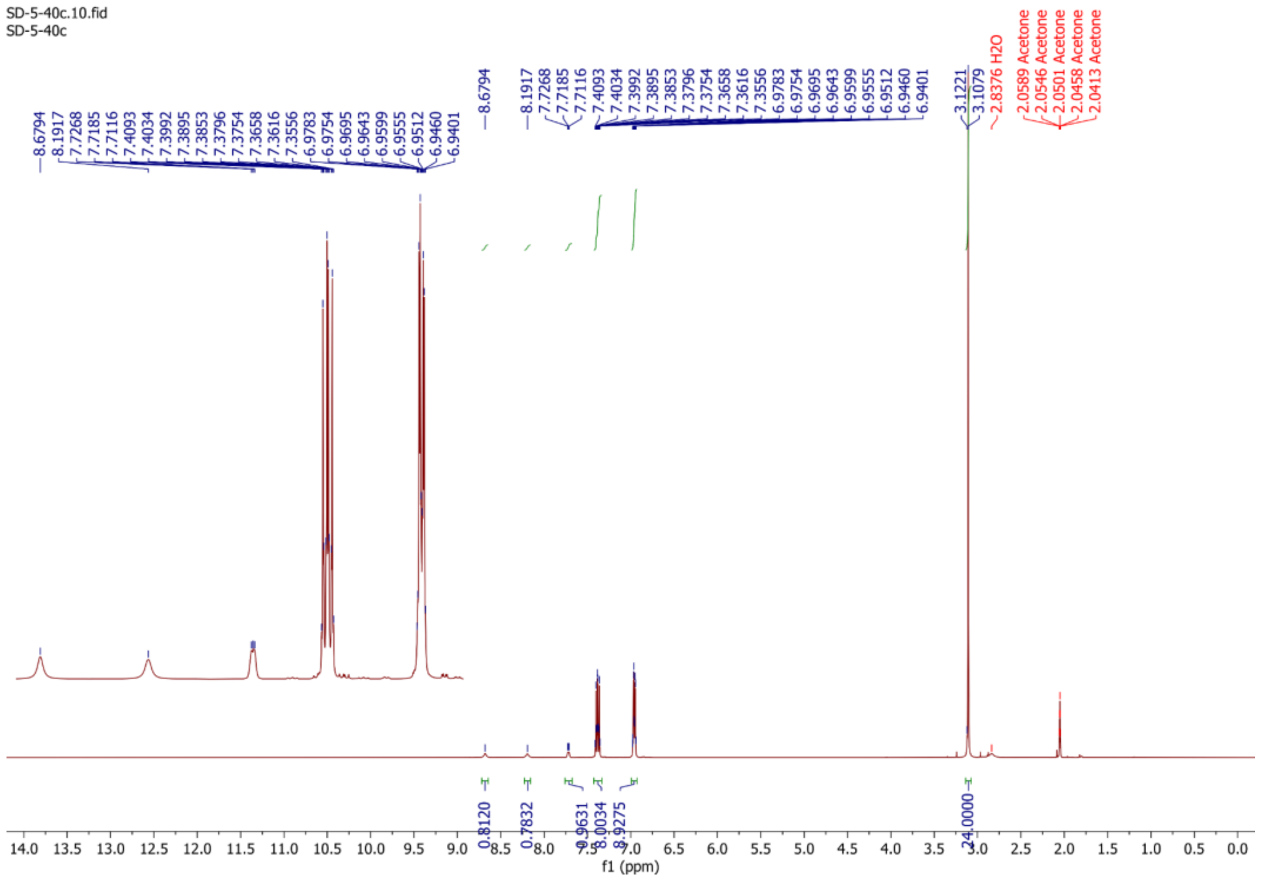

Tetrakis[4-(dimethylamino)phenyl]phosphonium trifluoro(pyridin-3-yl)borate (**9**) (^{11}B, $(CD_3)_2CO$, 128 MHz)

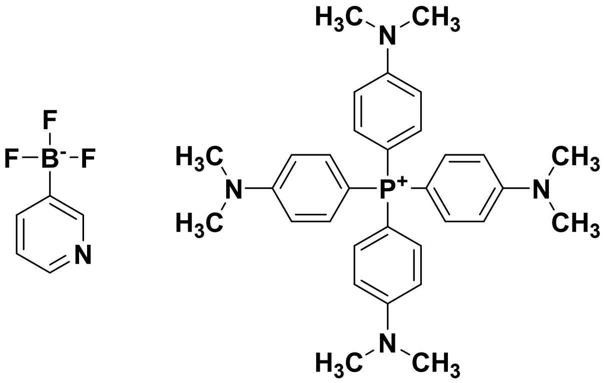

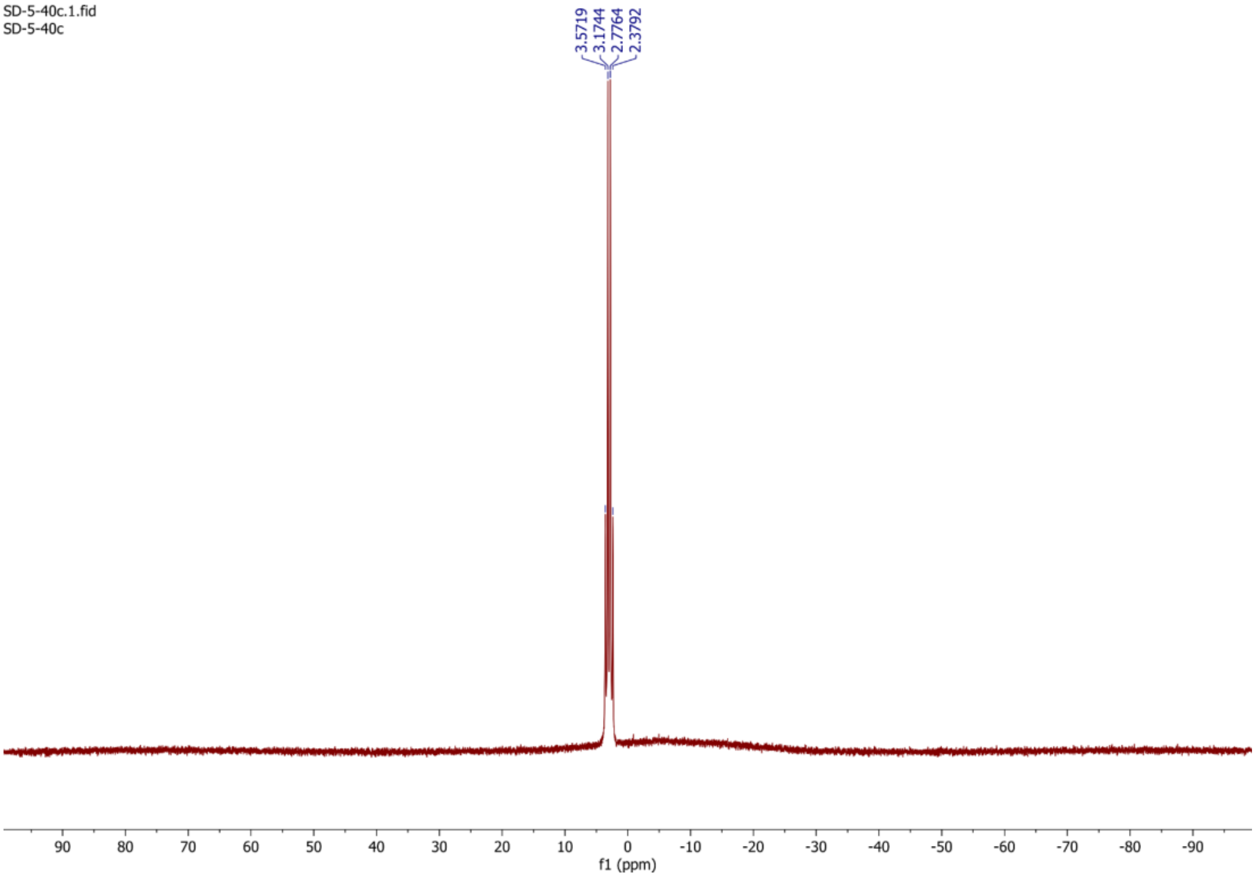

Tetrakis[4-(dimethylamino)phenyl]phosphonium trifluoro(pyridin-3-yl)borate (**9**) ($^{13}C\{H\}$, $(CD_3)_2CO$, 126 MHz)

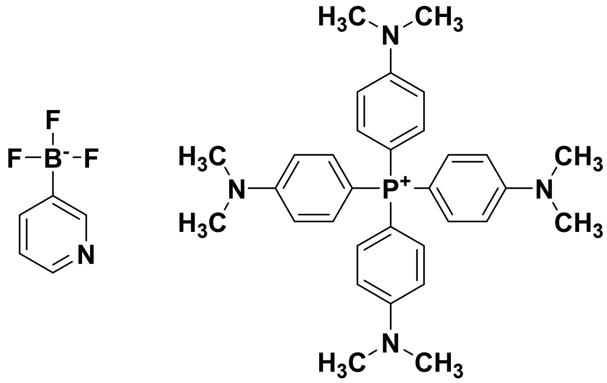

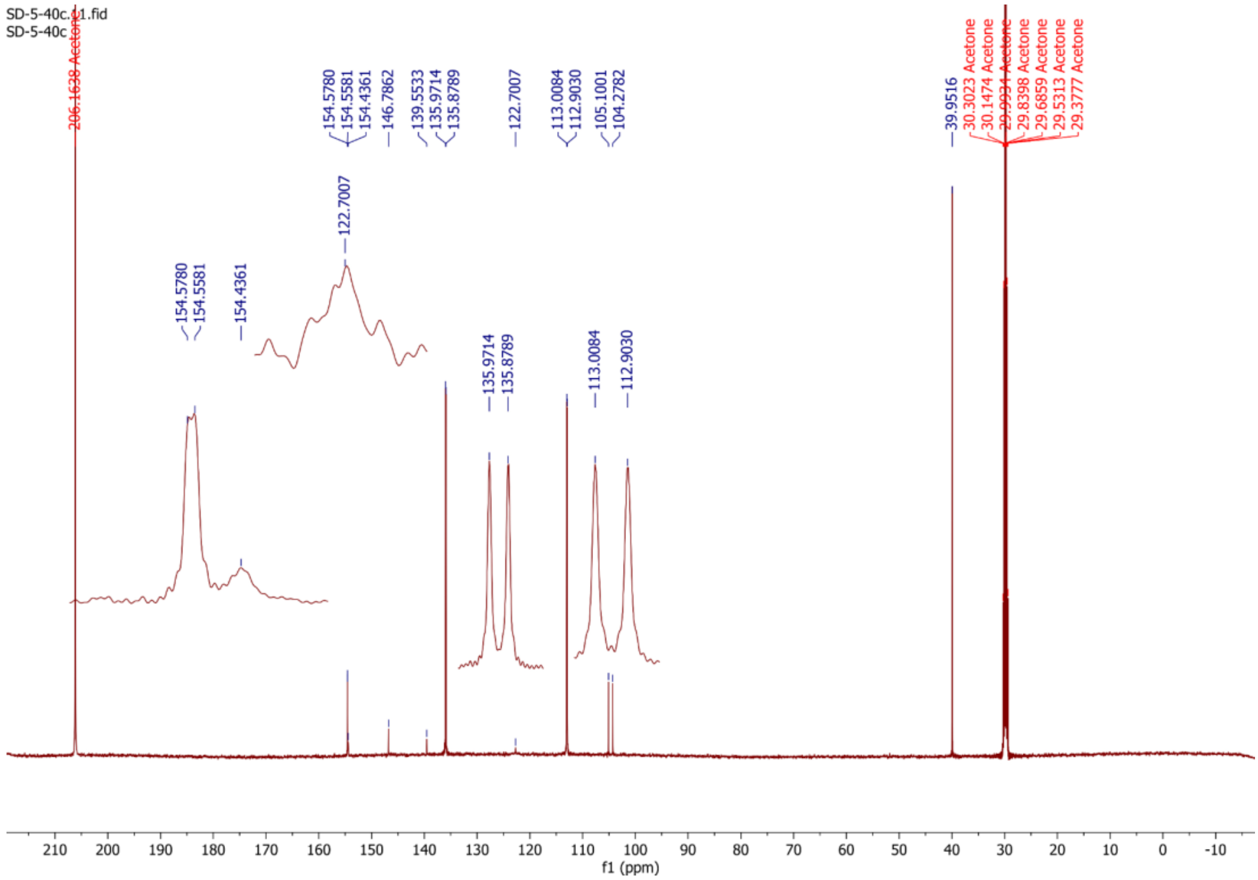

Tetrakis[4-(dimethylamino)phenyl]phosphonium trifluoro(pyridin-3-yl)borate (**9**) ($^{19}F\{H\}$, $(CD_3)_2CO$, 471 MHz)

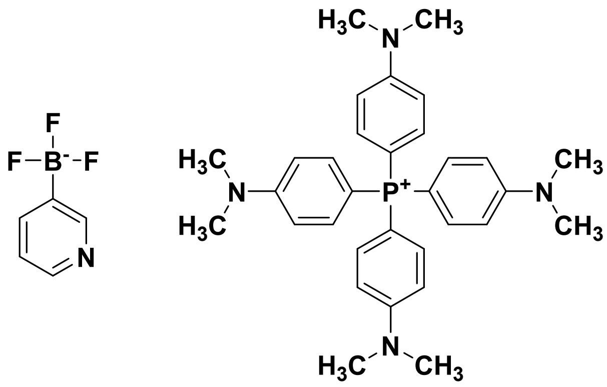

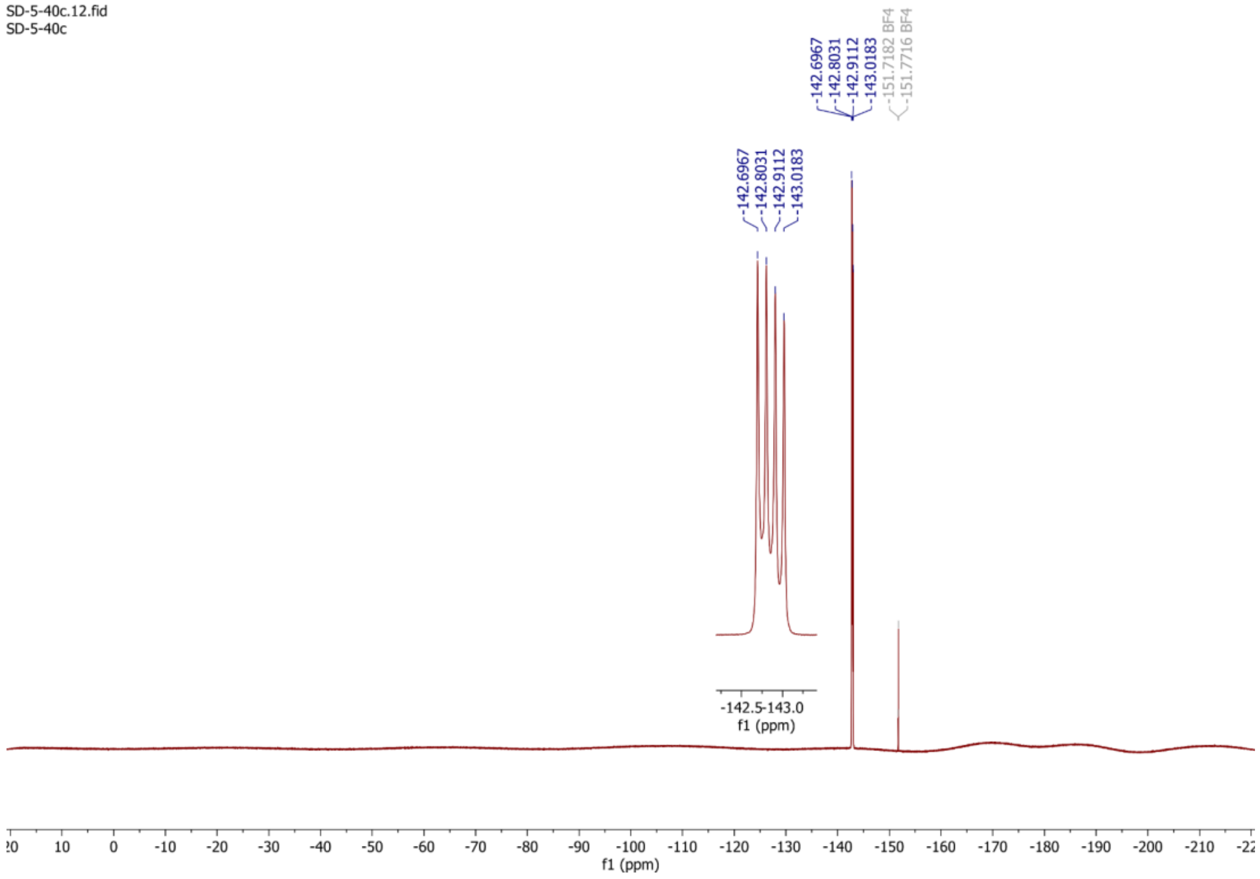

Tetrakis[4-(dimethylamino)phenyl]phosphonium trifluoro(pyridin-3-yl)borate (**9**) (^{31}P{H}, ($CDCl_3$, 203 MHz)

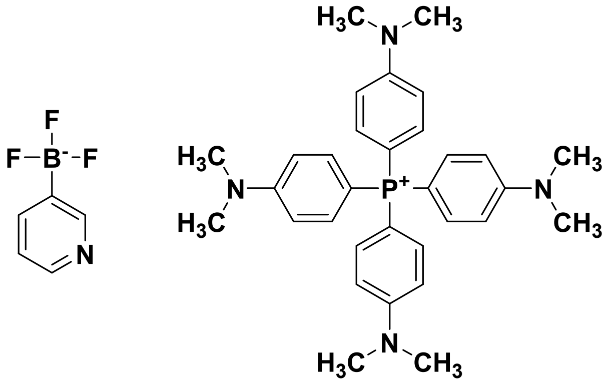

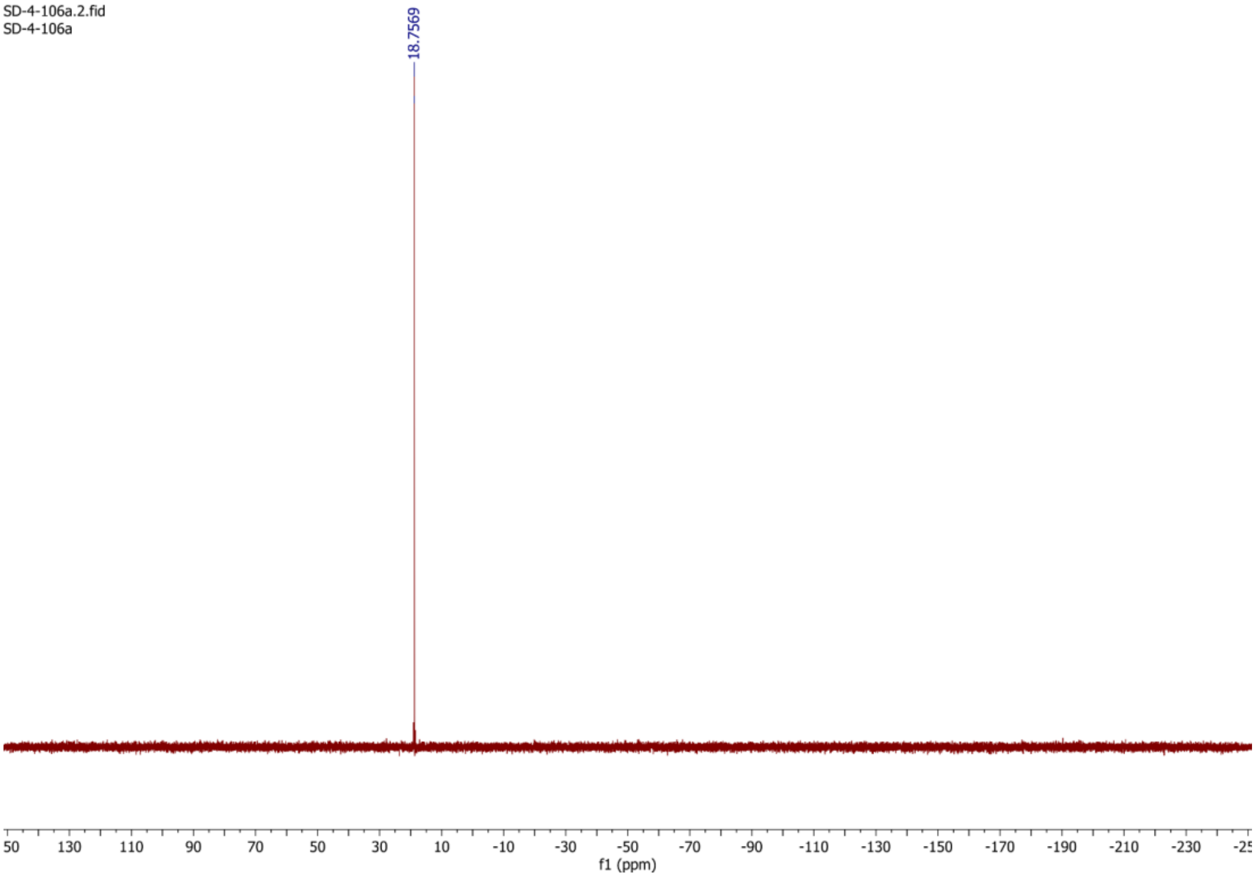

Tetrakis[4-(dimethylamino)phenyl]phosphonium trifluoro(pyridin-4-yl)borate (**10**) (^{1}H, CD_2Cl_2, 500 MHz)

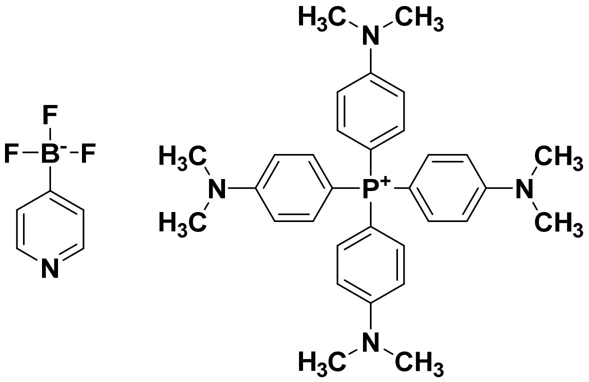

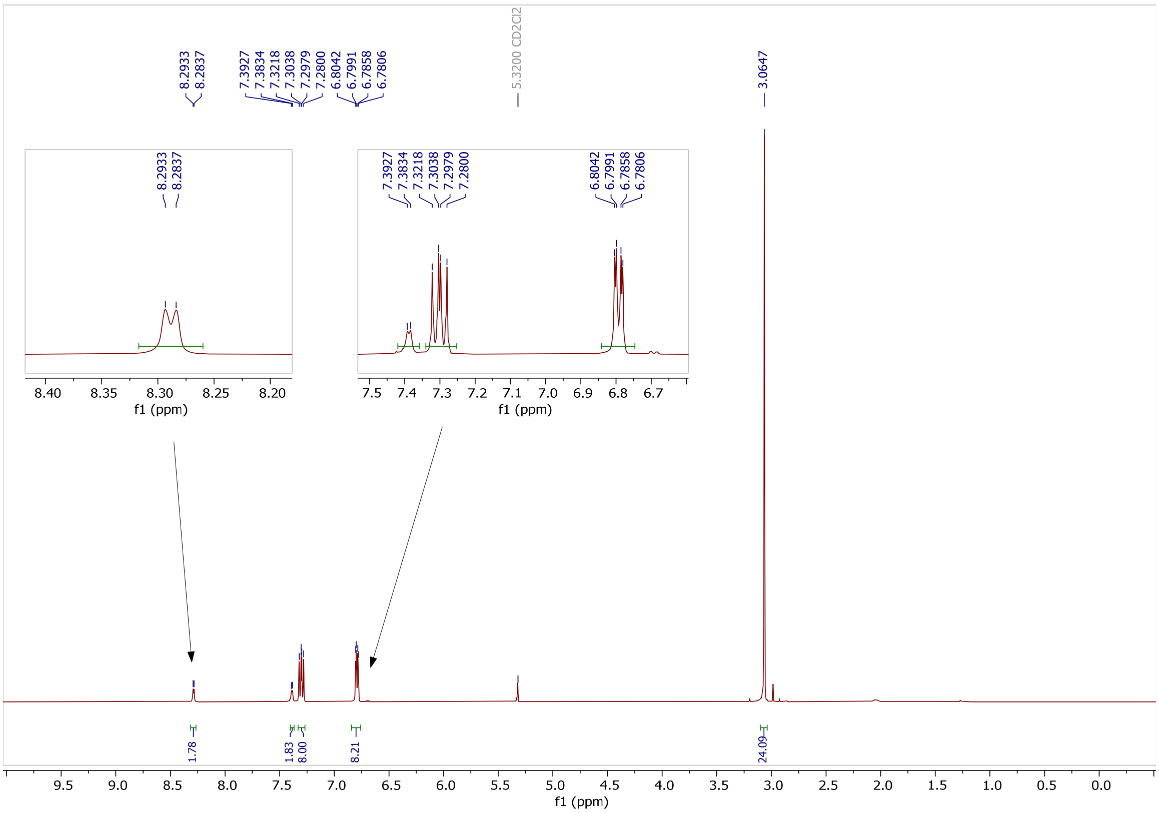

Tetrakis[4-(dimethylamino)phenyl]phosphonium trifluoro(pyridin-4-yl)borate (**10**) (^{11}B, CD_2Cl_2, 160 MHz) (not background suppressed)

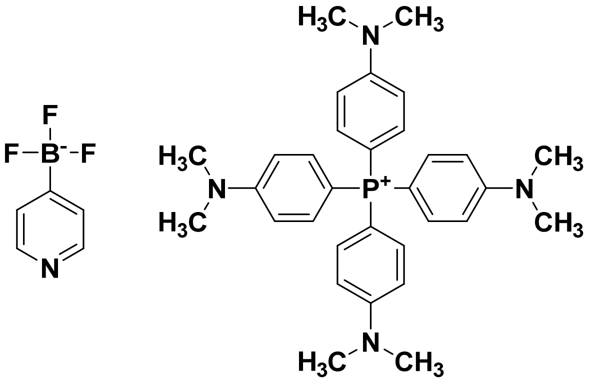

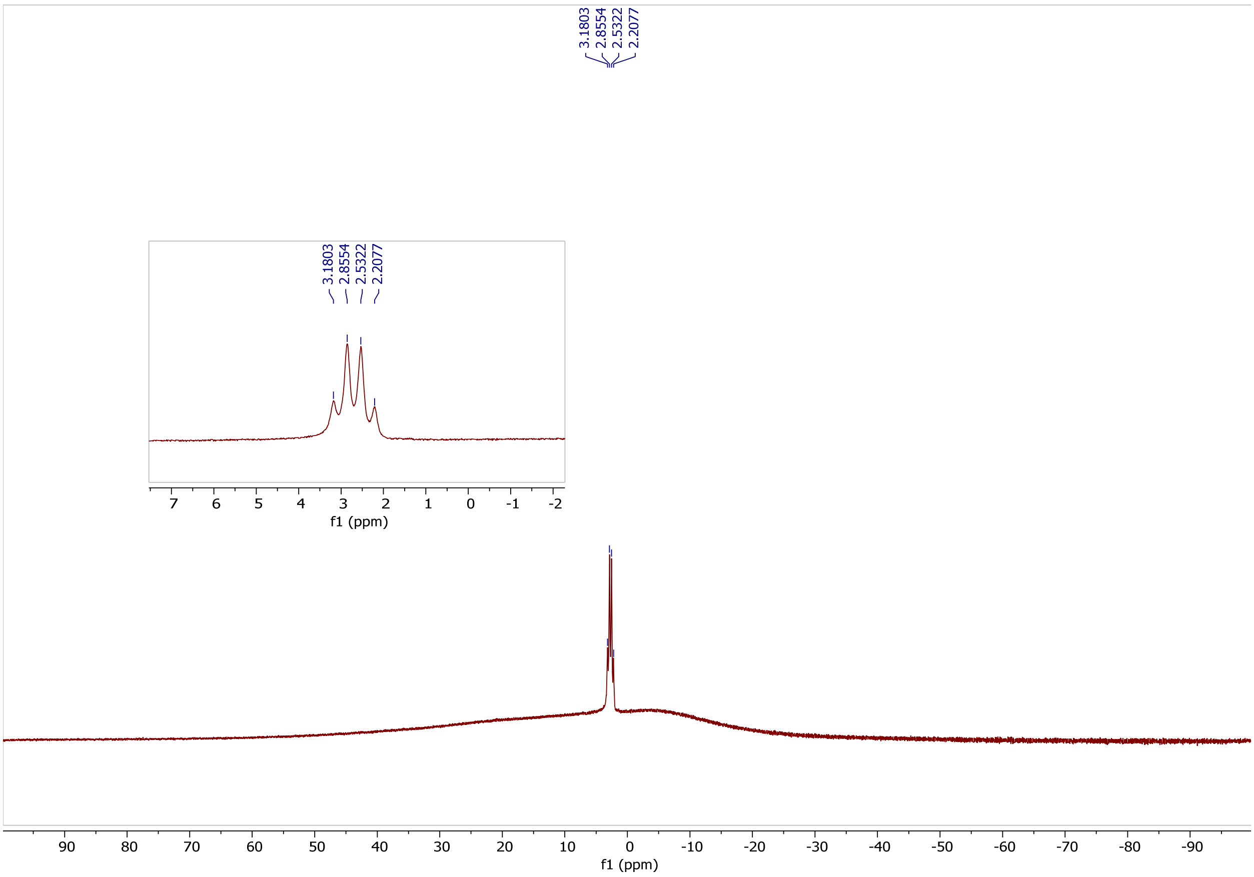

Tetrakis[4-(dimethylamino)phenyl]phosphonium trifluoro(pyridin-4-yl)borate (**10**) (^{13}C, CD_2Cl_2, 126 MHz)

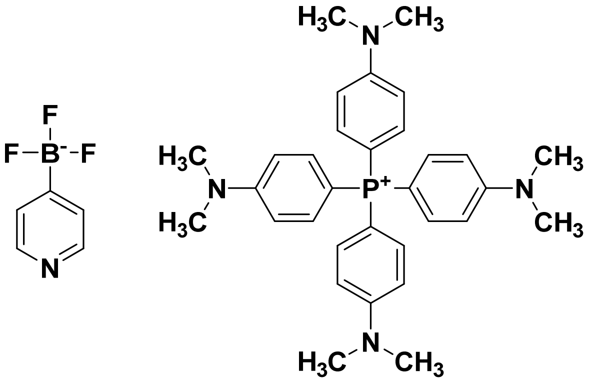

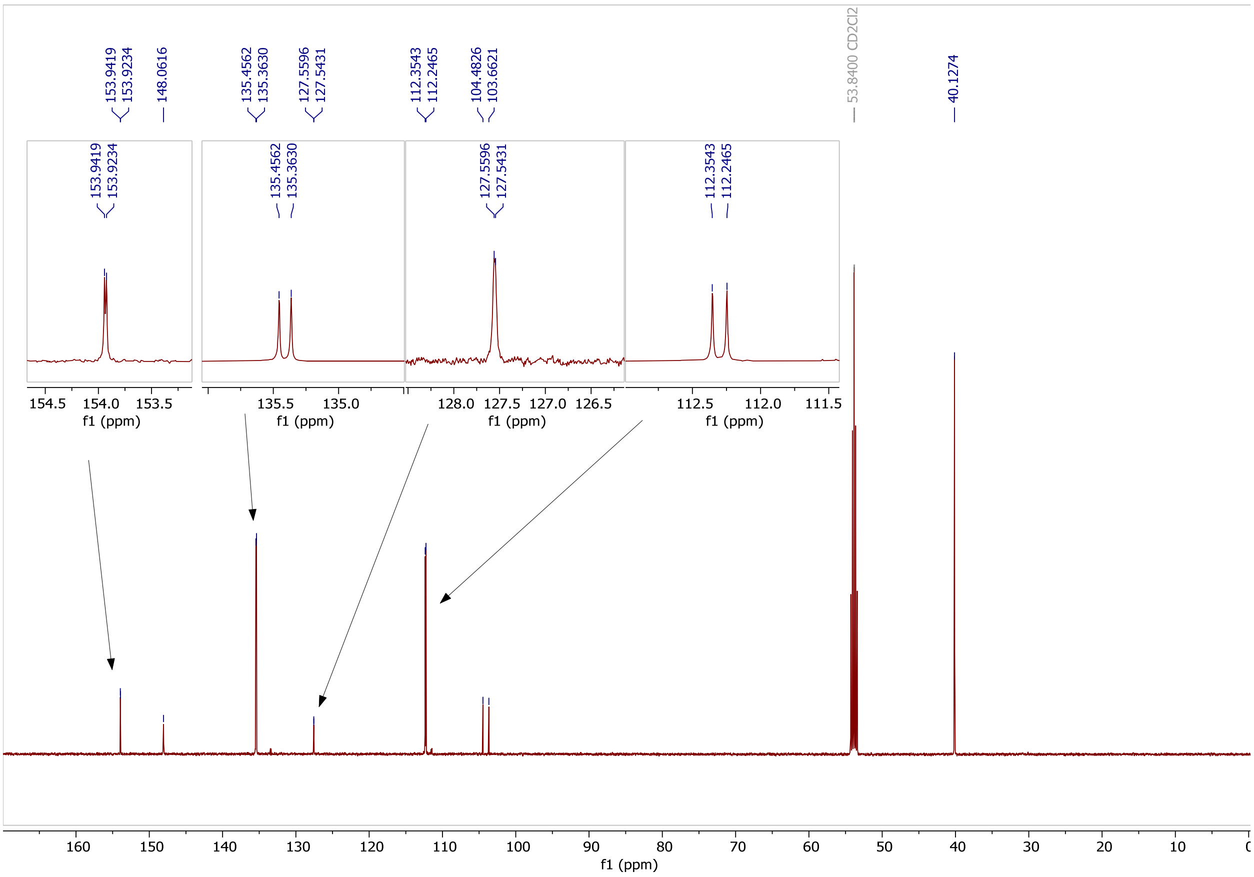

Tetrakis[4-(dimethylamino)phenyl]phosphonium trifluoro(pyridin-4-yl)borate (**10**) ($^{19}F\{H\}$, CD_2Cl_2, 471 MHz)

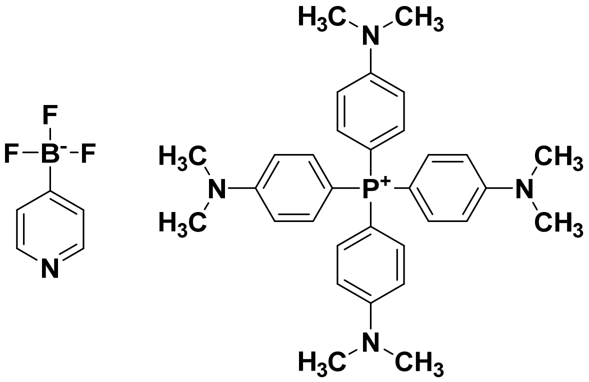

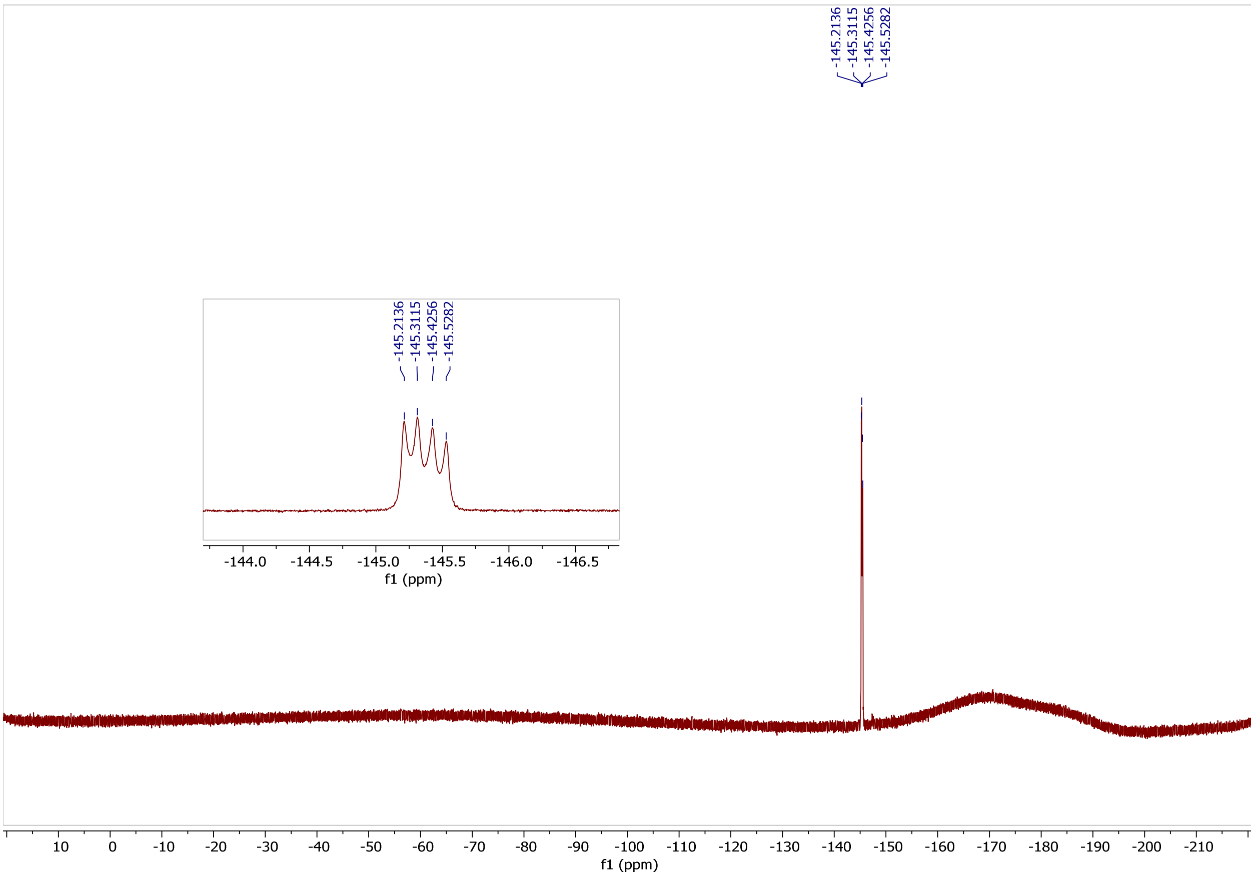

Tetrakis[4-(dimethylamino)phenyl]phosphonium trifluoro(pyridin-4-yl)borate (**10**) ($^{31}P\{H\}$, CD_2Cl_2, 202 MHz)

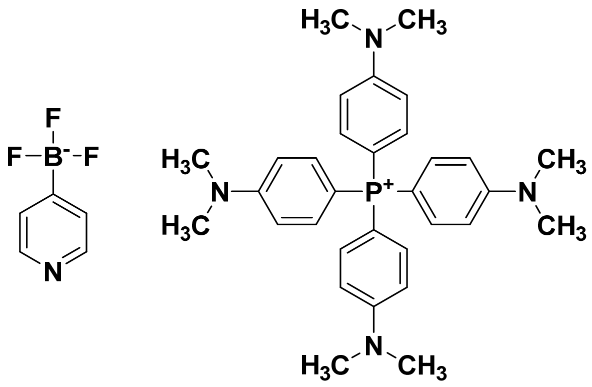

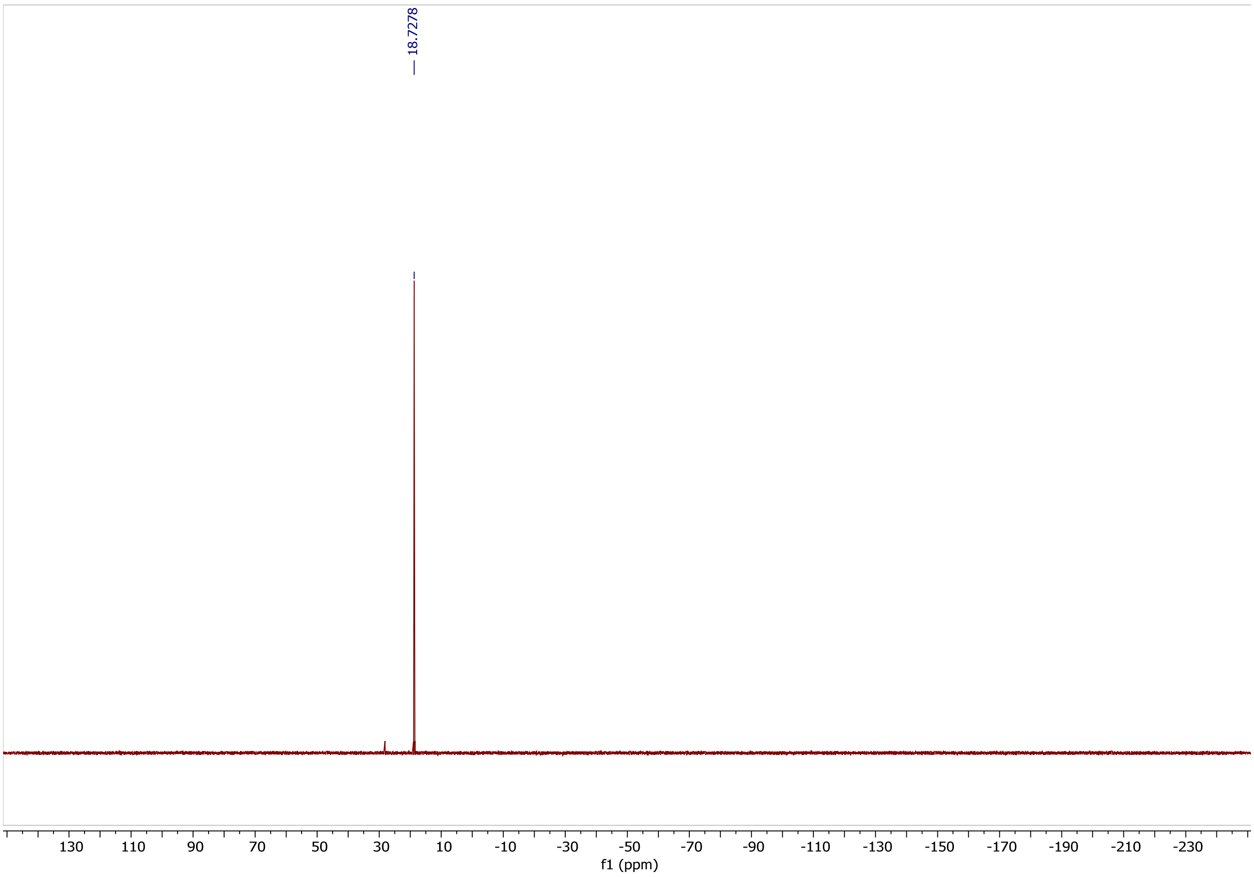

S3. Crystallographic and Refinement Details for Compounds 1-10 and 10h.

S3.1. General

In tables S5, S7, S9, S11, S13, S15, S17, S19, and S21 the reported uncertainties for contacts involving hydrogen atoms that are constrained as riding atoms are the uncertainties between the acceptor atom and the donor atom on which the hydrogen is riding.

S3.2. Refinement Details for 1.

Table S1 Crystallographic and refinement information for **1**.

CCDC number	2471465
Empirical formula	$C_5H_4BF_3KN$
Formula weight	185.00
Temperature [K]	100(2)
Crystal system, space group (number)	Orthorhombic, $P2_12_12_1$ (#19)
a, b, c [Å]	5.8637(2), 7.2260(3), 16.5602(8)
α, β, γ [°]	90, 90, 90
Volume [Å^3]	701.67(5)
Z	4
ρ_{calc} [gcm^{-3}], μ [mm^{-1}], *F*(000)	1.751, 0.736, 368
Crystal size [mm^3]	0.040×0.090×0.160
Crystal color, shape	Colorless, block
Radiation	MoK_α (λ=0.71073 Å)
2θ range [°]	4.92 to 56.54 (0.75 Å)
Reflections collected	11196
Independent reflections	1733, R_{int} = 0.0343, R_{sigma} = 0.0207
Completeness to θ = 25.242°	99.9 %
Data / Restraints / Parameters	1733 / 0 / 100
Absorption correction T_{min}/T_{max} (method)	0.6652 / 0.7457 (multi-scan)
Goodness-of-fit on F^2	0.992
Final *R* indexes [I≥2σ(I)]	R_1 = 0.0191, wR_2 = 0.0585
Final *R* indexes [all data]	R_1 = 0.0198, wR_2 = 0.0595
Largest peak/hole [eÅ^{-3}]	0.27/–0.17
Flack X parameter	0.008(16)

S3.3. Refinement Details for 2

Compound **2** crystallized as a pseudo-merohedral twin with a twin domain ratio of approximately 55:45. The following twin law was applied to the data: -1 0 0 0 1 0 0 0 1. The reflection 1 0 0 was omitted due to suspected interference from the beam stop. The pyridine ring is disordered and modeled in two equally occupied positions. Strong SAME restraints, RIGU restraints, and FLAT restraints were applied to both pyridine rings. To ensure the bond distances were in accordance, a SADI restraint was applied to C1, C5 and C1, C2 and a strong SADI restraint was C1', C5' and C1', C2'. Because of their proximity in space, the EADP constraint was applied to the following pairs of atoms: C1, C1'; N1, N1'; B1, B1'. All of the fluorine atoms were constrained using the EADP constraint. For the water molecule, a SADI restraint was applied to O1, H1O and O1, H2O to ensure similar bond distances.

Table S2 Crystallographic and refinement information for **2**.

CCDC number	2471466
Empirical formula	$C_5H_6BF_3KNO$
Formula weight	203.02
Temperature [K]	100(2)
Crystal system, space group (number)	Monoclinic, $P2_1/c$ (#14)
a, b, c [Å]	10.8271(15), 8.5041(11), 8.8435(12)
α, β, γ [°]	90, 90.043(5), 90
Volume [$Å^3$]	814.26(19)
Z	4
ρ_{calc} [gcm^{-3}], μ [mm^{-1}], $F(000)$	1.656, 0.651, 408
Crystal size [mm^3]	0.070×0.170×0.170
Crystal color, shape	Colorless, plate
Radiation	MoK_{α} (λ=0.71073 Å)
2θ range [°]	4.61 to 53.40 (0.79 Å)
Reflections collected	19163
Independent reflections	1723, R_{int} = 0.0754, R_{sigma} = 0.0296
Completeness to θ = 25.242°	99.9 %
Data / Restraints / Parameters	1723 / 176 / 158
Absorption correction T_{min}/T_{max} (method)	0.5790 / 0.7454 (multi-scan)
Goodness-of-fit on F^2	1.111
Final *R* indexes [$I \geq 2\sigma(I)$]	R_1 = 0.0321, wR_2 = 0.0817
Final *R* indexes [all data]	R_1 = 0.0405, wR_2 = 0.0850
Largest peak/hole [$eÅ^{-3}$]	0.47/–0.32

Table S3 Selected close contacts in **2**.

Contact (donor-H···acceptor)	Distance (H···acceptor) (Å)	Angle (donor-H···acceptor) (°)	Symmetry operator (applied to the donor atom)
O1-H2O···N1	1.814(30)	166.11	1-x, 1/2+y, 1/2-z
O1-H2O···N1’	1.848(30)	162.49	1-x, 1/2+y, 1/2-z

S3.4. Refinement Details for 3.

Table S4 Crystallographic and refinement information for **3**.

CCDC number	2471467
Empirical formula	$C_{21}H_{40}BF_3N_2$
Formula weight	388.36
Temperature [K]	100(2)
Crystal system, space group (number)	Monoclinic, $P2_1/n$ (#14)
a, b, c [Å]	9.4405(7), 15.9663(10), 15.4847(11)
α, β, γ [°]	90, 100.159(2), 90
Volume [Å^3]	2297.4(3)
Z	4
ρ_{calc} [gcm^{-3}], μ [mm^{-1}], F(000)	1.123, 0.081, 848
Crystal size [mm^3]	0.060×0.060×0.320
Crystal color, shape	Colorless, needle
Radiation	MoK_α (λ=0.71073 Å)
2θ range [°]	4.71 to 50.96 (0.83 Å)
Reflections collected	23792
Independent reflections	4228, R_{int} = 0.0709, R_{sigma} = 0.0564
Completeness to θ = 25.242°	100.0 %
Data / Restraints / Parameters	4228 / 0 / 248
Absorption correction T_{min}/T_{max} (method)	0.6624 / 0.7452 (multi-scan)
Goodness-of-fit on F^2	1.025
Final *R* indexes [I≥2σ(I)]	R_1 = 0.0433, wR_2 = 0.0920
Final *R* indexes [all data]	R_1 = 0.0700, wR_2 = 0.1057
Largest peak/hole [eÅ^{-3}]	0.20/−0.19

Table S5 Selected close contacts in **3**.

Contact (donor-H···acceptor)	Distance (H···acceptor) (Å)	Angle (donor-H···acceptor) (°)	Symmetry operator (applied to the donor atom)
C6-H6B···F2	2.312(2)	136.10	x, y, z
C18-H18A···F1	2.316(2)	152.01	1/2+x, 3/2-y, 1/2+z
C8-H8B···F2	2.372(2)	129.87	x, y, z
C5-H5···F3	2.430(2)	142.67	2-x, 2-y, 2-z
C10-H10A···F3	2.576(2)	149.36	1/2-x, 1/2+y, 3/2-z
C14-H14B···N1	2.583(2)	144.60	1+x, y, z
C6-H6A···N1	2.609(3)	144.22	1+x, y, z

S3.5. Refinement Details for 4.

Table S6 Crystallographic and refinement information for **4**.

CCDC number	2471468
Empirical formula	$C_{21}H_{40}BF_3N_2$
Formula weight	388.36
Temperature [K]	100(2)
Crystal system, space group (number)	Monoclinic, $P2_1/c$ (#14)
a, b, c [Å]	8.8089(6), 13.2030(8), 19.9075(14)
α, β, γ [°]	90, 98.652(2), 90
Volume [Å^3]	2289.0(3)
Z	4
ρ_{calc} [gcm^{-3}], μ [mm^{-1}], *F*(000)	1.127, 0.082, 848
Crystal size [mm^3]	0.060×0.070×0.080
Crystal color, shape	Colorless, block
Radiation	MoK_α (λ=0.71073 Å)
2θ range [°]	4.14 to 50.78 (0.83 Å)
Reflections collected	24696
Independent reflections	4206, R_{int} = 0.0593, R_{sigma} = 0.0408
Completeness to θ = 25.242°	100.0 %
Data / Restraints / Parameters	4206 / 0 / 248
Absorption correction T_{min}/T_{max} (method)	0.7047 / 0.7452 (multi-scan)
Goodness-of-fit on F^2	1.025
Final *R* indexes [$I \geq 2\sigma(I)$]	R_1 = 0.0400, wR_2 = 0.0864
Final *R* indexes [all data]	R_1 = 0.0667, wR_2 = 0.0978
Largest peak/hole [eÅ^{-3}]	0.19/−0.20

Table S7 Selected close contacts in **4**.

Contact (donor-H···acceptor)	Distance (H···acceptor) (Å)	Angle (donor-H···acceptor) (°)	Symmetry operator (applied to the donor atom)
C6-H6B···F2	2.279(2)	147.32	x, y, z
C10-H10···F3	2.313(2)	154.70	2-x, 1/2+y, 3/2-z
C18-H18B···F1	2.366(2)	147.02	2-x, 1/2+y, 3/2-z
C11-H11A···F1	2.369(2)	166.76	x, y, z
C19-H19A···F2	2.380(2)	147.17	1-x, 1/2+y, 3/2-z
C18-H18B···F3	2.454(2)	156.07	2-x, 1/2+y, 3/2-z
C16-H16A···F3	2.472(2)	138.46	1-x, 1/2+y, 3/2-z
C20-H20A···F1	2.494(2)	143.19	2-x, 1/2+y, 3/2-z
C17-H17B···N1	2.654(2)	174.95	x, 1+y, z

S3.6. Refinement Details for 5.

Table S8 Crystallographic and refinement information for **5**.

CCDC number	2471469
Empirical formula	$C_{29}H_{24}BF_3NP$
Formula weight	485.27
Temperature [K]	126(2)
Crystal system, space group (number)	Monoclinic, *Pc* (#7)
a, b, c [Å]	10.1711(2), 9.3254(2), 13.7076(3)
α, β, γ [°]	90, 108.7010(10), 90
Volume [Å^3]	1231.52(5)
Z	2
ρ_{calc} [gcm^{-3}], μ [mm^{-1}], F(000)	1.309, 1.334, 504
Crystal size [mm^3]	0.240×0.250×0.250
Crystal color	White, block
Radiation	CuK_α (λ=1.54178 Å)
2θ range [°]	9.18 to 149.26 (0.80 Å)
Reflections collected	24137
Independent reflections	4783, R_{int} = 0.0351, R_{sigma} = 0.0270
Completeness to θ = 67.679°	99.5 %
Data / Restraints / Parameters	4783 / 2 / 316
Absorption correction T_{min}/T_{max} (method)	0.6609 / 0.7538 (multi-scan)
Goodness-of-fit on F^2	1.021
Final *R* indexes [I≥2σ(I)]	R_1 = 0.0291, wR_2 = 0.0759
Final *R* indexes [all data]	R_1 = 0.0295, wR_2 = 0.0764
Largest peak/hole [eÅ^{-3}]	0.25/−0.26
Flack X parameter	-0.011(12)

Table S9 Selected close contacts in **5**.

Contact (donor-H···acceptor)	Distance (H···acceptor) (Å)	Angle (donor-H···acceptor) (°)	Symmetry operator (applied to the donor atom)
C25-H25···F3	2.122(2)	150.53	1+x, 1-y, 1/2+z
C9-H9···F1	2.184(3)	137.34	x, 1-y, -1/2+z
C10-H3···F3	2.196(3)	167.07	x, y, z
C16-H16···F2	2.244(3)	159.00	1+x, 1+y, z
C11-H11···F1	2.351(3)	127.20	x, y, z
C23-H23···F3	2.377(3)	147.62	1+x, 1-y, 1/2+z
C14-H14···F1	2.393(3)	133.00	1+x, y, z
C13-H13···F2	2.446(2)	157.31	1+x, y, z
C26-H26···F2	2.480(3)	141.59	1+x, 1-y, 1/2+z
C27-H27···N1	2.546(4)	154.95	x, 1-y, 1/2+z
C29-H29···N1	2.591(3)	118.67	x, 1+y, z
C20-H20···N1	2.699(3)	145.23	x, y, z
C15-H15···N1	2.842(4)	167.49	1+x, 1+y, z

S3.7. Refinement Details for 6.

Table S10 Crystallographic and refinement information for **6**.

CCDC number	2471470
Empirical formula	$C_{29}H_{24}BF_3NP$
Formula weight	485.27
Temperature [K]	125(2)
Crystal system, space group (number)	Triclinic, *P*1 (#1)
a, b, c [Å]	9.4898(3), 10.2000(3), 14.4239(5)
α, β, γ [°]	99.787(2), 101.625(2), 113.231(2)
Volume [Å^3]	1207.43(7)
Z	2
ρ_{calc} [gcm^{-3}], μ [mm^{-1}], *F*(000)	1.335, 1.360, 504
Crystal size [mm^3]	0.060×0.070×0.100
Crystal color, shape	Colorless, block
Radiation	CuK_α (λ=1.54178 Å)
2θ range [°]	6.51 to 159.23 (0.78 Å)
Reflections collected	24637
Independent reflections	9470, R_{int} = 0.0344, R_{sigma} = 0.0389
Completeness to θ = 67.679°	99.3 %
Data / Restraints / Parameters	9470 / 3 / 631
Absorption correction T_{min}/T_{max} (method)	0.6047 / 0.7543 (multi-scan)
Goodness-of-fit on F^2	1.056
Final *R* indexes [$I \geq 2\sigma(I)$]	R_1 = 0.0381, wR_2 = 0.1054
Final *R* indexes [all data]	R_1 = 0.0388, wR_2 = 0.1061
Largest peak/hole [eÅ^{-3}]	0.34/−0.26
Flack X parameter	0.000(15)

Table S11 Selected close contacts for **6**.

Contact (donor-H···acceptor)	Distance (H···acceptor) (Å)	Angle (donor-H···acceptor) (°)	Symmetry operator (applied to the donor atom)
C19A-H19A···F3A	2.216(5)	146.30	-1+x, y, z
C16B-H16B···F1A	2.272(4)	160.54	-1+x, y, z
C14A-H14A···F1A	2.317(4)	163.05	-1+x, -1+y, z
C29A-H29A···F1A	2.344(4)	146.92	-1+x, y, z
C15A-H15A···F2A	2.389(5)	126.79	-1+x, -1+y, z
C22B-H22B···F2A	2.447(3)	119.95	x, y, z
C13B-H13B···F3A	2.481(3)	117.32	x, 1+y, z
C23B-H23B···F2A	2.556(4)	115.56	x, y, z
C21A-H21A···F2A	2.599(6)	127.35	x, y, z
C14B-H14B···F3A	2.626(4)	112.12	x, 1+y, z
C9A-H9A···N1A	2.494(5)	133.58	x, y, 1+z
C26A-H26A···N1A	2.566(5)	154.26	-1+x, y, 1+z
C19B-H19B···N1A	2.745(6)	111.49	-1+x, y, z
C20B-H20B···F1B	2.296(3)	157.51	-1+x, -1+y, -1+z
C9B-H9B···F3B	2.363(5)	132.08	-1+x, y, -1+z
C8A-H8A···F2B	2.413(4)	128.83	x, y, z
C28B-H28B···F1B	2.429(4)	132.01	x, y, -1+z
C27A-H27A···F2B	2.476(5)	156.39	-1+x, y, z
C11A-H11A···F3B	2.537(4)	154.14	-1+x, -1+y, z
C29B-H29B···F2B	2.538(4)	128.04	x, y, -1+z
C8B-H8B···F1B	2.620(3)	154.90	-1+x, y, -1+z
C3A-H3A···F1B	2.657(6)	156.23	x, -1+y, -1+z
C13A-H13A···N1B	2.463(7)	146.70	x, -1+y, z
C15B-H15B···N1B	2.780(6)	114.20	x, y, z

S3.8. Refinement Details for 7.

Table S12 Crystallographic and refinement information for **7**.

CCDC number	2471471
Empirical formula	$C_{37}H_{40}BF_3NO_8P$
Formula weight	725.48
Temperature [K]	150(2)
Crystal system, space group (number)	Monoclinic, $P2_1/n$ (#14)
a, b, c [Å]	21.3755(8), 7.5380(3), 23.0617(8)
α, β, γ [°]	90, 99.998(2), 90
Volume [$Å^3$]	3659.5(2)
Z	4
ρ_{calc} [gcm^{-3}], μ [mm^{-1}], $F(000)$	1.317, 0.142, 1520
Crystal size [mm^3]	0.050×0.070×0.310
Crystal color, shape	Colorless, needle
Radiation	MoK_α (λ=0.71073 Å)
2θ range [°]	4.80 to 56.56 (0.75 Å)
Reflections collected	43069
Independent reflections	9058, R_{int} = 0.0515, R_{sigma} = 0.0496
Completeness to θ = 25.242°	99.8 %
Data / Restraints / Parameters	9058 / 0 / 468
Absorption correction T_{min}/T_{max} (method)	0.6610 / 0.7461 (multi-scan)
Goodness-of-fit on F^2	1.020
Final *R* indexes [$I \geq 2\sigma(I)$]	R_1 = 0.0461, wR_2 = 0.1089
Final *R* indexes [all data]	R_1 = 0.0734, wR_2 = 0.1229
Largest peak/hole [$eÅ^{-3}$]	0.45/−0.35

Table S13 Selected close contacts for **7**.

Contact (donor-H···acceptor)	Distance (H···acceptor) (Å)	Angle (donor-H···acceptor) (°)	Symmetry operator (applied to the donor atom)
C18-H18···F3	2.287(2)	159.20	1/2+x, 3/2-y, 1/2+z
C34-H34···F2	2.292(2)	174.74	3/2-x, -1/2+y, 1/2-z
C37-H37B···F3	2.327(3)	163.07	3/2-x, -1/2+y, 1/2-z
C21-H21B···F3	2.356(2)	140.39	1/2+x, 3/2-y, 1/2+z
C13-H13A···F2	2.434(3)	127.96	x, 1+y, z
C3-H3···F3	2.480(3)	162.0	x, -1+y, z
C14-H14B···F1	2.508(3)	132.16	x, y, z
C22-H22C···F3	2.603(2)	119.69	1/2+x, 3/2-y, 1/2+z
C38-H38C···F2	2.627(3)	110.84	3/2-x, -1/2+y, 1/2-z
C26-H26···N1	2.250(3)	164.44	1-x, -y, 1-z
C29-H29B···N1	2.778(3)	115.11	1-x, -y, 1-z
C30-H30C···N1	2.836(3)	147.94	1/2+x, 1/2-y, 1/2+z

S3.9. Refinement Details for 8.

Compound **8** crystallized as a non-merohedral twin with a twin domain ratio of approximately 50:50. The twin law relating the domains is: -1 0 -0.004 -0.134 0.061 0.938 -0.130 1.062 -0.061. Anion B is disordered and modeled in two parts with an occupancy ratio of approximately 66:34. A strong SAME restraint was applied to the minorly occupied part to ensure geometrical accordance with the majorly occupied part. A FLAT restraint was applied to the pyridine portion of both disordered parts. Because of their proximity in space, the EADP constraint was applied to the following pairs of atoms: B1B, B1B'; F2B, F1B'; F1B, F3B'; F3B F2B'; C1B, C1B'; C2B, C2B'; C3B, C3B'; N1B, N1B'; C4B, C4B'; C5B, C5B'.

Table S14 Crystallographic and refinement information for **8**.

CCDC number	2471472
Empirical formula	$C_{37}H_{40}BF_3NO_8P$
Formula weight	725.48
Temperature [K]	150(2)
Crystal system, space group (number)	Triclinic, $P\overline{1}$ (#2)
a, b, c [Å]	14.702(4), 15.141(5), 16.017(5)
α, β, γ [°]	84.473(11), 88.059(10), 85.136(10)
Volume [Å^3]	3534.7(18)
Z	4
ρ_{calc} [gcm^{-3}], μ [mm^{-1}], F(000)	1.363, 0.147, 1520
Crystal size [mm^3]	0.050×0.070×0.160
Crystal color, shape	Colorless, needle
Radiation	MoK_α (λ=0.71073 Å)
2θ range [°]	3.72 to 50.50 (0.83 Å)
Reflections collected	12796
Independent reflections	12796, R_{sigma} = 0.0476
Completeness to θ = 25.242°	99.8 %
Data / Restraints / Parameters	12796 / 30 / 967
Absorption correction T_{min}/T_{max} (method)	0.6817 / 0.7452 (multi-scan)
Goodness-of-fit on F^2	1.064
Final *R* indexes [$I \geq 2\sigma(I)$]	R_1 = 0.0531, wR_2 = 0.1146
Final *R* indexes [all data]	R_1 = 0.0818, wR_2 = 0.1315
Largest peak/hole [eÅ^{-3}]	0.44/−0.34

Table S15 Selected close contacts for **8**.

Contact (donor-H···acceptor)	Distance (H···acceptor) (Å)	Angle (donor-H···acceptor) (°)	Symmetry operator (applied to the donor atom)
C36A-H36B···F2A	2.260(5)	172.83	1-x, 1-y, 2-z
C21A-H21B···F1A	2.289(4)	151.37	1-x, 1-y, 2-z
C33A-H33A···F3A	2.358(4)	170.67	x, -1+y, z
C20A-H20A···F2A	2.371(5)	131.18	x, y, z
C13A-H13C···F1A	2.458(5)	153.49	x, y, z
C36B-H36F···F3A	2.496(5)	147.18	1+x, -1+y, z
C36A-H36B···F3A	2.640(5)	132.36	1-x, 1-y, 2-z
C37A-H37B···F3A	2.644(5)	116.05	x, -1+y, z
C28A-H28C···N1A	2.797(5)	134.05	1-x, 1-y, 1-z
C21B-H21D···N1A	2.633(5)	116.23	x, y, z
C13B-H13E···N1A	2.855(5)	99.81	-x, 1-y, 1-z
C29B-H29E···F1B	2.210(10)	136.03	-x, 2-y, 1-z
C36B-H36E···F3B	2.284(8)	162.75	x, y, -1+z
C20B-H20F···F2B	2.309(7)	144.53	-x, 1-y, 1-z
C13B-H13F···F1B	2.350(9)	148.45	x, y, z
C37B-H37F···F2B	2.417(8)	160.75	x, y, -1+z
C17B-H17B···F2B	2.417(6)	145.18	-x, 1-y, 1-z
C21B-H21E···F2B	2.603(7)	131.28	-x, 1-y, 1-z
C33B-H33B···F3B	2.612(7)	126.78	x, y, -1+z
C9A-H9A···N1B	2.661(10)	166.10	1-x, 1-y, 1-z
C29A-H29A···N1B	2.741(20)	100.54	x, y, z
C37B-H37F···F3B’	2.299(10)	159.95	x, y, -1+z
C13B-H13F···F2B’	2.351(10)	155.84	x, y, z
C20B-H20F···F3B’	2.566(20)	143.23	-x, 1-y, 1-z
C29B-H29E···F2B’	2.604(20)	134.71	-x, 2-y, 1-z
C33B-H33B···F3B’	2.610(10)	116.74	x, y, -1+z

C9A-H9A···N1B’	2.411(10)	167.54	1-x, 1-y, 1-z
C13A-H13C···N1B’	2.666(40)	106.28	1-x, 1-y, 1-z
C13A-H13B···N1B’	2.687(40)	105.01	1-x, 1-y, 1-z
C29A-H29A···N1B’	2.813(40)	103.76	x, y, z

S3.10. Refinement Details for 9.

The anion is disordered and modeled in two parts with equal occupancy. SAME restraints were applied to both parts of the anion to ensure similar geometries. The RIGU restraint was applied to both parts of the anion. The SADI restraint was applied to the following sets of atoms: F1 F2, F2 F3, F2 F3, F3 F1; F1’ F2’, F2’ F3’, F2’ F3’, F3’ F1’; B1 F1, B1 F2, B1 F3; B1’ F1’, B1’ F2’, B1’ F3’. The EADP constraint was applied to the following pairs of atoms due to their proximity in space: C1, C1’; C2, C2’; C3, C3’; C4, C4’; C5, C5’; N1, N1’.

Table S16 Crystallographic and refinement information for **9**.

CCDC number	2471473
Empirical formula	$C_{37}H_{44}BF_3N_5P$
Formula weight	657.55
Temperature [K]	150(2)
Crystal system, space group (number)	Monoclinic, $P2_1/n$ (#14)
a, b, c [Å]	15.802(2), 12.0571(15), 18.121(2)
α, β, γ [°]	90, 94.405(3), 90
Volume [Å^3]	3442.4(8)
Z	4
ρ_{calc} [gcm^{-3}], μ [mm^{-1}], *F*(000)	1.269, 0.130, 1392
Crystal size [mm^3]	0.050×0.100×0.170
Crystal color, shape	Colorless, block
Radiation	MoK_α (λ=0.71073 Å)
2θ range [°]	4.06 to 50.70 (0.83 Å)
Reflections collected	31607
Independent reflections	6295, R_{int} = 0.0368, R_{sigma} = 0.0276
Completeness to θ = 25.242°	100.0 %
Data / Restraints / Parameters	6295 / 186 / 486
Absorption correction T_{min}/T_{max} (method)	0.6291 / 0.7456 (multi-scan)
Goodness-of-fit on F^2	1.023
Final *R* indexes [$I \geq 2\sigma(I)$]	R_1 = 0.0401, wR_2 = 0.0955
Final *R* indexes [all data]	R_1 = 0.0565, wR_2 = 0.1071
Largest peak/hole [eÅ^{-3}]	0.21/−0.30

Table S17 Selected close contacts for **9**.

Contact (donor-H···acceptor)	Distance (H···acceptor) (Å)	Angle (donor-H···acceptor) (°)	Symmetry operator (applied to the donor atom)
C28-H28A···F3	2.165(7)	165.56	1/2-x, 3/2+y, 3/2-z
C29-H29A···F3	2.272(7)	159.02	1/2-x, 3/2+y, 3/2-z
C27-H27···F2	2.362(4)	167.64	x, 1+y, z
C34-H34···F2	2.395(4)	164.77	1/2-x, 1/2+y, 3/2-z
C12-H12B···F1	2.423(4)	139.73	1-x, 1-y, 2-z
C20-H20B···F1	2.549(5)	141.43	-1/2+x, 1/2-y, 1/2+z
C21-H21B···N1	2.521(10)	124.21	3/2-x, 1/2-y, 3/2-z
C28-H28A···F3’	2.169(9)	173.33	1/2-x, 3/2+y, 3/2-z
C34-H34···F2’	2.320(6)	152.75	1/2-x, 1/2+y, 3/2-z
C29-H29A···F3’	2.522(9)	154.15	1/2-x, 3/2+y, 3/2-z
C13-H13C···F2’	2.527(5)	94.05	x, y, z
C13-H13A···F2’	2.560(5)	92.25	x, y, z
C35-H35···F1’	2.565(5)	140.80	1/2-x, 1/2+y, 3/2-z
C4-H4···F1’	2.629(50)	135.30	1-x, 2-y, 2-z
C21-H21B···N1’	2.808(20)	121.80	3/2-x, 1/2+y, 3/2-z

S3.11. Refinement Details for 10.

The anion is heavily disordered and modeled in three partially occupied parts with approximate occupancies of 43%, 33%, and 24%. Each partially occupied part of the anion is modeled using strong SAME restraints to ensure good accord between the geometries of each part. Strong RIGU restraints were applied to the BF_3 groups of each anion, and standard RIGU restraints were applied to the pyridine rings. SADI restraints were used on the BF_3 groups of each anion to ensure proper geometry. Because of their proximity in space, the EADP constraint was applied to the following sets of atoms: F2, F2'; B1, B1', B1''; C1, C1', C1''; C2, C2', C2''; C3, C3', C3''; C4, C4', C4''; C5. C5', C5''; N1, N1', N1''.

Table S18 Crystallographic and refinement information for **10**.

CCDC number	2498616
Empirical formula	$C_{37}H_{44}BF_3N_5P$
Formula weight	657.55
Temperature [K]	100(2)
Crystal system, space group (number)	Monoclinic, $P2_1/n$ (#14)
a, b, c [Å]	15.9231(9), 11.5958(6), 18.7330(12)
α, β, γ [°]	90, 96.696(2), 90
Volume [Å^3]	3435.3(3)
Z	4
ρ_{calc} [gcm^{-3}], μ [mm^{-1}], F(000)	1.271, 0.131, 1392
Crystal size [mm^3]	0.040×0.250×0.340
Crystal color, shape	Colorless, plate
Radiation	MoK_α (λ=0.71073 Å)
2θ range [°]	4.14 to 51.41 (0.82 Å)
Reflections collected	52555
Independent reflections	6526, R_{int} = 0.0596, R_{sigma} = 0.0360
Completeness to θ = 25.242°	100.0 %
Data / Restraints / Parameters	6526 / 367 / 525
Absorption correction T_{min}/T_{max} (method)	0.6294 / 0.7453 (multi-scan)
Goodness-of-fit on F^2	1.035
Final *R* indexes, [I≥2σ(I)]	R_1 = 0.0513, wR_2 = 0.1232
Final *R* indexes, [all data]	R_1 = 0.0780, wR_2 = 0.1415
Largest peak/hole [eÅ^{-3}]	0.95/–0.35

Table S19 Selected close contacts for **10**.

Contact (donor-H···acceptor)	Distance (H···acceptor) (Å)	Angle (donor-H···acceptor) (°)	Symmetry operator (applied to the donor atom)
C21-H21A···F1″	2.097(8)	162.6	3/2-x, -3/2+y, 3/2-z
C16-H16···F1	2.191(10)	151.6	x, -1+y, z
C20-H20A···F2	2.207(10)	161.1	3/2-x, -3/2+y, 3/2-z
C20-H20A···F2′	2.211(10)	159.0	3/2-x, -3/2+y, 3/2-z
C37-H37B···F3	2.268(10)	139.9	1/2+x, 3/2-y, 1/2+z
C16-H16···F3″	2.303(10)	149.6	x, -1+y, z
C20-H20A-···F1″	2.305(10)	146.9	3/2-x, -3/2+y, 3/2-z
C12-H12C···F1	2.363(10)	113.3	x,y,z
C23-H23···F3″	2.371(8)	147.4	3/2-x, -1/2+y, 3/2-z
C21-H21A···F2	2.375(10)	158.2	3/2-x, -3/2+y, 3/2-z
C37-H37B···F2″	2.381(9)	131.6	1/2+x, 3/2-y, 1/2+z
C24-H24···F1′	2.381(8)	170.6	3/2-x, -1/2+y, 3/2-z
C12-H12C···F3′	2.388(8)	99.3	x,y,z
C15-H15···F2′	2.400(10)	126.3	x, -1+y, z
C21-H21A···F2′	2.417(10)	157.2	3/2-x, -3/2+y, 3/2-z
C13-H13B···F2″	2.494(10)	138.5	1-x, 1-y, 2-z
C21-H21A···F1′	2.530(10)	148.2	3/2-x, -3/2+y, 3/2-z
C15-H15···F1″	2.532(10)	138.0	x, -1+y, z
C24-H24···F2	2.579(10)	118.4	3/2-x, -1/2+y, 3/2-z
C28-H28B···F1′	2.604(8)	144.5	3/2-x, -1/2+y, 3/2-z
C37-H37B···F3′	2.607(10)	144.5	1/2+x, 3/2-y, 1/2+z
C24-H24···F3	2.610(10)	133.6	3/2-x, -1/2+y, 3/2-z
C13-H13B-F3	2.614(10)	143.2	1-x, 1-y, 2-z
C12-H12C···F3″	2.623(9)	107.9	x,y,z
C12-H12A···F3′	2.662(8)	84.6	x,y,z
C32-H32···N1″	2.677(8)	159.1	1/2-x, -1/2+y, 3/2-z

C28-H28A···N1′	2.744(20)	103.0	-1/2+x, 1/2-y, 1/2+z

S3.12. Refinement Details for 10h.

Table S20 Crystallographic information for **10h**.

CCDC number	2471474
Empirical formula	$C_{37}H_{45}BF_3N_5O_{0.50}P$
Formula weight	666.56
Temperature [K]	150(2)
Crystal system, space group (number)	Monoclinic, $P2_1/n$ (#14)
a, b, c [Å]	11.9373(3), 23.4315(7), 13.0949(3)
α, β, γ [°]	90, 105.9380(10), 90
Volume [Å^3]	3521.96(16)
Z	4
ρ_{calc} [gcm^{-3}], μ [mm^{-1}], F(000)	1.257, 0.129, 1412
Crystal size [mm^3]	0.100×0.160×0.160
Crystal color, shape	Colorless, block
Radiation	MoK_α (λ=0.71073 Å)
2θ range [°]	3.95 to 52.77 (0.80 Å)
Reflections collected	31433
Independent reflections	7198, R_{int} = 0.0324, R_{sigma} = 0.0285
Completeness to θ = 25.242°	100.0 %
Data / Restraints / Parameters	7198 / 4 / 448
Absorption correction T_{min}/T_{max} (method)	0.6444 / 0.7454 (multi-scan)
Goodness-of-fit on F^2	1.036
Final *R* indexes [$I \geq 2\sigma(I)$]	R_1 = 0.0438, wR_2 = 0.1075
Final *R* indexes [all data]	R_1 = 0.0576, wR_2 = 0.1169
Largest peak/hole [eÅ^{-3}]	0.55/−0.46

Table S21 Selected close contacts for **10h**.

Contact (donor-H···acceptor)	Distance (H···acceptor) (Å)	Angle (donor-H···acceptor) (°)	Symmetry operator (applied to the donor atom)
C29-H29A···F3	2.308(2)	151.90	x, y, 1+z
C28-H28A···F3	2.334(2)	146.03	x, y, 1+z
C20-H20A···F1	2.394(3)	150.44	-x, 1-y, 1-z
C28-H28B···F2	2.421(2)	154.26	-1/2+x, 3/2-y, 1/2+z
C24-H24···F2	2.477(2)	129.02	-1/2+x, 3/2-y, 1/2+z
C7-H7···F1	2.484(2)	120.15	x, y, z
C36-H36B···F2	2.572(2)	153.89	1/2-x, 1/2+y, 1/2-z
C8-H8···F1	2.585(2)	116.08	x, y, z
C24-H24···F3	2.608(2)	122.22	-1/2+x, 3/2-y, 1/2+z
C12-H12B···F2	2.638(3)	113.58	-1/2+x, 3/2-y, -1/2+z
C37B-H37B···N1	2.595(4)	140.57	-1/2+x, 3/2-y, 1/2+z
O1-H1O···N1[a]	2.019(30)	137.65	x, y, z

S4. Reaction Kinetics

S4.1. General Procedure for S_N2 Kinetics

Data was collected using a Bruker Avance 400 MHz NMR spectrometer. All salts were dried under vacuum (10^{-3} torr) and heat (50 °C) for at least 24 hours before use. 1-Iodooctane was dried on 3 Å molecular sieves before use. Before running a kinetics experiment, the sample temperature of the NMR spectrometer was determined using a pure methanol standard (Van Geet, 1970). All kinetics experiments were conducted in a temperature range of 298.0 – 298.6 K. In a nitrogen atmosphere glovebox, an amount of each salt to make a 25 mM solution and iodooctane to make a 500 mM solution were dissolved in deuterated dichloromethane and placed in a screw cap NMR tube. The tube was inverted several times to ensure mixing. The reaction solution was then placed in the spectrometer and ^{1}H-NMR spectra were taken at intervals ranging from 10 to 20 minutes. Data were processed using the Reaction Monitoring program within the MNova software package (Mestrelab Research, 2023). The pseudo-first order rate constant for DMAP and salts **3-10** were determined by fitting the integrated area of the product peak at δ ~ 4.2 to 4.5 ppm (depending on the salt being monitored) as a function of time to Equation 1, where B, F, and G were treated as variables; G is the rate constant. The value of G obtained by the program is in s^{-1}, this value is multiplied by 60 to get the unit of min^{-1}.

$$A_t = B + Fe^{-Gt} \qquad (1)$$

In the case of pyridine, a non-linear fit of the data proved inappropriate. Instead, the data set was fit by plotting the natural logarithm of the product peak integral area (at ~ 4.9 ppm) versus time. The slope of the linear fit to this data set corresponds to the rate constant.

S4.2. Kinetics Data for Pyridine

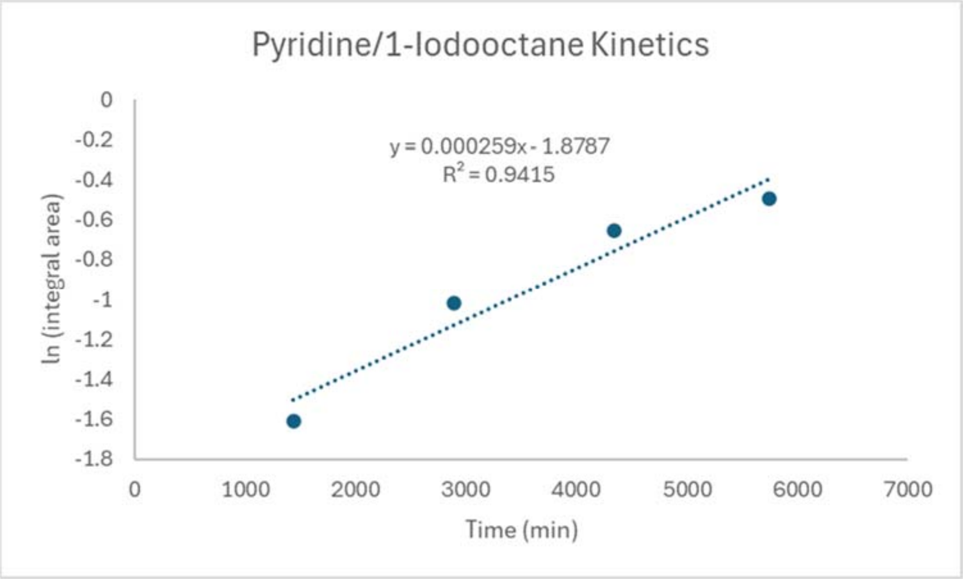

Figure S1 The kinetics data (Y, crosses) and fitted equation for pyridine. The slope of the equation is the rate constant for the reaction.

Table S22 Kinetics Data for pyridine.

[pyridine] 24.8 mM	[iodooctane] 509.6 mM	Volume CD_2Cl_2: 500 µL
Time (min)	Integral Area (a.u.)	ln (Integral Area)
1440.83	1885.39	-1.609
2899.07	2976.67	-1.022
4345.43	4773.6	-0.654
5751.77	6184.47	-0.494

Mass pyridine = 0.98 mg, mass iodooctane = 61.2 mg

S4.3. Kinetics Data for DMAP

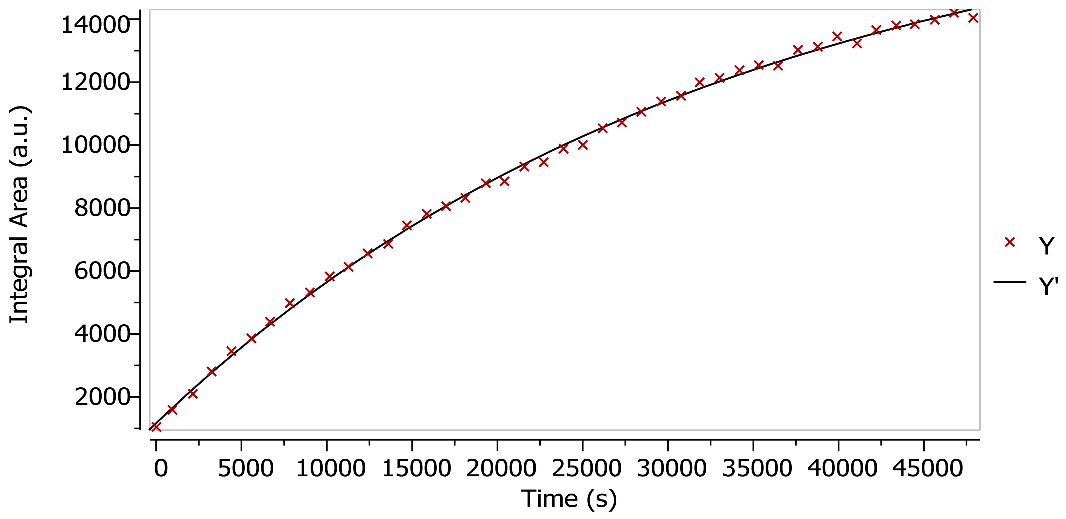

Figure S2 The kinetics data (Y, crosses) and fitted data (Y', line) for DMAP (Run 1).

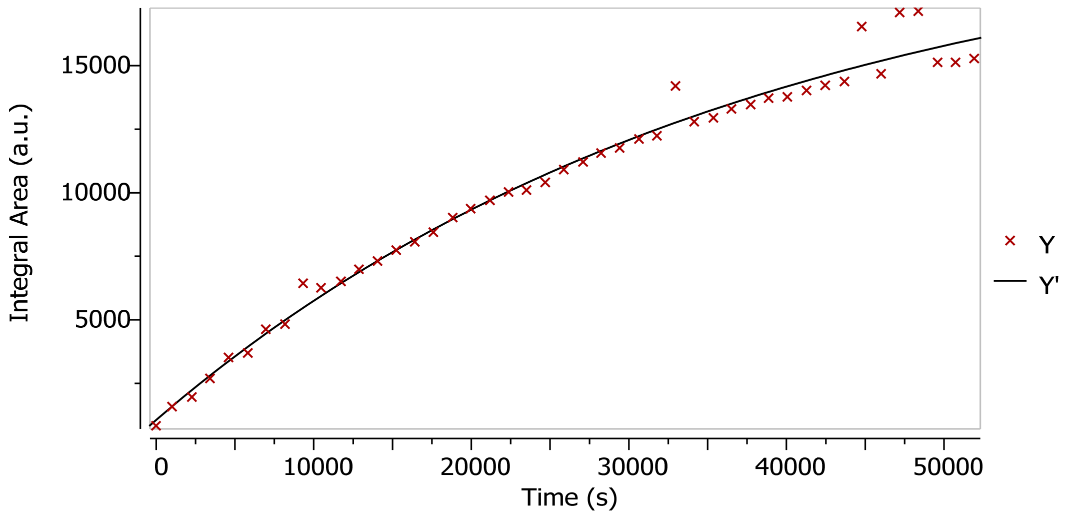

Figure S3 The kinetics data (Y, crosses) and fitted data (Y', line) for DMAP (Run 1).

Table S23 Kinetics data for DMAP.

Run 1			Run 2		
[DMAP] 24.6 mM	[iodooctane] 509.6 mM	Volume CD_2Cl_2: 1000 µL	[DMAP] 24.6 mM	[iodooctane] 509.6 mM	Volume CD_2Cl_2: 1000 µL
B= 18390	F= -17220	G= 0.00003012	B= 21000	F= -19950	G= 0.00002680
Time (s)	Integral Area (a.u.)	B+F*exp(-t*G)	Time (s)	Integral Area (a.u.)	B+F*exp(-t*G)
0	1046.93	1166.72	0	828.431	1056.58

966	1590.04	1660.65	1029	1584.94	1599.23
2140	2098.5	2241.9	2260	1971.11	2229.04
3256	2809.03	2775.7	3405	2698.44	2796.49
4403	3448.38	3305.95	4597	3510.14	3369.02
5573	3869.6	3828.29	5808	3687.49	3932.24
6682	4390.86	4306.68	6972	4627.15	4456.64
7845	4968.67	4791.5	8164	4838.47	4976.97
8999	5326.26	5256.07	9339	6422.85	5473.86
10173	5822.91	5712.4	10484	6256.47	5943.23
11271	6132.1	6124.84	11713	6519.72	6431.27
12415	6566.89	6540.29	12859	6991.62	6872.08
13598	6858.1	6955.11	14043	7305.79	7313.52
14697	7454.8	7327.45	15225	7744.73	7740.45
15852	7825.95	7705.71	16428	8063.52	8161.29
16987	8059.31	8064.82	17601	8450.44	8558.78
18133	8318.02	8415.16	18812	9026.76	8956.25
19314	8787.78	8763.77	19966	9367.48	9323.19
20422	8851.97	9079.75	21189	9706.13	9699.87
21577	9316.7	9398.1	22351	10038.3	10046.5
22703	9451.55	9697.97	23507	10108.7	10380.8
23867	9889.84	9997.46	24689	10418.8	10712.1
25021	9996.62	10284.2	25872	10919.4	11033.3
26166	10536	10559	27103	11202.3	11356.9
27301	10708.9	10822.2	28249	11561.1	11648.7
28437	11069.3	11076.8	29431	11773.2	11940.4
29582	11376.4	11324.7	30632	12109	12227.5
30773	11564	11573.7	31797	12231.8	12497.3
31863	11995.9	11793.9	32971	14203.3	12760.8
33026	12145.4	12021	34162	12794.9	13019.8
34180	12374.8	12238.6	35374	12947.7	13275

35307	12545.3	12443.9	36511	13296.3	13507
36443	12527.6	12643.9	37730	13467.4	13748
37615	13036.9	12843.3	38886	13724.4	13969.3
38798	13132.2	13037.5	40059	13766.8	14187
39897	13444.6	13211.8	41280	14019.3	14406.5
41078	13230.9	13392.7	42452	14222.4	14610.5
42195	13650.8	13558.1	43663	14381.2	14814.7
43372	13791.9	13726.4	44801	16550.2	15000.6
44467	13833.2	13877.8	45992	14682.7	15189.2
45649	13989	14035.6	47186	17084.1	15372.3
46748	14200.7	14177.4	48359	17144	15546.6
47911	14046.5	14322.4	49590	15121	15723.7
			50724	15133.6	15881.8
			51907	15284.8	16041.7

Run 1: Mass DMAP = 3.0 mg, mass iodooctane = 122.4 mg.

Run 2: Mass DMAP = 3.0 mg, mass iodooctane = 122.4 mg.

S4.4. Kinetics Data for 3.

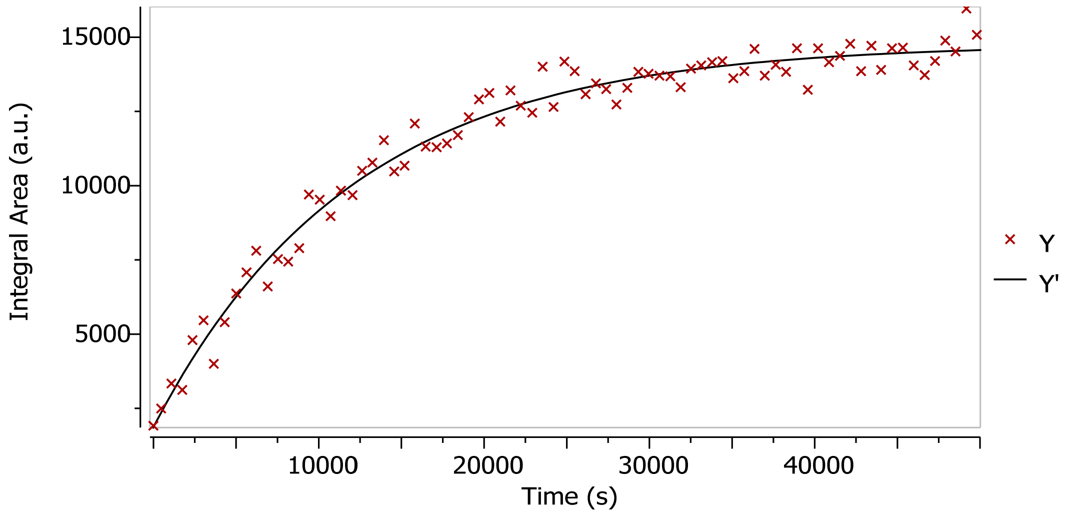

Figure S4 The kinetics data (Y, crosses) and fitted data (Y’, line) for **3** (Run 1).

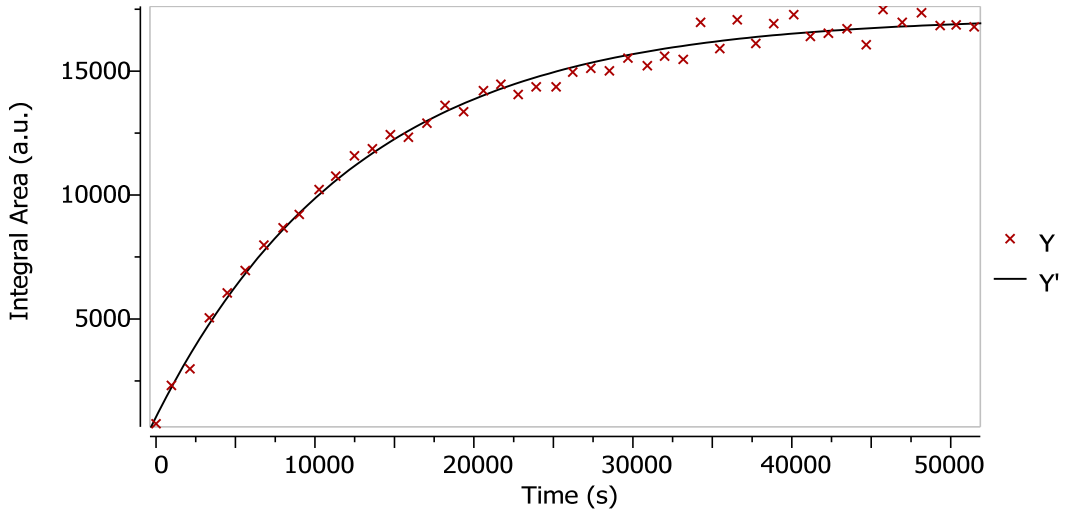

Figure S5 The kinetics data (Y, crosses) and fitted data (Y’, line) for **3** (Run 2).

Table S24 Kinetics data for **3**.

Run 1			Run 2		
[**3**]	[iodooctane]	Volume	[**3**]	[iodooctane]	Volume
24.7 mM	509.6 mM	CD_2Cl_2: 500 µL	24.7 mM	509.6 mM	CD_2Cl_2: 500 µL
B= 14790	F= -13450	G= 0.00008380	B= 17200	F= -16150	G= 0.0007896
Time (s)	Integral Area (a.u.)	B+F*exp(-t*G)	Time (s)	Integral Area (a.u.)	B+F*exp(-t*G)
0	1913.31	1874	0	766.56	1046.38
473	2502.83	2371.3	984	2329.04	2253.73
1084	3326.9	2985.41	2153	2988.4	3571.32
1736	3129.67	3607.21	3337	5055.5	4787.45
2378	4797.55	4187.39	4493	6057.4	5870.03
3017	5465.99	4734.91	5595	6955.22	6814
3650	4000.96	5249.35	6771	7993.04	7734.75
4299	5404.33	5749.41	7986	8685.23	8600.37
4997	6359.82	6257.94	9003	9225.41	9263.74
5617	7070.98	6685.54	10270	10211.8	10019
6213	7806.08	7076.33	11317	10759.4	10588.6
6910	6601.63	7509.42	12483	11587	11169.9
7523	7521.71	7870.12	13622	11866.6	11688.3

8146	7453.53	8218.34	14751	12424.5	12158.2
8797	7904.58	8563.44	15889	12340.3	12591.3
9410	9694.32	8871.76	17048	12889.1	12994.1
10051	9540.55	9177.77	18194	13615.8	13357.7
10700	8971.49	9471.43	19335	13363.2	13688.6
11352	9830.71	9750.91	20613	14209.3	14025.4
12029	9675.91	10025.5	21686	14466.6	14283.1
12642	10496.2	10261.2	22788	14069.3	14525.9
13255	10787.8	10485.1	23918	14360.5	14754
13960	11540.4	10728.9	25188	14374.5	14987.2
14583	10474.3	10932.8	26222	14958.2	15160.5
15205	10664.5	11126.1	27362	15105.8	15335.8
15818	12080.4	11307	28509	15014.8	15497
16468	11325.4	11489.1	29685	15533.5	15647.8
17119	11293.6	11661.8	30927	15218	15792.6
17753	11424.4	11821.3	31981	15603.2	15904.8
18401	11701	11975.8	33176	15481.3	16021.2
19052	12297.7	12122.9	34267	16974.8	16118.3
19674	12901.9	12256.2	35472	15907.5	16216.3
20306	13124.9	12384.7	36573	17058.4	16298
20956	12159.7	12510.1	37751	16125.1	16377.9
21588	13209.1	12625.6	38886	16921.9	16448.2
22237	12686.6	12738.1	40110	17289.5	16517.2
22925	12465.4	12851	41164	16389.2	16571.5
23520	14005.8	12943.5	42294	16533.4	16625
24189	12640.3	13042.1	43460	16716.8	16675.4
24848	14177.4	13134.1	44701	16070.8	16724.1
25481	13851.3	13217.8	45738	17487.5	16761.3
26147	13082.1	13301.3	46953	16978.6	16801.3
26752	13437.2	13373.2	48145	17353.7	16836.9

27403	13256.4	13446.7	49314	16831.8	16868.7
28008	12732.4	13511.4	50330	16864.5	16894
28684	13286.1	13580.1	51477	16774.8	16920.3
29335	13832.5	13642.6			
29930	13776	13696.9			
30627	13693.8	13757.1			
31258	13680.3	13808.8			
31901	13311	13858.6			
32503	13936.4	13903			
33126	14050.3	13946.6			
33793	14145.9	13990.9			
34408	14199.2	14029.6			
35085	13607	14069.9			
35745	13855.4	14107.2			
36349	14607.3	14139.5			
36972	13696	14171.1			
37631	14078.2	14202.9			
38264	13839.4	14231.8			
38904	14633	14259.6			
39591	13224	14287.7			
40205	14637	14311.6			
40863	14164.9	14335.8			
41515	14375.6	14358.6			
42156	14786.1	14379.8			
42806	13847.6	14400.1			
43418	14713.8	14418.3			
44023	13893	14435.4			
44682	14636.1	14453.1			
45323	14660.3	14469.4			
46000	14043.6	14485.7			

46643	13731.1	14500.3	
47256	14208.2	14513.6	
47878	14891.8	14526.4	
48520	14525.8	14538.9	
49169	15965.9	14550.8	
49801	15087.4	14561.9	

Run 1: Mass **3** = 4.8 mg, mass iodooctane = 61.2 mg.

Run 2: Mass **3** = 4.8 mg, mass iodooctane = 61.2 mg.

S4.5. Kinetics Data for 4.

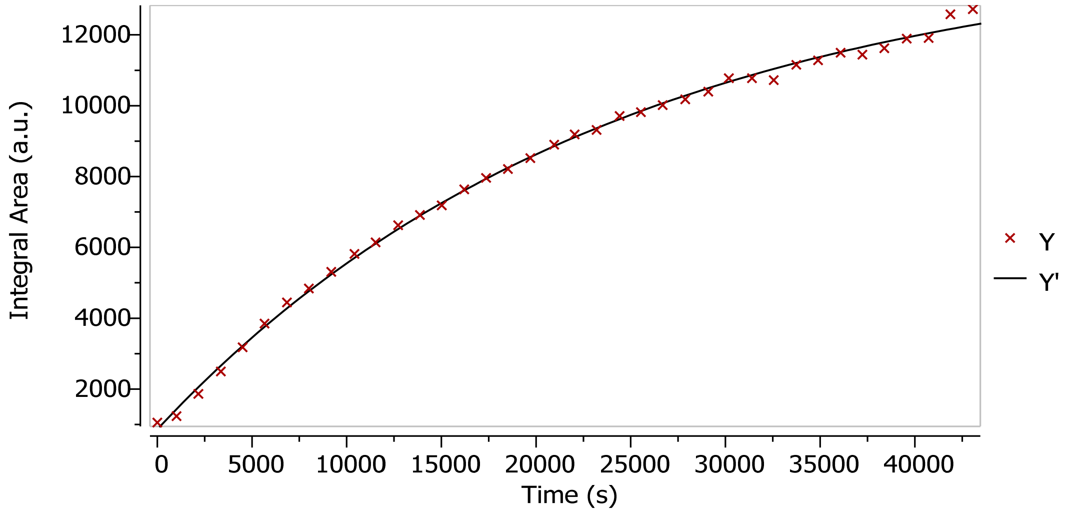

Figure S6 The kinetics data (Y, crosses) and fitted data (Y', line) for **4** (Run 1).

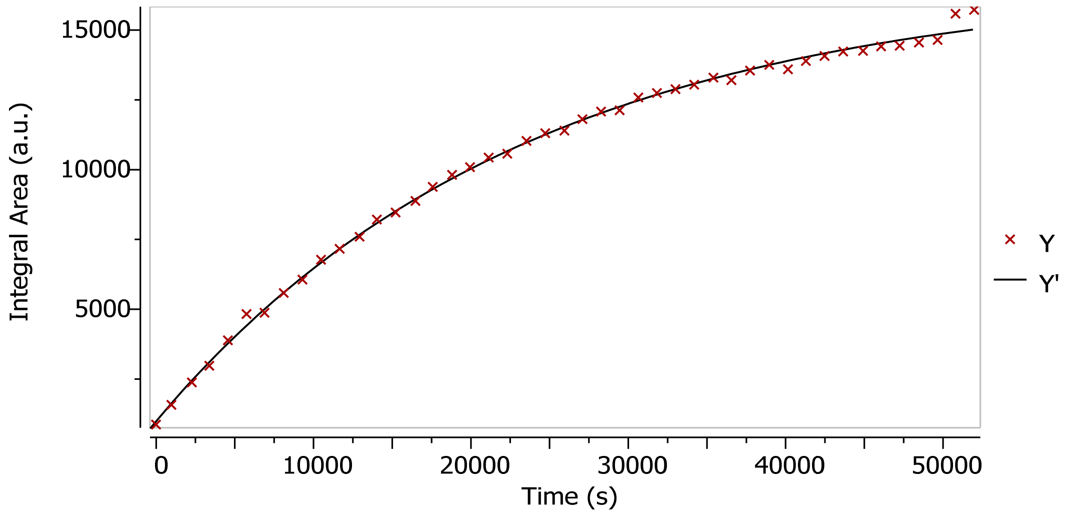

Figure S7 The kinetics data (Y, crosses) and fitted data (Y', line) for **4** (Run 2).

Table S25 Kinetics data for **4**.

Run 1			Run 2		
[**4**] 25.2 mM	[iodooctane] 509.6 mM	Volume CD_2Cl_2: 500 μL	[**4**] 25.2 mM	[iodooctane] 509.6 mM	Volume CD_2Cl_2: 500 μL
B= 14500	F= -13640	G= 0.00004211	B= 16730	F= -15750	G= 0.0004272
Time (s)	Integral Area (a.u.)	B+F*exp(-t*G)	Time (s)	Integral Area (a.u.)	B+F*exp(-t*G)
0	1049.79	861.26	0	863.775	986.307
1003	1240.27	1425.36	975	1564.05	1628.72
2180	1856.74	2057.64	2256	2381.8	2433.07
3357	2501.77	2659.35	3384	2972.07	3105.82
4507	3172.78	3219.14	4541	3889.36	3762.99
5674	3843.5	3760.17	5746	4829.91	4413.75
6843	4450.1	4276.11	6904	4882.92	5008.34
8011	4837.27	4766.86	8110	5579.81	5597.09
9179	5302.18	5234.06	9277	6064.12	6138.63
10402	5814.7	5699.24	10481	6774.63	6669.76
11523	6137.21	6105.09	11676	7169.68	7170.58
12729	6624.83	6520.85	12911	7596.11	7661.99
13860	6916	6892.02	14012	8204.45	8078.74
15037	7190.12	7259.97	15206	8471.75	8509.09
16195	7642.07	7604.62	16494	8869.78	8949.34
17354	7962.02	7933.14	17597	9392.71	9307.58
18514	8213.69	8246.27	18793	9808.78	9677.42
19691	8528.83	8548.74	19942	10086.7	10015.4
20941	8896.49	8853.97	21137	10441.8	10349.7
22026	9190.65	9106.19	22314	10565.6	10662.7
23204	9316.95	9367.3	23527	11020.5	10969.2
24427	9720.27	9625.02	24704	11315.2	11251.8
25531	9827.96	9846.53	25955	11401.8	11537

26698	10022.8	10069.8	27067	11796.6	11778
27867	10183.3	10282.6	28263	12091.6	12024.8
29099	10404.4	10495.9	29438	12129.8	12255.3
30194	10770.9	10676.4	30634	12581.9	12478.3
31398	10779.6	10865.5	31811	12753.8	12686.9
32557	10729.2	11038.7	33007	12876.7	12888.4
33725	11147.3	11204.9	34175	13042.4	13075.5
34893	11287.3	11363.1	35398	13291.3	13261.6
36071	11502.7	11515	36546	13206.7	13427.7
37219	11436.2	11656	37731	13544.9	13590.8
38379	11624.2	11791.6	38956	13746.1	13751
39564	11898.7	11923.5	40140	13601.3	13898.1
40734	11903.5	12047.5	41299	13894.3	14035
41873	12586.2	12162.4	42476	14071.9	14167.2
			43661	14223.5	14293.8
			44922	14262.3	14421.7
			46043	14416.1	14529.8
			47229	14448.5	14638.6
			48452	14555.8	14745.1
			49648	14639.1	14844.1
			50815	15589.2	14935.9
			51973	15724.3	15022.6

Run 1: Mass **4** = 4.9 mg, mass iodooctane = 61.2 mg.

Run 2: Mass **4** = 4.9 mg, mass iodooctane = 61.2 mg.

S4.6. Kinetics Data for 5.

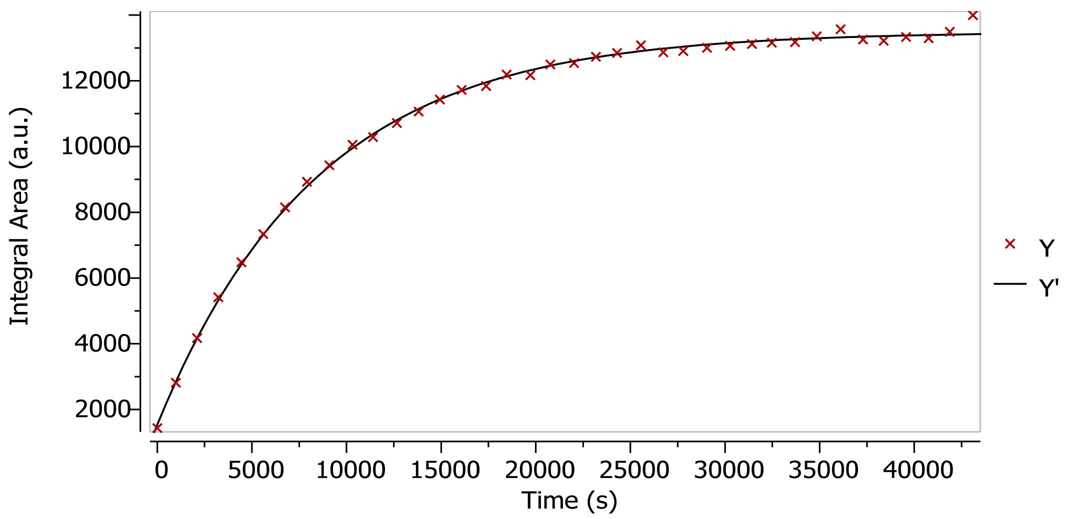

Figure S8 The kinetics data (Y, crosses) and fitted data (Y’, line) for **5** (Run 1).

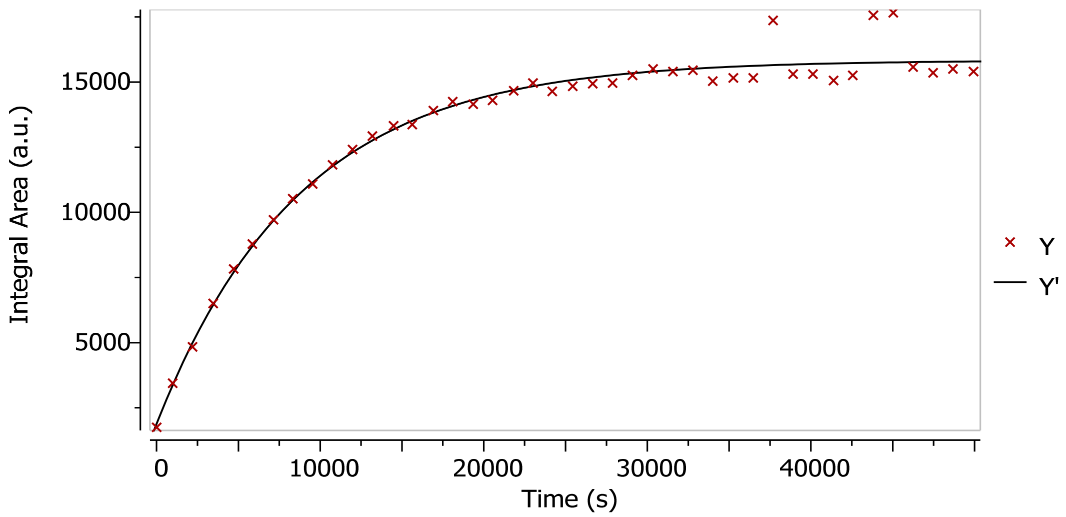

Figure S9 The kinetics data (Y, crosses) and fitted data (Y’, line) for **5** (Run 2).

Table S26 Kinetics data for **5**.

Run 1			Run 2		
[**5**]	[iodooctane]	Volume	[**5**]	[iodooctane]	Volume
25.1 mM	509.6 mM	CD_2Cl_2: 500 µL	25.1 mM	509.6 mM	CD_2Cl_2: 500 µL
B= 13490	F= -11950	G= 0.0001179	B= 15840	F= -13950	G= 0.0001146
Time (s)	Integral Area (a.u.)	B+F*exp(-t*G)	Time (s)	Integral Area (a.u.)	B+F*exp(-t*G)

0	1430.59	1541.61	0	1755.94	1885.85
976	2819.18	2840.75	971	3438.03	3355.35
2089	4161.92	4150.77	2213	4846.29	5011.61
3230	5416.75	5326.64	3463	6499.08	6456.7
4459	6474.28	6428.14	4696	7834.33	7693.02
5601	7330.74	7318.02	5854	8774.06	8705.34
6762	8155.33	8107.84	7154	9723.49	9692.65
7922	8924.21	8796.05	8318	10535.8	10460.1
9085	9427.74	9397.71	9533	11080.3	11159.1
10324	10059.9	9954.09	10764	11824.9	11774.7
11409	10295.2	10378.8	11979	12420.4	12302.8
12656	10722.5	10804.4	13201	12929.3	12764.7
13825	11054.2	11150.3	14470	13323.3	13180.6
14939	11427.2	11438.4	15639	13367.2	13513.7
16092	11724.8	11699.3	16935	13915.2	13834.4
17378	11832.2	11951.3	18093	14252.5	14083.3
18452	12184.5	12134.4	19374	14150	14322.8
19702	12171.7	12320.3	20531	14297.5	14510.8
20775	12493.1	12459.4	21820	14675.7	14693
22023	12537.2	12600.5	23013	14956.5	14839.2
23166	12725.5	12712.7	24199	14641.2	14966
24308	12842.3	12810.7	25431	14843.4	15080.6
25544	13080.2	12902.9	26673	14930.7	15181
26736	12877.1	12980	27869	14966.6	15265
27802	12912.8	13040.3	29099	15247.9	15340.2
29060	13003.5	13102.3	30361	15495.6	15407.1
30269	13057.4	13153.9	31573	15396.8	15462.8
31411	13115.3	13196.3	32789	15458.3	15511.5
32486	13161	13231.3	34000	15044.7	15553.6
33694	13188.7	13265.7	35271	15164	15592

34837	13348.7	13294.1	36457	15166	15623.1
36093	13562.1	13321.1	37689	17367.2	15651.3
37304	13256.2	13343.7	38911	15311	15675.5
38378	13212.5	13361.1	40107	15297.4	15696.2
39579	13339.6	13378.2	41396	15065.4	15715.5
40770	13292	13392.9	42571	15268	15730.8
41883	13491.7	13404.9	43805	17564	15744.7
			45045	17652.5	15756.9
			46241	15566.1	15767.2
			47472	15350.3	15776.3
			48678	15513.7	15784.1
			49946	15401.9	15791.2

Run 1: Mass **5** = 6.1 mg, mass iodooctane = 61.2 mg.

Run 2: Mass **5** = 6.1 mg, mass iodooctane = 61.2 mg.

S4.7. Kinetics Data for 6.

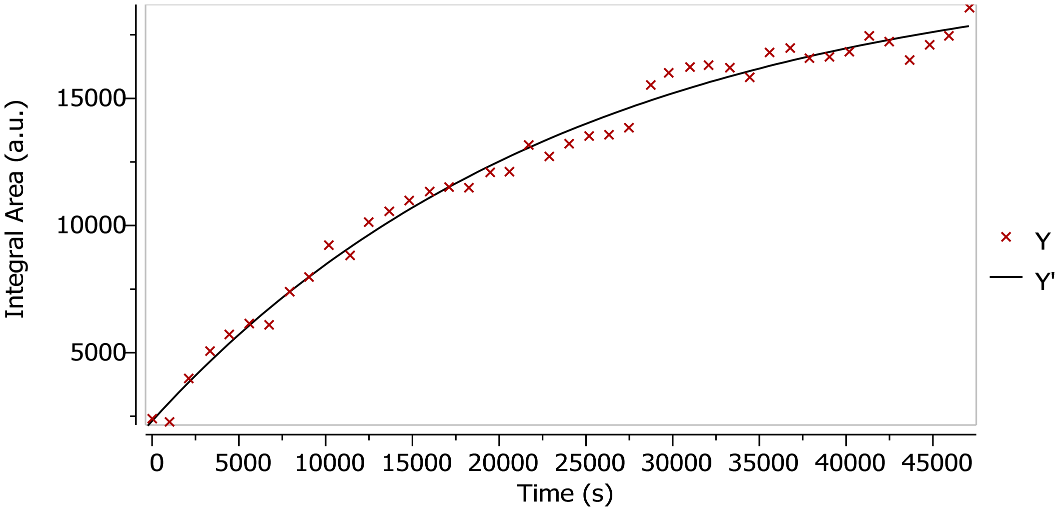

Figure S10 The kinetics data (Y, crosses) and fitted data (Y', line) for **6** (Run 1).

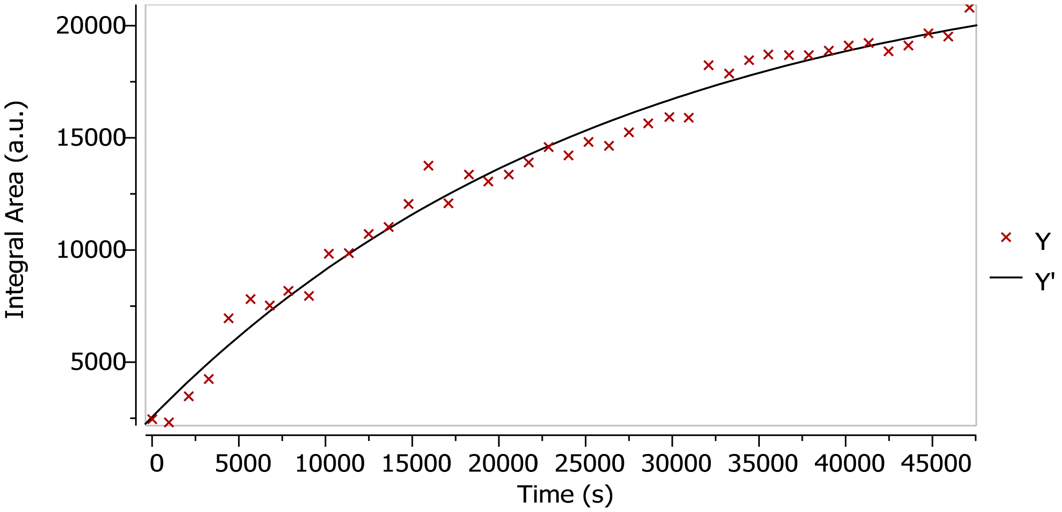

Figure S11 The kinetics data (Y, crosses) and fitted data (Y', line) for **6** (Run 2).

Table S27 Kinetics data for **6**.

Run 1			Run 2		
[**6**]	[iodooctane]	Volume	[**6**]	[iodooctane]	Volume
25.1 mM	509.6 mM	CD_2Cl_2: 500 µL	25.1 mM	509.6 mM	CD_2Cl_2: 500 µL
B= 20400	F= -18080	G= 0.00004154	B= 23560	F= -21000	G= 0.00003744
Time (s)	Integral Area (a.u.)	B+F*exp(-t*G)	Time (s)	Integral Area (a.u.)	B+F*exp(-t*G)
0	2394.62	2319.06	0	2467.33	2559.47

986	2284.68	3044.68	960	2319.74	3300.65
2120	3992.51	3843.29	2112	3469.18	4155.61
3349	5057.88	4667.37	3265	4256.44	4975.18
4436	5716.48	5361.99	4428	6956.42	5766.79
5588	6154.84	6064.7	5666	7805.74	6572.43
6751	6093.73	6740.82	6789	7530.83	7271.63
7922	7401.17	7389.38	7858	8170.58	7910.45
9056	7969.99	7988.08	9039	7949.86	8587.1
10192	9236.83	8560.22	10183	9821.23	9214.63
11391	8837.31	9135.51	11335	9858.02	9819.97
12497	10128.3	9641.36	12498	10710.8	10405.2
13660	10547.9	10148.8	13642	11031.7	10956.5
14831	10991.4	10635.6	14795	12048.7	11488.8
15985	11348.1	11092.6	15947	13766.9	11998.1
17117	11521.8	11520.2	17100	12073	12486.4
18272	11480.2	11936.2	18255	13365.7	12954.8
19499	12097.2	12356.9	19397	13058.1	13398.5
20578	12111.9	12709.4	20587	13355.6	13841.1
21721	13170.5	13066.1	21731	13911.7	14248.4
22893	12721.7	13414.6	22874	14589.7	14638.2
24046	13222	13741.4	24009	14212.9	15009.2
25172	13528	14045.7	25172	14820.5	15373.4
26325	13570.3	14342.9	26335	14642.6	15722
27488	13850.3	14628.6	27488	15246.1	16052.9
28734	15529.8	14919.8	28613	15630.6	16362.4
29783	16006.6	15153.5	29832	15938.9	16683.3
31001	16241.2	15412.4	30946	15909.7	16964
32089	16299.2	15632.8	32080	18243.1	17238
33288	16201	15864.5	33271	17869.1	17513.5
34434	15840.8	16075.4	34415	18463.2	17766.9

35595	16813.3	16279.1	35558	18712.7	18009.3
36758	16994.3	16473.5	36740	18679.3	18249.4
37901	16592.1	16655.6	37883	18701.6	18471.7
39017	16631.4	16825.3	39035	18877.7	18686.3
40179	16829.1	16993.8	40161	19111.6	18887.4
41331	17463.7	17153	41305	19233.5	19083.1
42495	17235.8	17306.3	42458	18850	19272.1
43655	16508.1	17451.9	43629	19114.6	19455.8
44801	17106.8	17589	44782	19655	19629
45934	17466.3	17718.3	45926	19517	19793.7
47114	18557.2	17846.6	47147	20788.1	19961.8

Run 1: Mass **6** = 6.1 mg, mass iodooctane = 61.2 mg.

Run 2: Mass **6** = 6.1 mg, mass iodooctane = 61.2 mg.

S4.8. Kinetics Data for 7.

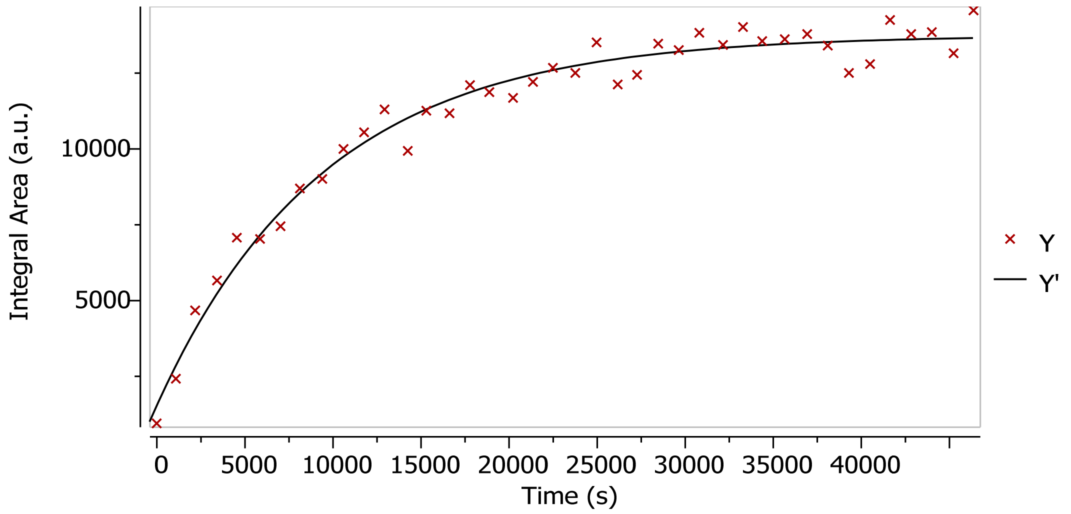

Figure S12 The kinetics data (Y, crosses) and fitted data (Y’, line) for **7** (Run 1).

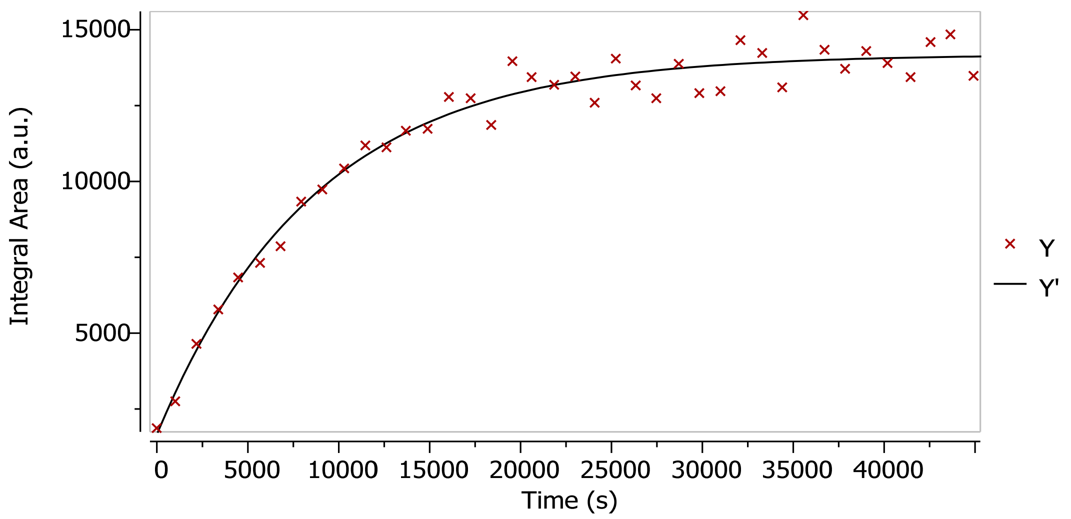

Figure S13 The kinetics data (Y, crosses) and fitted data (Y', line) for **7** (Run 2).

Table S28 Kinetics data for **7**.

Run 1			Run 2		
[7] 24.8 mM	[iodooctane] 509.6 mM	Volume CD_2Cl_2: 500 µL	[7] 25.1 mM	[iodooctane] 509.6 mM	Volume CD_2Cl_2: 500 µL
B= 13750	F= -12190	G= 0.0001051	B= 14190	F= -12520	G= 0.0001152
Time (s)	Integral Area (a.u.)	B+F*exp(-t*G)	Time (s)	Integral Area (a.u.)	B+F*exp(-t*G)
0	939.875	1553.97	0	1860.75	1666.26
1062	2417.65	2841.69	1025	2761.13	3060.63
2170	4683.12	4040.46	2157	4649.67	4420.82
3403	5663.51	5220.21	3369	5776.27	5693.33
4558	7065.84	6194.93	4481	6837.16	6714.44
5859	7020.96	7160.08	5654	7319.02	7658.8
7032	7448.09	7924.08	6805	7871.78	8469.33
8122	8691.49	8554.36	7918	9341.19	9157.24
9401	9015.2	9207.51	9079	9728.48	9786.76
10606	9998.53	9747.52	10301	10437	10364.4
11769	10537.3	10207.7	11471	11179	10846.3
12944	11305.9	10618.9	12613	11114.8	11257.9
14227	9930.48	11013.5	13678	11670.2	11596

15294	11271.4	11303.5	14899	11733.3	11935.8
16605	11184.1	11618.1	16050	12775.2	12215.2
17787	12096.1	11866.8	17253	12752.2	12470.2
18876	11881.7	12070.2	18384	11856	12679.8
20206	11674	12288.9	19556	13961.6	12870
21351	12217.9	12454.3	20602	13437.9	13019.4
22469	12670	12597.6	21860	13182	13176.7
23748	12495.2	12742.2	23014	13465.3	13302.3
24962	13521.8	12862.6	24067	12601.5	13403.3
26168	12120.9	12967.9	25250	14047	13502.9
27254	12432.1	13052	26330	13154	13582.8
28477	13470.5	13135.9	27474	12745.7	13657.3
29614	13265.5	13204.7	28692	13885	13726.5
30808	13832.2	13268.7	29815	12900.3	13782.2
32146	13437.1	13331.5	31009	12983.4	13834.1
33262	14024.2	13377.5	32081	14651.3	13874.9
34390	13551.2	13418.9	33301	14231.4	13915.7
35632	13615.1	13459.1	34404	13094.1	13947.9
36922	13795.4	13495.6	35557	15485.5	13977.5
38075	13401.1	13524.3	36719	14332.9	14003.6
39280	12494.2	13550.9	37842	13714.9	14025.7
40483	12792.4	13574.2	39012	14300.6	14045.9
41630	14241.7	13593.9	40175	13891.5	14063.4
42834	13788.9	13612.1	41443	13445.7	14080
44018	13851.3	13627.9	42529	14592.2	14092.5
45231	13161.6	13642.2			

Run 1: Mass **7** = 9.0 mg, mass iodooctane = 61.2 mg.

Run 2: Mass **7** = 9.1 mg, mass iodooctane = 61.2 mg.

S4.9. Kinetics data for 8.

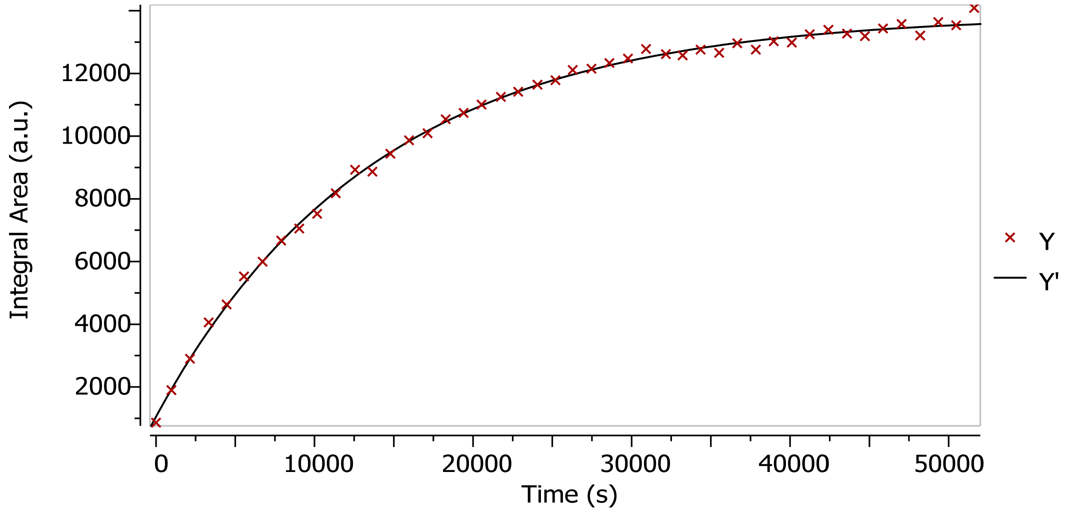

Figure S14 The kinetics data (Y, crosses) and fitted data (Y', line) for **8** (Run 1).

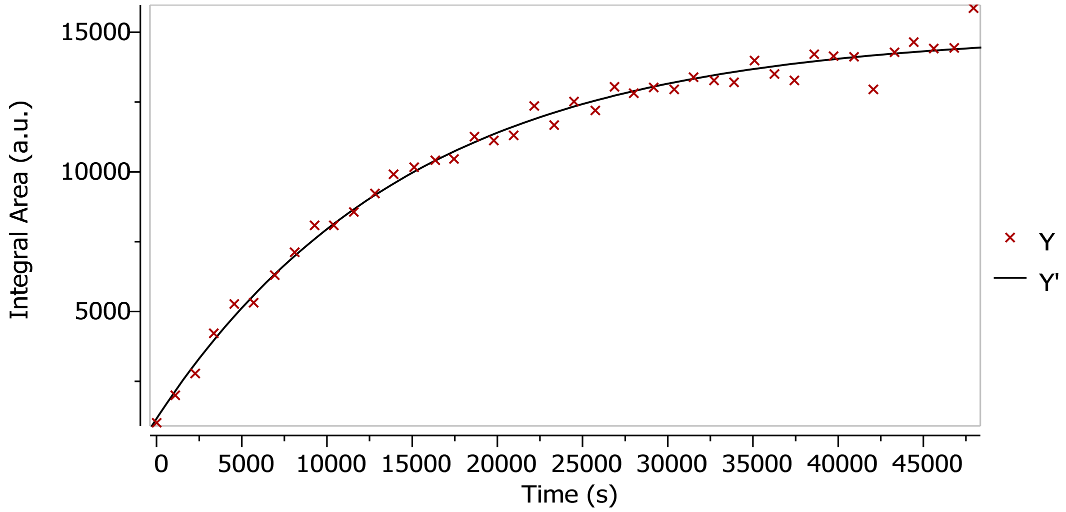

Figure S15 The kinetics data (Y, crosses) and fitted data (Y', line) for **8** (Run 2).

Table S29 Kinetics data for **8**.

Run 1			Run 2		
[**8**]	[iodooctane]	Volume	[**8**]	[iodooctane]	Volume
25.1 mM	509.6 mM	CD_2Cl_2: 500 µL	25.1 mM	509.6 mM	CD_2Cl_2: 500 µL
B= 13870	F= -12820	G= 0.00007244	B= 14980	F= -131810	G= 0.00006758
Time (s)	Integral Area (a.u.)	B+F*exp(-t*G)	Time (s)	Integral Area (a.u.)	B+F*exp(-t*G)
0	856.022	1054.95	0	1023.48	1168.07
971	1889.38	1925.56	1085	2000.34	2144.25

2132	2893.79	2889.24	2258	2784.63	3122.12
3302	4062.21	3781.77	3366	4226.61	3977.25
4454	4629.16	4589.67	4557	5281.57	4827.77
5566	5524.45	5308.1	5711	5327.06	5589.08
6718	5993.1	5993.79	6942	6298.79	6338.33
7895	6671.49	6637.69	8095	7129.85	6985.77
9048	7064.8	7217.41	9287	8088.54	7604.12
10179	7530.97	7740.9	10422	8073.16	8148.37
11348	8175.2	8238.74	11577	8556.89	8660.98
12538	8931.1	8704.02	12806	9223.2	9164.23
13642	8872.87	9101.25	13914	9904.89	9583.46
14802	9443.51	9485.77	15105	10170.3	10000.4
15971	9862.47	9841.92	16344	10425.1	10400
17141	10082	10169.4	17459	10454.9	10732.1
18274	10544.1	10461.2	18670	11254.9	11065.5
19396	10734.7	10727.4	19788	11134.4	11350
20547	11014	10979	20961	11312.4	11626.3
21754	11246.1	11221.2	22180	12363.3	11891.1
22840	11418.9	11421.8	23315	11677	12118.9
24068	11635.1	11630.3	24516	12509.3	12341.5
25199	11787.9	11806.7	25731	12193.3	12549.2
26303	12101	11965.4	26891	13038.1	12732.1
27471	12141	12120.1	27990	12806.8	12892.6
28597	12342.8	12257.3	29191	13026.2	13055
29774	12474.9	12389.3	30363	12952.6	13201.2
30906	12789.3	12506	31527	13377.9	13335.4
32154	12614	12624.1	32718	13272.7	13462.2
33200	12585.3	12715.1	33882	13212.2	13576.7
34359	12771.3	12808.3	35102	13992.8	13687.4
35511	12655.8	12893.5	36247	13497.1	13783.3

36652	12960.9	12971.1	37441	13275.1	13875.7
37851	12768	13046	38593	14222.2	13958
38956	13031.1	13109.6	39738	14133.3	14033.8
40114	12987.3	13170.9	40929	14117.4	14106.6
41257	13256.2	13226.6	42075	12961.4	14171.3
42407	13383.1	13278.2	43313	14281.5	14235.8
43586	13278.9	13326.8	44421	14645.2	14289.2
44710	13194.8	13369.4	45622	14428.9	14342.7
45852	13429.7	13409.3	46794	14445.5	14390.8
47021	13573.5	13446.9	47949	15852.5	14434.7
48190	13215.5	13481.4			
49332	13629.5	13512.4			
50492	13526.6	13541.4			
51615	14090	13567.2			

Run 1: Mass **8** = 9.1 mg, mass iodooctane = 61.2 mg.

Run 2: Mass **8** = 9.1 mg, mass iodooctane = 61.2 mg.

S4.10. Kinetics Data for 9.

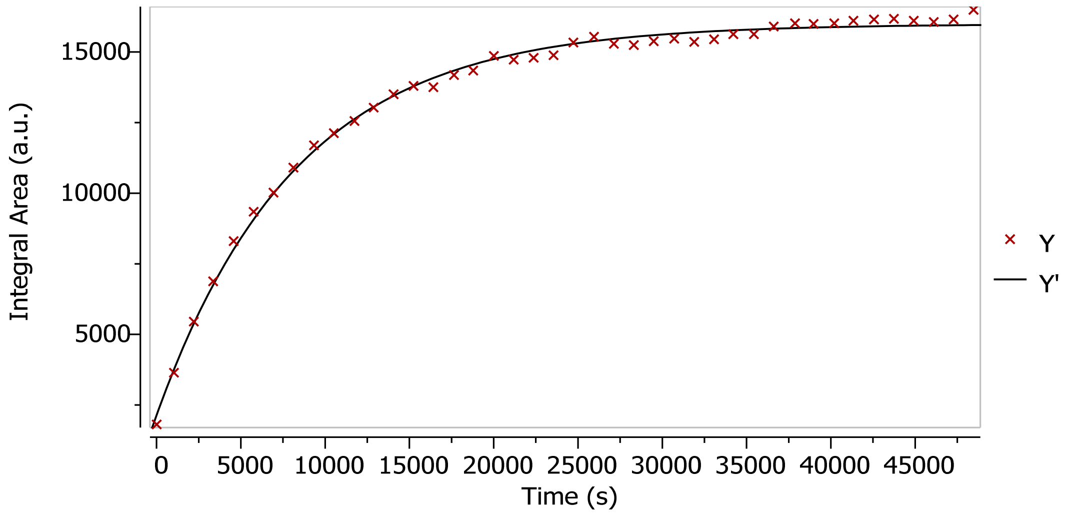

Figure S16 The kinetics data (Y, crosses) and fitted data (Y', line) for **9** (Run 1).

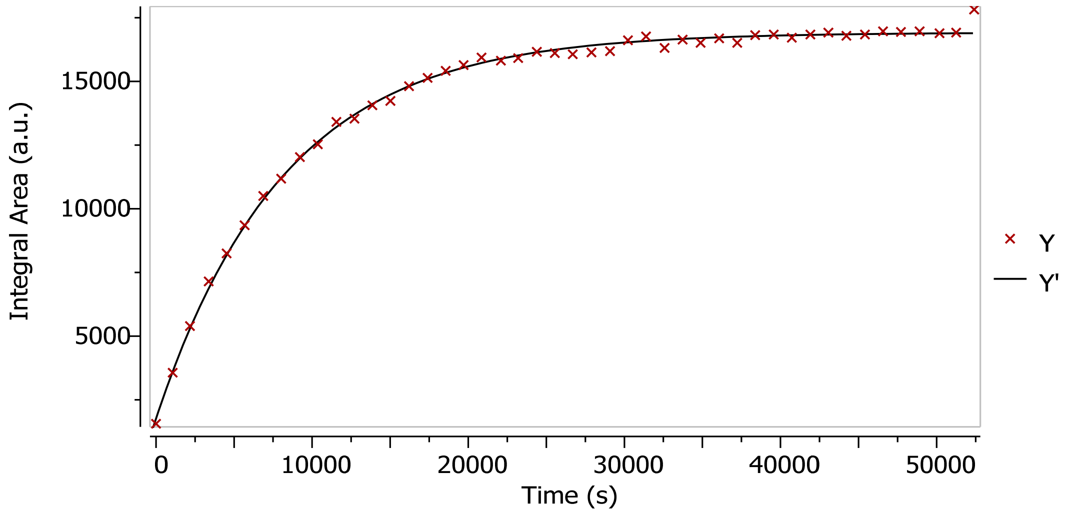

Figure S17 The kinetics data (Y, crosses) and fitted data (Y', line) for **9** (Run 2).

Table S30 Kinetics data for **9**.

Run 1			Run 2		
[**9**] 24.6 mM	[iodooctane] 509.6 mM	Volume CD_2Cl_2: 500 µL	[**9**] 25.2 mM	[iodooctane] 509.6 mM	Volume CD_2Cl_2: 500 µL
B= 15990	F= -13850	G= 0.0001205	B= 16920	F= -15150	G= 0.0001218
Time (s)	Integral Area (a.u.)	B+F*exp(-t*G)	Time (s)	Integral Area (a.u.)	B+F*exp(-t*G)
0	1818.87	2143.68	0	1557.61	1766.66
1046	3649.47	3784.24	1083	3570.38	3639.17
2196	5457.89	5364.52	2196	5390.91	5322.75
3373	6887.32	6770.3	3380	7158.37	6880.11
4569	8313.95	8008.44	4540	8245.8	8202.91
5747	9334.5	9065.32	5689	9345.92	9341.29
6926	10032	9983.02	6858	10513.4	10347
8131	10913.2	10795.4	8026	11192.8	11218.7
9328	11705	11493.7	9213	12023.5	11986.1

10514	12135.6	12092.9	10361	12532.8	12630
11749	12552.2	12632.3	11548	13408.7	13207.5
12870	13041.2	13056.9	12707	13528.8	13696.3
14067	13503.1	13451.3	13857	14072.1	14117.7
15243	13789.5	13787.1	15033	14248.9	14491.7
16422	13747.2	14079.2	16210	14810.1	14816.1
17646	14179.3	14341.6	17398	15133.1	15099.6
18796	14348.4	14555.3	18547	15426.1	15337.4
20010	14858.4	14750.9	19705	15651.9	15545.6
21189	14722.3	14915.3	20865	15943	15726.8
22374	14796	15058.6	22088	15811.4	15892
23562	14884.4	15183.2	23200	15927.8	16022.3
24739	15339.8	15290.2	24405	16156.7	16144.9
25965	15535.6	15386.6	25546	16120.5	16245.6
27114	15285.8	15464.9	26713	16064.1	16335
28292	15242.8	15534.7	27890	16141.2	16413.2
29487	15381.7	15596	29068	16199.9	16481.1
30693	15474.4	15649.6	30254	16627.1	16540.2
31898	15352	15695.9	31385	16773.5	16589.2
33067	15449.7	15734.8	32581	16327.4	16634.1
34227	15631.8	15768.4	33730	16632.3	16671.5
35423	15627.5	15798.4	34881	16518.8	16704.1
36629	15895	15824.6	36085	16703.5	16733.7
37862	16010.5	15847.7	37235	16529.4	16758.1
38993	15991.4	15866.1	38385	16814.1	16779.4
40189	16004.7	15883	39571	16855.6	16798.4
41357	16116.6	15897.3	40729	16729.6	16814.5
42543	16148.3	15909.9	41898	16833.2	16828.6
43731	16184.4	15920.9	43066	16913.8	16840.8
44909	16105.8	15930.3	44234	16790.1	16851.4

46105	16056.6	15938.6	45402	16855.2	16860.6
47292	16160.9	15945.7	46570	16975.6	16868.6
			47737	16951.1	16875.5
			48906	16971.6	16881.5
			50168	16887.4	16887.1
			51242	16920.3	16891.2

Run 1: Mass **9** = 8.1 mg, mass iodooctane = 61.2 mg.

Run 2: Mass **9** = 8.3 mg, mass iodooctane = 61.2 mg.

S4.11. Kinetics Data for 10.

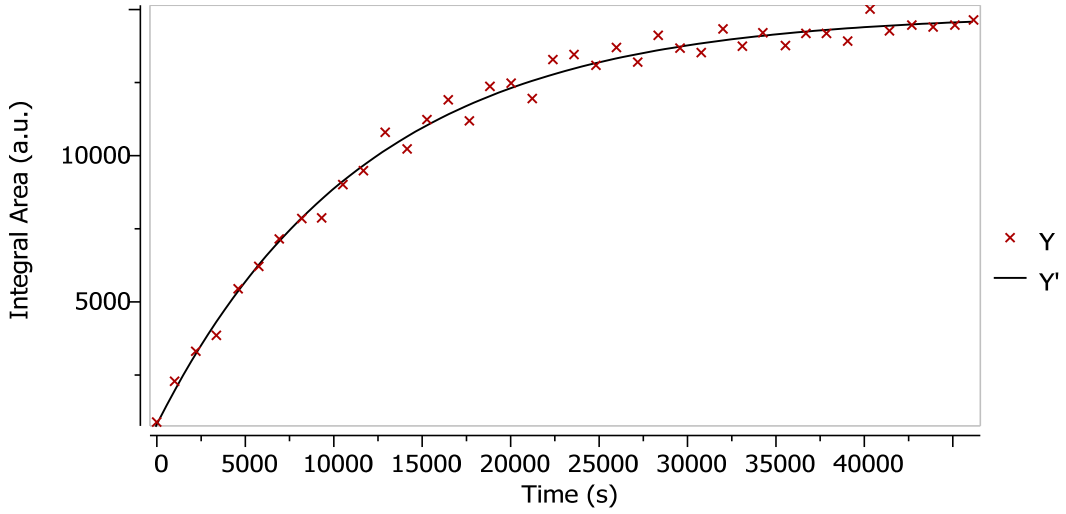

Figure S18 The kinetics data (Y, crosses) and fitted data (Y', line) for **10** (Run 1).

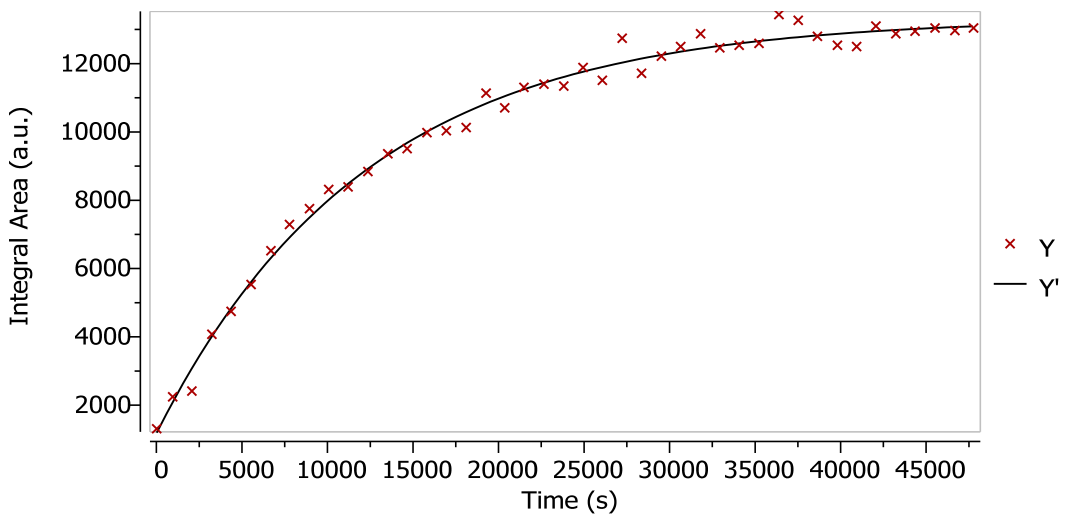

Figure S19 The kinetics data (Y, crosses) and fitted data (Y', line) for **10** (Run 2).

Table S31 Kinetics data for **10**.

Run 1			Run 2		
[**10**] 24.6 mM	[iodooctane] 509.6 mM	Volume CD_2Cl_2: 500 µL	[**10**] 24.9 mM	[iodooctane] 509.6 mM	Volume CD_2Cl_2: 500 µL
B= 14860	F= -14040	G= 0.00008524	B= 13340	F= -12160	G= 0.00008198
Time (s)	Integral Area (a.u.)	B+F*exp(-t*G)	Time (s)	Integral Area (a.u.)	B+F*exp(-t*G)
0	878.894	823.1	0	1312.24	1174.53
1013	2294.85	1984.2	950	2236.67	2085.82
2192	3307.13	3215.18	2093	2420.89	3092.22
3363	3848.07	4321.28	3226	4081.65	4000.95
4580	5448.12	5359.64	4378	4755.56	4842.32
5742	6212.13	6255.44	5521	5524.12	5602.17
6931	7146.36	7084.68	6693	6526.85	6310.77
8186	7857.56	7873.41	7779	7297.27	6909.31
9309	7867.44	8511.1	8932	7757.12	7489.07
10507	9015.71	9127.34	10066	8317.05	8008.25
11685	9499.35	9674.93	11208	8388.55	8484.53
12904	10805.7	10186.6	12370	8839.65	8925.48
14132	10237	10650.9	13522	9360.62	9323.07

15253	11233.1	11034.4	14655	9513.21	9679.15
16460	11912.5	11408.4	15808	9988.1	10009.1
17659	11197.4	11743.6	16942	10033.6	10304.6
18830	12363.3	12039.6	18094	10124.4	10577.9
20018	12488.3	12311.2	19256	11138.6	10828.6
21227	11956	12560.7	20361	10708.1	11045.9
22396	13278.6	12778.7	21496	11305.9	11249.5
23578	13451.7	12978.1	22666	11399.7	11440.5
24813	13094.5	13166.1	23828	11342.3	11612.9
25975	13696.7	13325.7	24925	11886.8	11761.2
27163	13204	13473.4	26085	11505.9	11904.2
28342	14107.6	13605.9	27229	12735.7	12032.5
29561	13690.2	13729.6	28353	11724.2	12147.4
30758	13523.3	13839.2	29525	12221	12256.4
32013	14333.6	13942.7	30667	12507.1	12353
33098	13744.2	14023.7	31820	12869	12441.8
34275	14207.2	14103.4	32934	12465	12520
35514	13758.8	14179.2	34078	12535.6	12593.2
36693	14176.6	14244.2	35240	12593.4	12660.8
37872	14191.9	14303.1	36391	13433.3	12721.7
39042	13929.4	14355.9	37535	13270.2	12776.9
40316	15022	14407.7	38650	12809.1	12825.9
41411	14271.6	14447.9	39821	12530.2	12872.7
42676	14466.5	14490	40945	12498.4	12913.6
43873	14403.9	14525.8	42069	13101.4	12950.9
45101	14460.4	14559	43222	12882.4	12985.8
46158	14648.1	14584.8	44355	12947.1	13017
			45526	13044.4	13046.3
			46678	12973.4	13072.6

	47785	13040.2	13095.6

Run 1: Mass **10** = 8.1 mg, mass iodooctane = 61.2 mg.

Run 2: Mass **10** = 8.2 mg, mass iodooctane = 61.2 mg.

S5. References

Batey, R. A. & Quach, T. D. (2001). *Tetrahedron Letters* **42**, 9099–9103.

Dempsey, S. H. & Kass, S. R. (2022). *J. Org. Chem.* **87**, 15466–15482.

Li, Y., Asadi, A. & Perrin, D. M. (2009). *Journal of Fluorine Chemistry* **130**, 377–382.

Mestrelab Research, (2023), MNova, Santiago de Compostela, Spain.

Petruzziello, D., Gualandi, A., Giaffar, H., Lopez-Carrillo, V. & Cozzi, P. G. (2013). *European Journal of Organic Chemistry* **2013**, 4909–4917.

Van Geet, A. L. (1970). *Anal. Chem.* **42**, 679–680.
